# Salen–scandium(III) complex-catalyzed asymmetric (3 + 2) annulation of aziridines and aldehydes

**DOI:** 10.3762/bjoc.21.86

**Published:** 2025-05-28

**Authors:** Linqiang Wang, Jiaxi Xu

**Affiliations:** 1 State Key Laboratory of Chemical Resource Engineering, Department of Organic Chemistry, College of Chemistry, Beijing University of Chemical Technology, Beijing 100029, People’s Republic of China

**Keywords:** aldehyde, annulation, aziridine, oxazolidine, ring expansion, scandium triflate

## Abstract

Oxazolidine is one of the crucial structural moieties of biologically active compounds. A salen–scandium triflate complex-catalyzed asymmetric (3 + 2) annulation of dialkyl 1-sulfonylaziridine-2,2-dicarboxylates and aldehydes generated optically active functionalized oxazolidine derivatives in moderate to good yields and good to excellent enantioselectivities and high diastereoselectivities. A reasonable reaction mechanism was proposed and rationalized the experimental results.

## Introduction

**Figure 1:**
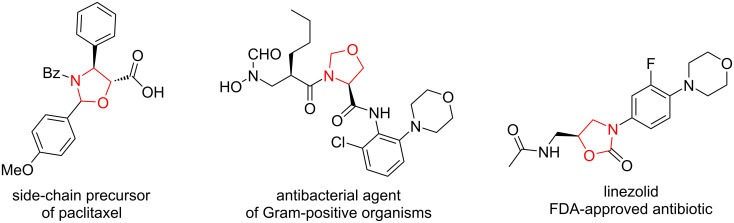
Oxazolidine-containing bioactive compounds.

Oxazolidine derivatives are an important class of nitrogen and oxygen-containing five-membered saturated heterocycles. They are not only useful building blocks for the synthesis of biologically active compounds [1], for example, side-chain precursor of paclitaxel [2], but also widely exist in some pharmaceuticals [3–4], such as antibacterial agents of Gram-positive organisms [5] and the FDA-approved antibiotic linezolid [6] (Figure 1). Both chiral oxazolidines [7–8] and oxazolidinones [9–10] have been utilized as chiral auxiliary groups in many asymmetric organic transformations.

**Scheme 1:**
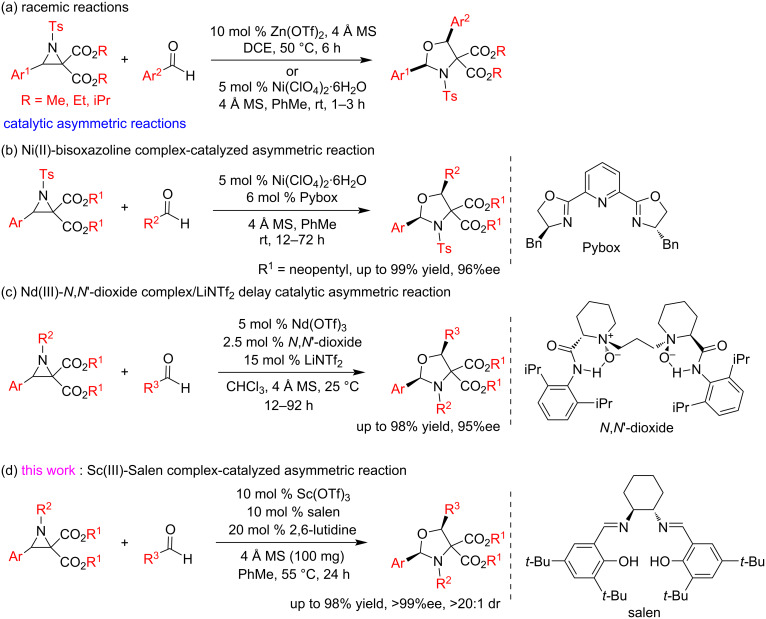
Asymmetric catalytic synthetic methods of oxazolidine derivatives.

Oxazolidine derivatives have been prepared mainly from condensation of vicinal amino alcohols and aldehydes [11] and through [2 + 3] cycloaddition of azomethine ylides and carbonyl dipolarophiles [12]. Recently, the [2 + 3] annulation of aldehydes and donor–acceptor dialkyl 2-aryl-1-sulfonylaziridine-2,2-dicarboxylates, which generate azomethine ylides, has been developed for the synthesis of racemic and optically active functionalized *cis*-2,5-diaryloxazolidine derivatives [13–16]. Racemic *cis*-2,5-diaryloxazolidine derivatives were prepared under the catalysis of zinc triflate or nickel diperchlorate (Scheme 1a) [13–14]. Later, highly enantiomeric *cis*-2,5-diaryloxazolidine derivatives were obtained under asymmetric catalysis with a Ni(II)–bisoxazoline complex [15] and a Nd(OTf)_3_/*N*,*N*'-dioxide/LiNTf_2_ delay catalytic system [16], respectively (Scheme 1b and c). However, further exploration for convenient asymmetric catalytic synthetic methods is still in demand because the former require dineopentyl aziridine-2,2-dicarboxylates to realize high enantioselectivity, while the synthesis of the chiral ligand *N*,*N*'-dioxide requires multiple steps. Herein, we present a convenient highly diastereo- and enantioselective synthesis of dialkyl 2,5-diaryl-1-sulfonyloxazolidine-2,2-dicarboxylates from aldehydes and dialkyl 2-aryl-1-sulfonylaziridine-2,2-dicarboxylates under the catalysis of the readily available salen–Sc(OTf)_3_ complex (Scheme 1d). The salen ligand can be prepared in one step from enantiopure cyclohexane-1,2-diamines and substituted salicylaldehydes.

## Results and Discussion

The reaction of diethyl 3-phenyl-1-(4-toluenesulfonyl)aziridine-2,2-dicarboxylate (**1a**) and benzaldehyde (**2a**) was first selected as a model reaction to optimize the reaction conditions (Table 1). When aziridine **1a** (0.2 mmol) and aldehyde **2a** (0.3 mmol) in dried toluene (1 mL) were stirred at 10 °C for 35 h in the presence of commercially available Sc(OTf)_3_ (0.01 mmol, 5 mol %), only a trace amount of product **3aa** was observed (Table 1, entry 1). When chiral ligand **L1** (0.01 mmol) was added, a trace amount of product **3aa** was obtained with 17% ee (Table 1, entry 2). The enantioselectivity increased to 32% when 2,6-lutidine (0.02 mmol) was added (Table 1, entry 3). When pre-dried Sc(OTf)_3_ was used instead of commercially available one, the reaction generated the desired product **3aa** in 39% yield with 70% ee and >20:1 diastereoselectivity (Table 1, entry 4). The product **3aa** was identified as diethyl (2*R*,5*S*)-2,5-diphenyl-3-tosyloxazolidine-4,4-dicarboxylate on the basis of comparison with reported NMR and specific rotation data [16]. The enantiomeric excess increased to 96% when the amounts of Sc(OTf)_3_ and **L1** were increased to 10 mol %, but the yield remained moderate (38%) (Table 1, entry 5). Other rare-earth salts, including Y(OTf)_3_, La(OTf)_3_, Sm(OTf)_3_, Tb(OTf)_3_, Er(OTf)_3_, and Lu(OTf)_3_, were also screened, but no better results were observed (Table 1, entries 6–11). In addition, different ligands **L2**–**L4** were evaluated (Table 1, entries 12–14). The enantiomer of product **3aa** was obtained in 28% yield with 31% ee and >20:1 diastereomeric ratio in the presence of ligand **L2** (Table 1, entry 12). However, ligands **L3** and **L4** were completely inactive. Thus, further optimizations were performed with 10 mol % of Sc(OTf)_3_ and **L1**. The reaction was conducted at different temperatures for 12 h for saving time. The yield was improved from 21% to 29% to 34% with similar enantio- and diastereoselectivities at 25 °C, 45 °C, and 55 °C, respectively, but the enantioselectivity decreased slightly at 55 °C (Table 1, entries 15–17). Further extending the reaction time to 48 h at 55 °C, resulted in an increased yield of 60%, but the enantioselectivity decreased to 90% (Table 1, entry 18). Solvent screening indicated that toluene was the best choice (Table 1, entries 18–21). Because Sc(OTf)_3_ is moisture sensitive and the reaction is obviously impacted by the quality of Sc(OTf)_3_, Sc(OTf)_3_ was strictly dried at 220 °C for 2 h under an oil pump vacuum and used in further optimizations in toluene with or without simultaneously dried molecular sieves. When the reaction was conducted at 55 °C for 24 h, product **3aa** was obtained in 61% yield and 98%ee in the presences of MS and in 49% yield and 97% ee without MS. Finally, the optimal reaction conditions were selected as: Sc(OTf)_3_ (0.02 mmol) and 4 Å MS (100 mg) were dried at 220 °C for 2 h under oil pump vacuum. 2,6-Lutidine (0.04 mmol), **L1** (0.02 mmol), and dry toluene (1 mL) were added and the mixture was stirred at 55 °C for 3 h. To the solution was added a solution of aziridine **1a** (0.2 mmol) and aldehyde **2a** (0.3 mmol) in dry toluene (1 mL) and the resulting mixture was stirred at 55 °C for 24 h.

**Table 1:** Optimization of reaction conditions.^a^

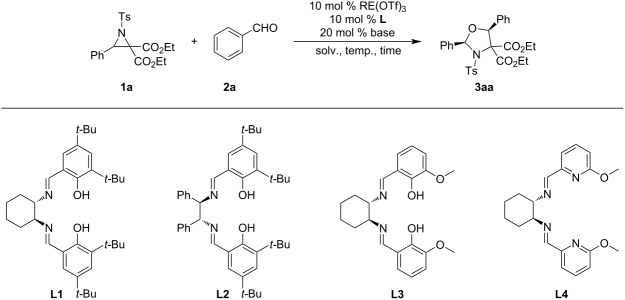									

Entry	Ligand	RE(OTf)_3_	Base	Solv	Temp [°C]	Time [h]	Yield [%]^b^	ee [%] *cis*^c^	dr *cis*/*trans*^d^

1^e^	–	Sc(OTf)_3_	–	PhMe	10	35	trace	–	–
2^e^	**L1**	Sc(OTf)_3_	–	PhMe	10	35	trace	17	–
3^e^	**L1**	Sc(OTf)_3_	lutidine	PhMe	10	35	trace	32	–
4^f^	**L1**	Sc(OTf)_3_	lutidine	PhMe	10	35	39	70	>20:1
5	**L1**	Sc(OTf)_3_	lutidine	PhMe	10	35	38	96	>20:1
6	**L1**	Y(OTf)_3_	lutidine	PhMe	10	35	33	4	60:40
7	**L1**	La(OTf)_3_	lutidine	PhMe	10	35	22	50	79:21
8	**L1**	Sm(OTf)_3_	lutidine	PhMe	10	35	29	0	>19:1
9	**L1**	Tb(OTf)_3_	lutidine	PhMe	10	35	49	0	>20:1
10	**L1**	Er(OTf)_3_	lutidine	PhMe	10	35	31	0	>20:1
11	**L1**	Lu(OTf)_3_	lutidine	PhMe	10	35	46	−6	>20:1
12	**L2**	Sc(OTf)_3_	lutidine	PhMe	10	35	28	−31	>20:1
13	**L3**	Sc(OTf)_3_	lutidine	PhMe	10	35	N.R.	–	–
14	**L4**	Sc(OTf)_3_	lutidine	PhMe	10	35	N.R.	–	–
15	**L1**	Sc(OTf)_3_	lutidine	PhMe	25	12	21	96	>20:1
16	**L1**	Sc(OTf)_3_	lutidine	PhMe	45	12	29	96	>20:1
17	**L1**	Sc(OTf)_3_	lutidine	PhMe	55	12	34	94	>20:1
18	**L1**	Sc(OTf)_3_	lutidine	PhMe	55	48	60	90	>20:1
19	**L1**	Sc(OTf)_3_	lutidine	THF	55	24	14	84	>20:1
20	**L1**	Sc(OTf)_3_	lutidine	MeCN	55	24	19	36	>20:1
21	**L1**	Sc(OTf)_3_	lutidine	DCE	55	24	16	88	>20:1
22^g^	**L1**	Sc(OTf)_3_	lutidine	PhMe	55	24	61	98	>20:1
23^h^	**L1**	Sc(OTf)_3_	lutidine	PhMe	55	24	49	97	>20:1

^a^Unless otherwise noted, the reactions were performed as follows: Sc(OTf)_3_ (9.8 mg, 0.02 mmol) dried in a vacuum drying oven at 106 °C for 12 h, 2,6-lutidine (lutidine for short in Table 1) (4.7 μL, 0.04 mmol), and **L1** (10.9 mg, 0.02 mmol) in solvent (1 mL) were stirred for 3 h. To the solution was added a solution of aziridine **1a** (0.2 mmol) and aldehyde **2a** (0.3 mmol) in solvent (1 mL). ^b^Isolated by flash basic Al_2_O_3_ column chromatography. ^c^Determined by chiral HPLC analysis AD-H, hexane/iPrOH 70:30, flow rate = 0.8 mL/min, λ = 210 nm. ^d^Determined by ^1^H NMR spectroscopy. ^e^Undried Sc(OTf)_3_ was used. ^f^Undried Sc(OTf)_3_ (4.9 mg, 0.01mmol), **L1** (5.5 mg, 0.01 mmol). ^g^Sc(OTf)_3_ and 4 Å molecular sieves (MS) (100 mg) were dried at 220 °C for 2 h under oil pump vacuum and used. ^h^Sc(OTf)_3_ was dried at 220 °C for 2 h under oil pump vacuum.

**Scheme 2:**
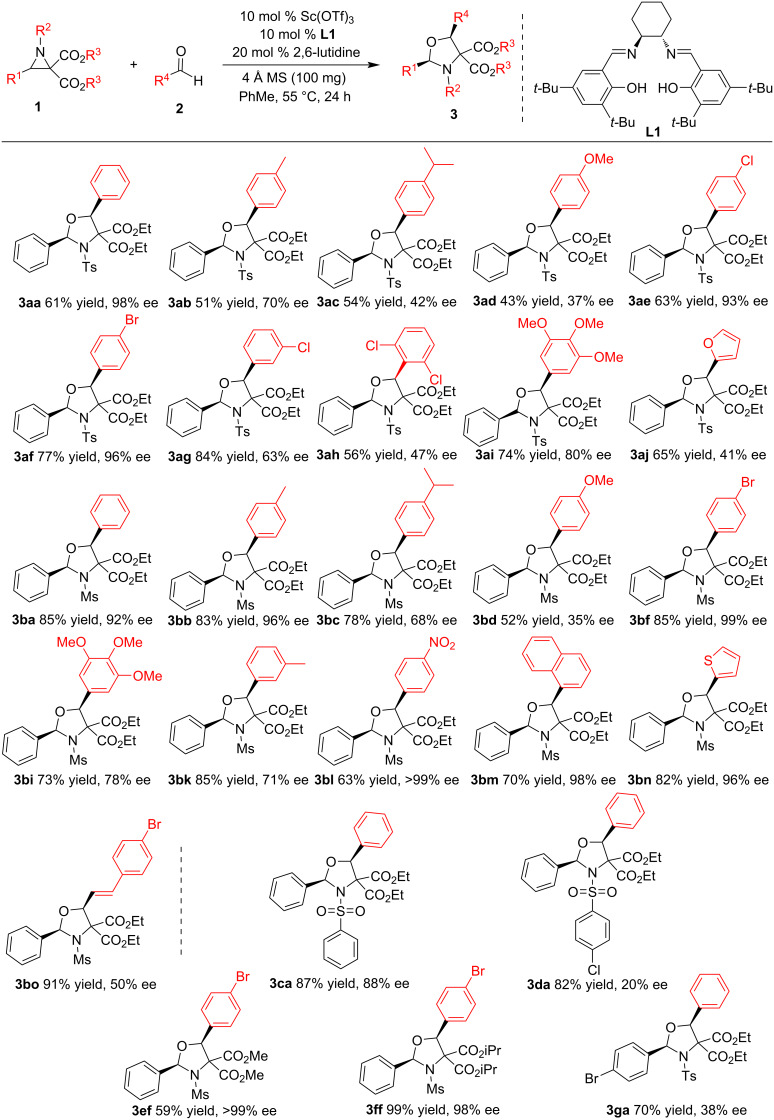
Scope of aziridines and aldehydes.

With the optimal reaction conditions in hand, the scope and generality of both aziridines **1** and aldehydes **2** were investigated (Scheme 2). Different aldehydes **2** were first reacted with diethyl 3-phenyl-1-(4-toluene)sulfonylaziridine-2,2-dicarboxylate (**1a**), affording *cis-*oxazolidines **3aa–aj** in 43–84% yields and 37–98% ee. Obvious influences of electronic and steric effects on both the yields and enantioselectivities were observed. Aromatic aldehydes **2e** and **2f** with electron-withdrawing *para*-substituents generally gave the desired products **3ae** and **3af** in higher yields and enantioselectivities than aldehydes with electron-donating substituents (**2b**–**d**). Sterically congested 3-chlorobenzaldehyde (**2g**) and 2,6-dichlorobenzaldehyde (**2h**) produced the desired products **3ag** and **3ah** in lower yields and enantioselectivities than 4-chlorobenzaldehyde (**2e**). Multisubstituted 3,4,6-trimethoxybenzaldehyde (**2i**) also delivered the corresponding product **3ai** in good yield (74%) and enantioselectivity (80% ee). However, heteroaromatic furan-2-carbaldehyde (**2j**) yielded the expected product **3aj** in moderate yield (65%) and low enantioselectivity (41% ee). The reactions of different aldehydes **2** and diethyl 1-methanesulfonyl-3-phenylaziridine-2,2-dicarboxylate (**1b**) were performed, generating the corresponding oxazolidines **3ba**–**bo** in 52–91% yields and 35–99% ee. Similar influence tendencies of electronic effect on the yield and stereoselectivity were observed as those in the reactions involving **1a**, but the enantioselectivities were generally higher than those in reactions involving **1a**. Electron-rich benzaldehydes **2c** and **2d** and sterically bulky benzaldehydes **2i** and **2k** generated the desired products **3bc**, **3bd**, **3bi**, and **3bk** in relatively low yields and enantioselectivities. The strongly electron-deficient 4-nitrobenzaldehyde (**2l**) showed the highest enantioselectivity, affording the desired product **3bl** in 63% yield and >99% ee. Polycyclic fused naphthalene-1-carbaldehyde (**2m**) and heteroaromatic thiophen-2-carbaldehyde (**2n**) also smoothly underwent reaction, affording the expected products **3bm** in 70% yield and 98% ee and **3bn** in 82% yield and 96% ee, respectively. The reaction could be extended to 4-bromocinnamaldehyde (**2o**), giving the desired product **3bo** in 91% yield and 50% ee. Furthermore, other diethyl 2-phenylaziridine-2,2-dicarboxylates **1c** and **1d** with different 1-arenesulfonyl groups were attempted, giving rise to the corresponding products **3ca** and **3da** in 87% yield with 88% ee and in 82% yield with 20% ee. The results indicate that aziridine **1d** with an electron-deficient 1-(4-chlorophenyl)sulfonyl group presented low enantioselectivity. Next, aziridines **1e** and **1f** with methyl and isopropyl carboxylates also showed excellent enantioselectivities as the corresponding aziridine **1b** with ethyl carboxylate groups. Finally, diethyl 3-(4-bromophenyl)-1-tosylaziridine-2,2-dicarboxylate (**1g**) was attempted as well, delivering the expected product **3ga** in 70% yield and 38% ee, illustrating that the 3-aryl group of aziridines **1** also has an important effect on the enantioselectivity in the (2 + 3) annulation. Compared with previously reported methods, our current method is more convenient and uses readily available components.

**Scheme 3:**
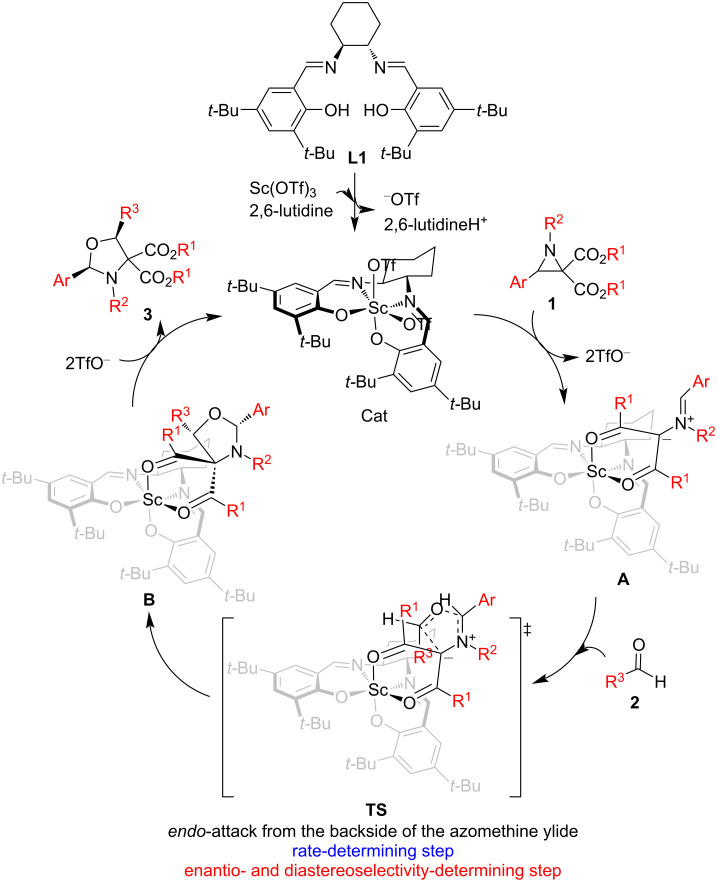
Proposed reaction mechanism.

On the basis of the experimental results and a previous report [16], a possible reaction mechanism is presented in Scheme 3. The catalyst (Cat) is first generated from salen **L1** and Sc(OTf)_3_. Aziridines **1** coordinate to the Sc ion in the catalyst with their two carboxylate groups followed by a ring opening of the aziridine ring, forming azomethine ylide intermediates **A**. The intermediates **A** further undergo a [2 + 3] annulation (or cycloaddition) with aldehydes **2** through *endo* transition state **TS** to generate intermediates **B**, which release products **3** with regeneration of the catalyst for the next catalytic cycle. The electron-deficient aromatic aldehydes exhibit excellent stereoselectivity due to the π-stacking interaction between their aryl group and the electron-rich malonate group. Similar π-stacking interaction-controlled stereoselectivities were observed in our previous studies [17–20].

**Scheme 4:**
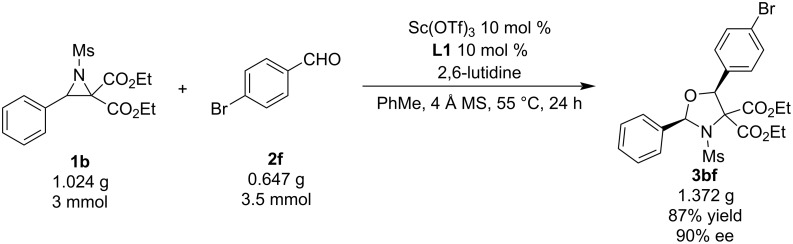
Gram-scale synthesis.

To show the practicality of the reaction, a gram-scale reaction of **1b** and **2f** was carried out, giving the desired product **3bf** in 87% yield (1.372 g) and 90% ee (Scheme 4). The yield was slightly higher than that in the micromolar reaction, but the enantioselectivity obviously was lower compared to the smaller scale reaction.

## Conclusion

A convenient and efficient synthetic method for the synthesis of optically active (2*R*,5*S*)-oxazolidines has been developed with aldehydes and dialkyl 3-aryl-1-sulfonylaziridine-2,2-dicarboxylates as starting materials. The method uses readily available salen as chiral ligand, which coordinates with scandium triflate to generate a salen–Sc complex acting as efficient catalyst. The catalytic asymmetric (3 + 2) annulation of dialkyl 3-aryl-1-sulfonylaziridine-2,2-dicarboxylates and aldehydes generated optically active functionalized oxazolidine derivatives in moderate to good yields and good to excellent enantioselectivities and high diastereoselectivities. The stereocontrol was rationalized in the proposed reaction mechanism.

## Experimental

Unless otherwise noted, all materials were purchased from commercial suppliers. Toluene was refluxed over sodium with benzophenone as an indicator and freshly distilled prior to use. Column chromatography was performed on silica gel (normal phase, 200–300 mesh) from Anhui Liangchen Silicon Material Co., Ltd. or basic aluminum oxide (pH 9–10) from Shanghai Titan Technology Co., Ltd. Petroleum ether (PE, 60–90 °C fraction) and ethyl acetate (EA) were used as eluent. Reactions were monitored by thin-layer chromatography (TLC) on GF254 silica gel plates (0.2 mm) from Anhui Liangchen Silicon Material Co., Ltd. The plates were visualized by UV light. ^1^H NMR (400 MHz) and ^13^C NMR (101 MHz) spectra were recorded on a Bruker 400 NMR spectrometer (Billerica, MA, USA), usually with TMS as an internal standard for ^1^H NMR and the centered peak of CDCl_3_ as an internal standard (77.16) for ^13^C NMR in CDCl_3_ solution. The chemical shifts (δ) were reported in parts per million (ppm) relative to tetramethylsilane (TMS). Melting points were obtained on a melting point apparatus. HRMS measurements were carried out on an LC/MSD TOF mass spectrometer. Specific rotations were measured on an Anton Paar MCP500 polarimeter (Singapore) and are reported as follows: 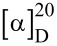 (*c* in g/100 mL, solvent). The enantiomeric excesses were determined using chiral HPLC analysis using an Agilent 1260 LC instrument (Santa Clara, CA, USA) with Daicel Chiralcel AD-H column (Hyderabad, India) with a mixture of isopropyl alcohol and hexane as eluents. All liquid aldehydes were washed with saturated aqueous sodium bicarbonate solution, dried over sodium sulfate, and freshly distilled prior to use. All solid aldehydes were used after recrystallization from petroleum ether (PE, 60–90 °C fraction) or a mixture of ethanol and water. All dialkyl 3-aryl-1-sulfonylaziridine-2,2-dicarboxylates **1** were synthesized by previously reported procedure [16].

### General procedure for the synthesis of ethyl 2-(oxazol-2-yl)alkanoates **3**

Sc(OTf)_3_ (9.8 mg, 0.02 mmol) was added in a dried 10 mL reaction tube, then it was heated to 220 °C and dried at 220 °C for 2 h under oil pump vacuum. After gradually cooling to room temperature, the chiral salen ligand **L1** (10.9 mg, 0.02 mmol), 4 Å MS (100 mg), 2,6-lutidine (4.7 μL, 0.04 mmol), and dry toluene (1 mL) were added. The resulting mixture was stirred at 55 °C for 3 h under a nitrogen atmosphere. A solution of aziridine **1** (0.2 mmol) and aldehyde (0.3 mmol) in 1 mL of dry toluene was added into the mixture with a syringe and the mixture was stirred at 55 °C for 24 h. After gradually cooling to room temperature and removal of the solvent under reduced pressure, the crude residue was purified by basic aluminum oxide column chromatography with petroleum ether/ethyl acetate 1:10 to 3:7 (v/v) as eluent to afford the pure product **3**. The enantiomeric excess was determined using chiral HPLC analysis with a mixture of iPrOH and hexane as eluent.

## Supporting Information

File 1 Analytic data and copies of ^1^H and ^13^C NMR spectra of compounds **1** and **3**, copies of HRMS spectra of unknown compound **3** and copies of HLPC profiles of compounds **3**.

## Data Availability

All data that supports the findings of this study is available in the published article and/or the supporting information of this article.
