# Supplementary material for: Salen–scandium(III) complex-catalyzed asymmetric (3 + 2) annulation of aziridines and aldehydes
Source: Beilstein J Org Chem. 2025 May 28;21:1087–94. doi: 10.3762/bjoc.21.86 (PMC12130625; doi:10.3762/bjoc.21.86)

BEILSTEIN JOURNAL OF ORGANIC CHEMISTRY

Supporting Information

for

Salen–scandium(III) complex-catalyzed asymmetric (3 + 2) annulation of aziridines and aldehydes

Linqiang Wang and Jiaxi Xu

Beilstein J. Org. Chem. **2025,** *21,* 1087–1094. doi:10.3762/bjoc.21.86

Analytic data and copies of ^{1}H and ^{13}C NMR spectra of compounds 1 and 3, copies of HRMS spectra of unknown compound 3 and copies of HLPC profiles of compounds 3

License and Terms: This is a supporting information file under the terms of the Creative Commons Attribution License (https://creativecommons.org/licenses/by/4.0). Please note that the reuse, redistribution and reproduction in particular requires that the author(s) and source are credited and that individual graphics may be subject to special legal provisions.
The license is subject to the *Beilstein Journal of Organic Chemistry* terms and conditions: (https://www.beilstein-journals.org/bjoc/terms)

Table of contents

1. General information S2

2. Starting materials S2

2.1 General procedure for the synthesis of salens **L** S2

2.2 General procedure for the synthesis of aziridines **1** S2

3. General procedure for the synthesis of products **3** S5

4. Experimental procedure for the scale-up reaction S16

5. References S16

6. Copies of NMR and HRMS spectra, and HPLC profiles of products S17

1. General information

Unless otherwise noted, all materials were purchased from commercial suppliers. Toluene was refluxed over sodium with benzophenone as an indicator and freshly distilled prior to use. Column chromatography was performed on silica gel (normal phase, 200–300 mesh) from Anhui Liangchen Silicon Material Co., Ltd. or basic aluminum oxide (pH 9–10) from Shanghai Titan Technology Co., Ltd. Petroleum ether (PE, 60–90 °C fraction) and ethyl acetate (EA) were used as eluent. Reactions were monitored by thin-layer chromatography (TLC) on GF254 silica gel plates (0.2 mm) from Anhui Liangchen Silicon Material Co., Ltd. The plates were visualized by UV light. 1H NMR (400 MHz) and 13C NMR (101 MHz) spectra were recorded on a Bruker 400 NMR spectrometer (Billerica, MA, USA), usually with TMS as an internal standard for 1H NMR and the centered peak of CDCl3 as an internal standard (77.16) for 13C NMR in CDCl3 solution. The chemical shifts (δ) were reported in parts per million (ppm) relative to tetramethylsilane (TMS). Melting points were obtained on a melting point apparatus. HRMS measurements were carried out on an LC/MSD TOF mass spectrometer. Specific rotations were measured on an Anton Paar MCP500 polarimeter (Singapore) and are reported as follows: $[\alpha]_D^{20}$(*c* in g/100 mL, solvent). The enantiomeric excesses were determined using chiral HPLC analysis using an Agilent 1260 LC instrument (Santa Clara, CA, USA) with Daicel Chiralcel AD-H column (Hyderabad, India) with a mixture of isopropyl alcohol and hexane as eluents. All liquid aldehydes were washed with saturated aqueous sodium bicarbonate solution, dried over sodium sulfate, and freshly distilled prior to use. All solid aldehydes were used after recrystallization from petroleum ether (PE, 60–90 °C fraction) or a mixture of ethanol and water.

All the imines were prepared according to literature.[1, 2]

2. Starting materials

2.1 General procedure for the synthesis of salens L

Salen ligands **L1–L4** were prepared according to the literature procedure.[3]

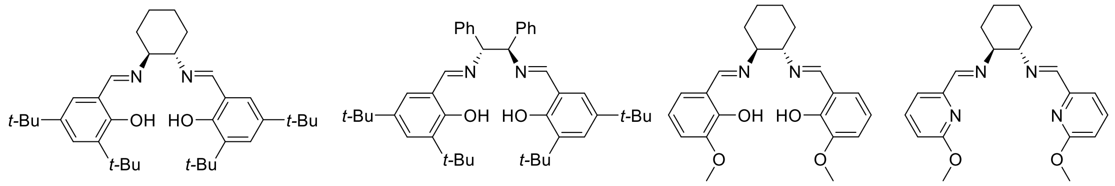

L1 **L2** **L3** **L4**

2.2 General procedure for the synthesis of aziridines 1[4]

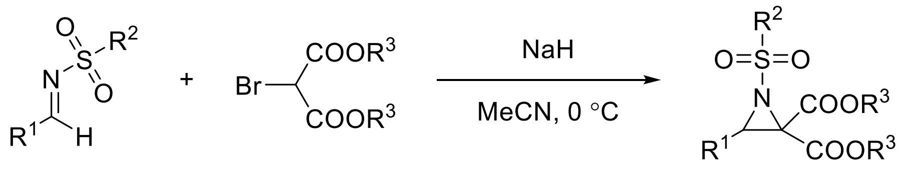

Under nitrogen atmosphere, NaH (60%) (88 mg, 2.2 mmol, 1.1 equiv) and substituted aldimine (2 mmol, 1.0 equiv) were dissolved in dry MeCN (20 mL) in an ice-water bath followed by addition of dialkyl bromomalonate (2.1 mmol, 1.05 equiv). After gradually warming to room temperature, the reaction mixture was stirred for half-hour. The reaction mixture was filtered through celite and silica gel. The filter cake was washed with DCM. The combined organic layer was dried over anhydrous Na_2SO_4. After the evaporation of the solvent under reduced pressure, the crude residue was purified by silica gel column chromatography with petroleum petroleum ether/ethyl acetate (1:10 to 3:7, *v/v*) as eluent to afford pure aziridine **1**.

**Diethyl 3-phenyl-1-tosylaziridine-2,2-dicarboxylate (1a)**
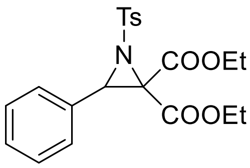
Colorless oil, 88% yield. R_f = 0.31 (PE/EtOAc = 10:1, *v/v*); ^{1}H NMR (400 MHz, $CDCl_3$) δ 7.96 (d, *J* = 8.3 Hz, 2H), 7.35 (d, *J* = 8.1 Hz, 2H), 7.27–7.22 (m, 5H), 4.88 (s, 1H), 4.39 (qd, *J* = 7.1, 1.3 Hz, 2H), 3.95 (q, *J* = 7.1 Hz, 2H), 2.45 (s, 3H), 1.37 (t, *J* = 7.1 Hz, 3H), 0.88 (t, *J* = 7.1 Hz, 3H); ^{13}C NMR (101 MHz, $CDCl_3$) δ 163.1, 162.5, 144.8, 136.5, 131.0, 129.7, 128.8, 128.3, 127.6, 127.0, 63.3, 62.1, 57.5, 49.7, 21.6, 13.7, 13.6.
**Diethyl 1-(methylsulfonyl)-3-phenylaziridine-2,2-dicarboxylate (1b)**
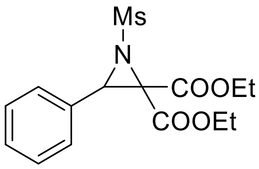
Colorless crystals (Crystallized by ethyl acetate /petroleum ether), 90% yield, m.p. 76–77°C. R_f = 0.3 (PE/EtOAc = 10:1, *v/v*); ^{1}H NMR (400 MHz, $CDCl_3$) δ 7.44–7.29 (m, 5H), 4.77 (s, 1H), 4.36 (qd, *J* = 7.2, 1.6 Hz, 2H), 4.01 (q, *J* = 7.1 Hz, 2H), 3.33 (s, 3H), 1.36 (t, *J* = 7.1 Hz, 3H), 0.93 (t, *J* = 7.1 Hz, 3H); ^{13}C NMR (101 MHz, $CDCl_3$) δ 163.1, 162.5, 130.9, 129.1, 128.6, 127.2, 63.5, 62.5, 57.4, 48.4, 42.0, 13.8, 13.8.
**Diethyl 3-phenyl-1-(phenylsulfonyl)aziridine-2,2-dicarboxylate (1c)**
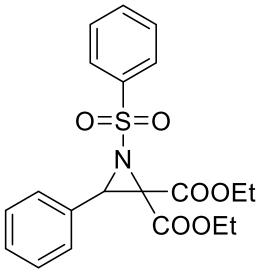
Colorless oil, 82% yield. R_f = 0.33 (PE/EtOAc = 10:1, *v/v*); ^{1}H NMR (400 MHz, $CDCl_3$) δ 8.07 (dd, *J* = 7.4, 1.8 Hz, 2H), 7.67–7.48 (m, 3H), 7.23 (s, 5H), 4.93 (s, 1H), 4.38 (qd, *J* = 7.1, 1.8 Hz, 2H), 3.93 (q, *J* = 7.1 Hz, 2H), 1.34 (t, *J* = 7.2 Hz, 3H), 0.86 (t, *J* = 7.1 Hz, 3H); ^{13}C NMR (101 MHz,

CDCl3) δ 163.1, 162.4, 139.6, 133.9, 130.9, 129.2, 129.0, 128.4, 127.5, 127.0, 63.4, 62.2, 57.6, 49.9, 13.8, 13.6.

Diethyl 1-((4-chlorophenyl)sulfonyl)-3-phenylaziridine-2,2-dicarboxylate (1d)

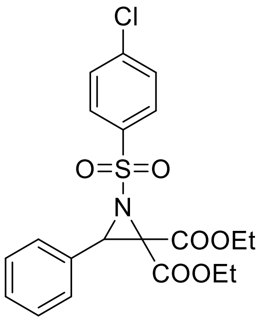

Colorless oil, 79% yield. R_f = 0.32 (PE/EtOAc = 10:1, *v/v*); ^{1}H NMR (400 MHz, $CDCl_3$) δ 8.03 (d, *J* = 8.3 Hz, 2H), 7.53 (d, *J* = 8.2 Hz, 2H), 7.33–7.22 (m, 5H), 4.93 (s, 1H), 4.39 (q, *J* = 7.1 Hz, 2H), 3.96 (q, *J* = 7.1 Hz, 2H), 1.37 (t, *J* = 7.1 Hz, 3H), 0.89 (t, *J* = 7.1 Hz, 3H); ^{13}C NMR (101 MHz, $CDCl_3$) δ 163.0, 162.3, 140.3, 138.2, 130.8, 129.5, 129.10, 129.06, 128.5, 126.1, 63.5, 62.3, 57.7, 50.1, 13.8, 13.6.

Dimethyl 1-(methylsulfonyl)-3-phenylaziridine-2,2-dicarboxylate (1e)

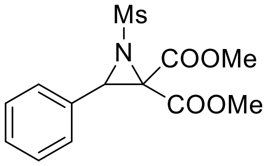

Colorless oil, 95% yield. R_f = 0.10 (PE/EtOAc = 10:1, *v/v*); ^{1}H NMR (400 MHz, $CDCl_3$) δ 7.42–7.30 (m, 5H), 4.76 (s, 1H), 3.87 (s, 3H), 3.53 (s, 3H), 3.30 (s, 3H); ^{13}C NMR (101 MHz, $CDCl_3$) δ 163.2, 162.6, 130.4, 128.9, 128.3, 126.7, 56.8, 53.8, 52.9, 48.1.

Diisopropyl 1-(methylsulfonyl)-3-phenylaziridine-2,2-dicarboxylate (1f)

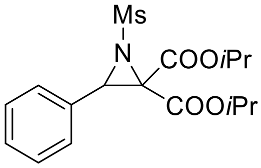

Colorless oil, 81% yield. R_f = 0.26 (PE/EtOAc = 10:1, *v/v*); 1H NMR (400 MHz, $CDCl_3$) δ 7.45–7.28 (m, 5H), 5.21 (heptet, *J* = 6.3 Hz, 1H), 4.84 (heptet, *J* = 6.3 Hz, 1H), 4.75 (s, 1H), 3.32 (s, 3H), 1.34 (dd, *J* = 10.0, 6.3 Hz, 6H), 1.08 (d, *J* = 6.3 Hz, 3H), 0.78 (d, *J* = 6.3 Hz, 3H); ^{13}C NMR (101 MHz, $CDCl_3$) δ 162.5, 161.9, 131.0, 129.1, 128.5, 127.2, 71.6, 70.3, 57.8, 48.4, 42.1, 21.5, 21.3, 21.2.

Diethyl 3-(4-bromophenyl)-1-tosylaziridine-2,2-dicarboxylate (1g)
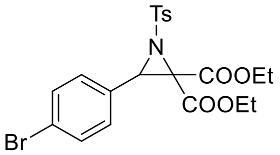
Colorless oil, 78% yield. R_f = 0.28 (PE/EtOAc = 10:1, *v/v*); ^{1}H NMR (400 MHz, $CDCl_3$) δ 7.94 (d, *J* = 6.4 Hz, 2H), 7.9 (d, *J* = 6.4 Hz, 2H), 7.35 (d, *J* = 8.1 Hz, 2H), 7.15–7.09 (m, 2H), 4.81 (s, 1H), 4.39 (qd, *J* = 7.1, 2.3 Hz, 2H), 3.98 (qd, *J* = 7.1, 2.7 Hz, 2H), 2.45 (s, 3H), 1.36 (t, *J* = 7.2 Hz, 3H), 0.95 (t, *J* = 7.1 Hz, 3H); ^{13}C NMR (101 MHz, $CDCl_3$) δ 162.9, 162.3, 145.0, 136.3, 131.6, 130.2, 129.8, 128.8, 127.7, 123.1, 63.5, 62.3, 57.4, 49.0, 21.7, 13.8, 13.7.

3 General procedure for the synthesis of products 3

$Sc(OTf)_3$ (9.8 mg, 0.02 mmol) was added in a dried 10 mL reaction tube, then it was heated to 220 °C and dried at 220 °C for 2 h under oil pump vacuum. After gradually cooling to room temperature, the chiral salen ligand **L1** (10.9 mg, 0.02 mmol), 4 Å MS (100 mg), 2,6-lutidine (4.7 µL, 0.04 mmol), and dry toluene (1 mL) were added. The resulting mixture was stirred at 55 °C for 3 h under nitrogen atmosphere. A solution of aziridine **1** (0.2 mmol) and aldehyde (0.3 mmol) in 1 mL of dry toluene was added into the mixture with a syringe. The mixture was stirred at 55 °C for 24 h. After gradually cooling to room temperature and removal of the solvent under reduced pressure, the crude residue was purified by basic aluminum oxide column chromatography with petroleum ether/ethyl acetate (1:10 to 3:7, *v/v*) as eluent to afford the pure product **3**. The enantiomeric excess was determined using chiral HPLC analysis with a mixture of iPrOH and hexane as eluent.

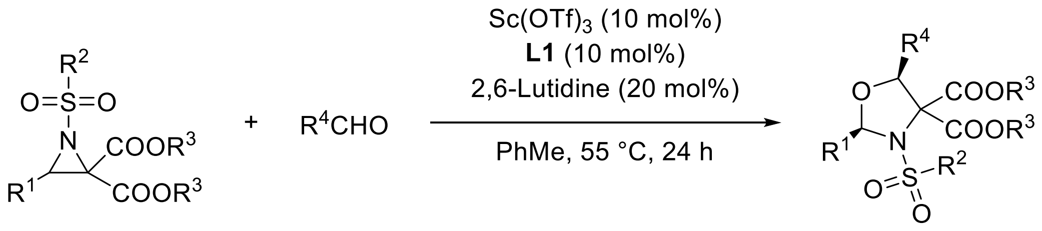

Diethyl (2*R*,5*S*)-2,5-diphenyl-3-tosyloxazolidine-4,4-dicarboxylate (3aa)[5]
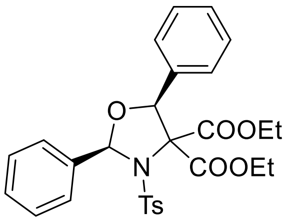
Colorless oil, 61% yield, R_f = 0.30 (PE/EtOAc = 10:1, *v/v*). $[\alpha]_D^{20}$ = +27.2 (*c* = 2.56 in CH_2Cl_2), Lit.[5] $[\alpha]_D^{14}$ = +54.9 (c = 0.39 in CH_2Cl_2). 98% ee (Chiralpak AD-H, hexane/^{i}PrOH = 70/30, flow rate = 0.8 mL/min, λ = 210 nm: t_R (major) = 9.86 min, t_R (minor) = 12.94 min.); ^{1}H NMR (400 MHz, $CDCl_3$) δ 7.51–7.47 (m, 2H), 7.34–7.26 (m, 6H), 7.16 (t, *J* = 7.7 Hz, 4H), 6.90 (d, *J* = 8.1 Hz, 2H), 6.24 (s, 1H), 5.83 (s, 1H), 4.58–4.41 (m, 2H), 3.92 (dq, *J* = 10.6, 7.1 Hz, 1H), 3.50 (dq, *J* = 10.6, 7.2 Hz, 1H), 2.29 (s, 3H), 1.46 (t, *J* = 7.1 Hz, 3H), 0.81 (t, *J* = 7.2 Hz, 3H); ^{13}C NMR (101 MHz, $CDCl_3$) δ 167.5, 166.4, 143.0, 137.8, 134.8, 134.2, 130.0, 129.1, 128.5, 128.33, 128.27, 128.1, 126.7, 93.1, 87.6, 77.1, 63.2, 62.1, 21.6, 14.1, 13.4.

Diethyl (2*R*,5*S*)-2-phenyl-5-(*p*-tolyl)-3-tosyloxazolidine-4,4-dicarboxylate (3ab)[6]

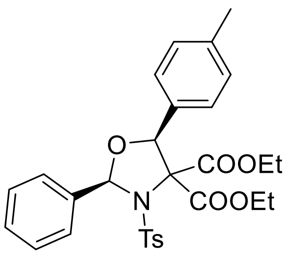

Colorless oil, 51% yield, R_f = 0.31 (PE/EtOAc = 10:1, *v/v*). $[\alpha]_D^{20}$ = +46.6 (*c* = 5.54 in CH_2Cl_2). 70% ee (Chiralpak AD-H, hexane/^{i}PrOH = 70/30, flow rate = 0.8 mL/min, λ = 210 nm: t_R (major) = 8.87 min, t_R (minor) = 10.28 min.); ^{1}H NMR (400 MHz, $CDCl_3$) *δ* 7.54–7.48 (m, 2H), 7.34–7.29 (m, 1H), 7.24 (d, *J* = 8.0 Hz, 2H), 7.22–7.12 (m, 6H), 6.92 (d, *J* = 8.1 Hz, 2H), 6.25 (s, 1H), 5.82 (s, 1H), 4.61–4.41 (m, 2H), 3.96 (dqd, *J* = 11.1, 7.1, 4.0 Hz, 1H), 3.57 (dq, *J* = 10.7, 7.2 Hz, 1H), 2.35 (s, 3H), 2.32 (s, 3H), 1.48 (td, *J* = 7.1, 2.1 Hz, 3H), 0.85 (t, *J* = 7.2 Hz, 3H); ^{13}C NMR (101 MHz, $CDCl_3$) *δ* 167.5, 166.4, 142.9, 138.9, 137.8, 134.2, 131.7, 130.0, 128.9, 128.4, 128.2, 128.0, 126.6, 93.0, 87.6, 77.0, 63.1, 62.1, 21.5, 21.3, 14.1, 13.4.

Diethyl (2*R*,5*S*)-5-(4-isopropylphenyl)-2-phenyl-3-tosyloxazolidine-4,4-dicarboxylate (3ac)

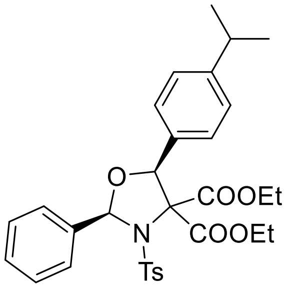

Colorless crystals, m.p. 116–117 °C, 54% yield, R_f = 0.30 (PE/EtOAc = 10:1, *v/v*). $[\alpha]_D^{20}$ = +30.7 (*c* = 5.58 in CH_2Cl_2). 42% ee (Chiralpak AD-H, hexane/^{i}PrOH = 70/30, flow rate = 0.8 mL/min, λ = 210 nm: t_R (major) = 7.58 min, t_R (minor) = 11.04 min.); ^{1}H NMR (400 MHz, $CDCl_3$) *δ* 7.52–7.46 (m, 2H), 7.30–7.23 (m, 3H), 7.20–7.11 (m, 6H), 6.90 (d, *J* = 8.1 Hz, 2H), 6.22 (s, 1H), 5.79 (s, 1H), 4.61–4.36 (m, 2H), 3.92 (dq, *J* = 10.7, 7.1 Hz, 1H), 3.49 (dq, *J* = 10.6, 7.2 Hz, 1H), 2.89 (hept, *J* = 7.0 Hz, 1H), 2.29 (s, 3H), 1.46 (t, *J* = 7.1 Hz, 3H), 1.21 (d, *J* = 6.9 Hz, 6H), 0.78 (t, *J* = 7.1 Hz, 3H); ^{13}C NMR (101 MHz, $CDCl_3$) *δ* 167.6, 166.5, 150.0, 142.9, 137.8, 134.3, 132.1, 130.0, 128.5, 128.3, 128.0, 126.7, 126.4, 93.0, 87.6, 77.2, 63.1, 62.1, 34.1, 24.1, 21.6, 14.1, 13.4; HRMS-ESI (*m/z*): calcd for $C_{31}H_{35}NNaO_7S^+$ $[M + Na]^+$: 588.2027; found 588.2031.

Diethyl (2*R*,5*S*)-5-(4-methoxyphenyl)-2-phenyl-3-tosyloxazolidine-4,4-dicarboxylate (3ad)[7]

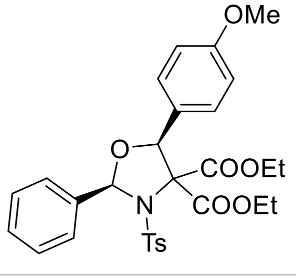

Colorless crystals, m.p. 119–120 °C, 43% yield, R_f = 0.29 (PE/EtOAc = 10:1, *v/v*). $[\alpha]_D^{20}$ = +27.2 (*c* = 4.47 in CH_2Cl_2). 37 % ee (Chiralpak AD-H, hexane/^{i}PrOH = 70/30, flow rate = 0.8 mL/min, λ = 210 nm: t_R (major) = 12.07 min, t_R (minor) = 14.11 min.); ^{1}H NMR (400 MHz, $CDCl_3$) *δ* 7.50–7.46 (m, 2H), 7.31–7.24 (m, 3H),

7.13–7.11 (m, 4H), 6.90 (d, *J* = 8.1 Hz, 2H), 6.85 (d, *J* = 8.8 Hz, 2H), 6.21 (s, 1H), 5.77 (s, 1H), 4.57–4.38 (m, 2H), 3.96 (dq, *J* = 10.7, 7.2 Hz, 1H), 3.78 (s, 3H), 3.57 (dq, *J* = 10.7, 7.2 Hz, 1H), 2.29 (s, 3H), 1.45 (t, *J* = 7.1 Hz, 3H), 0.88 (t, *J* = 7.1 Hz, 3H); ^{13}C NMR (101 MHz, $CDCl_3$) δ 167.5, 166.4, 160.3, 142.9, 137.8, 134.2, 130.0, 128.4, 128.2, 128.0, 126.7, 113.7, 92.9, 87.5, 77.0, 63.1, 62.1, 55.4, 21.5, 14.1, 13.5.

Diethyl (2*R*,5*S*)-5-(4-chlorophenyl)-2-phenyl-3-tosyloxazolidine-4,4-dicarboxylate (3ae)

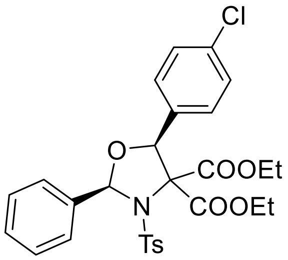

Colorless crystals, m.p. 39–40 °C, 63% yield, R_f = 0.28 (PE/EtOAc = 10:1, *v/v*). $[\alpha]_D^{20}$ = +73.1 (*c* = 4.67 in CH_2Cl_2). 93% ee (Chiralpak AD-H, hexane/^{i}PrOH = 70/30, flow rate = 0.8 mL/min, λ = 210 nm: t_R (major) = 9.04 min, t_R (minor) = 12.53 min.); ^{1}H NMR (400 MHz, $CDCl_3$) δ 7.46 (d, *J* = 7.2 Hz, 2H), 7.35–7.25 (m, 5H), 7.15 (dt, *J* = 14.7, 7.6 Hz, 4H), 6.91 (d, *J* = 8.1 Hz, 2H), 6.22 (s, 1H), 5.80 (s, 1H), 4.58–4.39 (m, 2H), 3.96 (dq, *J* = 10.6, 7.2 Hz, 1H), 3.60 (dq, *J* = 10.7, 7.2 Hz, 1H), 2.30 (s, 3H), 1.45 (t, *J* = 7.2 Hz, 3H), 0.87 (t, *J* = 7.2 Hz, 3H); ^{13}C NMR (101 MHz, $CDCl_3$) δ 167.3, 166.2, 143.1, 137.6, 135.0, 134.0, 133.3, 130.1, 129.9, 128.5, 128.2, 128.1, 128.0, 93.1, 86.7, 76.8, 63.3, 62.2, 21.5, 14.1, 13.5; HRMS-ESI (*m/z*): calcd for $C_{28}H_{28}ClNNaO_7S^+$ $[M + Na]^+$: 580.1168; found 580.1169.

Diethyl (2*R*,5*S*)-5-(4-bromophenyl)-2-phenyl-3-tosyloxazolidine-4,4-dicarboxylate (3af)

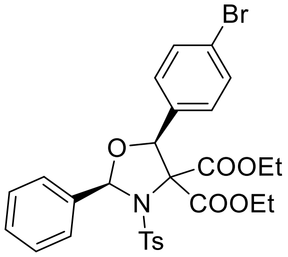

Colorless crystals, m.p. 41–42 °C, 77% yield, R_f = 0.29 (PE/EtOAc = 10:1, *v/v*). $[\alpha]_D^{20}$ = +88.0 (*c* = 5.52 in CH_2Cl_2). 96% ee (Chiralpak AD-H, hexane/^{i}PrOH = 70/30, flow rate = 0.8 mL/min, λ = 210 nm: t_R (major) = 9.20 min, t_R (minor) = 12.98 min.); ^{1}H NMR (400 MHz, $CDCl_3$) δ 7.52–7.40 (m, 4H), 7.30 (t, *J* = 7.4 Hz, 1H), 7.25–7.10 (m, 6H), 6.94–6.86 (m, 2H), 6.22 (s, 1H), 5.78 (s, 1H), 4.58–4.39 (m, 2H), 3.96 (dq, *J* = 10.7, 7.1 Hz, 1H), 3.60 (dq, *J* = 10.7, 7.2 Hz, 1H), 2.30 (s, 3H), 1.45 (t, *J* = 7.2 Hz, 3H), 0.87 (t, *J* = 7.2 Hz, 3H); ^{13}C NMR (101 MHz, $CDCl_3$) δ 167.2, 166.2, 143.0, 137.6, 134.0, 133.8, 131.4, 130.1, 129.9, 128.5, 128.3, 128.2, 128.1, 123.1, 93.1, 86.7, 76.8, 63.3, 62.2, 21.5, 14.1, 13.4; HRMS-ESI (*m/z*): calcd for $C_{28}H_{28}BrNNaO_7S^+$ $[M + Na]^+$: 624.0663; found 624.0662.

Diethyl (2*R*,5*S*)-5-(3-chlorophenyl)-2-phenyl-3-tosyloxazolidine-4,4-dicarboxylate (3ag)

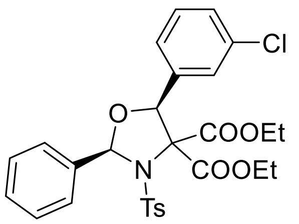

Colorless oil, 84% yield, R_f = 0.28 (PE/EtOAc = 10:1, *v/v*). $[\alpha]_D^{20}$ = +35.5 (*c* = 8.89 in CH_2Cl_2). 63% ee (Chiralpak AD-H, hexane/^{i}PrOH = 70/30, flow rate = 0.8 mL/min, λ = 210 nm: t_R (major) = 11.48 min, t_R (minor) = 13.04 min.); ^{1}H NMR (400 MHz, $CDCl_3$) δ 7.51–7.42 (m, 2H), 7.35–7.27 (m, 4H), 7.24–7.11 (m, 5H), 6.94–6.86 (m, 2H), 6.22 (s, 1H), 5.79 (s, 1H), 4.60–4.38 (m, 2H), 3.96 (dq, *J* = 10.7, 7.1 Hz, 1H), 3.62 (dq, *J* = 10.7, 7.2 Hz, 1H), 2.29 (s, 3H), 1.45 (t, *J* = 7.1 Hz, 3H), 0.87 (t, *J* = 7.2 Hz, 3H); ^{13}C NMR (101 MHz, $CDCl_3$) δ 167.2, 166.1, 143.1, 137.6, 136.7, 134.4, 133.9, 130.1, 129.9, 129.6, 129.1, 128.5, 128.2, 128.1, 126.7, 125.0, 93.1, 86.5, 77.4, 63.3, 62.1, 21.5, 14.1, 13.4. HRMS-ESI (*m/z*): calcd for $C_{28}H_{28}ClNNaO_7S^+$ $[M + Na]^+$: 580.1168; found 580.1165.

Diethyl (2*R*,5*S*)-5-(2,6-dichlorophenyl)-2-phenyl-3-tosyloxazolidine-4,4-dicarboxylate (3ah)

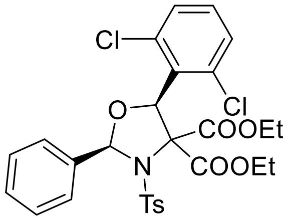

Colorless oil, 56% yield, R_f = 0.28 (PE/EtOAc = 10:1, *v/v*). $[\alpha]_D^{20}$ = +47.3 (*c* = 3.18 in CH_2Cl_2). 47% ee (Chiralpak AD-H, hexane/^{i}PrOH = 70/30, flow rate = 0.8 mL/min, λ = 210 nm: t_R (major) = 9.79 min, t_R (minor) = 12.58 min.); ^{1}H NMR (400 MHz, $CDCl_3$) δ 7.49 (dd, *J* = 8.2, 1.4 Hz, 2H), 7.36–7.28 (m, 5H), 7.19–7.12 (m, 3H), 6.90 (d, *J* = 8.0 Hz, 2H), 6.24 (s, 1H), 5.83 (s, 1H), 4.61–4.40 (m, 2H), 3.92 (dq, *J* = 10.6, 7.1 Hz, 1H), 3.50 (dq, *J* = 10.6, 7.2 Hz, 1H), 2.29 (s, 3H), 1.46 (t, *J* = 7.1 Hz, 3H), 0.80 (t, *J* = 7.2 Hz, 3H); ^{13}C NMR (101 MHz, $CDCl_3$) δ 167.4, 166.3, 142.8, 137.7, 134.6, 134.1, 129.9, 129.0, 128.8, 128.3, 128.21, 128.15, 127.9, 126.6, 92.9, 87.4, 77.3, 63.1, 62.0, 21.5, 14.0, 13.3, HRMS-ESI (*m/z*): calcd for $C_{28}H_{27}Cl_2NNaO_7S^+$ $[M + Na]^+$: 614.0778; found 614.0773.

Diethyl (2*R*,5*S*)-2-phenyl-3-tosyl-5-(3,4,5-trimethoxyphenyl)oxazolidine-4,4-dicarboxylate (3ai)[8]

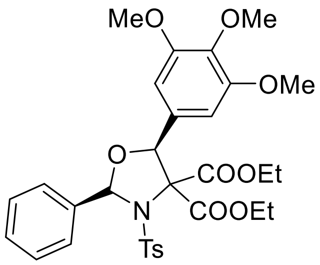

Colorless oil, 74% yield, R_f = 0.25 (PE/EtOAc = 10:1, *v/v*). $[\alpha]_D^{20}$ = +37.8 (*c* = 7.48 in CH_2Cl_2). 80% ee (Chiralpak AD-H, hexane/^{i}PrOH = 70/30, flow rate = 0.8 mL/min, λ = 210 nm: t_R (major) = 19.10 min, t_R (minor) = 27.74 min.); ^{1}H NMR (400 MHz, $CDCl_3$) δ 7.49 (d, *J* = 8.0 Hz, 2H), 7.35–7.28 (m, 1H), 7.22–7.07 (m, 4H), 6.91 (d, *J* = 8.0 Hz, 2H), 6.57 (s, 2H), 6.23 (s, 1H), 5.78 (s, 1H), 4.59–4.38 (m, 2H), 4.07–3.94

(m, 1H), 3.81 (dd, J = 7.9, 1.3 Hz, 9H), 3.60 (dqd, J = 10.7, 7.2, 1.2 Hz, 1H), 2.29 (s, 3H), 1.45 (td, J = 7.2, 1.2 Hz, 3H), 0.90 (td, J = 7.2, 1.2 Hz, 3H); ^{13}C NMR (101 MHz, $CDCl_3$) δ 167.4, 166.3, 153.2, 142.9, 138.5, 137.6, 134.2, 130.1, 129.9, 129.8, 128.4, 128.1, 128.0, 103.6, 93.0, 87.3, 77.3, 63.1, 62.0, 60.8, 56.2, 21.5, 14.0, 13.5.

Diethyl (2*R*,5*S*)-5-(furan-2-yl)-2-phenyl-3-tosyloxazolidine-4,4-dicarboxylate (3aj)

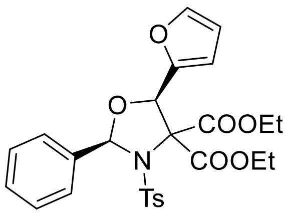

Colorless oil, 65% yield, R_f = 0.29 (PE/EtOAc = 10:1, *v/v*). $[\alpha]_D^{20}$ = +9.6 (c = 5.57 in CH_2Cl_2). 41% ee (Chiralpak AD-H, hexane/^{i}PrOH = 70/30, flow rate = 0.8 mL/min, λ = 210 nm: t_R (major) = 9.95 min, t_R (minor) = 16.14 min.); ^{1}H NMR (400 MHz, $CDCl_3$) δ 7.53–7.36 (m, 3H), 7.26–7.07 (m, 5H), 6.90 (d, J = 8.0 Hz, 2H), 6.48–6.30 (m, 2H), 6.21 (s, 1H), 5.85 (s, 1H), 4.57–4.36 (m, 2H), 4.19 (dq, J = 10.6, 7.1 Hz, 1H), 3.81 (dq, J = 10.6, 7.2 Hz, 1H), 2.29 (s, 3H), 1.44 (t, J = 7.2 Hz, 3H), 1.07 (t, J = 7.1 Hz, 3H); ^{13}C NMR (101 MHz, $CDCl_3$) δ 167.2, 166.3, 147.6, 143.2, 143.0, 137.5, 134.0, 129.9, 129.8, 128.34, 128.31, 128.0, 110.6, 109.8, 93.1, 81.8, 75.8, 63.3, 62.5, 21.5, 13.9, 13.7; HRMS-ESI (*m/z*): calcd for $C_{26}H_{27}NNaO_8S^+$ $[M + Na]^+$: 536.1350; found 536.1351.

Diethyl (2*R*,5*S*)-3-(methylsulfonyl)-2,5-diphenyloxazolidine-4,4-dicarboxylate (3ba)[5]

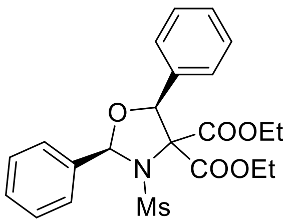

Colorless oil, 85% yield, R_f = 0.30 (PE/EtOAc = 10:1, *v/v*). $[\alpha]_D^{20}$ = +14.9 (c = 6.82 in CH_2Cl_2), Lit.[5] $[\alpha]_\lambda^{31}$ = +58.9 (c = 0.43 in CH_2Cl_2, λ = 365 nm). 92% ee (Chiralpak AD-H, hexane/^{i}PrOH = 70/30, flow rate = 0.8 mL/min, λ = 210 nm: t_R (major) = 6.93 min, t_R (minor) = 14.87 min.); ^{1}H NMR (400 MHz, $CDCl_3$) δ 7.82–7.75 (m, 2H), 7.54–7.45 (m, 3H), 7.36 (s, 5H), 6.24 (s, 1H), 5.81 (s, 1H), 4.50–4.31 (m, 2H), 3.96 (dq, J = 10.6, 7.1 Hz, 1H), 3.58 (dq, J = 10.6, 7.2 Hz, 1H), 2.48 (s, 3H), 1.39 (t, J = 7.2 Hz, 3H), 0.79 (t, J = 7.2 Hz, 3H); ^{13}C NMR (101 MHz, $CDCl_3$) δ 167.2, 167.0, 134.7, 134.6, 130.7, 129.8, 129.1, 128.6, 128.4, 126.5, 92.4, 87.7, 77.1, 63.2, 62.2, 43.1, 14.0, 13.3.

Diethyl (2*R*,5*S*)-3-(methylsulfonyl)-2-phenyl-5-(4-tolyl)oxazolidine-4,4-dicarboxylate (3bb)

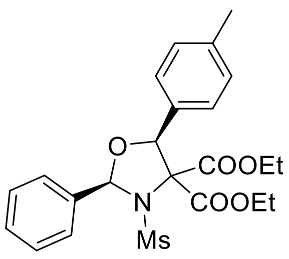

Colorless oil, 83% yield, R_f = 0.31 (PE/EtOAc = 10:1, *v/v*). $[\alpha]_D^{20}$ = +22.5 (*c* = 6.51 in CH_2Cl_2). 96% ee (Chiralpak AD-H, hexane/^{i}PrOH = 70/30, flow rate = 0.8 mL/min, λ = 210 nm: t_R (major) = 7.35 min, t_R (minor) = 11.13 min.); ^{1}H NMR (400 MHz, $CDCl_3$) *δ* 7.82–7.76 (m, 2H), 7.51–7.44 (m, 3H), 7.26–7.11 (m, 4H), 6.23 (s, 1H), 5.78 (s, 1H), 4.49–4.30 (m, 2H), 3.96 (dq, *J* = 10.6, 7.1 Hz, 1H), 3.61 (dq, *J* = 10.6, 7.2 Hz, 1H), 2.47 (s, 3H), 2.35 (s, 3H), 1.38 (t, *J* = 7.1 Hz, 3H), 0.82 (t, *J* = 7.1 Hz, 3H); ^{13}C NMR (101 MHz, $CDCl_3$) *δ* 167.2, 167.0, 139.0, 134.8, 131.6, 130.7, 129.8, 129.0, 128.6, 126.4, 92.3, 87.8, 77.1, 63.2, 62.2, 43.1, 21.3, 14.0, 13.3; HRMS-ESI (*m/z*): calcd for $C_{23}H_{27}NNaO_7S^+$ $[M + Na]^+$: 484.1401; found 484.1400.

Diethyl (2*R*,5*S*)-5-(4-isopropylphenyl)-3-(methylsulfonyl)-2-phenyloxazolidine-4,4-dicarboxylate (3bc)

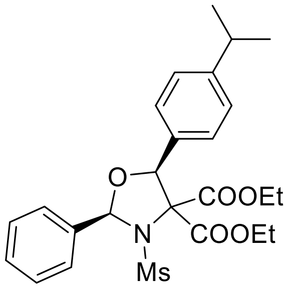

Colorless oil, 78% yield, 68% ee. $[\alpha]_D^{20}$ = +14.3 (*c* = 7.22 in CH_2Cl_2). (Chiralpak AD-H, hexane/^{i}PrOH = 70/30, flow rate = 0.8 mL/min, λ = 210 nm: t_R (major) = 6.88 min, t_R (minor) = 9.57 min.)R_f = 0.30 (PE/EtOAc = 10:1, *v/v*); ^{1}H NMR (400 MHz, $CDCl_3$) *δ* 7.82 – 7.76 (m, 2H), 7.51 – 7.45 (m, 3H), 7.29 (d, *J* = 8.2 Hz, 2H), 7.22 (d, *J* = 8.1 Hz, 2H), 6.23 (s, 1H), 5.78 (s, 1H), 4.49 – 4.30 (m, 2H), 3.94 (dq, *J* = 10.6, 7.1 Hz, 1H), 3.57 (dq, *J* = 10.6, 7.1 Hz, 1H), 2.91 (p, *J* = 6.9 Hz, 1H), 2.47 (s, 3H), 1.39 (t, *J* = 7.2 Hz, 3H), 1.24 (d, *J* = 6.9 Hz, 6H), 0.77 (t, *J* = 7.2 Hz, 3H); ^{13}C NMR (101 MHz, $CDCl_3$) *δ* 167.2, 167.0, 150.0, 134.7, 132.0, 130.6, 129.7 , 128.5 , 126.44 , 126.38 , 92.3, 87.7, 77.06, 63.1, 62.1, 43.0, 34.0, 24.0 , 14.0, 13.2; HRMS-ESI (*m/z*): calcd for $C_{25}H_{31}NNaO_7S^+$ $[M + Na]^+$: 512.1714; found 512.1716.

Diethyl (2*R*,5*S*)-5-(4-methoxyphenyl)-3-(methylsulfonyl)-2-phenyloxazolidine-4,4-dicarboxylate (3bd)

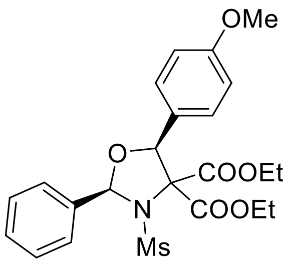

Colorless oil, 52% yield, R_f = 0.28 (PE/EtOAc = 10:1, *v/v*). $[\alpha]_D^{20}$ = +11.3 (*c* = 4.79 in CH_2Cl_2). 35% ee (Chiralpak AD-H, hexane/^{i}PrOH = 70/30, flow rate = 0.8 mL/min, λ = 210 nm: t_R (major) = 9.13 min, t_R (minor) = 16.30 min.); ^{1}H NMR (400 MHz, $CDCl_3$) *δ* 7.83–7.73 (m, 2H), 7.52–7.44 (m, 3H), 7.35–7.27 (m, 2H), 6.96–6.85 (m, 2H), 6.22 (s, 1H), 5.76 (s, 1H), 4.49–4.30 (m, 2H), 3.99 (dq, *J* = 10.6, 7.1 Hz, 1H), 3.81 (s, 3H), 3.65 (dq, *J* = 10.6, 7.2 Hz, 1H), 2.47 (s, 3H), 1.39 (t, *J* = 7.1 Hz, 3H), 0.87 (t, *J* = 7.1 Hz, 3H); ^{13}C NMR (101 MHz, $CDCl_3$) *δ* 167.3, 167.0, 160.4, 134.8, 130.7, 129.8, 128.6, 127.9, 126.7, 113.8, 92.3, 87.7, 77.0, 63.2, 62.3, 55.5, 43.1, 14.1, 13.5; HRMS-ESI (*m/z*): calcd for $C_{23}H_{27}NNaO_8S^+$ $[M + Na]^+$: 500.1350; found 500.1354.

Diethyl (2*R*,5*S*)-5-(4-bromophenyl)-3-(methylsulfonyl)-2-phenyloxazolidine-4,4-dicarboxylate (3bf)

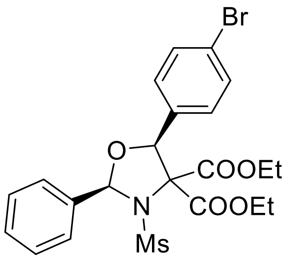

Colorless oil, 85% yield, R_f = 0.28 (PE/EtOAc = 10:1, *v/v*). $[\alpha]_D^{20}$ = +23.8 (*c* = 7.00 in CH_2Cl_2). 99% ee (Chiralpak AD-H, hexane/^{i}PrOH = 70/30, flow rate = 0.8 mL/min, λ = 210 nm: t_R (major) = 8.66 min, t_R (minor) = 15.78 min.); ^{1}H NMR (400 MHz, $CDCl_3$) *δ* 7.79–7.71 (m, 2H), 7.57–7.42 (m, 5H), 7.28–7.22 (m, 2H), 6.23 (s, 1H), 5.77 (s, 1H), 4.50–4.29 (m, 2H), 3.99 (dq, *J* = 10.6, 7.2 Hz, 1H), 3.68 (dq, *J* = 10.7, 7.1 Hz, 1H), 2.48 (s, 3H), 1.38 (t, *J* = 7.1 Hz, 3H), 0.87 (t, *J* = 7.2 Hz, 3H); ^{13}C NMR (101 MHz, $CDCl_3$) *δ* 167.0, 166.8, 134.6, 133.7, 131.6, 130.8, 129.7, 128.7, 128.2, 123.2, 92.5, 87.0, 77.4, 63.4, 62.4, 43.1, 14.0, 13.4; HRMS-ESI (*m/z*): calcd for $C_{22}H_{24}BrNNaO_7S^+$ $[M + Na]^+$: 548.0350; found 548.0354.

Diethyl (2*R*,5*S*)-3-(methylsulfonyl)-2-phenyl-5-(3,4,5-trimethoxyphenyl)oxazolidine-4,4-dicarboxylate (3bi)

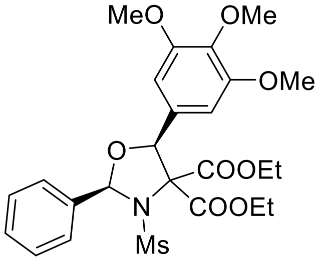

Colorless oil, 73% yield, 78% ee. $[\alpha]_D^{20}$ = +3.7 (*c* = 7.48 in CH_2Cl_2). (Chiralpak AD-H, hexane/^{i}PrOH = 70/30, flow rate = 0.8 mL/min, λ = 210 nm: t_R (major) = 10.80 min, t_R (minor) = 21.64 min.)R_f = 0.25 (PE/EtOAc = 10:1, *v/v*); ^{1}H NMR (400 MHz, $CDCl_3$) *δ* 7.84 – 7.71 (m, 2H), 7.53 – 7.45 (m, 3H), 6.60 (s, 2H), 6.24 (s, 1H), 5.75 (s, 1H), 4.40 (ddd, *J* = 38.1, 10.7, 7.1 Hz, 2H), 4.08 – 3.96 (m, 1H), 3.85 (d, *J* = 1.7 Hz, 9H), 3.71 – 3.66 (m, 1H), 2.49 (s, 3H), 1.39 (t, *J* = 7.1 Hz, 3H), 0.91 (t, *J* = 7.2 Hz, 3H); ^{13}C NMR (101 MHz, $CDCl_3$) *δ* 167.3, 167.0, 153.4, 138.7, 134.7, 130.7, 130.1, 129.7 , 128.7 , 103.7, 92.4, 87.6, 77.4, 63.2, 62.3, 60.9, 58.5, 56.3, 43.1, 18.5, 14.1, 13.5; HRMS-ESI (*m/z*): calcd for $C_{25}H_{31}NNaO_{10}S$ $^+$ $[M + Na]^+$: 560.1561; found 560.1564.

Diethyl (2*R*,5*S*)-3-(methylsulfonyl)-2-phenyl-5-(3-tolyl)oxazolidine-4,4-dicarboxylate (3bk)

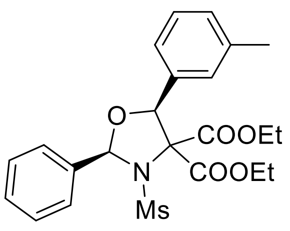

Colorless oil, 85% yield, R_f = 0.30 (PE/EtOAc = 10:1, *v/v*). $[\alpha]_D^{20}$ = +9.5 (*c* = 4.78 in CH_2Cl_2). 71% ee (Chiralpak AD-H, hexane/^{i}PrOH = 70/30, flow rate = 0.8 mL/min, λ = 210 nm: t_R (major) = 6.43 min, t_R (minor) = 9.41 min.); ^{1}H NMR (400 MHz, $CDCl_3$) *δ* 7.83–7.74 (m, 2H), 7.52–7.45 (m, 3H), 7.38–7.12 (m,

4H), 6.23 (s, 1H), 5.77 (s, 1H), 4.51–4.30 (m, 2H), 3.96 (dq, J = 10.6, 7.1 Hz, 1H), 3.60 (dq, J = 10.6, 7.2 Hz, 1H), 2.48 (s, 3H), 2.35 (s, 3H), 1.39 (t, J = 7.1 Hz, 3H), 0.81 (t, J = 7.2 Hz, 3H); ^{13}C NMR (101 MHz, $CDCl_3$) δ 167.2, 167.0, 138.1, 134.7, 134.5, 130.7, 129.9, 129.8, 128.6, 128.3, 127.1, 123.7, 92.4, 87.8, 77.1, 63.2, 62.1, 43.1, 21.5, 14.0, 13.3; HRMS-ESI (*m/z*): calcd for $C_{23}H_{27}NNaO_7S^+$ [M + Na]$^+$: 484.1401; found 484.1402.

Diethyl (2*R*,5*S*)-3-(methylsulfonyl)-5-(4-nitrophenyl)-2-phenyloxazolidine-4,4-dicarboxylate (3bl)

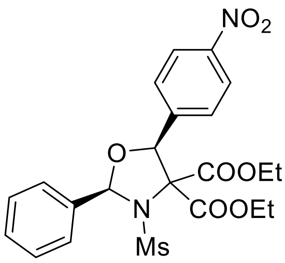

Colorless oil, 63% yield, R_f = 0.20 (PE/EtOAc = 10:1, *v/v*). $[\alpha]_D^{20}$ = +19.2 (c = 5.89 in CH_2Cl_2). > 99% ee (Chiralpak AD-H, hexane/^{i}PrOH = 70/30, flow rate = 0.8 mL/min, λ = 210 nm: t_R (major) = 17.26 min, t_R (minor) = 42.69 min.); ^{1}H NMR (400 MHz, $CDCl_3$) δ 8.31–8.21 (m, 2H), 7.81–7.69 (m, 2H), 7.60–7.35 (m, 5H), 6.26 (s, 1H), 5.91 (s, 1H), 4.52–4.32 (m, 2H), 4.01 (dq, J = 10.8, 7.1 Hz, 1H), 3.71 (dq, J = 10.7, 7.1 Hz, 1H), 2.51 (s, 3H), 1.39 (t, J = 7.1 Hz, 3H), 0.85 (t, J = 7.1 Hz, 3H); ^{13}C NMR (101 MHz, $CDCl_3$) δ 166.7, 166.6, 148.3, 141.7, 134.3, 131.0, 129.6, 128.8, 127.5, 123.5, 92.7, 86.3, 76.7, 63.8, 62.5, 43.1, 14.0, 13.5; HRMS-ESI (*m/z*): calcd for $C_{22}H_{24}N_2NaO_9S^+$ [M + Na]$^+$: 515.1095; found 515.1093.

Diethyl (2*R*,5*S*)-3-(methylsulfonyl)-5-(naphthalen-1-yl)-2-phenyloxazolidine-4,4-dicarboxylate (3bm)

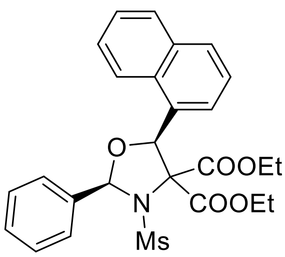

Colorless oil, 70% yield, R_f = 0.32 (PE/EtOAc = 10:1, *v/v*). $[\alpha]_D^{20}$ = +62.1 (c = 4.66 in CH_2Cl_2). 98% ee (Chiralpak AD-H, hexane/^{i}PrOH = 70/30, flow rate = 0.8 mL/min, λ = 210 nm: t_R (major) = 9.46 min, t_R (minor) = 13.78 min.); ^{1}H NMR (400 MHz, $CDCl_3$) δ 7.92–7.72 (m, 6H), 7.58–7.44 (m, 6H), 6.59 (s, 1H), 6.37 (s, 1H), 4.53–4.32 (m, 2H), 3.76 (dq, J = 10.5, 7.1 Hz, 1H), 3.15 (dq, J = 10.5, 7.2 Hz, 1H), 2.52 (s, 3H), 1.35 (t, J = 7.2 Hz, 3H), 0.34 (t, J = 7.2 Hz, 3H); ^{13}C NMR (101 MHz, $CDCl_3$) δ 168.3, 167.1, 134.8, 133.5, 131.5, 131.4, 130.8, 129.9, 129.6, 128.9, 128.7, 126.7, 126.0, 125.2, 124.1, 122.8, 92.3, 84.9, 77.4, 63.4, 61.9, 43.2, 13.9, 12.7. HRMS-ESI (*m/z*): calcd for $C_{26}H_{27}NNaO_7S^+$ [M + Na]$^+$: 520.1401; found 520.1407.

Diethyl (2*R*,5*S*)-3-(methylsulfonyl)-2-phenyl-5-(thiophen-2-yl)oxazolidine-4,4-dicarboxylate (3bn)

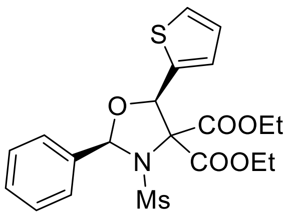

Colorless oil, 82% yield, R_f = 0.29 (PE/EtOAc = 10:1, *v/v*). $[\alpha]_D^{20}$ = +12.2 (*c* = 6.41 in CH_2Cl_2). 96% ee (Chiralpak AD-H, hexane/^{i}PrOH = 70/30, flow rate = 0.8 mL/min, λ = 210 nm: t_R (major) = 9.14 min, t_R (minor) = 20.47 min.); ^{1}H NMR (400 MHz, $CDCl_3$) δ 7.79–7.72 (m, 2H), 7.50–7.44 (m, 3H), 7.40–7.27 (m, 2H), 7.06 (dd, J = 5.0, 1.3 Hz, 1H), 6.21 (s, 1H), 5.86 (s, 1H), 4.49–4.29 (m, 2H), 4.04 (dq, J = 10.6, 7.1 Hz, 1H), 3.73 (dq, J = 10.6, 7.2 Hz, 1H), 2.48 (s, 3H), 1.38 (t, J = 7.2 Hz, 3H), 0.96 (t, J = 7.2 Hz, 3H); ^{13}C NMR (101 MHz, $CDCl_3$) δ 167.1, 167.0, 135.5, 134.7, 130.7, 129.7, 128.6, 126.00, 125.97, 123.1, 92.4, 84.7, 76.6, 63.3, 62.4, 43.1, 14.0, 13.5; HRMS-ESI (*m/z*): calcd for $C_{20}H_{23}NNaO_7S_2^+$ [M + Na]$^+$: 476.0809; found 476.0812.

Diethyl (2*R*,5*S*)-5-((*E*)-4-bromostyryl)-3-(methylsulfonyl)-2-phenyloxazolidine-4,4-dicarboxylate (3bo)

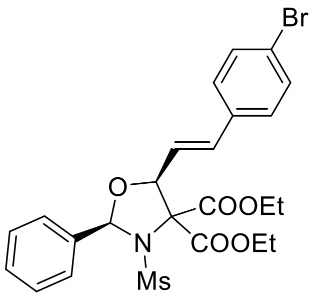

Colorless oil, 91% yield, R_f = 0.28 (PE/EtOAc = 10:1, *v/v*). $[\alpha]_D^{20}$ = +11.8 (*c* = 8.50 in CH_2Cl_2). 50% ee (Chiralpak AD-H, hexane/^{i}PrOH = 70/30, flow rate = 0.8 mL/min, λ = 210 nm: t_R (major) = 14.77 min, t_R (minor) = 20.30 min.); ^{1}H NMR (400 MHz, $CDCl_3$) δ 77.70–7.61 (m, 2H), 7.50–7.43 (m, 5H), 7.27 (d, J = 8.0 Hz, 2H), 6.67 (d, J = 15.9 Hz, 1H), 6.32 (dd, J = 15.9, 6.7 Hz, 1H), 6.16 (s, 1H), 5.31 (d, J = 6.6 Hz, 1H), 4.42–4.26 (m, 4H), 2.48 (s, 3H), 1.36 (t, J = 7.2 Hz, 3H), 1.26 (t, J = 7.2 Hz, 3H); ^{13}C NMR (101 MHz, $CDCl_3$) δ 167.2, 166.8, 134.8, 134.7, 132.4, 132.0, 130.7, 129.5, 128.7, 128.4, 122.5, 92.6, 86.6, 76.1, 63.4, 62.7, 43.0, 27.0, 14.3, 14.0; HRMS-ESI (*m/z*): calcd for $C_{24}H_{26}BrNNaO_7S^+$ [M + Na]$^+$: 574.0506; found 574.0511.

Diethyl (2*R*,5*S*)-2,5-diphenyl-3-(phenylsulfonyl)oxazolidine-4,4-dicarboxylate (3ca)[5]

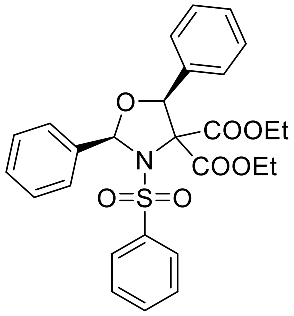

Colorless oil, 87% yield, R_f = 0.31 (PE/EtOAc = 10:1, *v/v*). $[\alpha]_D^{20}$ = +45.8 (*c* = 7.71 in CH_2Cl_2), Lit.[5] $[\alpha]_D^{14}$ = +49.5 (c = 0.59 in CH_2Cl_2). 88% ee (Chiralpak AD-H, hexane/^{i}PrOH = 70/30, flow rate = 0.8 mL/min, λ = 210 nm: t_R (major) = 9.34 min, t_R (minor) = 10.42 min.); ^{1}H NMR (400 MHz, $CDCl_3$) *δ* 7.48 (d, *J* = 6.9 Hz, 2H), 7.36–7.23 (m, 9H), 7.18–7.06 (m, 4H), 6.24 (s, 1H), 5.83 (s, 1H), 4.62–4.38 (m, 2H), 3.92 (dq, *J* = 10.6, 7.1 Hz, 1H), 3.50 (dq, *J* = 10.6, 7.2 Hz, 1H), 1.46 (t, *J* = 7.1 Hz, 3H), 0.80 (t, *J* = 7.2 Hz, 3H); ^{13}C NMR (101 MHz, $CDCl_3$) *δ* 167.4, 166.3, 140.6, 134.6, 133.9, 132.2, 130.1, 129.9, 129.1, 128.3, 128.2, 128.1, 127.8, 126.6, 93.01, 87.5, 77.0, 63.2, 62.1, 14.1, 13.4.

Diethyl (2*R*,5*S*)-3-((4-chlorophenyl)sulfonyl)-2,5-diphenyloxazolidine-4,4-dicarboxylate (3da)[5]

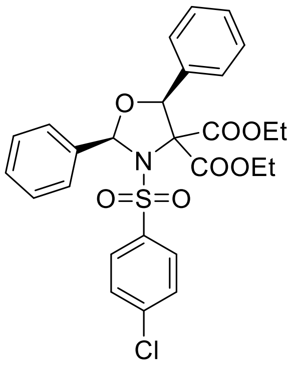

Colorless crystals, m.p. 136–137 °C, 82% yield, R_f = 0.28 (PE/EtOAc = 10:1, *v/v*). $[\alpha]_D^{20}$ = +18.6 (*c* = 7.56 in CH_2Cl_2), Lit.[5] $[\alpha]_D^{14}$ = +59.6 (c = 0.70 in CH_2Cl_2). 20%ee (Chiralpak AD-H, hexane/^{i}PrOH = 70/30, flow rate = 0.8 mL/min, λ = 210 nm: t_R (major) = 8.51 min, t_R (minor) = 9.71 min.); ^{1}H NMR (400 MHz, $CDCl_3$) *δ* 7.50–7.42 (m, 2H), 7.33 (s, 5H), 7.32–7.14 (m, 5H), 7.06 (d, *J* = 8.8 Hz, 2H), 6.22 (s, 1H), 5.82 (s, 1H), 4.59–4.39 (m, 2H), 3.94 (dq, *J* = 10.6, 7.1 Hz, 1H), 3.50 (dq, *J* = 10.6, 7.1 Hz, 1H), 1.46 (t, *J* = 7.1 Hz, 3H), 0.80 (t, *J* = 7.1 Hz, 3H); ^{13}C NMR (101 MHz, $CDCl_3$) *δ* 167.3, 166.4, 139.1, 138.7, 134.6, 133.7, 130.3, 130.0, 129.7, 129.2, 128.4, 128.2, 128.0, 126.7, 92.9, 87.6, 77.2, 63.3, 62.2, 14.1, 13.4.

Dimethyl (2*R*,5*S*)-5-(4-bromophenyl)-3-(methylsulfonyl)-2-phenyloxazolidine-4,4-dicarboxylate (3ef)

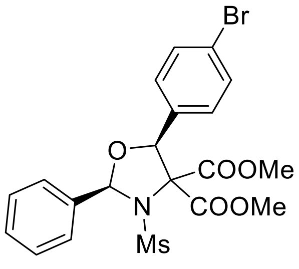

Colorless oil, 59% yield, R_f = 0.28 (PE/EtOAc = 10:1, *v/v*). $[\alpha]_D^{20}$ = +29.2 (*c* = 7.04 in CH_2Cl_2). > 99% ee (Chiralpak AD-H, hexane/^{i}PrOH = 70/30, flow rate = 0.8 mL/min, λ = 210 nm: t_R (major) = 9.77 min, t_R (minor) = 37.61 min.); ^{1}H NMR (400 MHz, $CDCl_3$) *δ* 7.82–7.69 (m, 2H), 7.60–7.42 (m, 5H), 7.28–7.18 (m, 2H), 6.23 (s, 1H), 5.76 (s, 1H), 3.93 (s, 3H), 3.36 (s, 3H), 2.48 (s, 3H); ^{13}C NMR (101 MHz, $CDCl_3$) *δ* 167.5, 167.3, 134.4, 133.4, 131.7, 130.9, 129.7, 128.7, 128.1, 123.3, 92.6, 87.1, 76.8, 54.2, 52.9, 43.1. HRMS-ESI (*m/z*): calcd for $C_{20}H_{20}BrNNaO_7S^+$ $[M + Na]^+$: 520.0037; found 520.0042.

Diisopropyl (2*R*,5*S*)-5-(4-bromophenyl)-3-(methylsulfonyl)-2-phenyloxazolidine-4,4-dicarboxylate (3ff)

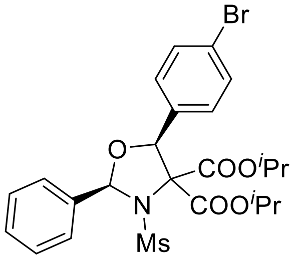

Colorless oil, 99% yield, R_f = 0.28 (PE/EtOAc = 10:1, *v/v*). $[\alpha]_D^{20}$ = +20.5 (*c* = 9.48 in CH_2Cl_2). 98% ee (Chiralpak AD-H, hexane/^{i}PrOH = 70/30, flow rate = 0.8 mL/min, λ = 210 nm: t_R (major) = 8.46 min, t_R (minor) = 22.05 min.); ^{1}H NMR (400 MHz, $CDCl_3$) δ 7.81–7.66 (m, 2H), 7.53–7.45 (m, 5H), 7.29–7.17 (m, 2H), 6.20 (s, 1H), 5.77 (s, 1H), 5.24 (hept, *J* = 6.3 Hz, 1H), 4.77 (hept, *J* = 6.2 Hz, 1H), 2.48 (s, 3H), 1.37 (d, *J* = 6.4 Hz, 6H), 1.08 (d, *J* = 6.2 Hz, 3H), 0.73 (d, *J* = 6.3 Hz, 3H); ^{13}C NMR (101 MHz, $CDCl_3$) δ 166.6, 166.4, 134.7, 133.9, 131.5, 130.7, 129.6, 128.7, 128.2, 122.9, 92.3, 86.9, 76.7, 71.5, 70.7, 43.1, 21.8, 21.6, 21.4, 20.8. HRMS-ESI (*m/z*): calcd for $C_{24}H_{28}BrNNaO_7S^+$ [M + Na]$^+$: 576.0663; found 576.0668.

Diethyl (2*R*,5*S*)-2-(4-bromophenyl)-5-phenyl-3-tosyloxazolidine-4,4-dicarboxylate (3ga)

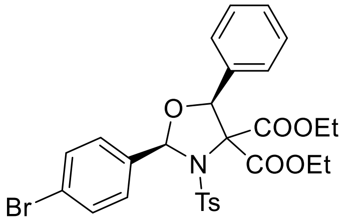

Colorless crystals, m.p. 171–172 °C, 70% yield, R_f = 0.28 (PE/EtOAc = 10:1, *v/v*). $[\alpha]_D^{20}$ = +11.7 (*c* = 6.36 in CH_2Cl_2). 38% ee (Chiralpak AD-H, hexane/^{i}PrOH = 70/30, flow rate = 0.8 mL/min, λ = 210 nm: t_R (major) = 10.70 min, t_R (minor) = 23.72 min.); ^{1}H NMR (400 MHz, $CDCl_3$) δ 7.50–7.27 (m, 7H), 7.26–7.17 (m, 4H), 6.97 (d, *J* = 8.1 Hz, 2H), 6.16 (s, 1H), 5.81 (s, 1H), 4.59–4.37 (m, 2H), 3.93 (dq, *J* = 10.6, 7.1 Hz, 1H), 3.49 (dq, *J* = 10.6, 7.2 Hz, 1H), 2.35 (s, 3H), 1.45 (t, *J* = 7.1 Hz, 3H), 0.80 (t, *J* = 7.1 Hz, 3H); ^{13}C NMR (101 MHz, $CDCl_3$) δ 167.2, 166.4, 143.4, 137.6, 134.4, 133.2, 131.5, 131.2, 129.2, 128.6, 128.4, 128.3, 126.6, 124.5, 92.1, 87.6, 77.0, 63.2, 62.2, 21.6, 14.1, 13.4, HRMS-ESI (*m/z*): calcd for $C_{28}H_{28}BrNNaO_7S^+$ [M + Na]$^+$: 624.0663; found 624.0667.

4. Experimental procedure for the scale-up reaction

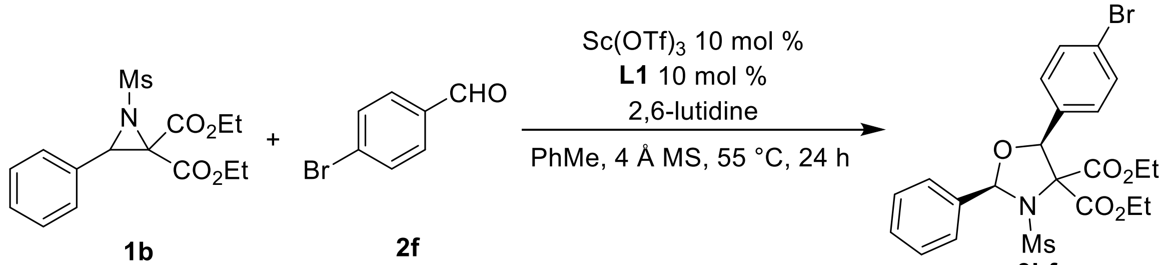

1b
1.024 g
3 mmol

2f
0.647 g
3.5 mmol

3bf
1.372 g
87% yield
90% ee

$Sc(OTf)_3$ (147 mg, 0.3 mmol, 10 mol%) in an oven-dried 20 mL Schlenk tube was heated at 220 °C for 2 h under oil pump vacuum. After gradually cooling to room temperature, **L1** (164 mg, 0.3 mmol, 10 mol%), 2,6-lutidine (70 µL, 0.6 mmol, 20 mol%), 4 Å MS (1 g), and 10 mL of dry toluene were added into the Schlenk tube. The resulting solution was stirred at 55 °C for 3 h under nitrogen atmosphere. A solution of aldehyde **2f** (0.647 g, 3.5 mmol) and aziridine **1b** (1.024 g, 3.0 mmol) in 5 mL of dry toluene was added dropwise into the mixture. The mixture was stirred at 55 °C for 24 h. After gradually cooling to room temperature and removal the solvent under reduced pressure, the crude residue was purified on basic aluminum oxide column chromatography with petroleum ether/ethyl acetate (1:10 to 3:7, *v*/*v*) as eluent to afford the desired product **3bf** (1.372 g, 87% yield, >20:1 dr, 90% ee).

5. References

1. Lee, K. Y.; Lee, C. G.; Kim, J. N. *Tetrahedron Lett.*, **2003**, *44*, 1231-1234.
2. Morales, S.; Guijarro, F. G.; García Ruano, J. L.; Cid, M. B. *J. Am. Chem. Soc.*, **2014**, *136*, 1082-1089.
3. Chapman, M. R.; Henkelis, S. E.; Kapur, N.; Nguyen, B. N.; Willans, C. E. *ChemistryOpen*, 2016, **5**, 351-356.
4. Wu, X.; Li, L.; Zhang, J. *Adv. Synth. Catal.*, **2012**, *354*, 3485-3489.
5. Liao, Y.; Liu, X.; Zhang, Y.; Xu, Y.; Xia, Y.; Lin, L.; Feng, X. *Chem. Sci.*, **2016**, *7*, 3775-3779.
6. Wu, X.; Zhou, W.; Wu, H.-H.; Zhang, J. *Chem. Commun.*, **2017**, *53*, 5661-5664.
7. Jiang, Z.; Wang, J.; Lu, P.; Wang, Y. *Tetrahedron*, **2011**, *67*, 9609-9617.
8. Wu, X.; Li, L.; Zhang, J. *Chem. Commun.*, **2011**, *47*, 7824-7826.

6. Copies of NMR and HRMS spectra, and HPLC profiles of products

^{1}H NMR of diethyl 3-phenyl-1-tosylaziridine-2,2-dicarboxylate (1a) (400 MHz, $CDCl_3$)

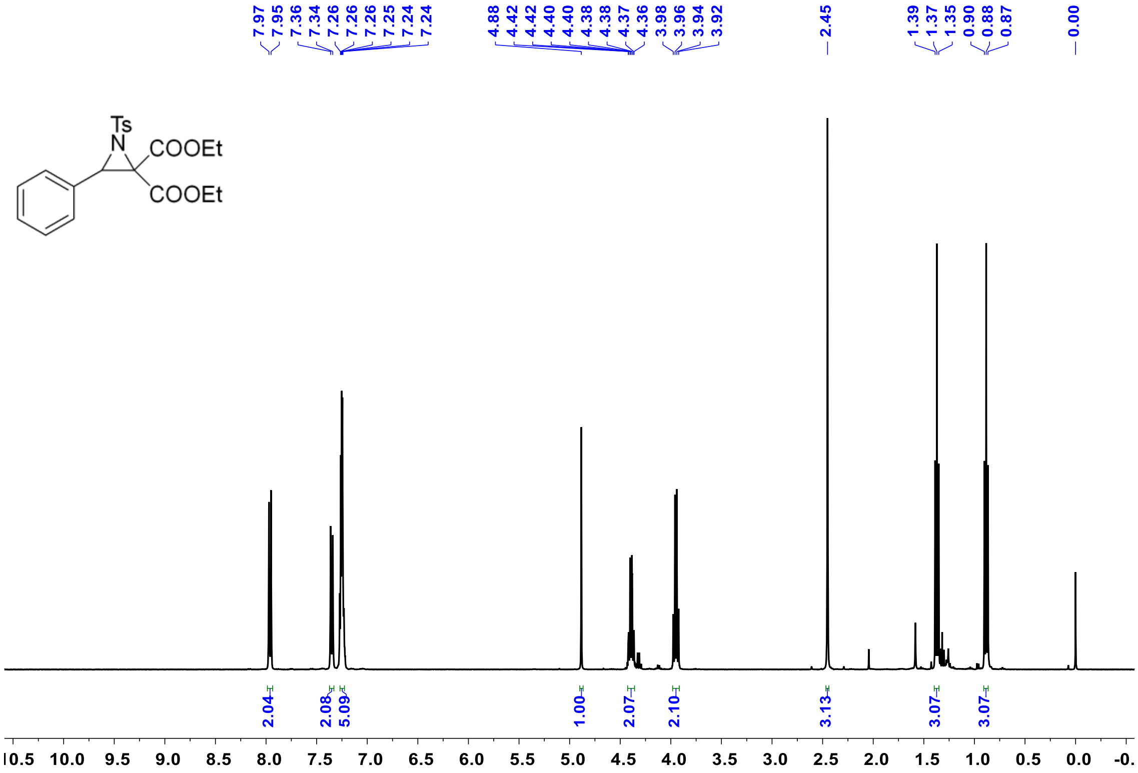

$^{13}C\{^{1}H\}$ NMR of diethyl 3-phenyl-1-tosylaziridine-2,2-dicarboxylate (1a) (101 MHz, $CDCl_3$)

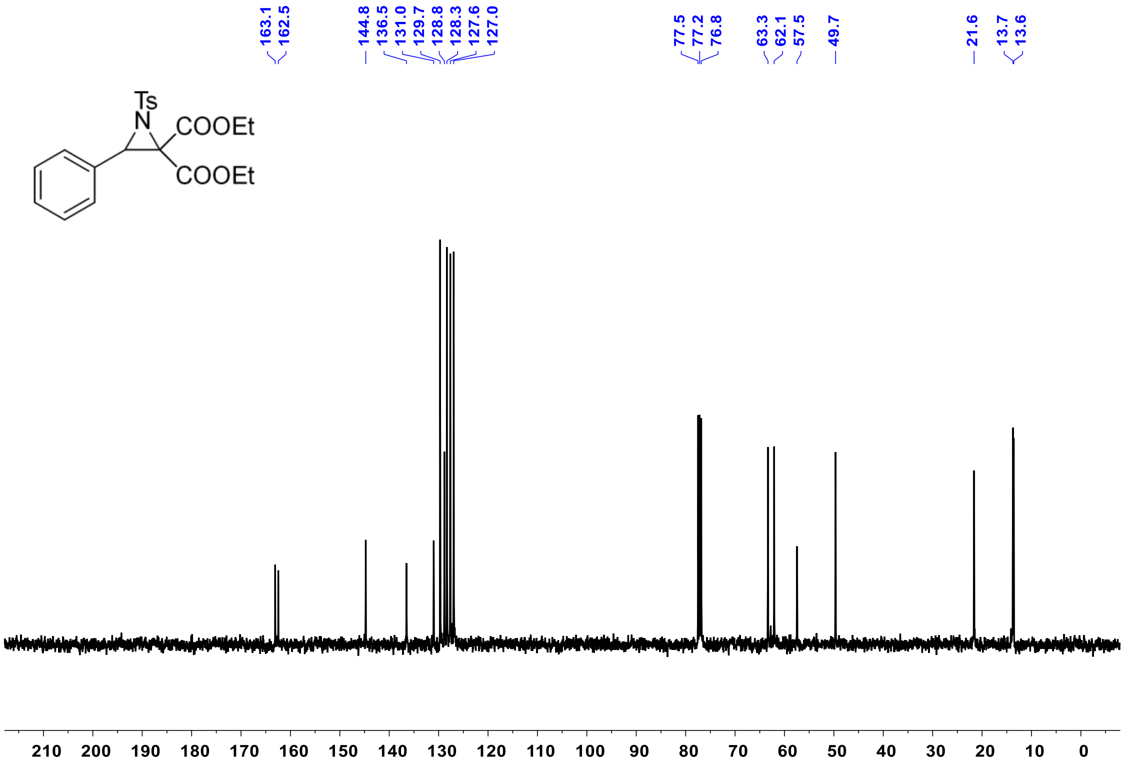

^{1}H NMR of diethyl 1-(methylsulfonyl)-3-phenylaziridine-2,2-dicarboxylate (1b) (400 MHz, $CDCl_3$)

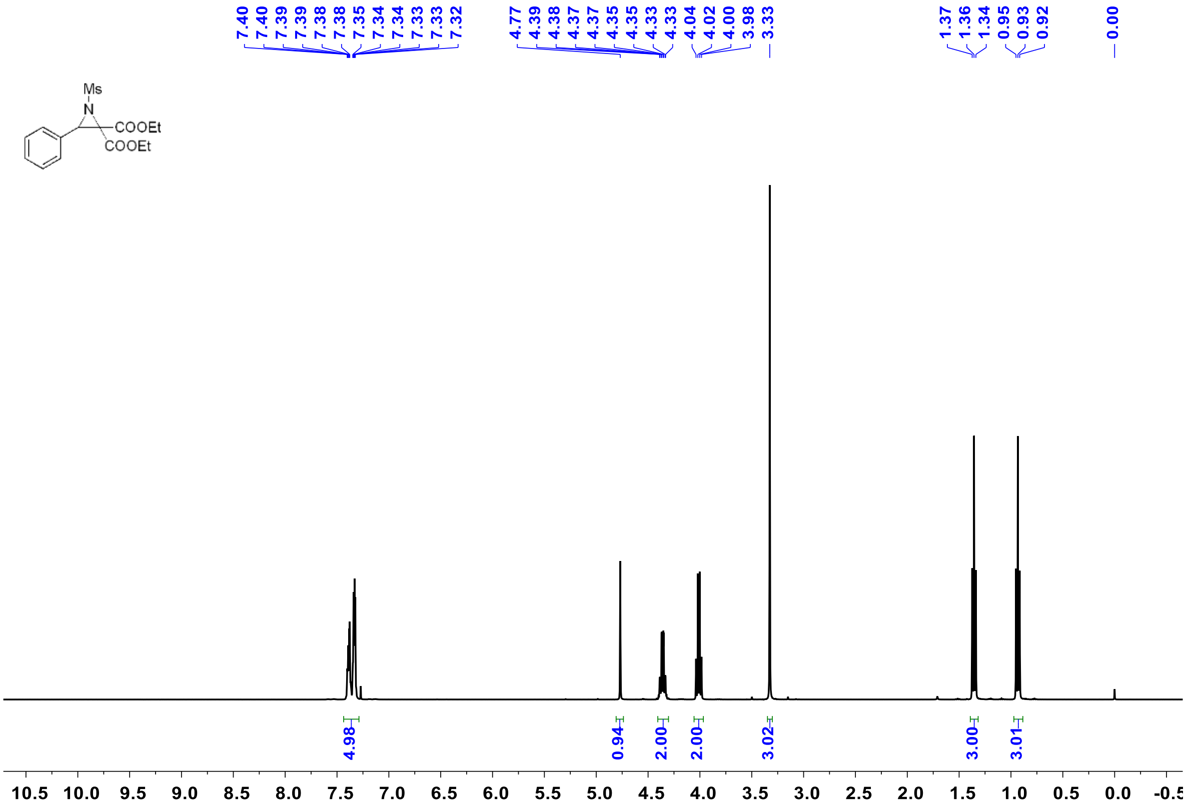

^{13}C{^{1}H} NMR of diethyl 1-(methylsulfonyl)-3-phenylaziridine-2,2-dicarboxylate (1b) (101 MHz, $CDCl_3$)

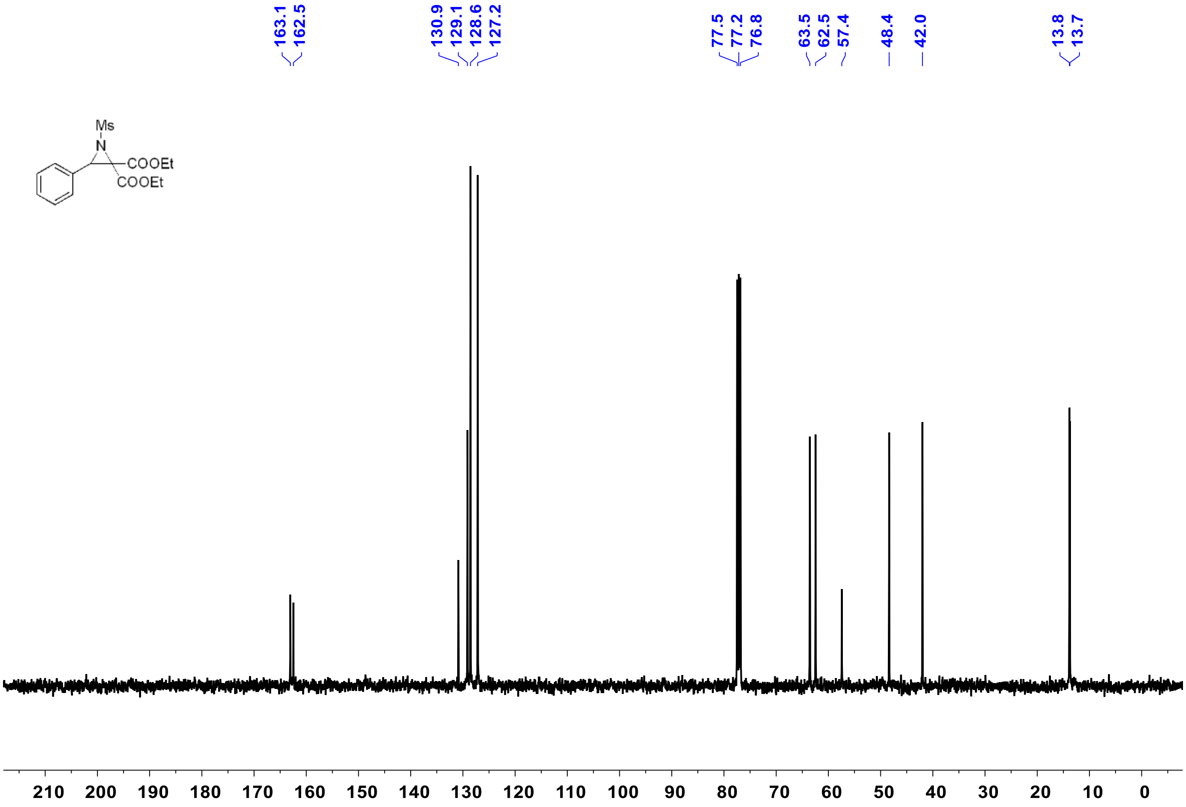

^{1}H NMR of diethyl 3-phenyl-1-(phenylsulfonyl)aziridine-2,2-dicarboxylate (1c) (400 MHz, $CDCl_3$)

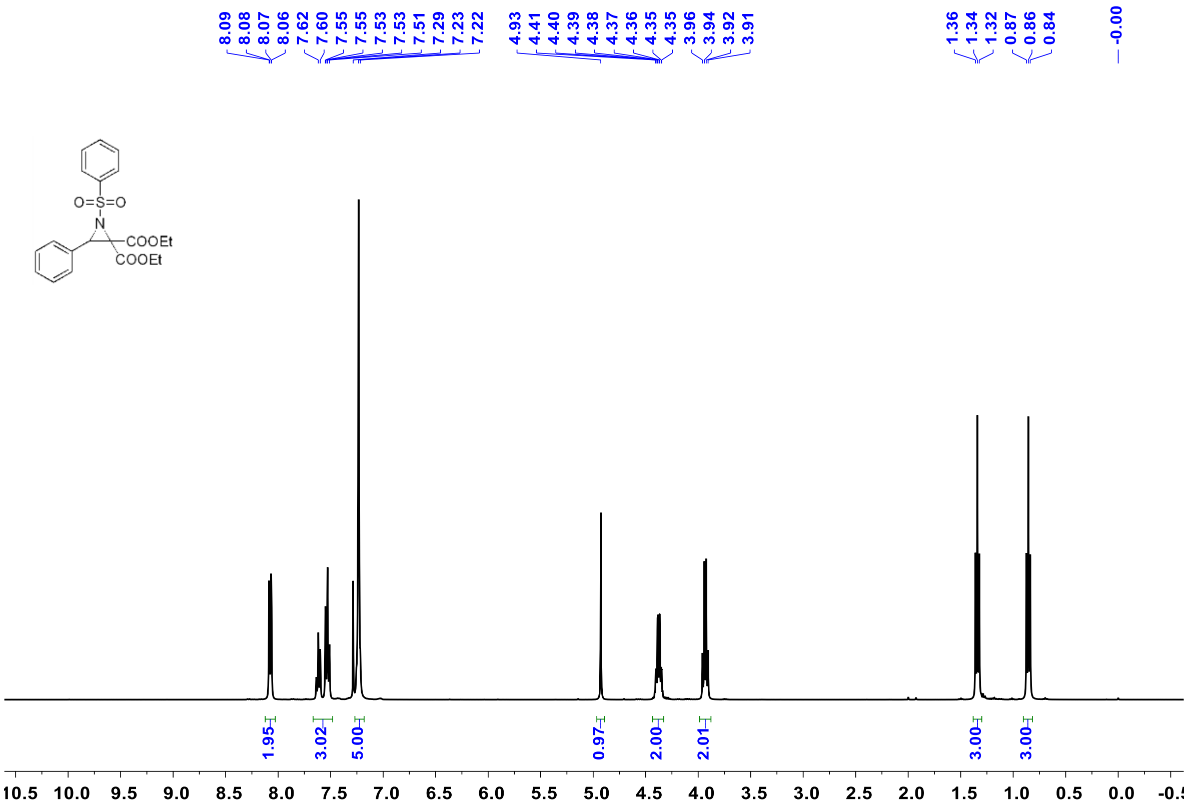

^{13}C{^{1}H} NMR of diethyl 3-phenyl-1-(phenylsulfonyl)aziridine-2,2-dicarboxylate (1c) (101 MHz, $CDCl_3$)

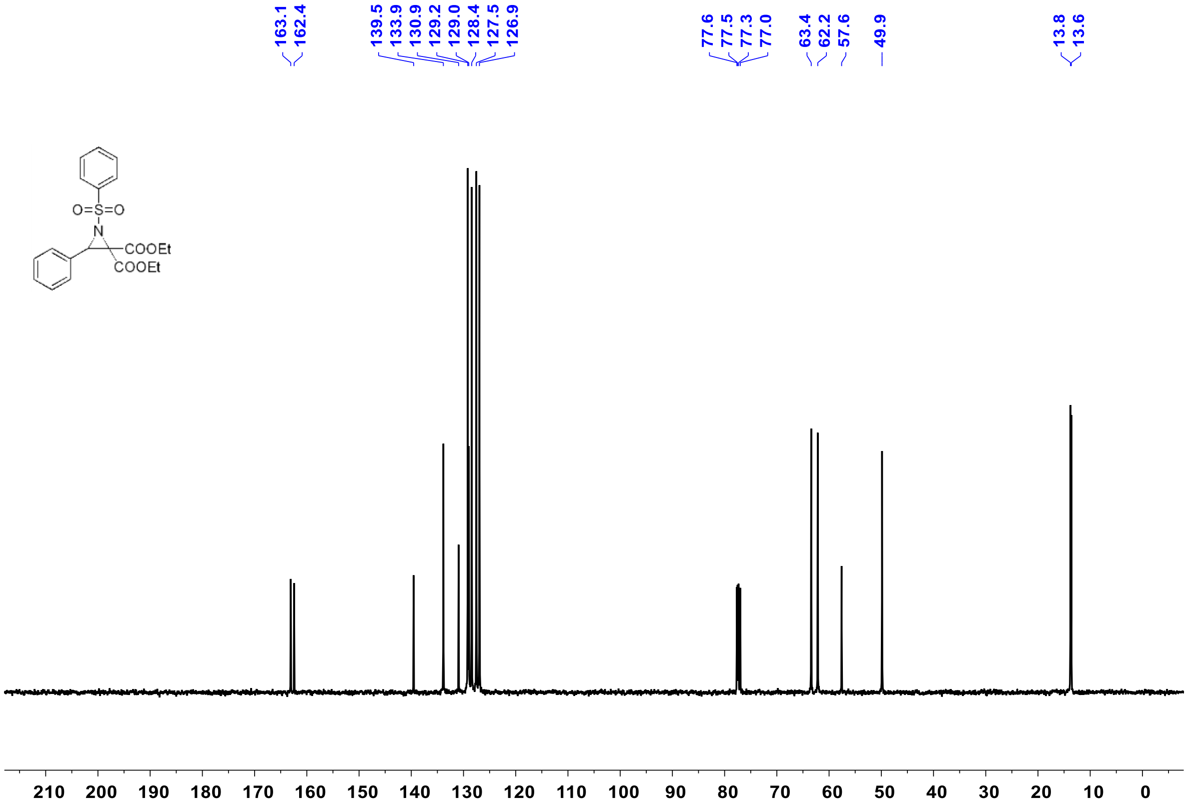

^{1}H NMR of diethyl 1-((4-chlorophenyl)sulfonyl)-3-phenylaziridine-2,2-dicarboxylate (1d) (400 MHz, CDCl3)

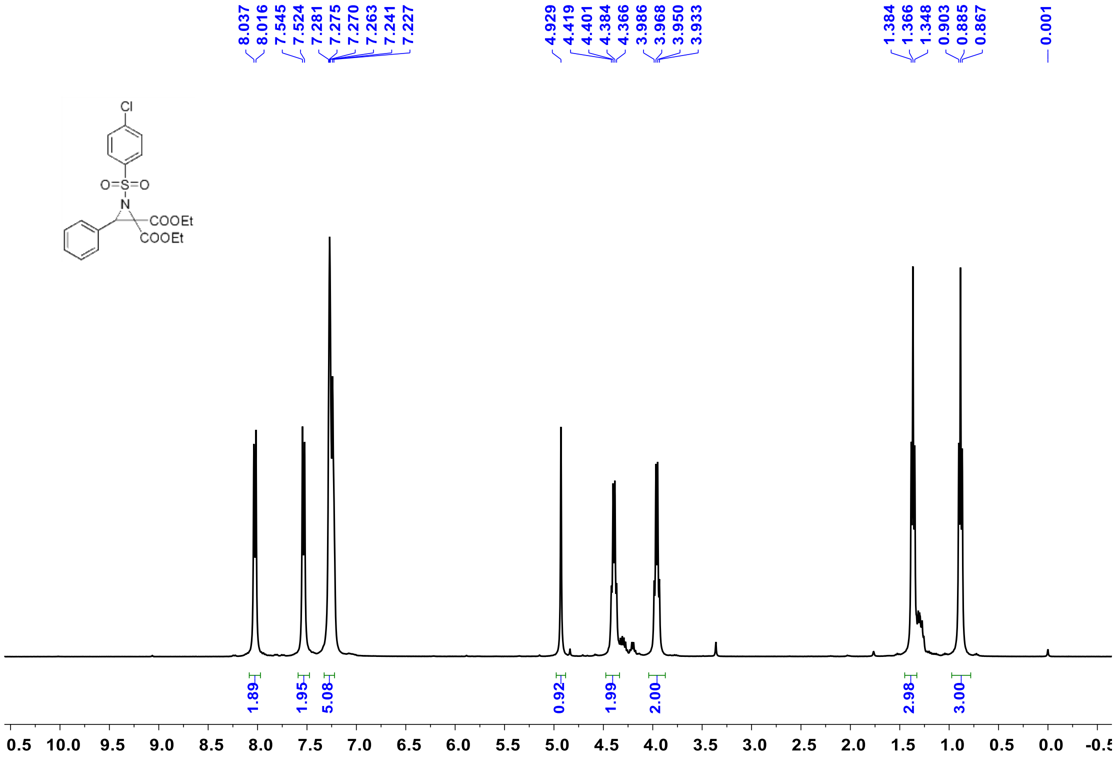

^{13}C{^{1}H} NMR of diethyl 1-((4-chlorophenyl)sulfonyl)-3-phenylaziridine-2,2-dicarboxylate (1d) (101 MHz, CDCl3)

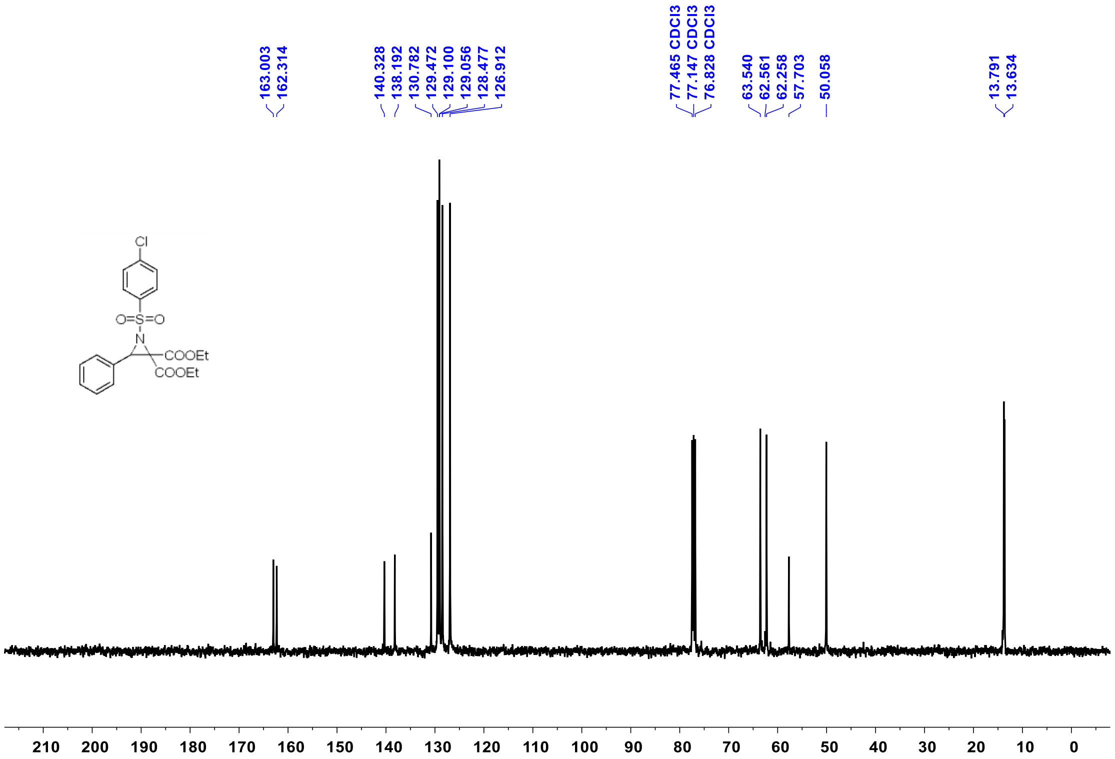

^{1}H NMR of dimethyl 1-(methylsulfonyl)-3-phenylaziridine-2,2-dicarboxylate (1e) (400 MHz, $CDCl_3$)

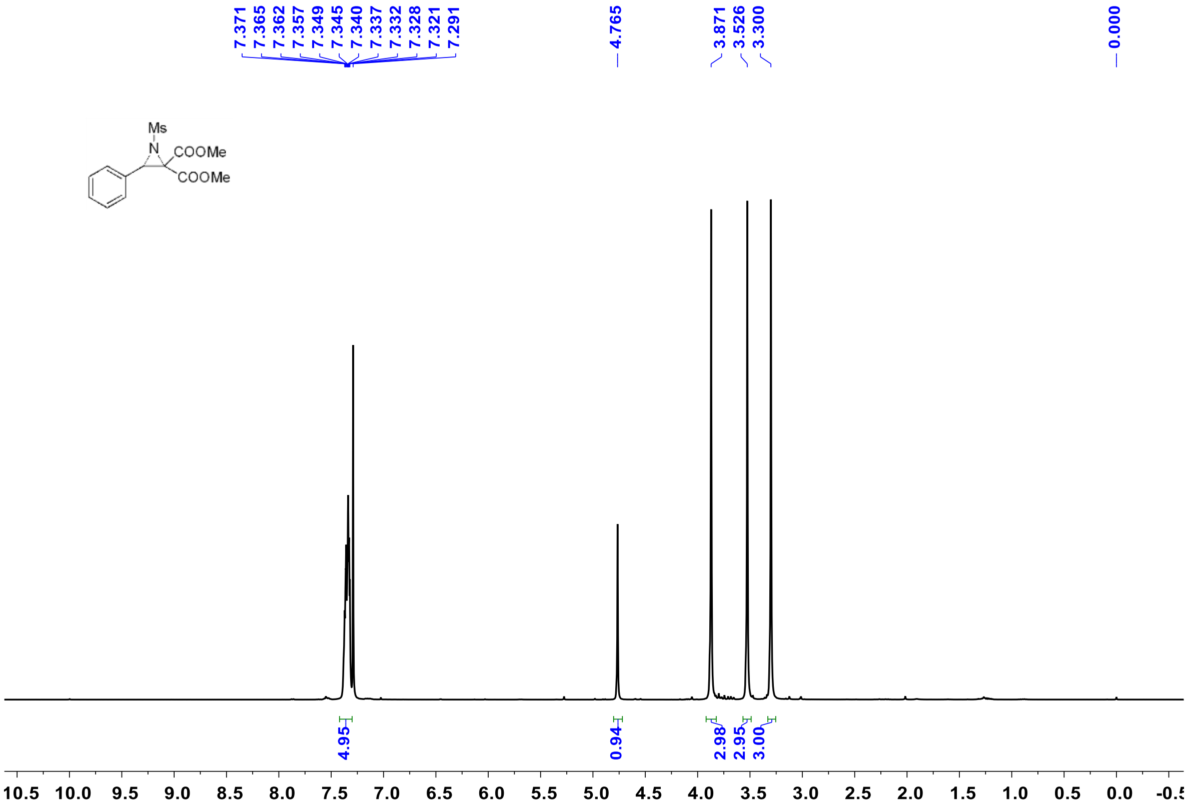

$^{13}C\{^{1}H\}$ NMR of dimethyl 1-(methylsulfonyl)-3-phenylaziridine-2,2-dicarboxylate (1e) (101 MHz, $CDCl_3$)

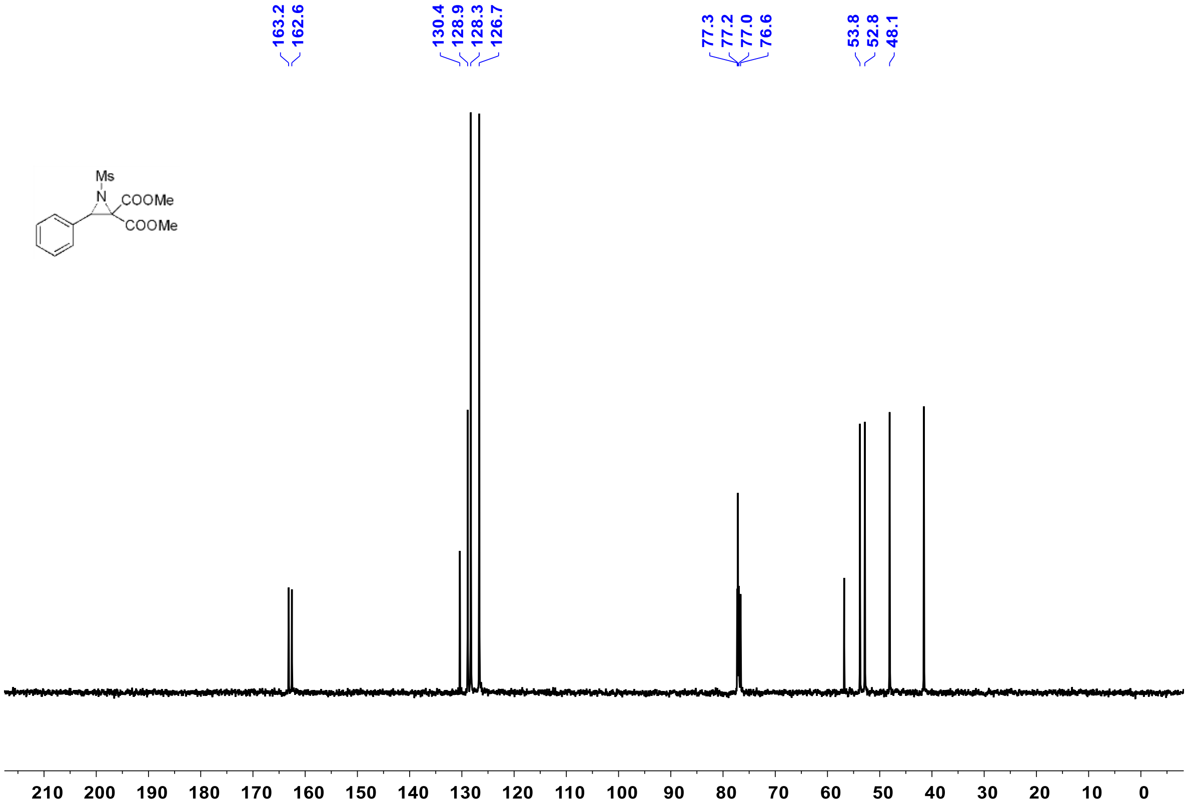

^{1}H NMR of diisopropyl 1-(methylsulfonyl)-3-phenylaziridine-2,2-dicarboxylate (1f) (400 MHz, $CDCl_3$)

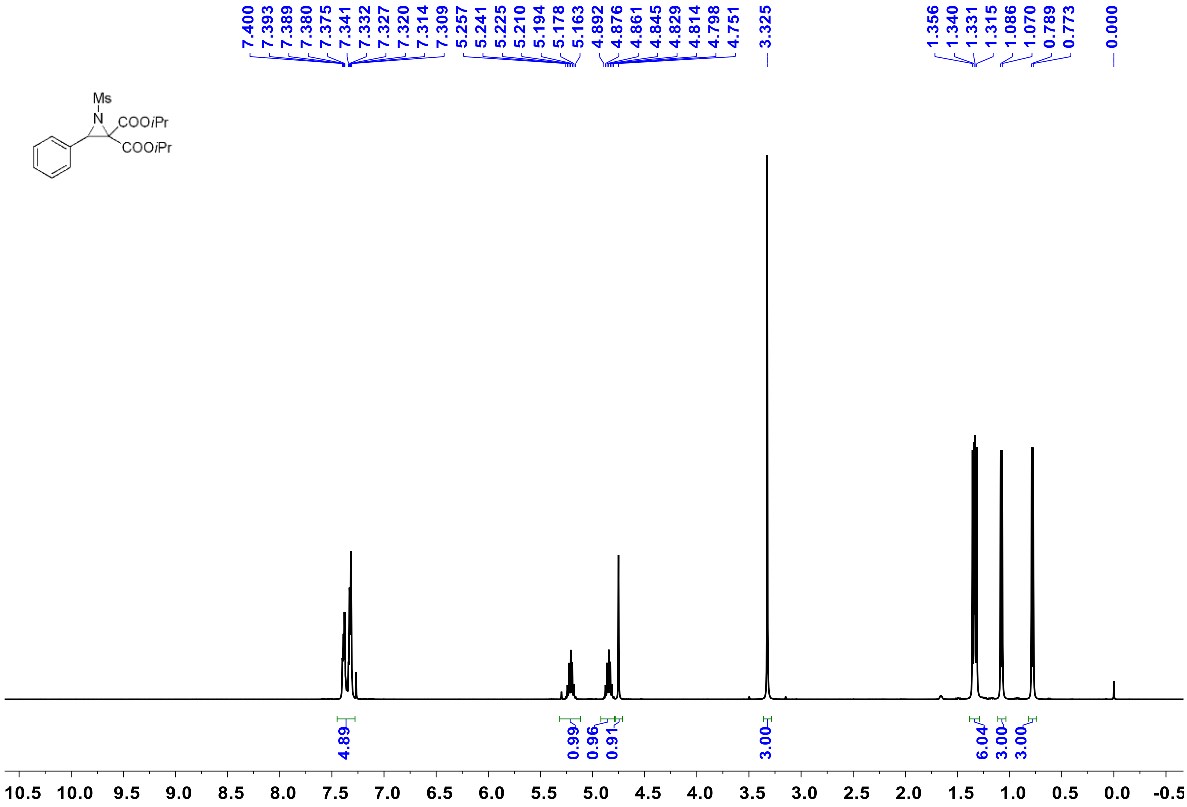

$^{13}C\{^{1}H\}$ NMR of diisopropyl 1-(methylsulfonyl)-3-phenylaziridine-2,2-dicarboxylate (1f) (101 MHz, $CDCl_3$)

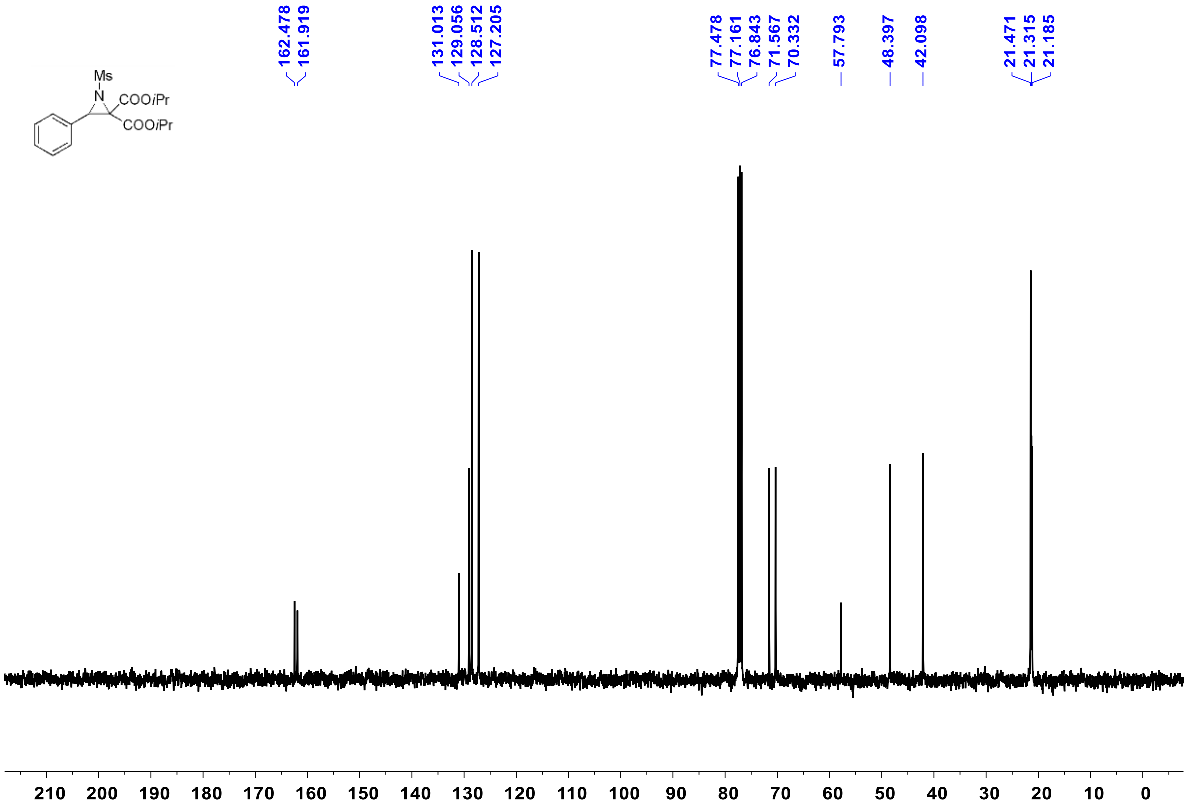

^{1}H NMR of diethyl 3-(4-bromophenyl)-1-tosylaziridine-2,2-dicarboxylate (1g) (400 MHz, $CDCl_3$)

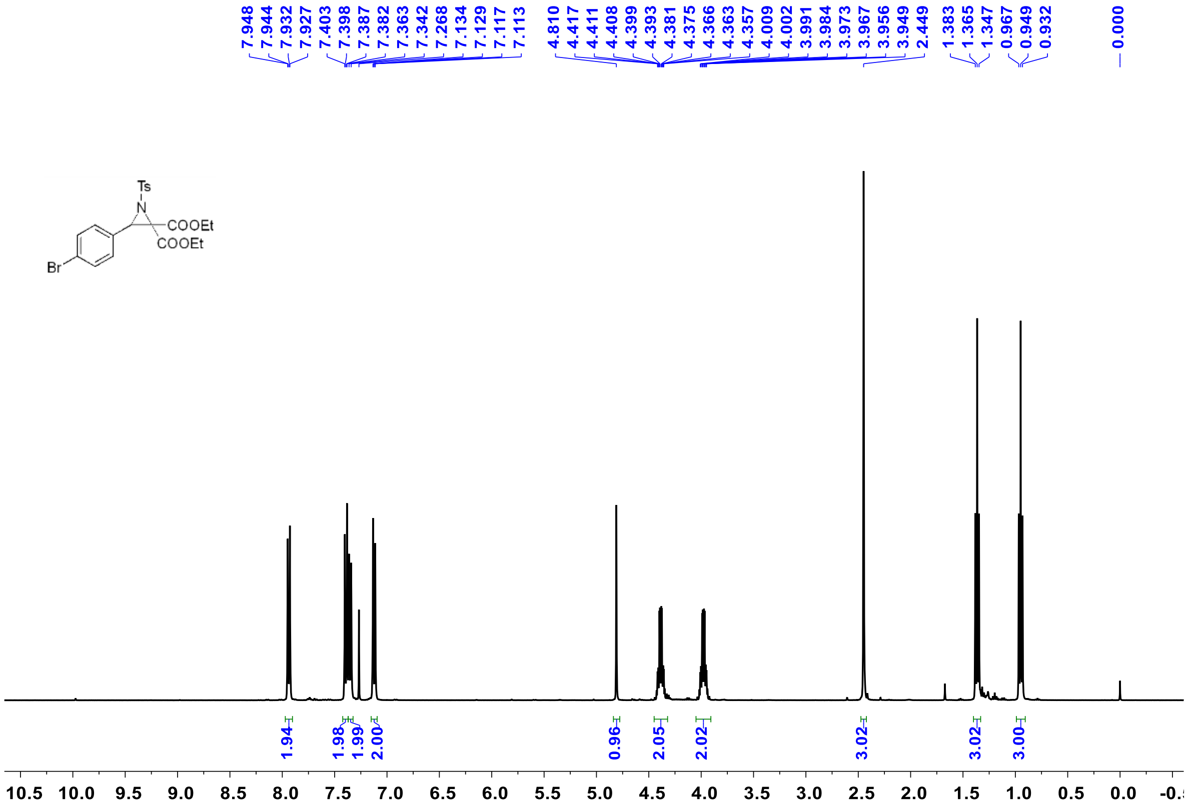

^{13}C{^{1}H} NMR of diethyl 3-(4-bromophenyl)-1-tosylaziridine-2,2-dicarboxylate (1g) (101 MHz, $CDCl_3$)

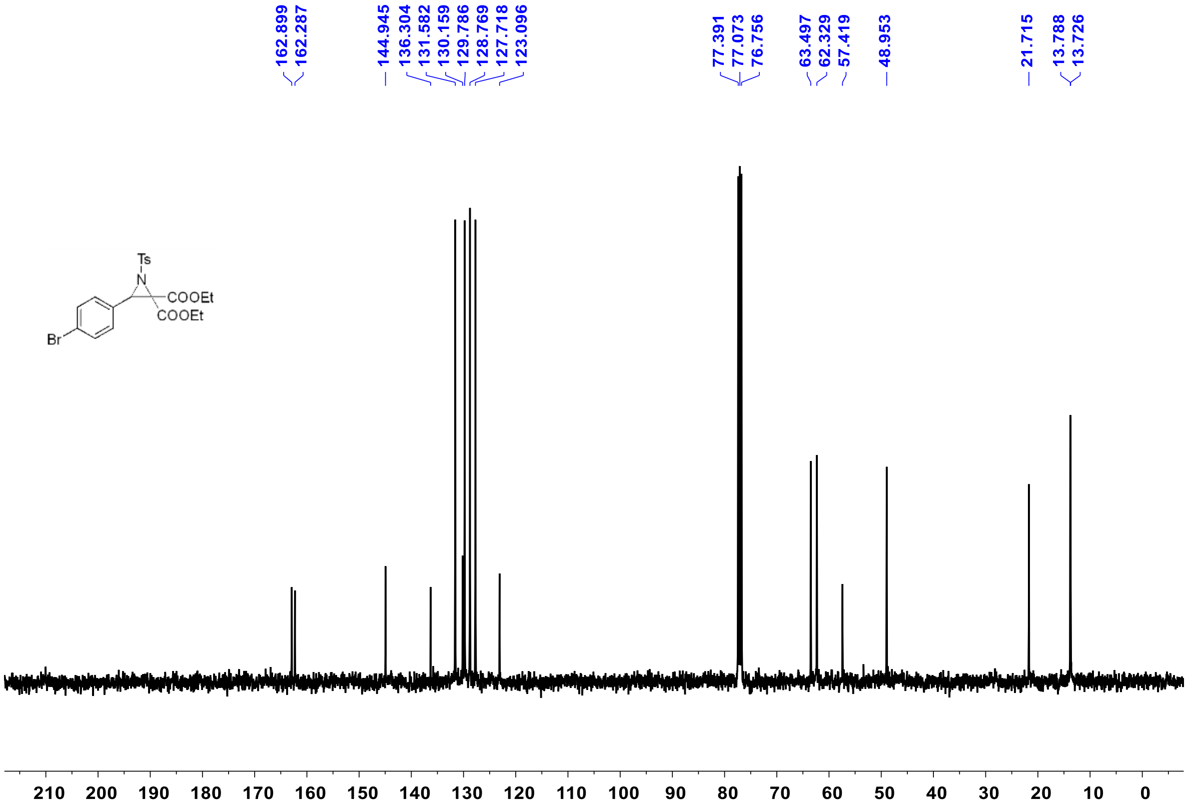

^{1}H NMR of diethyl (2*R*,5*S*)-2,5-diphenyl-3-tosyloxazolidine-4,4-dicarboxylate (3aa) (400 MHz, $CDCl_3$)

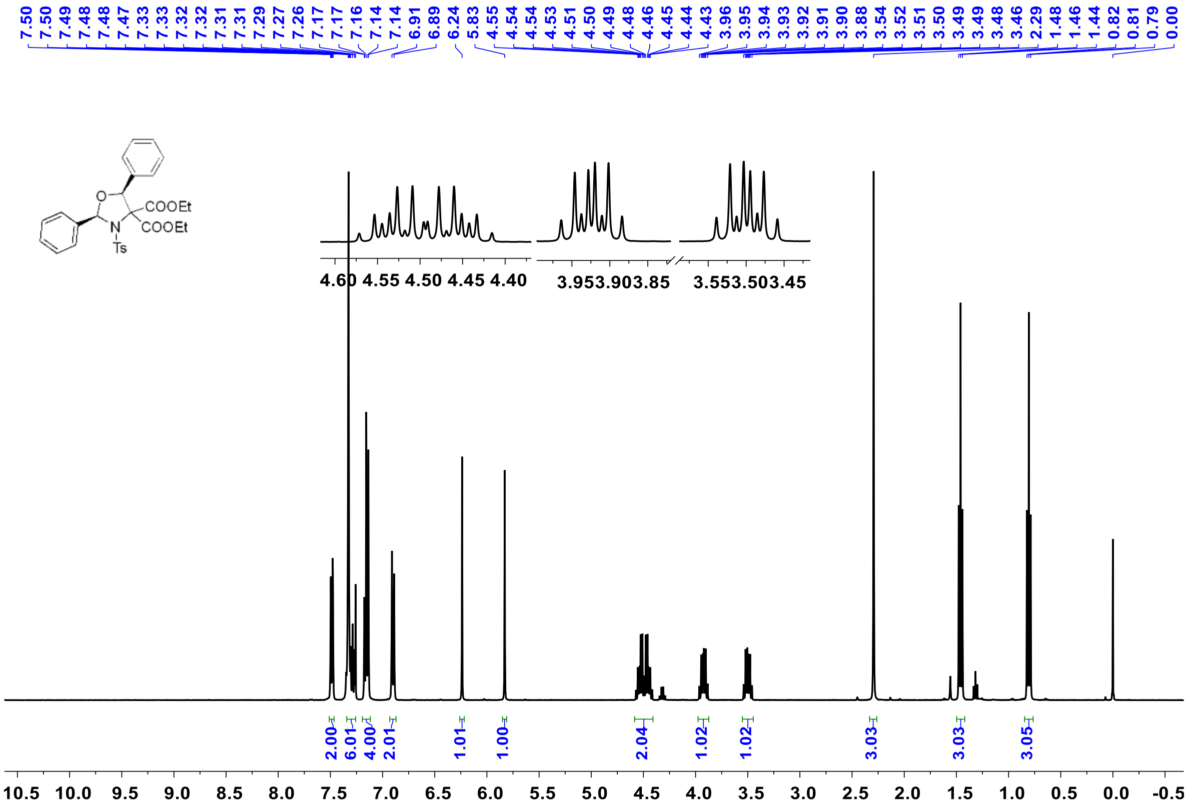

^{13}C{^{1}H} NMR of diethyl (2*R*,5*S*)-2,5-diphenyl-3-tosyloxazolidine-4,4-dicarboxylate (3aa) (101 MHz, $CDCl_3$)

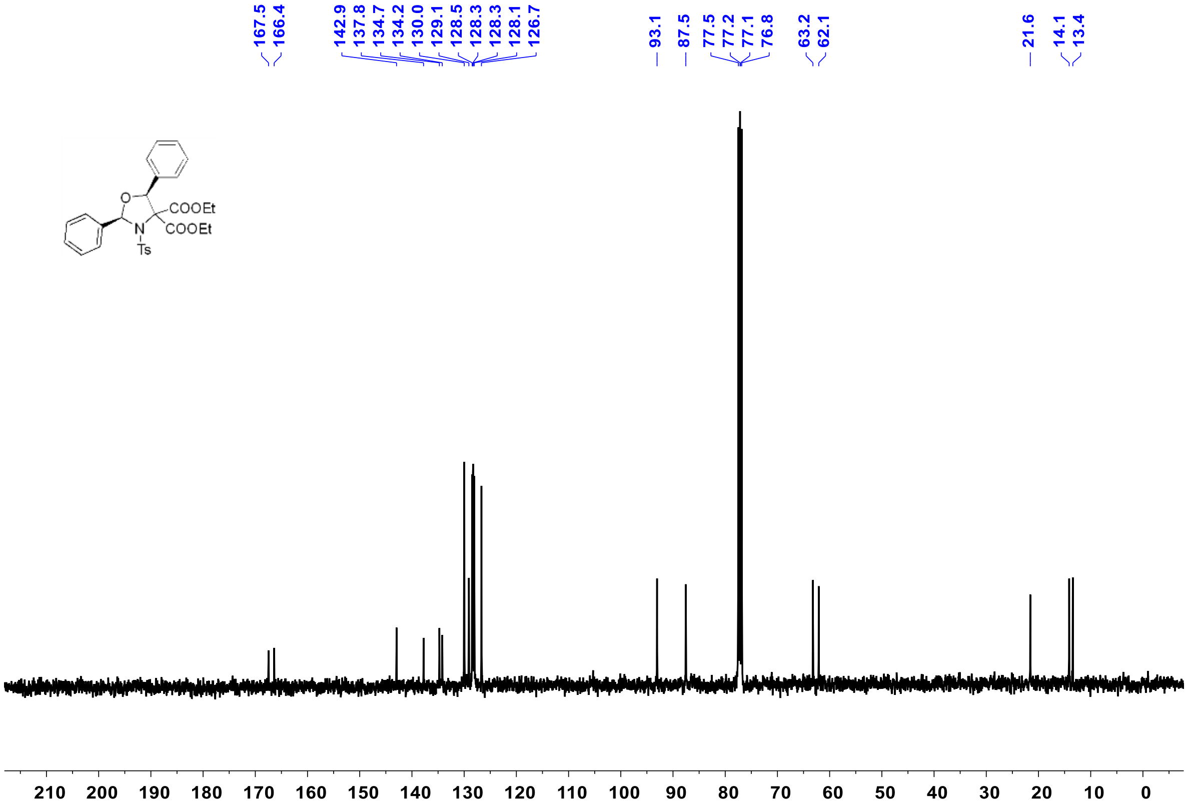

HPLC graph of racemic 3aa

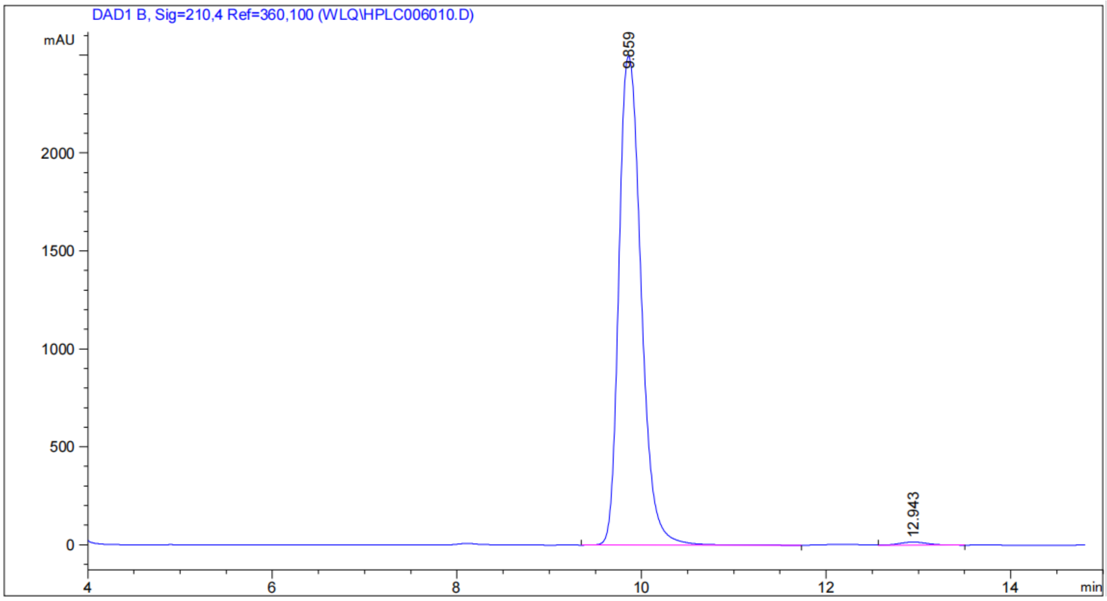

Peak #	Rt time [min]	Type	Width [min]	Peak Area [mAU*s]	Peak Height [mAU]	Peak Area %
1	9.859	BB	0.2605	4.16167e4	2496.35571	99.2229
2	12.943	VB	0.3183	325.92825	15.82628	0.7771

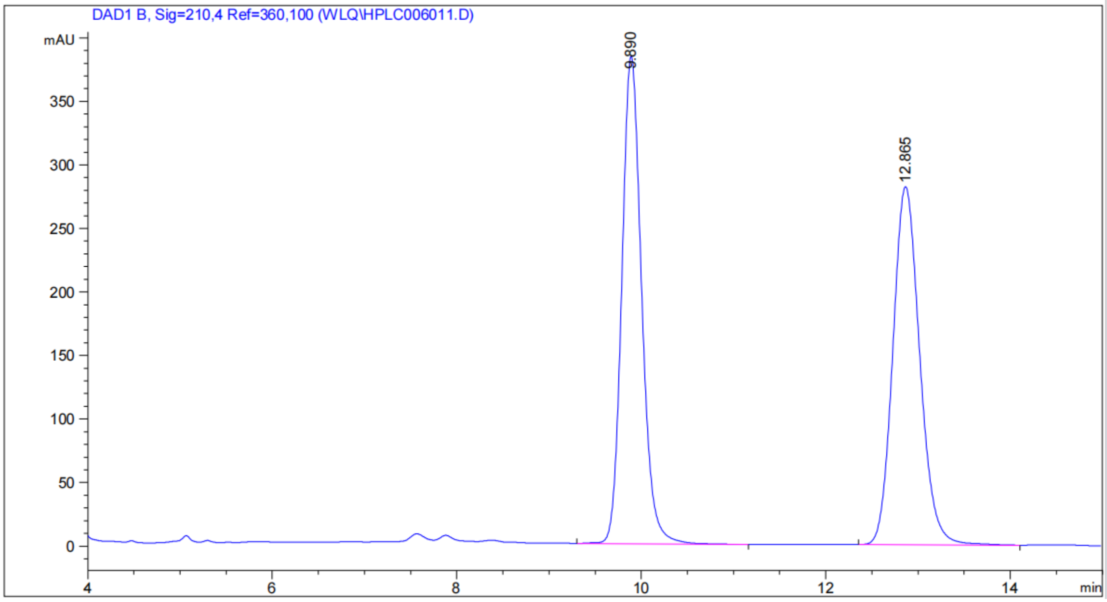

Peak #	Rt time [min]	Type	Width [min]	Peak Area [mAU*s]	Peak Height [mAU]	Peak Area %
1	9.890	BB	0.2293	5701.50439	383.93927	50.0755
2	12.865	BB	0.3113	5684.31494	281.84811	49.9245

^{1}H NMR of diethyl (2*R*,5*S*)-2-phenyl-5-(p-tolyl)-3-tosyloxazolidine-4,4-dicarboxylate (3ab) (400 MHz, $CDCl_3$)

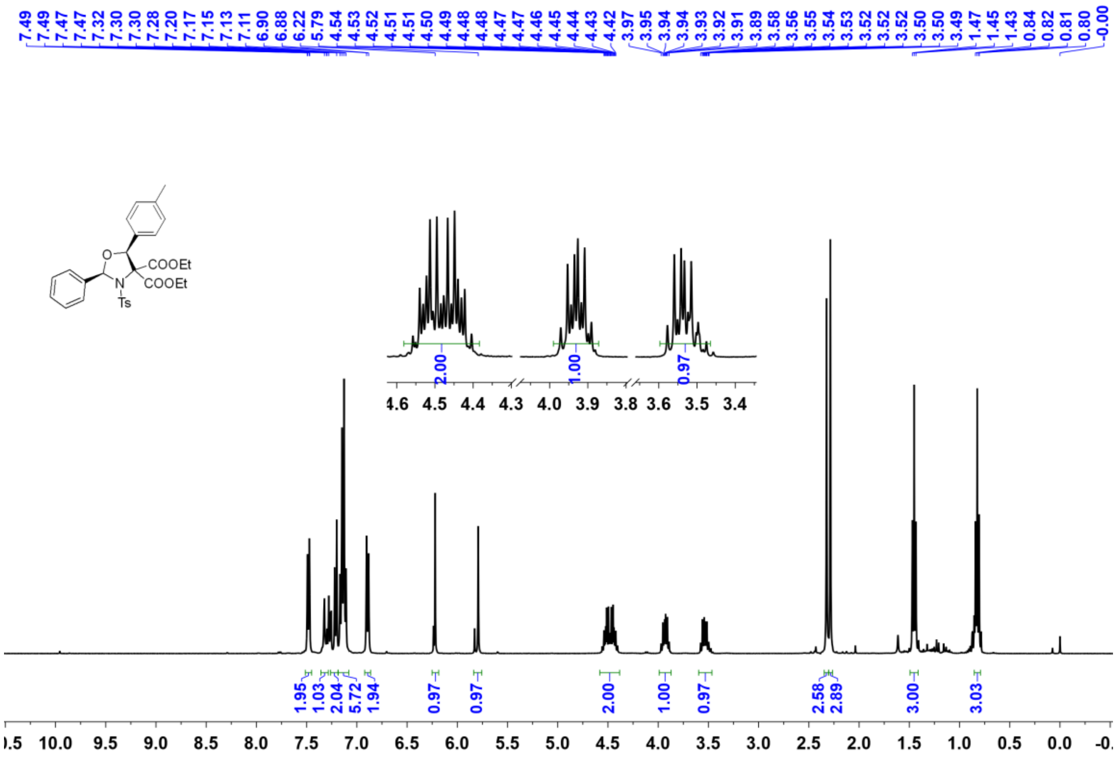

^{13}C{^{1}H} NMR of diethyl (2*R*,5*S*)-2-phenyl-5-(p-tolyl)-3-tosyloxazolidine-4,4-dicarboxylate (3ab) (101 MHz, $CDCl_3$)

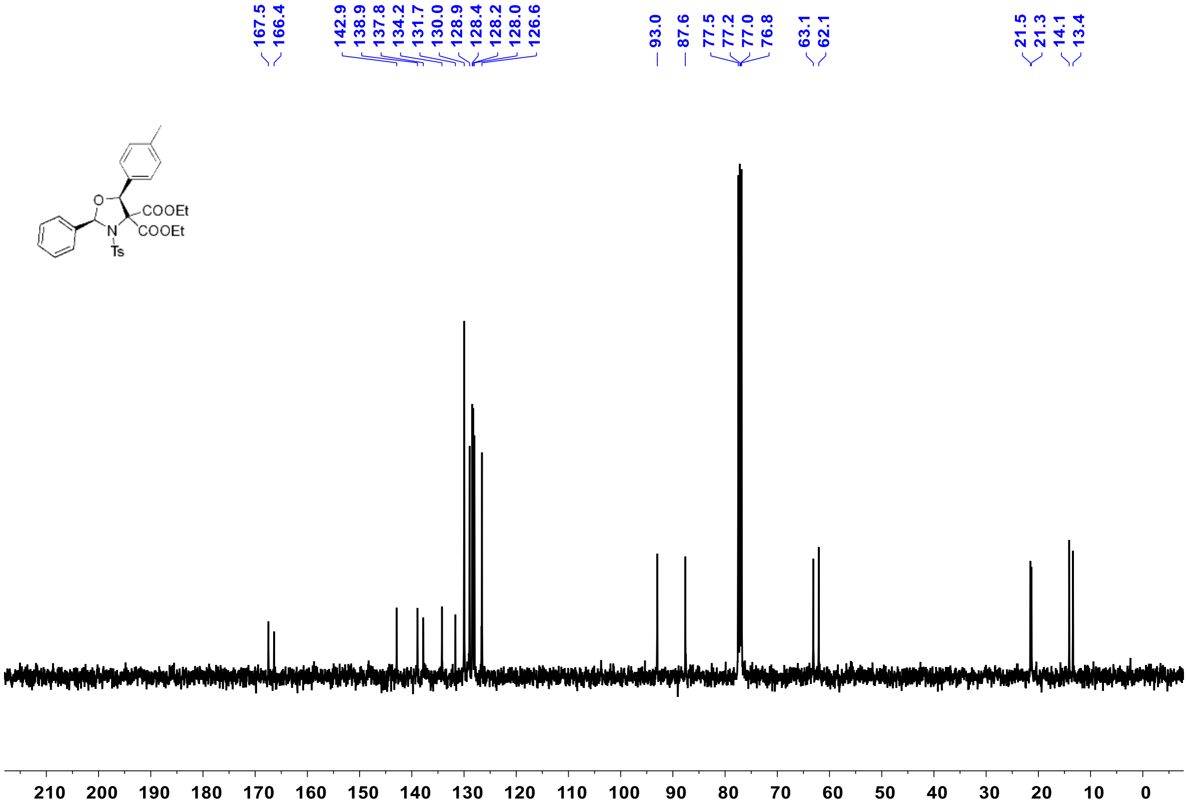

HPLC graph of racemic 3ab

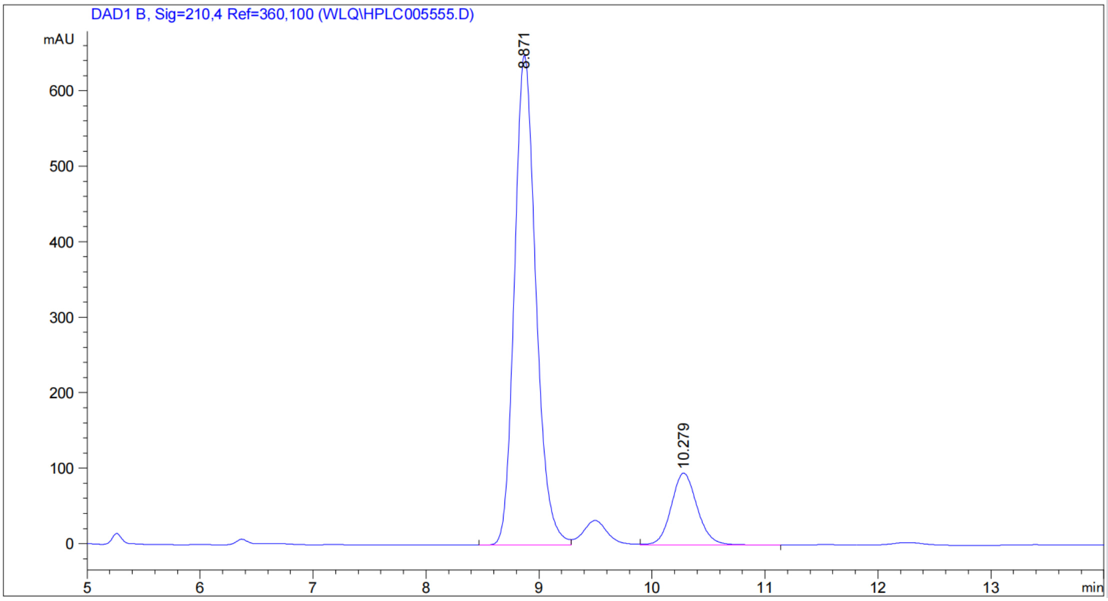

Peak #	Rt time [min]	Type	Width [min]	Peak Area [mAU*s]	Peak Height [mAU]	Peak Area %
1	8.871	BV	0.2020	8564.55078	648.87158	84.8863
2	10.279	VB	0.2468	1524.88782	95.23225	15.1137

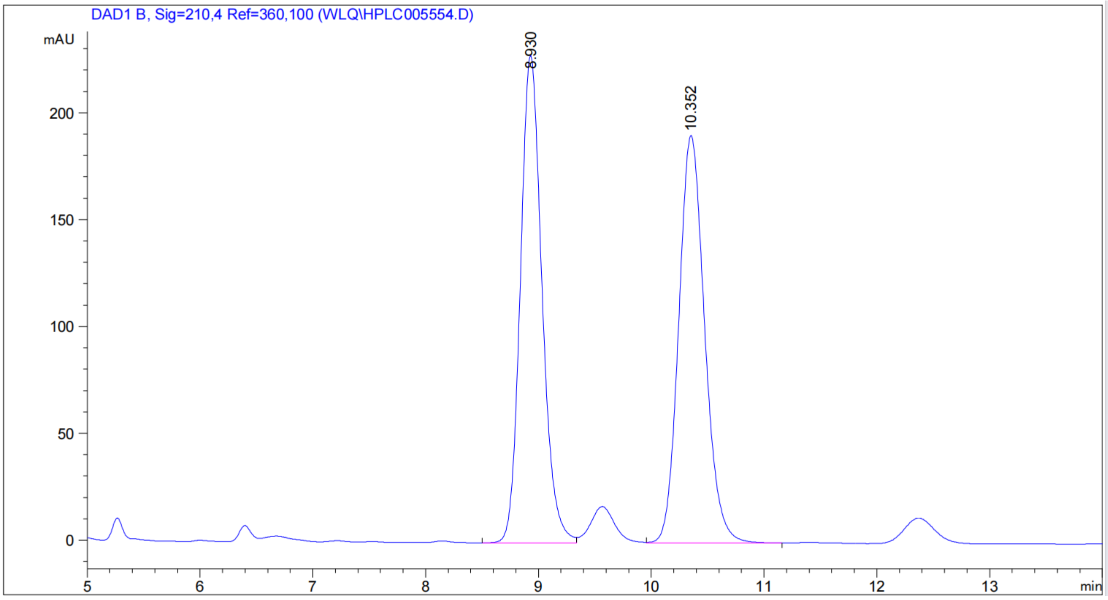

Peak #	Rt time [min]	Type	Width [min]	Peak Area [mAU*s]	Peak Height [mAU]	Peak Area %
1	8.930	BV	0.2031	3034.19189	228.17038	49.9439
2	10.352	VB	0.2461	3041.01172	190.68448	50.0561

^{1}H NMR of diethyl (2*R*,5*S*)-5-(4-isopropylphenyl)-2-phenyl-3-tosyloxazolidine-4,4-dicarboxylate (3ac) (400 MHz, $CDCl_3$)

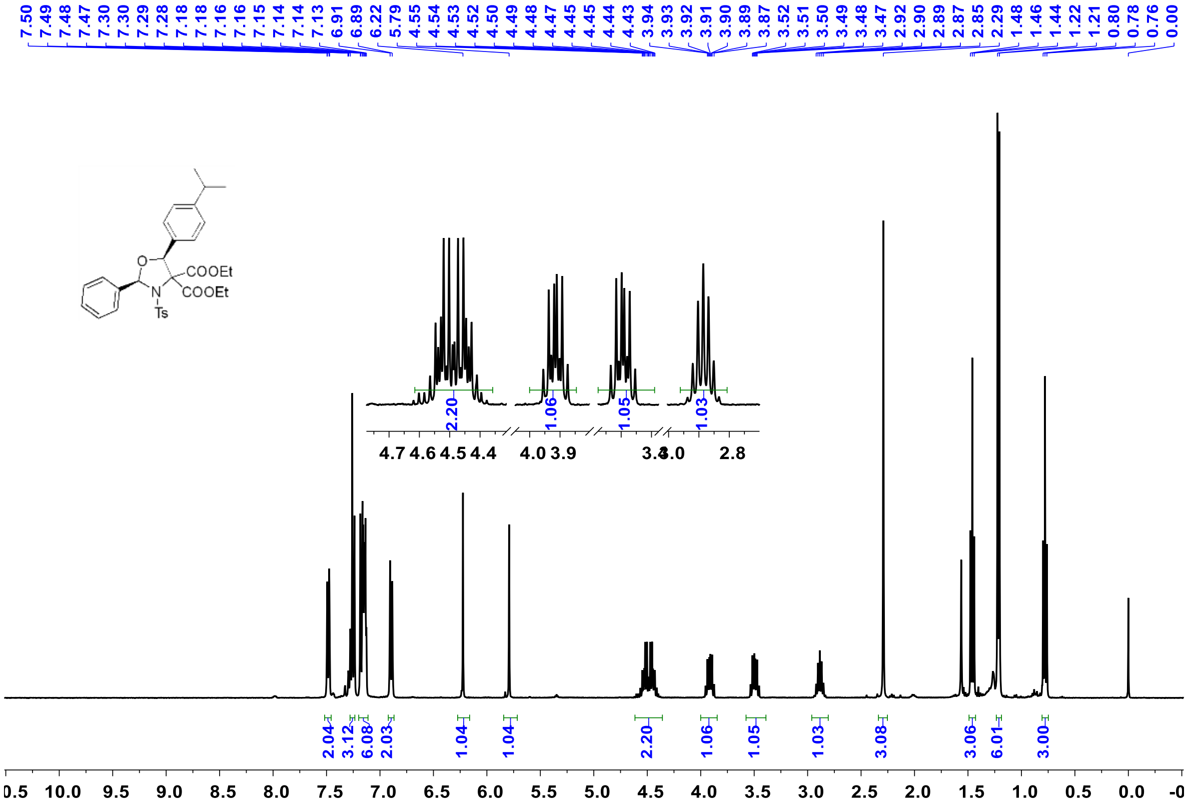

^{13}C{^{1}H} NMR of diethyl (2*R*,5*S*)-5-(4-isopropylphenyl)-2-phenyl-3-tosyloxazolidine-4,4-dicarboxylate (3ac) (101 MHz, $CDCl_3$)

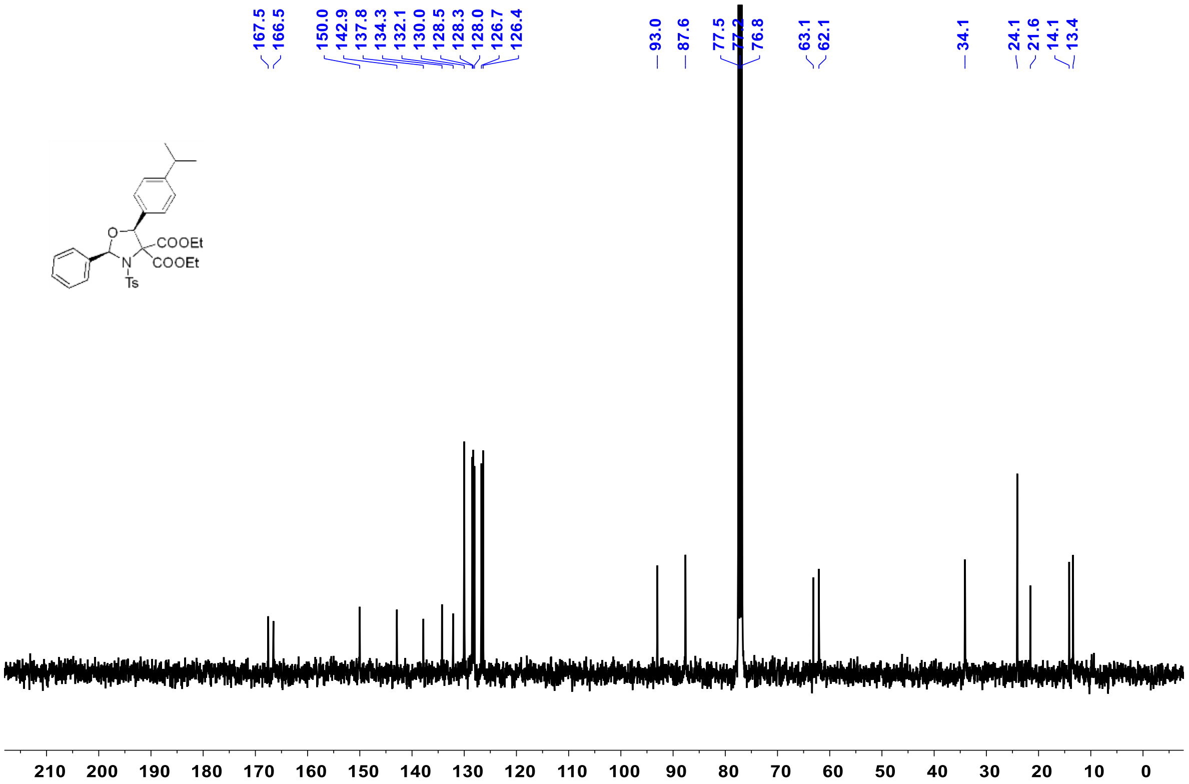

HPLC graph of racemic 3ac

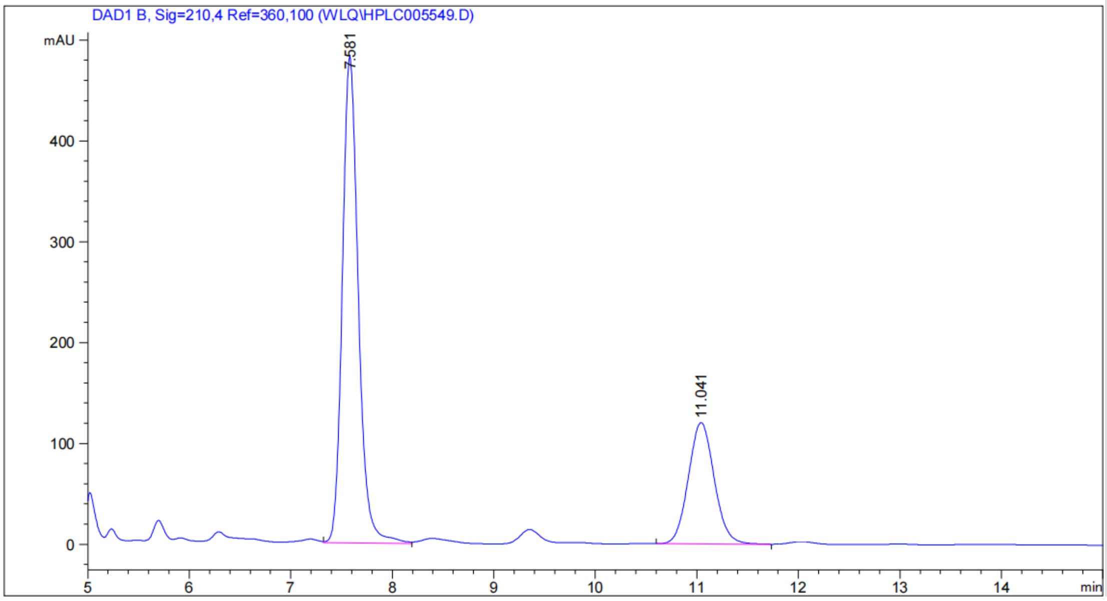

Peak #	Rt time [min]	Type	Width [min]	Peak Area [mAU*s]	Peak Height [mAU]	Peak Area %
1	7.581	VV	0.1673	5263.89990	482.27225	71.2207
2	11.041	BB	0.2744	2127.07178	120.20442	28.7793

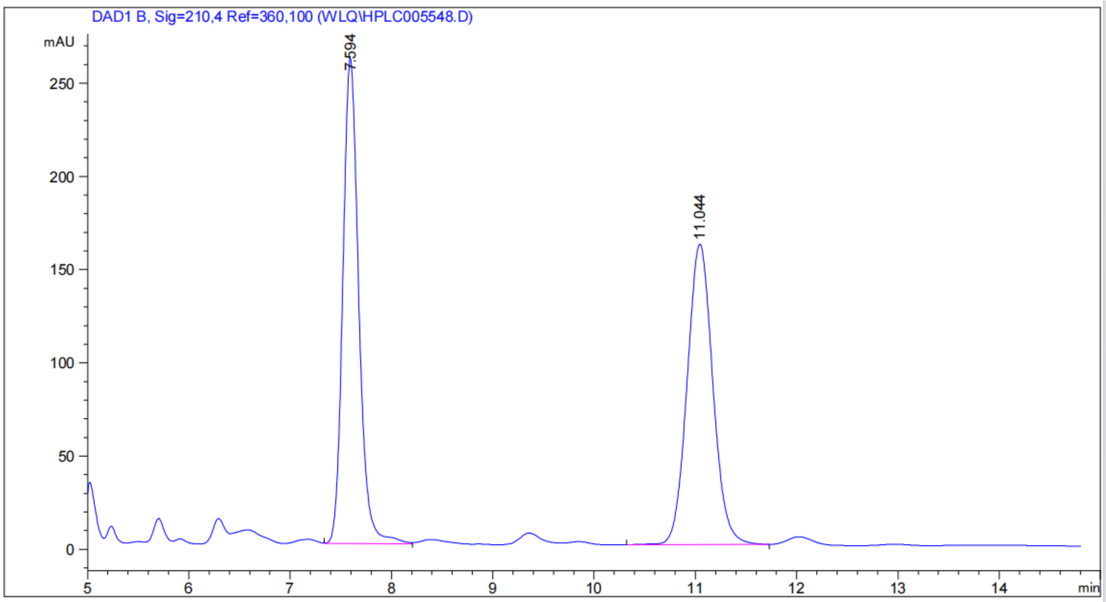

Peak #	Rt time [min]	Type	Width [min]	Peak Area [mAU*s]	Peak Height [mAU]	Peak Area %
1	7.594	VV	0.1673	2843.17993	260.58841	50.0321
2	11.044	BB	0.2736	2839.53687	161.16245	49.9679

HRMS (ESI) of diethyl (2*R*,5*S*)-5-(4-isopropylphenyl)-2-phenyl-3-tosyloxazolidine-4,4-dicarboxylate (3ac)

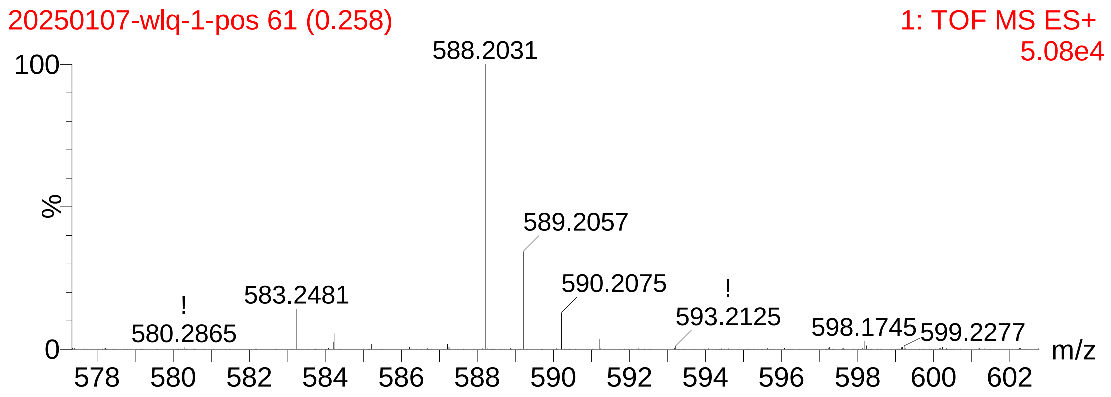

^{1}H NMR of diethyl (2*R*,5*S*)-5-(4-methoxyphenyl)-2-phenyl-3-tosyloxazolidine-4,4-dicarboxylate (3ad) (400 MHz, CDCl$_3$)

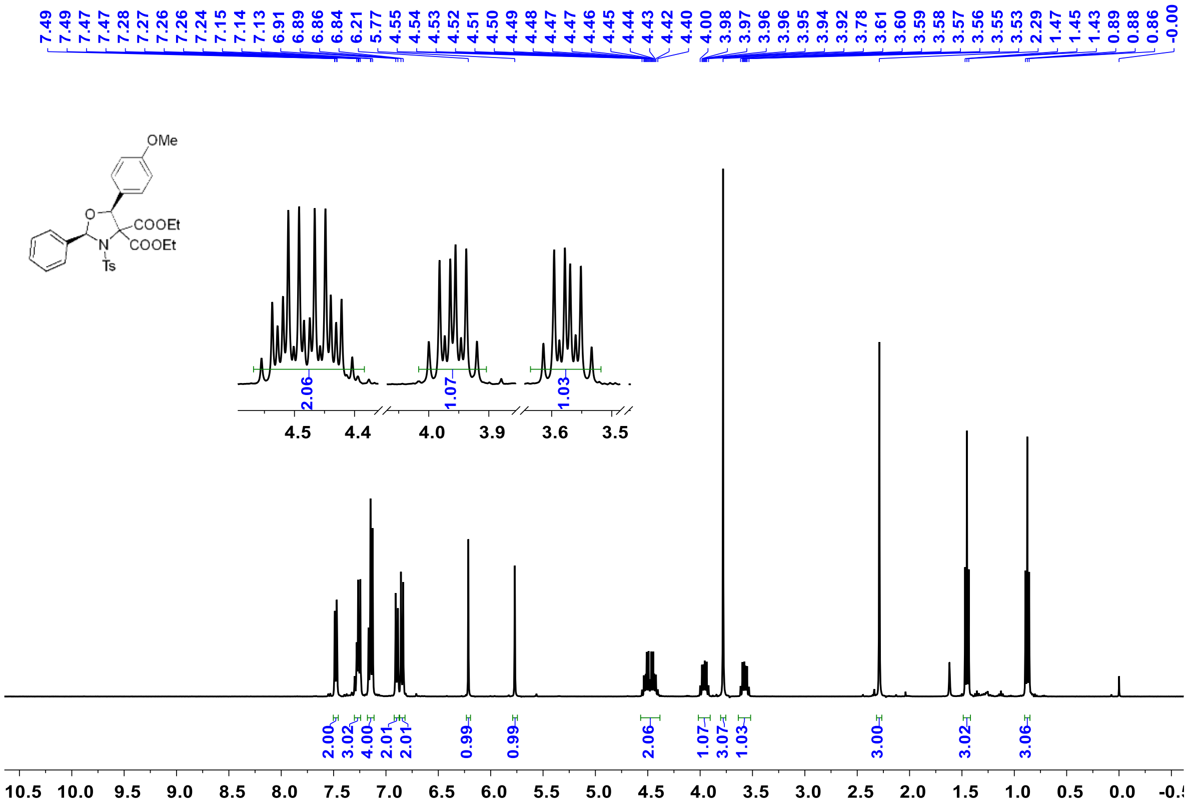

^{13}C{^{1}H} NMR of diethyl (2*R*,5*S*)-5-(4-methoxyphenyl)-2-phenyl-3-tosyloxazolidine-4,4-dicarboxylate (3ad) (101 MHz, CDCl$_3$)

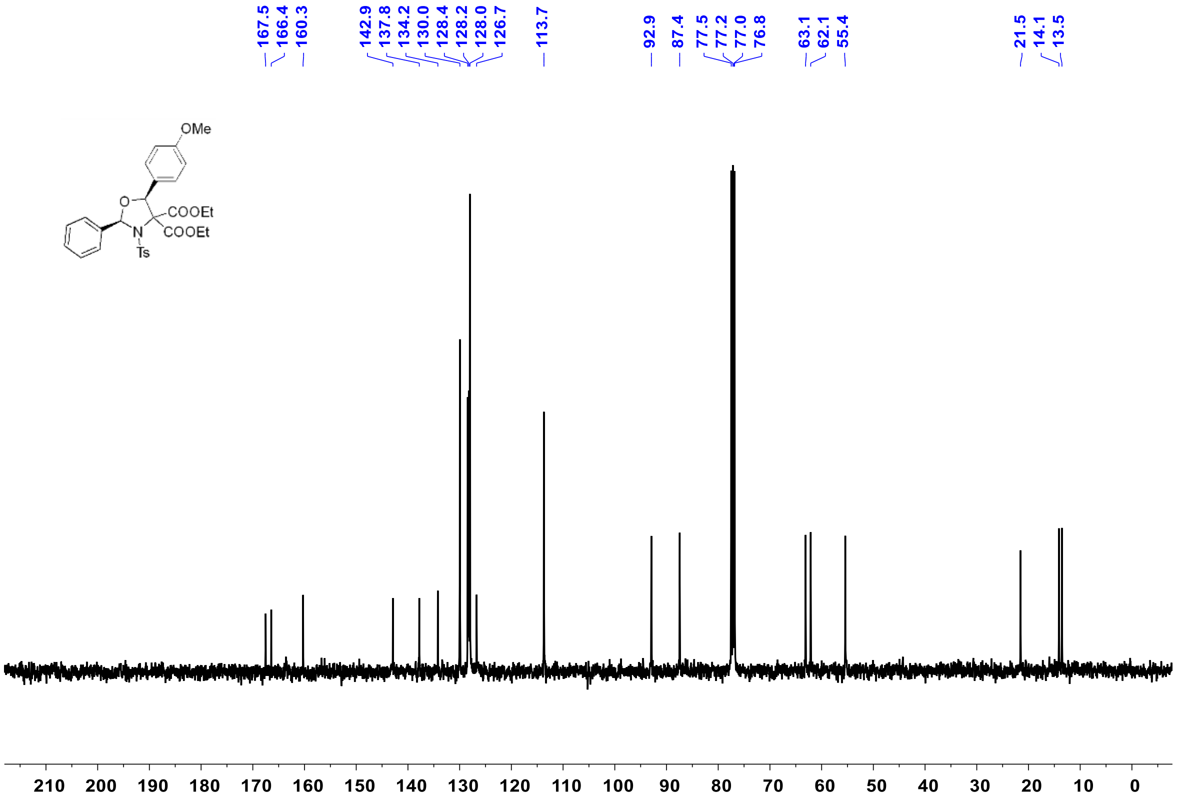

HPLC graph of racemic 3ad

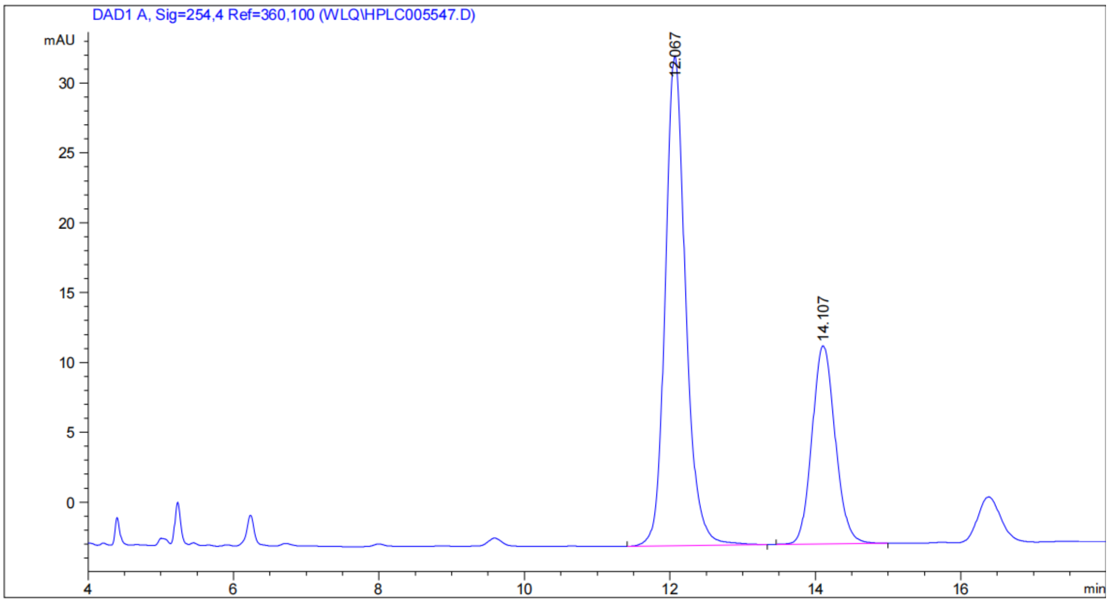

Peak #	Rt time [min]	Type	Width [min]	Peak Area [mAU*s]	Peak Height [mAU]	Peak Area %
1	12.067	BB	0.2974	682.92255	35.03257	68.4762
2	14.107	BB	0.3446	314.39124	14.18221	31.5238

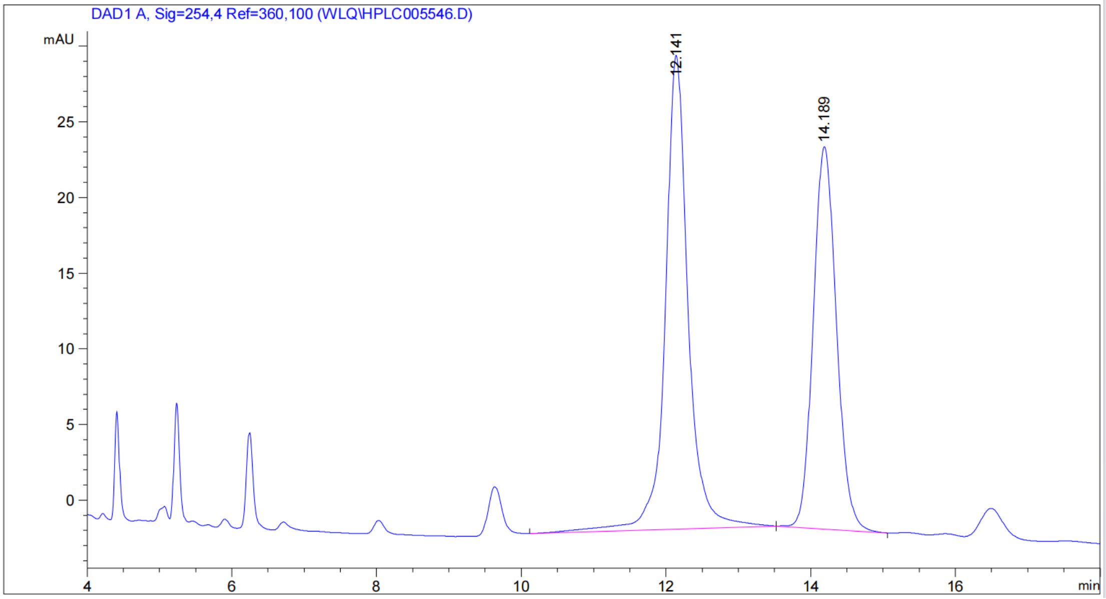

Peak #	Rt time [min]	Type	Width [min]	Peak Area [mAU*s]	Peak Height [mAU]	Peak Area %
1	12.141	BB	0.3278	691.56976	31.29115	55.1208
2	14.189	BB	0.3460	563.07422	25.26398	44.8792

^{1}H NMR of diethyl (2*R*,5*S*)-5-(4-chlorophenyl)-2-phenyl-3-tosyloxazolidine-4,4-dicarboxylate (3ae) (400 MHz, $CDCl_3$)

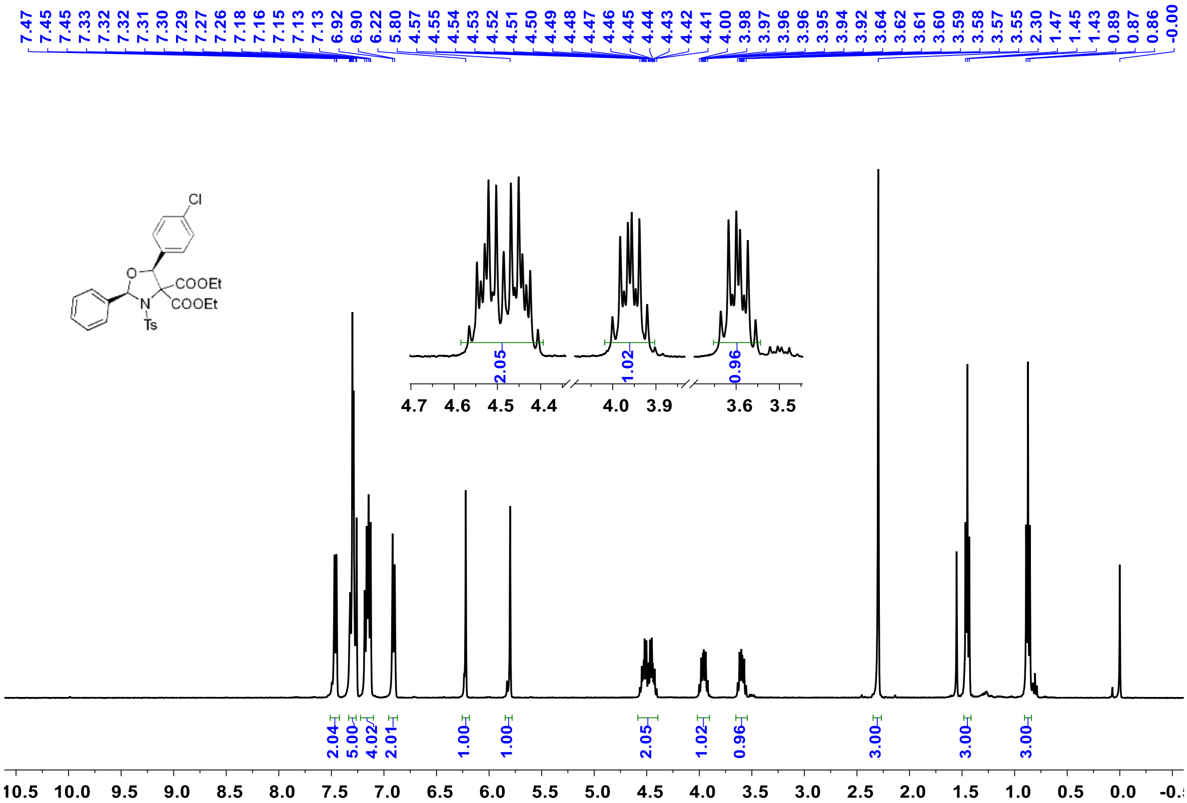

^{13}C{^{1}H} NMR of diethyl (2*R*,5*S*)-5-(4-chlorophenyl)-2-phenyl-3-tosyloxazolidine-4,4-dicarboxylate (3ae) (101 MHz, $CDCl_3$)

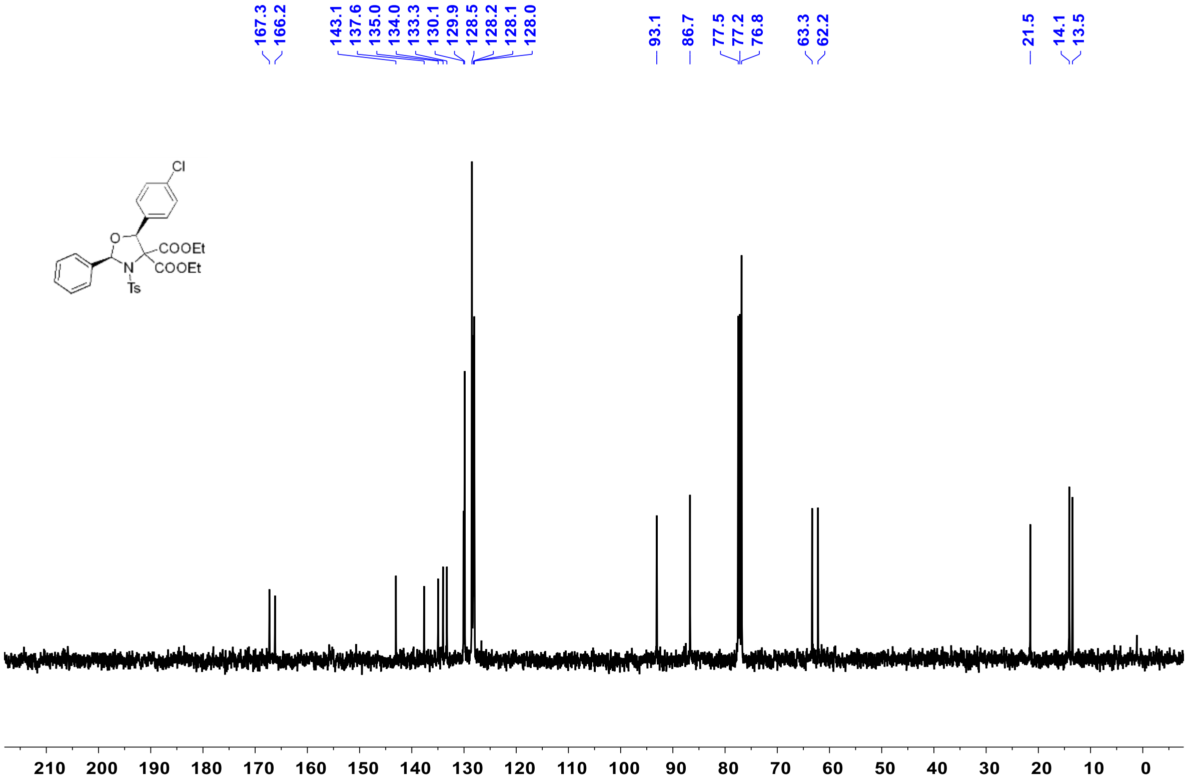

HPLC graph of racemic 3ae

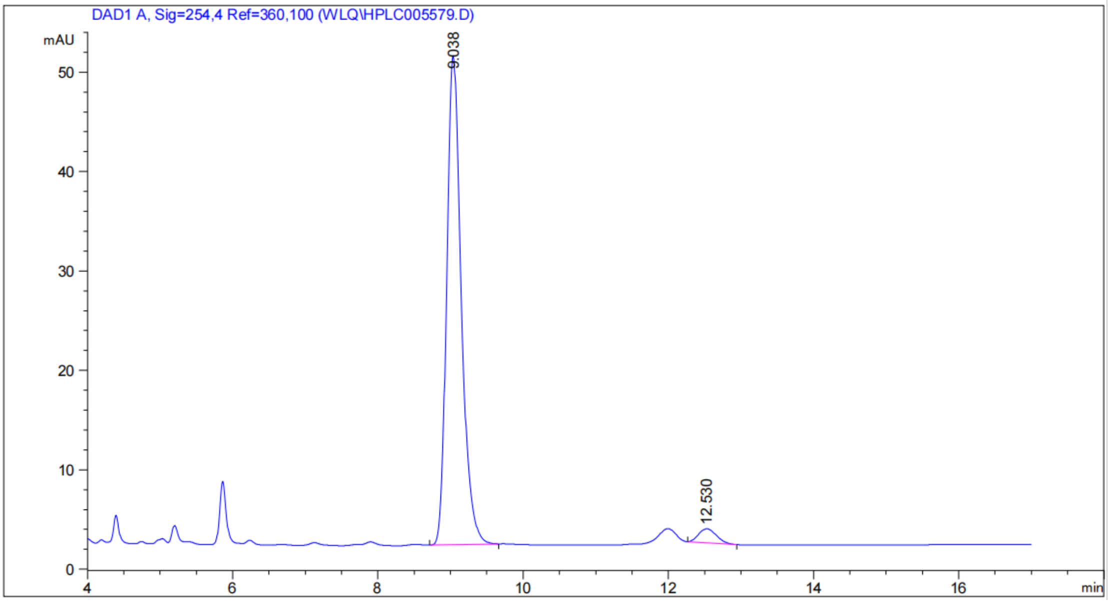

Peak #	Rt time [min]	Type	Width [min]	Peak Area [mAU*s]	Peak Height [mAU]	Peak Area %
1	9.038	BB	0.2100	682.77612	49.16662	96.5739
2	12.530	BB	0.2737	24.22256	1.41487	3.4261

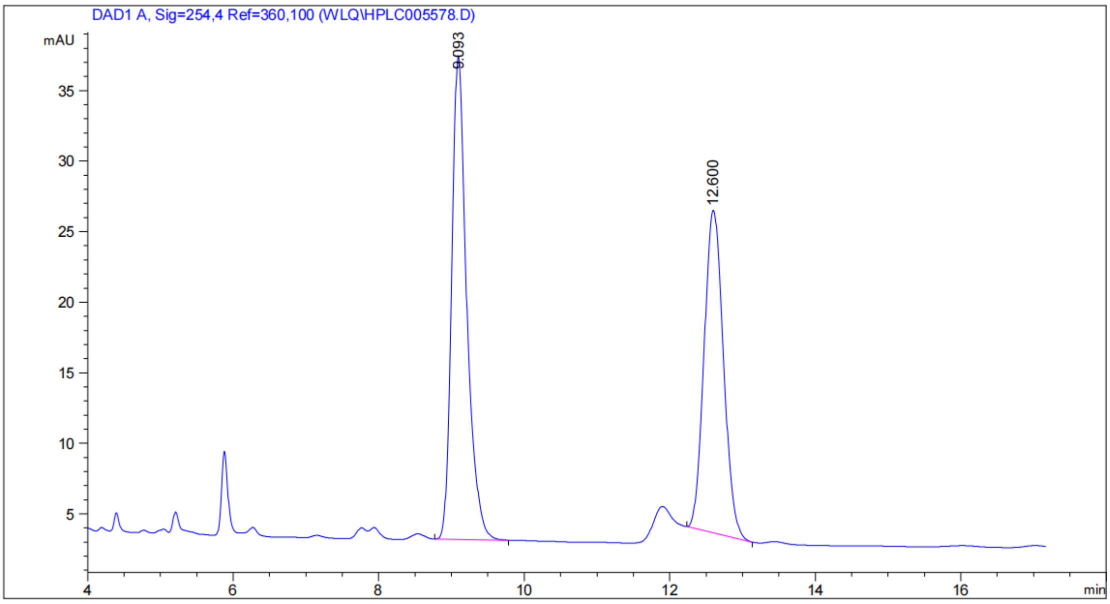

Peak #	Rt time [min]	Type	Width [min]	Peak Area [mAU*s]	Peak Height [mAU]	Peak Area %
1	9.093	BB	0.2156	491.80411	34.22980	53.8291
2	12.600	BB	0.2890	421.83551	22.88559	46.1709

HRMS (ESI) of diethyl (2*R*,5*S*)-5-(4-chlorophenyl)-2-phenyl-3-tosyloxazolidine-4,4-dicarboxylate (3ae)

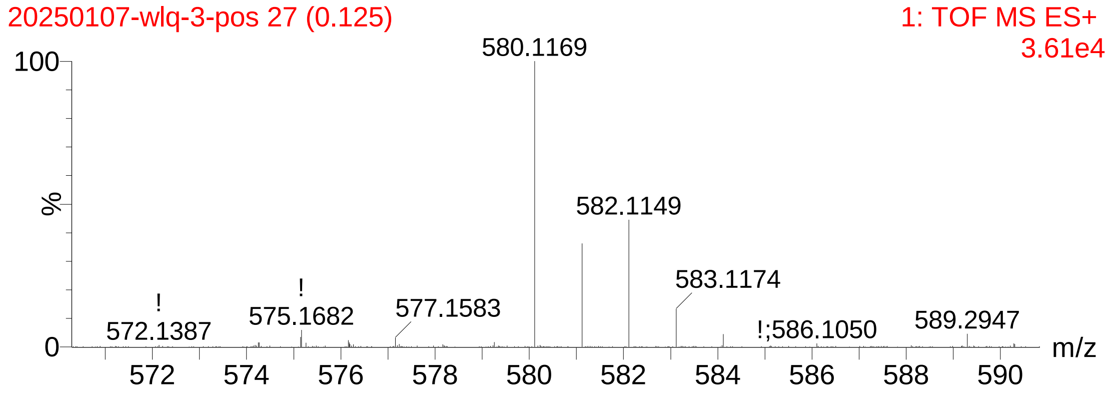

^{1}H NMR of diethyl (2*R*,5*S*)-5-(4-bromophenyl)-2-phenyl-3-tosyloxazolidine-4,4-dicarboxylate (3af) (400 MHz, $CDCl_3$)

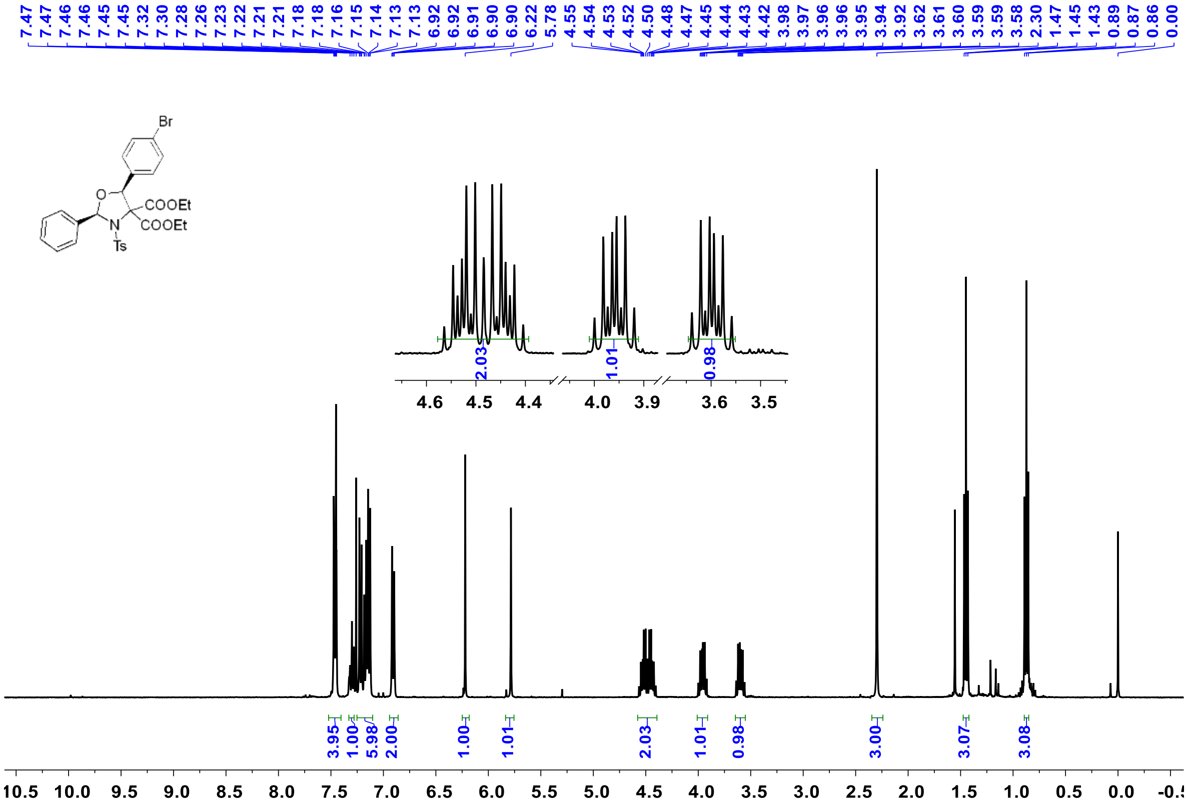

^{13}C{^{1}H} NMR of diethyl (2*R*,5*S*)-5-(4-bromophenyl)-2-phenyl-3-tosyloxazolidine-4,4-dicarboxylate (3af) (101 MHz, $CDCl_3$)

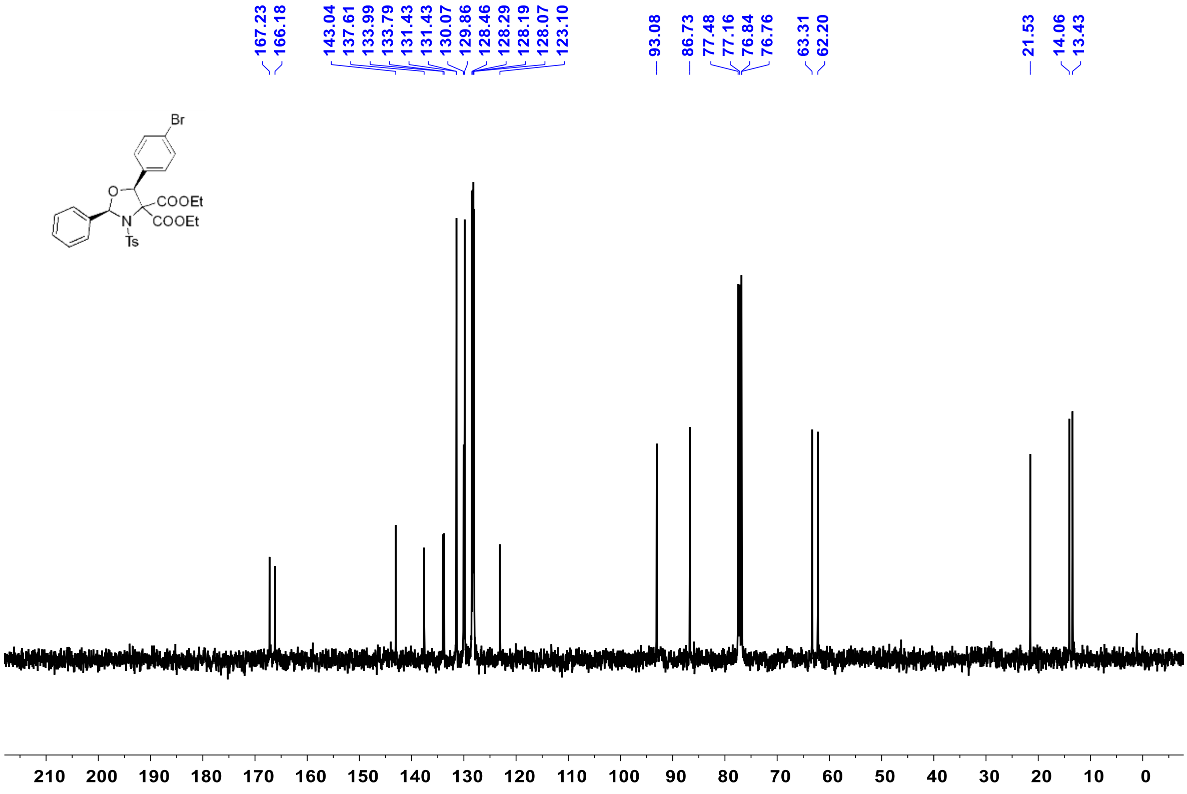

HPLC graph of racemic 3af

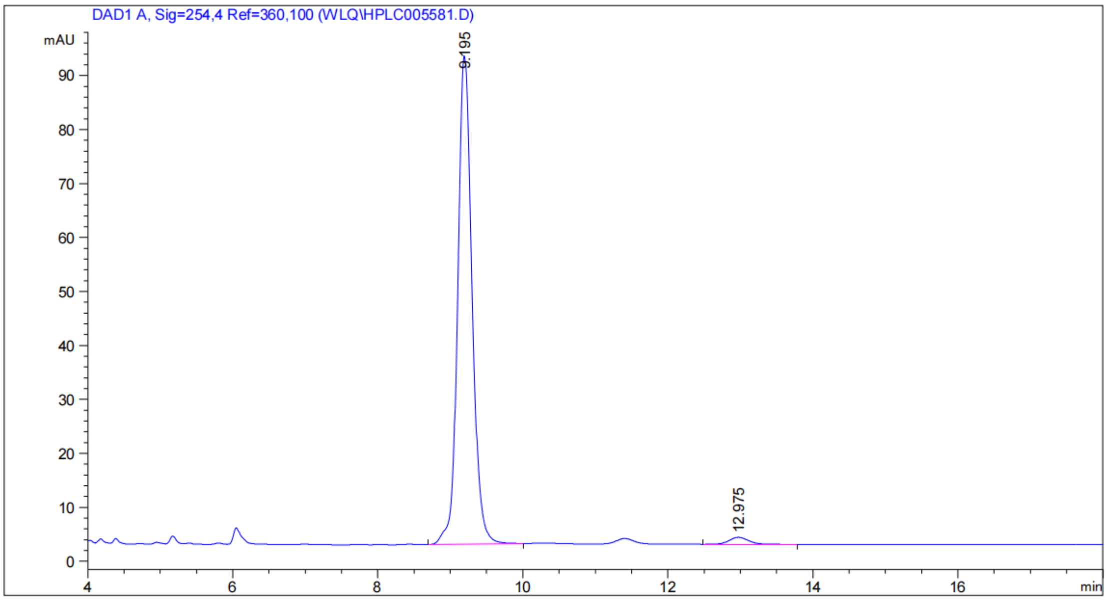

Peak #	Rt time [min]	Type	Width [min]	Peak Area [mAU*s]	Peak Height [mAU]	Peak Area %
1	9.195	BB	0.2101	1241.49023	90.45134	98.0043
2	12.975	BB	0.2977	25.28091	1.30700	1.9957

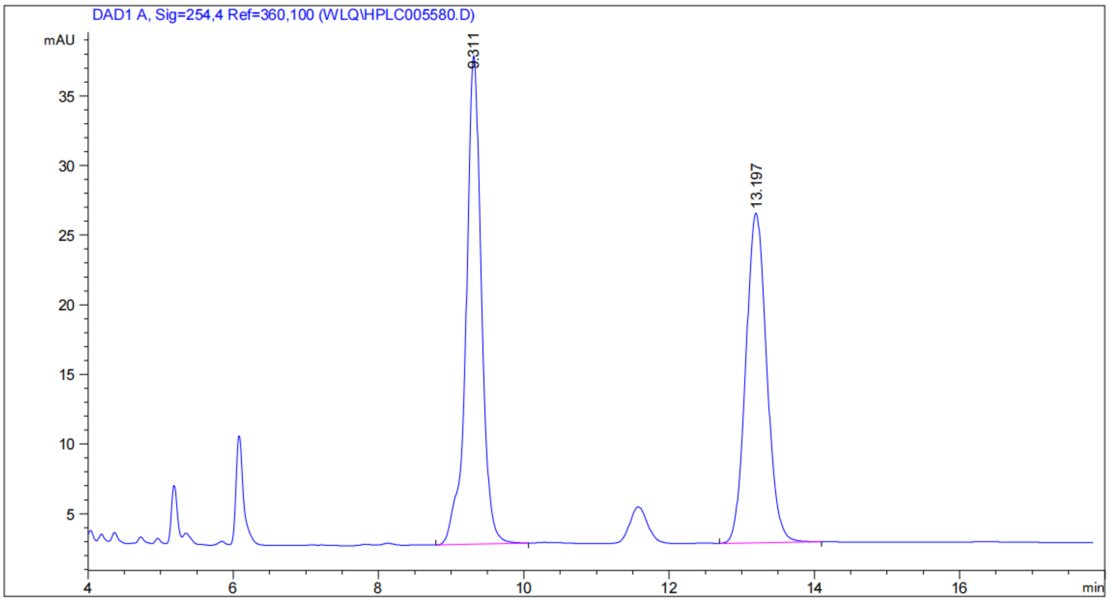

Peak #	Rt time [min]	Type	Width [min]	Peak Area [mAU*s]	Peak Height [mAU]	Peak Area %
1	9.311	BB	0.2212	514.27863	35.04451	52.0569
2	13.197	BB	0.3095	473.63684	23.66868	47.9431

HRMS (ESI) of diethyl (2*R*,5*S*)-5-(4-bromophenyl)-2-phenyl-3-tosyloxazolidine-4,4-dicarboxylate (3af)

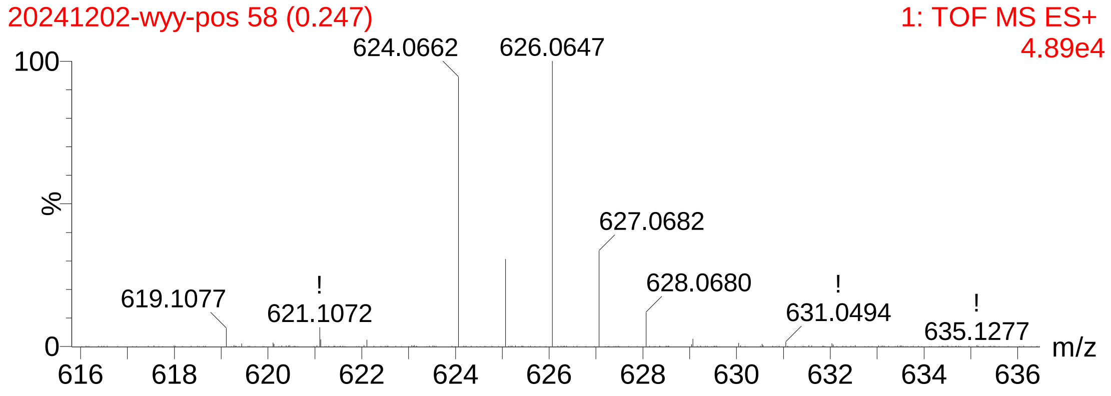

^{1}H NMR of diethyl (2*R*,5*S*)-5-(3-chlorophenyl)-2-phenyl-3-tosyloxazolidine-4,4-dicarboxylate (3ag) (400 MHz, CDCl$_3$)

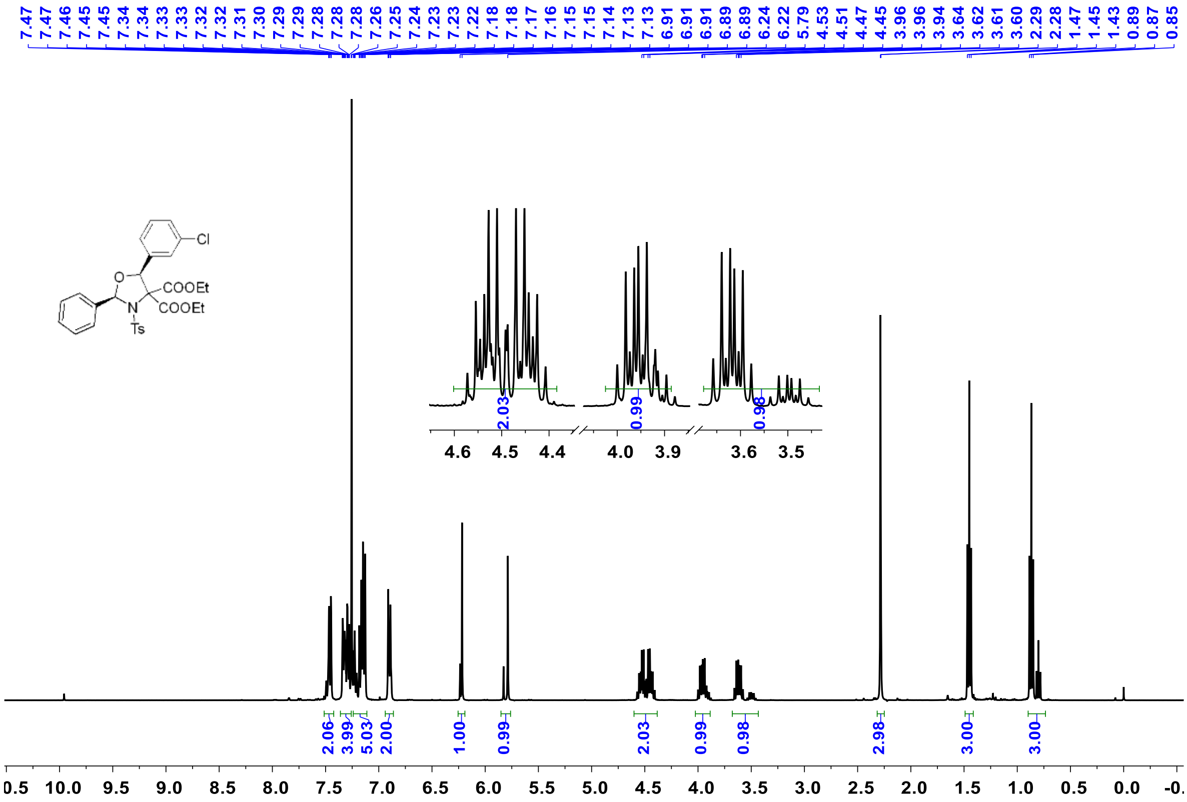

^{13}C{^{1}H} NMR of diethyl (2*R*,5*S*)-5-(3-chlorophenyl)-2-phenyl-3-tosyloxazolidine-4,4-dicarboxylate (3ag) (101 MHz, CDCl$_3$)

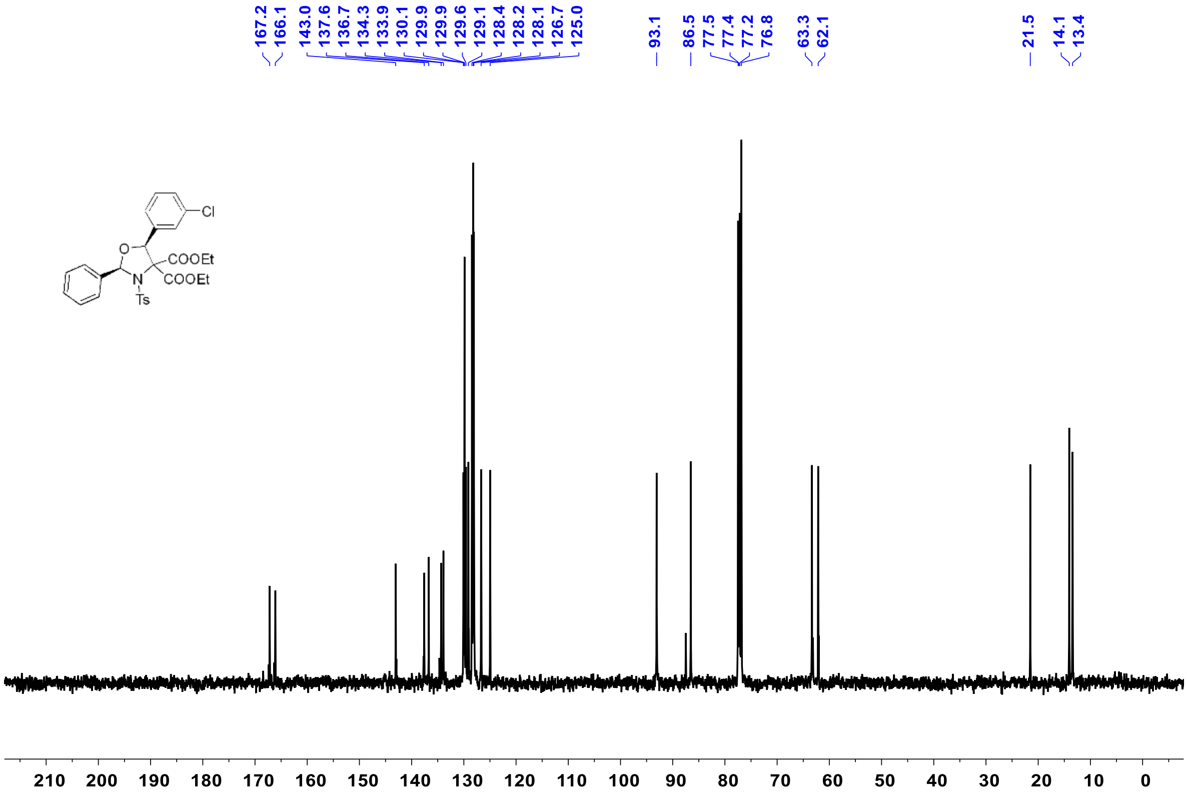

HPLC graph of racemic 3ag

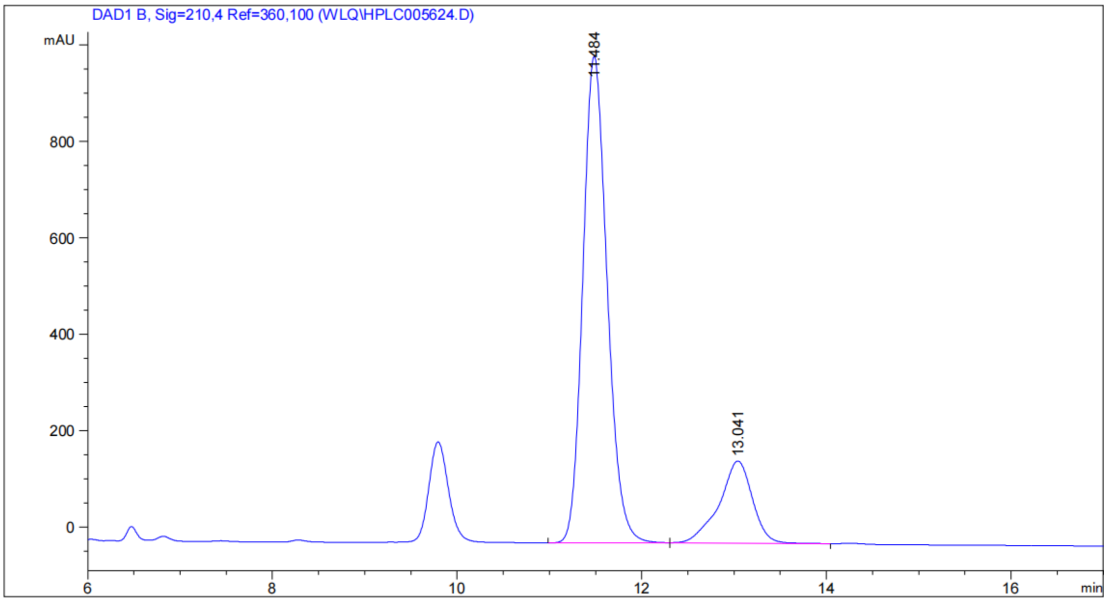

Peak #	Rt time [min]	Type	Width [min]	Peak Area [mAU*s]	Peak Height [mAU]	Peak Area %
1	11.484	BB	0.2835	1.84699e4	1009.07782	81.2782
2	13.041	BB	0.3675	4254.42041	170.25172	18.7218

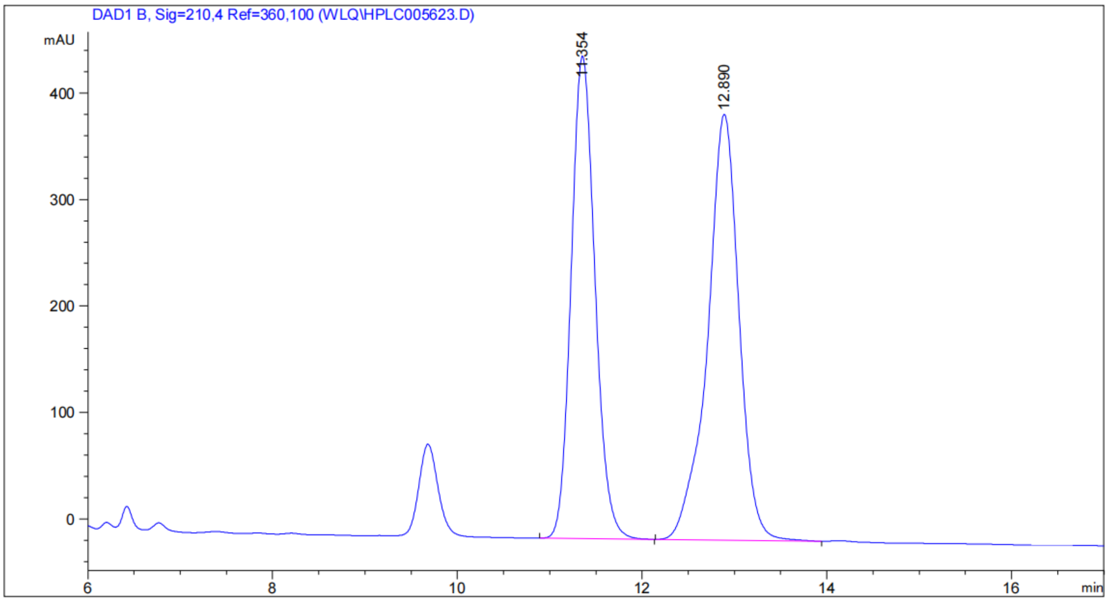

Peak #	Rt time [min]	Type	Width [min]	Peak Area [mAU*s]	Peak Height [mAU]	Peak Area %
1	11.354	BB	0.2792	8120.70605	452.90213	46.1997
2	12.890	BB	0.3539	9456.69824	399.90775	53.8003

HRMS (ESI) of diethyl (2*R*,5*S*)-5-(3-chlorophenyl)-2-phenyl-3-tosyloxazolidine-4,4-dicarboxylate (3ag)

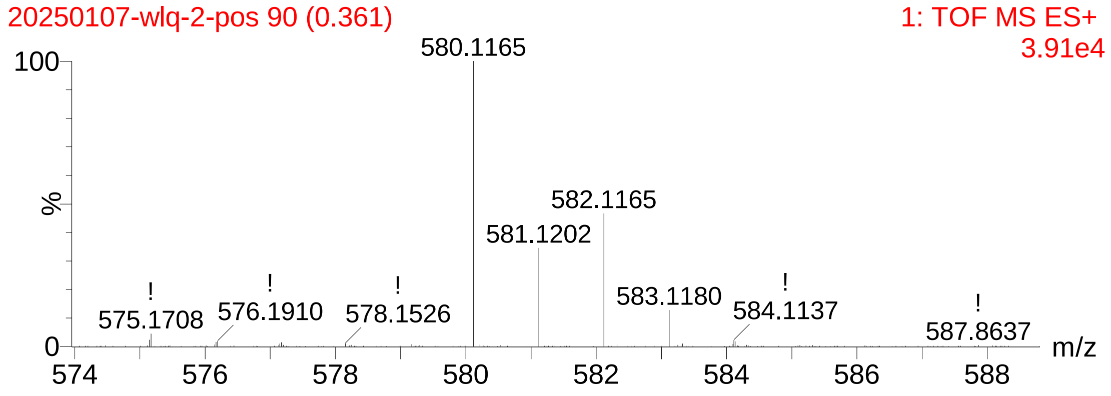

^{1}H NMR of diethyl (2*R*,5*S*)-5-(2,6-dichlorophenyl)-2-phenyl-3-tosyloxazolidine-4,4-dicarboxylate (3ah) (400 MHz, CDCl3)

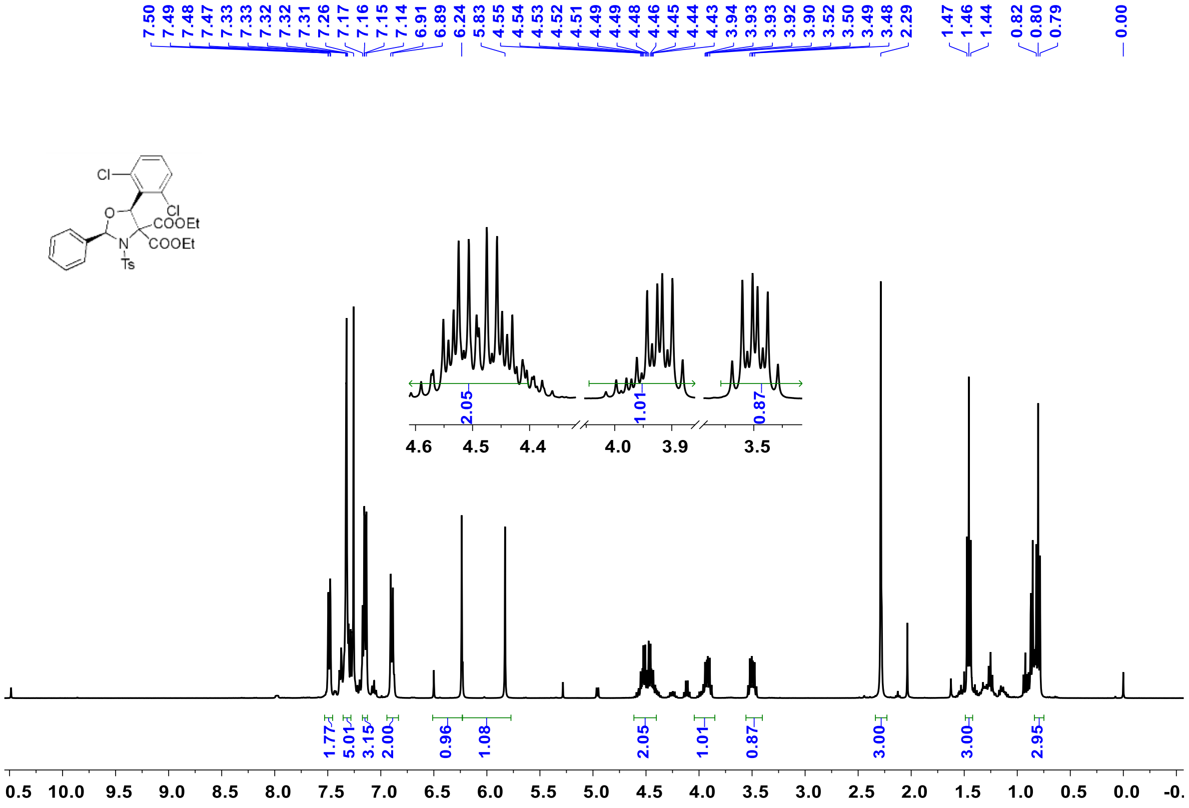

^{13}C{^{1}H} NMR of Diethyl (2*R*,5*S*)-5-(2,6-dichlorophenyl)-2-phenyl-3-tosyloxazolidine-4,4-dicarboxylate (3ah) (101 MHz, CDCl3)

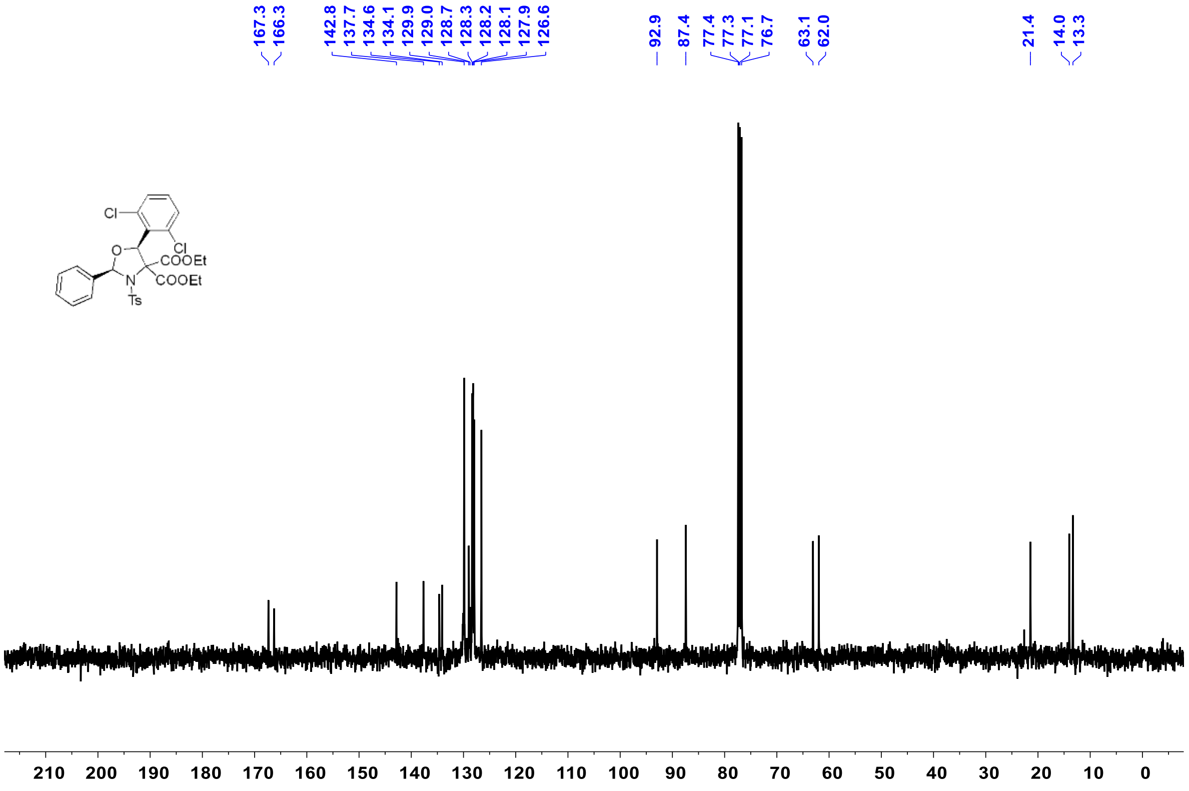

HPLC graph of racemic 3ah

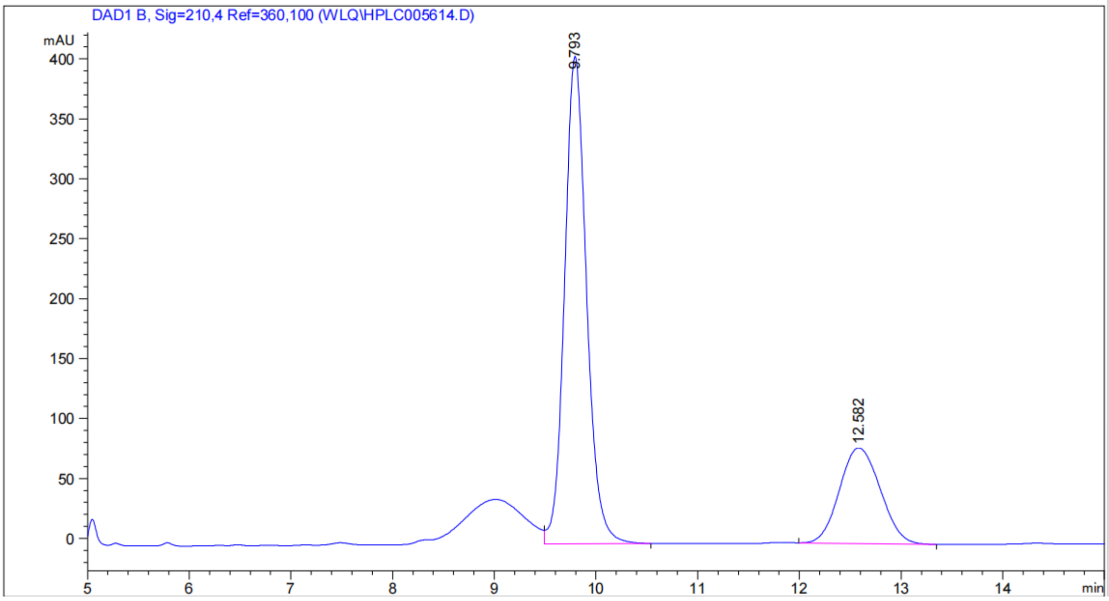

Peak #	Rt time [min]	Type	Width [min]	Peak Area [mAU*s]	Peak Height [mAU]	Peak Area %
1	9.793	VB	0.2291	6101.13086	406.42291	73.3435
2	12.582	BB	0.4442	2217.44287	79.78986	26.6565

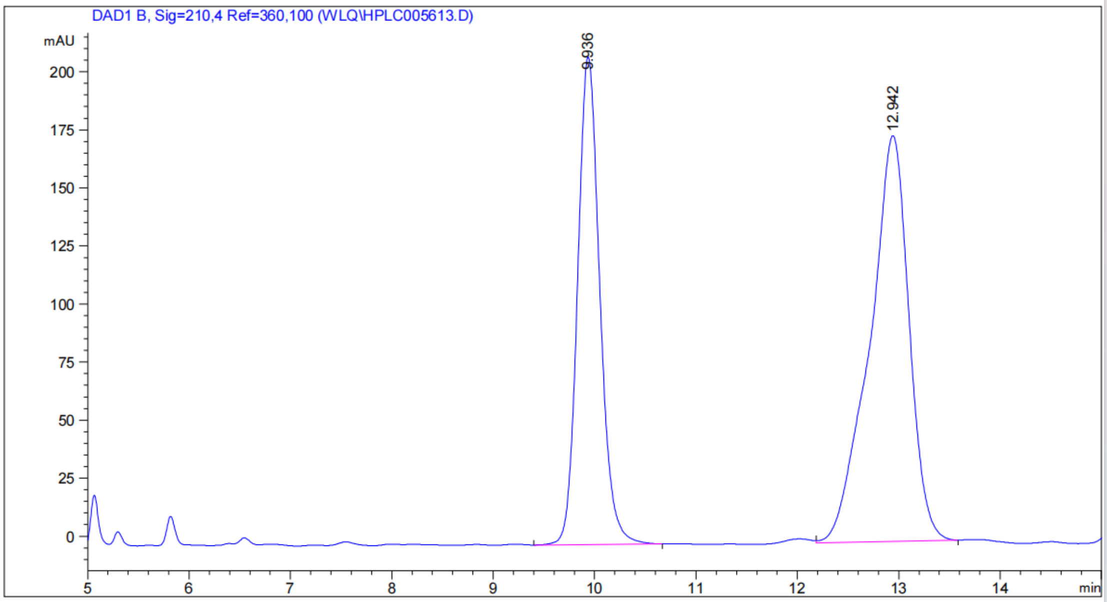

Peak #	Rt time [min]	Type	Width [min]	Peak Area [mAU*s]	Peak Height [mAU]	Peak Area %
1	9.936	BB	0.2331	3187.85254	210.02013	40.3638
2	12.942	VB	0.3923	4709.94580	174.85410	59.6362

HRMS (ESI) of diethyl (2*R*,5*S*)-5-(2,6-dichlorophenyl)-2-phenyl-3-tosyloxazolidine-4,4-dicarboxylate (3ah)

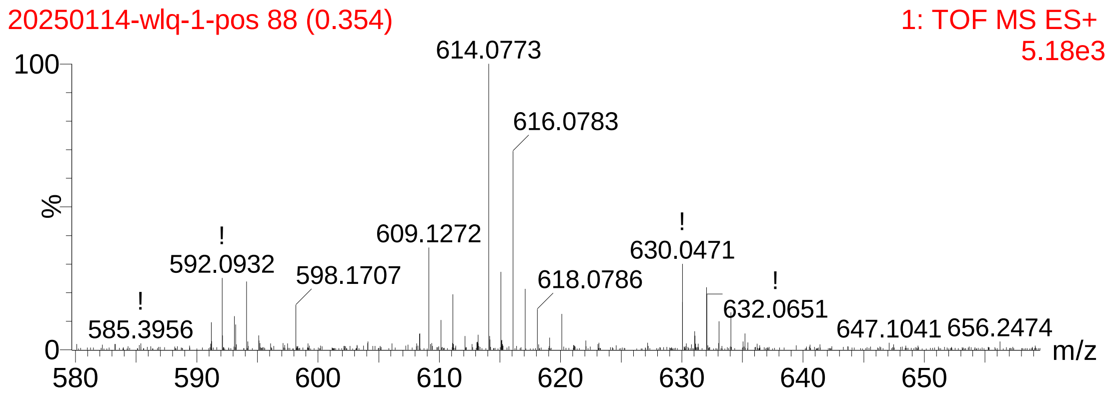

^{1}H NMR of diethyl (2*R*,5*S*)-2-phenyl-3-tosyl-5-(3,4,5-trimethoxyphenyl)oxazolidine-4,4-dicarboxylate (3ai) (400 MHz, CDCl$_3$)

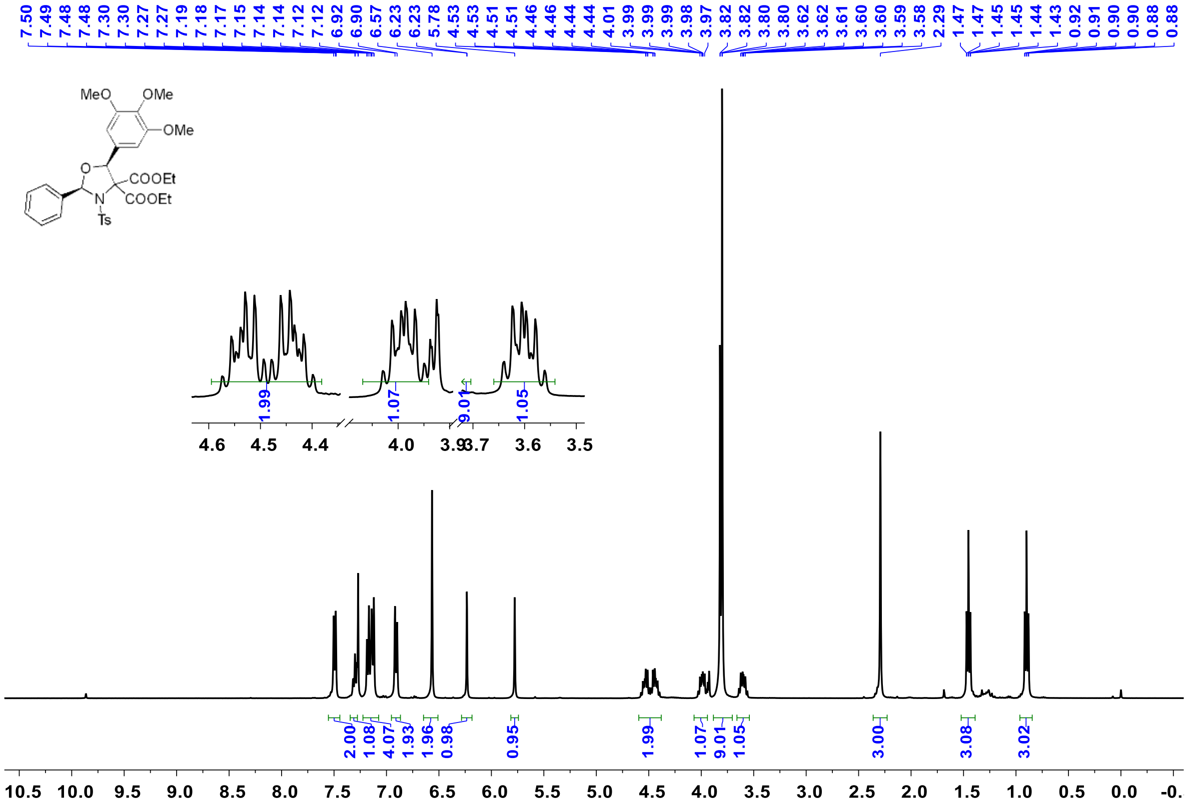

^{13}C{^{1}H} NMR of diethyl (2*R*,5*S*)-2-phenyl-3-tosyl-5-(3,4,5-trimethoxyphenyl)oxazolidine-4,4-dicarboxylate (3ai) (101 MHz, CDCl$_3$)

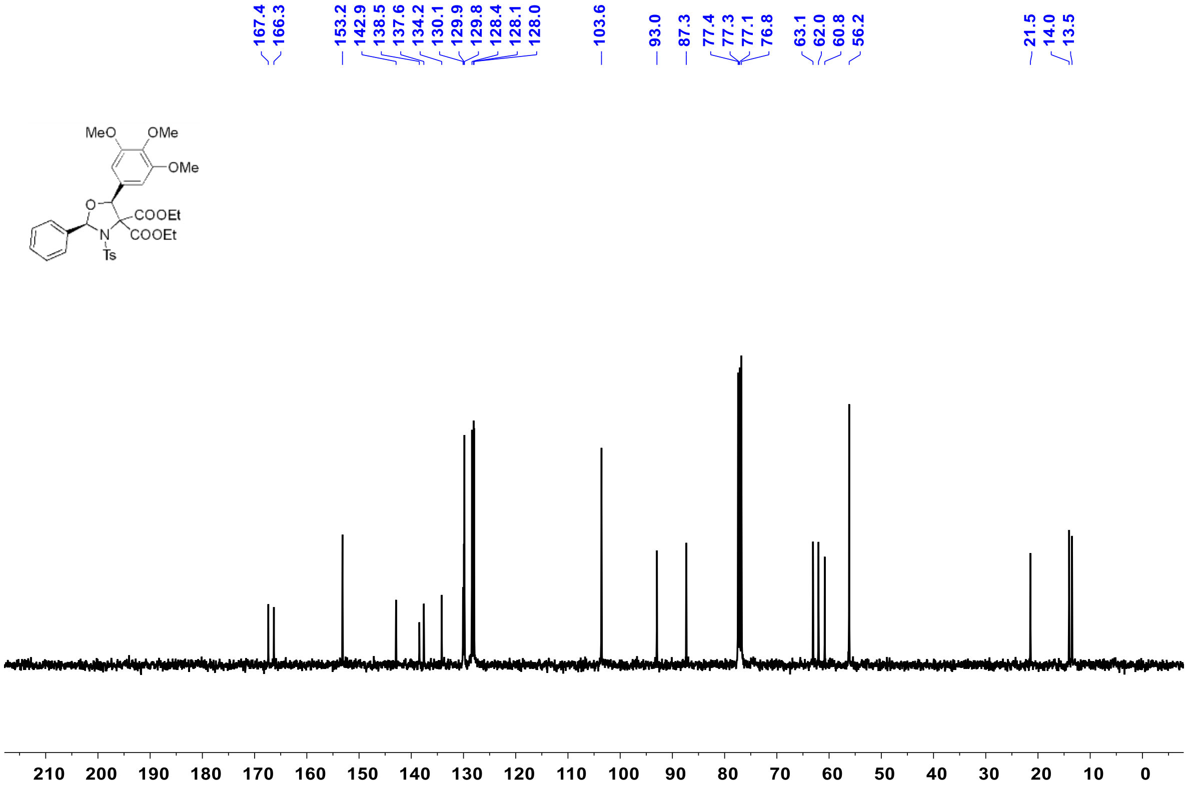

HPLC graph of racemic 3ai

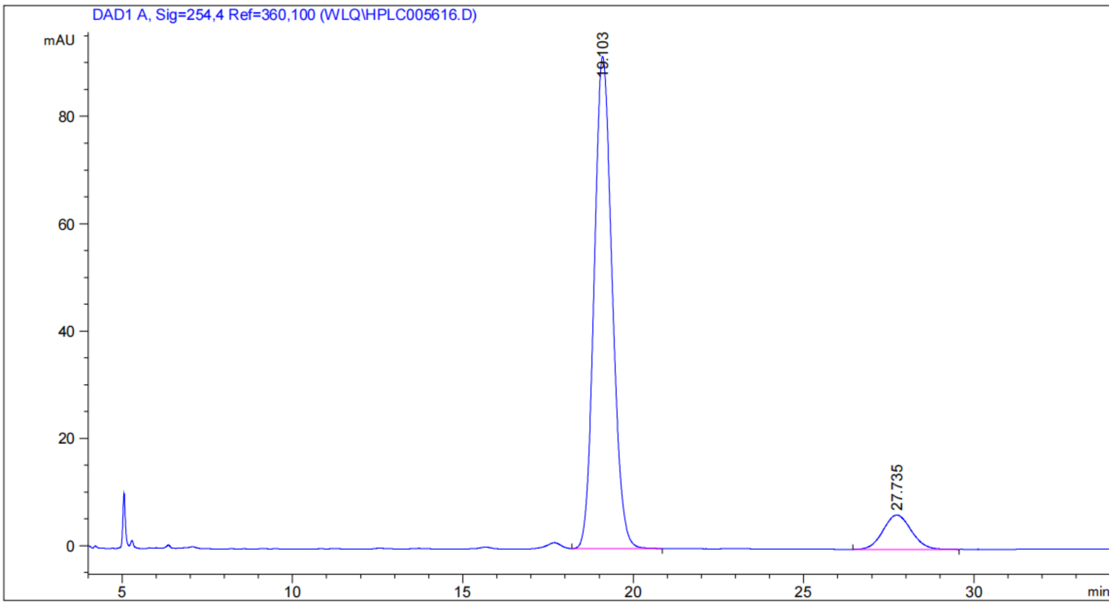

Peak	Rt time	Type	Width	Peak Area	Peak Height	Peak Area
#	[min]		[min]	[mAU*s]	[mAU]	%
1	19.103	BB	0.5702	3364.03540	91.66433	90.1798
2	27.735	BB	0.8838	366.32816	6.38102	9.8202

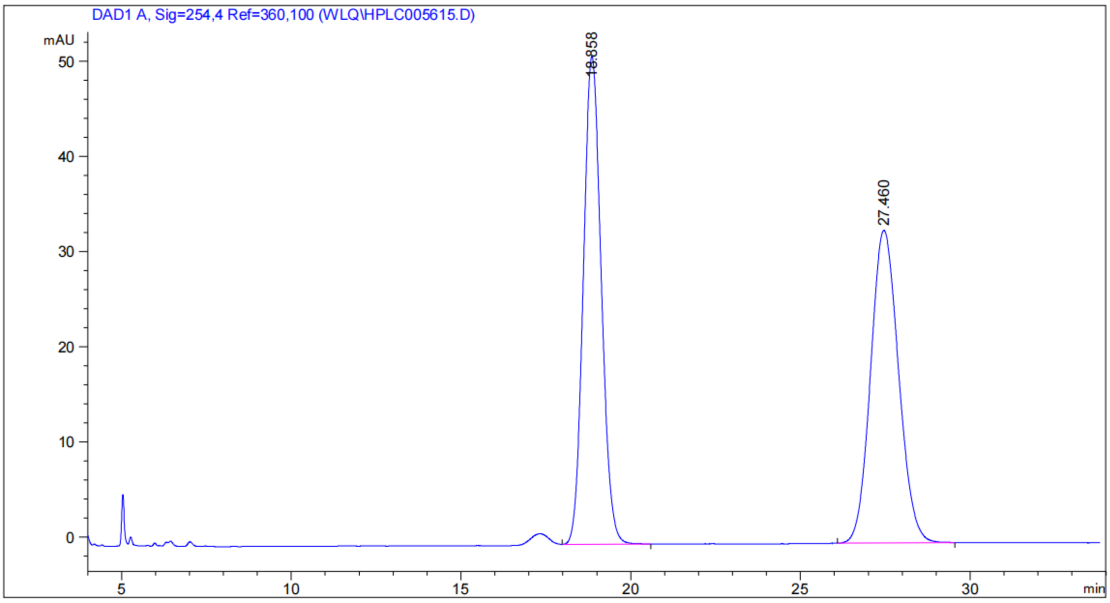

Peak	Rt time	Type	Width	Peak Area	Peak Height	Peak Area
#	[min]		[min]	[mAU*s]	[mAU]	%
1	18.858	BB	0.5664	1874.91370	51.30067	49.9391
2	27.460	BB	0.8892	1879.48926	32.86562	50.0609

^{1}H NMR of diethyl (2*R*,5*S*)-5-(furan-2-yl)-2-phenyl-3-tosyloxazolidine-4,4-dicarboxylate (3aj) (400 MHz, $CDCl_3$)

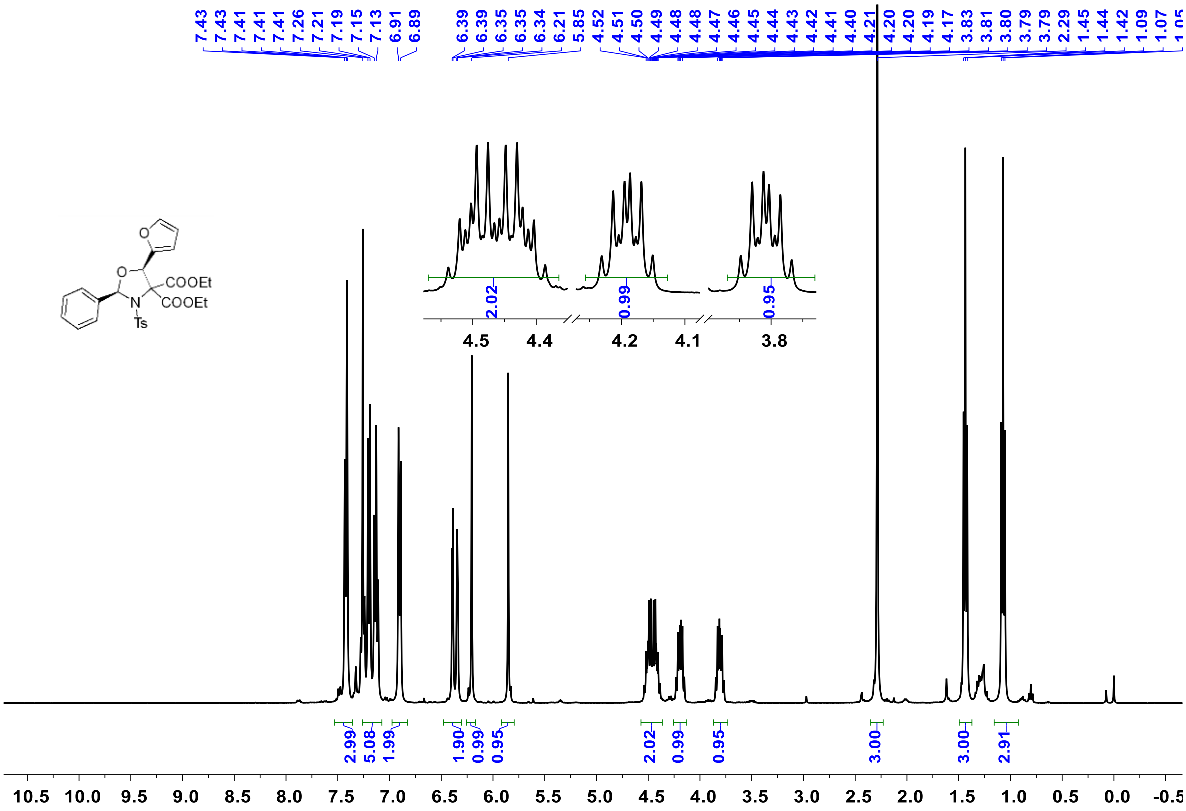

^{13}C{^{1}H} NMR of diethyl (2*R*,5*S*)-5-(furan-2-yl)-2-phenyl-3-tosyloxazolidine-4,4-dicarboxylate (3aj) (101 MHz, $CDCl_3$)

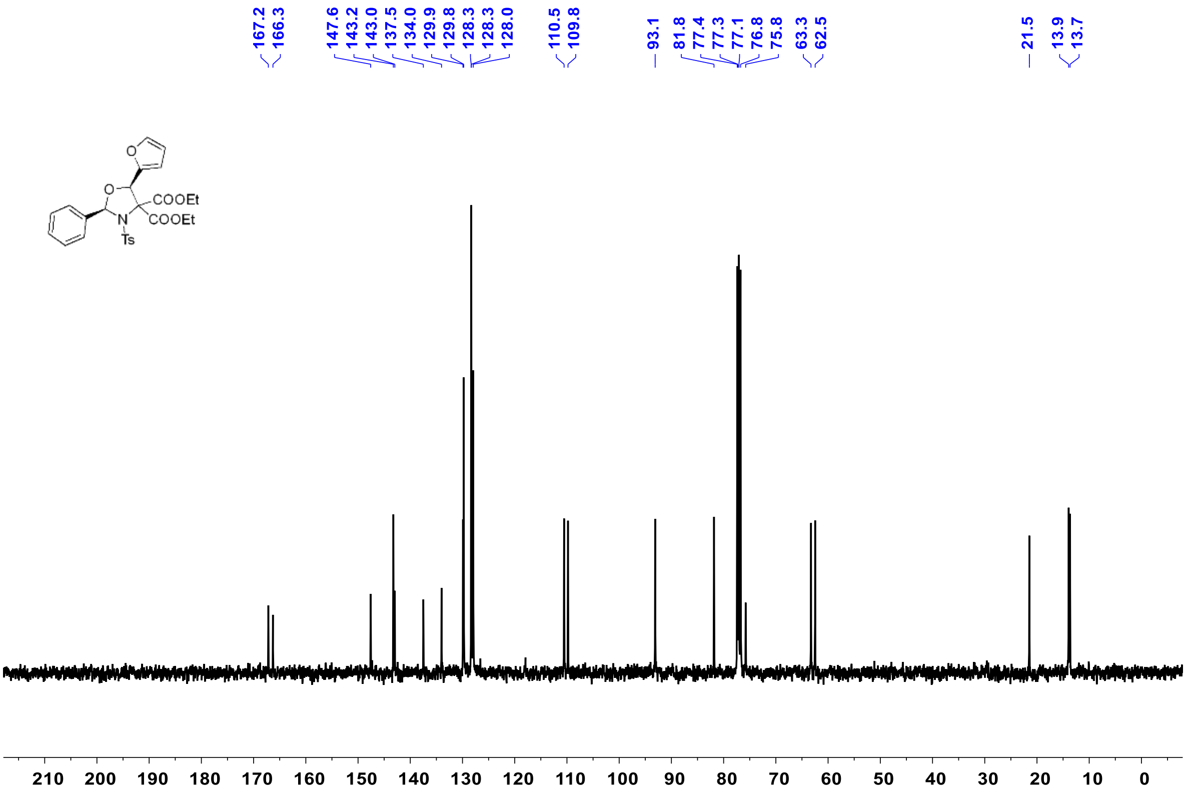

HPLC graph of racemic 3aj

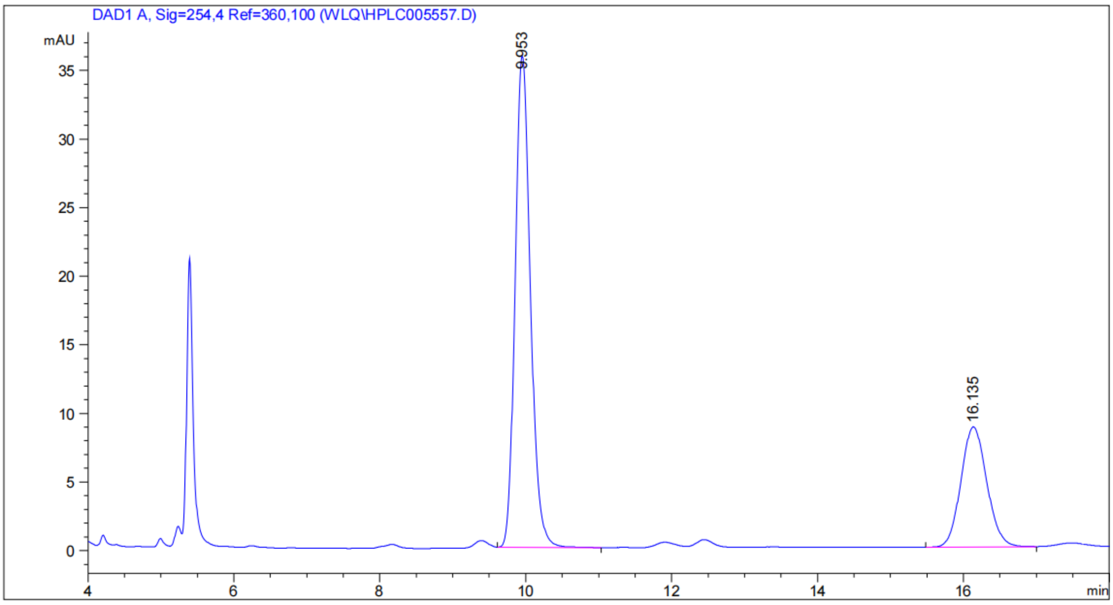

Peak #	Rt time [min]	Type	Width [min]	Peak Area [mAU*s]	Peak Height [mAU]	Peak Area %
1	9.953	BB	0.2239	521.19073	35.78238	70.7457
2	16.135	BB	0.3832	215.51941	8.75616	29.2543

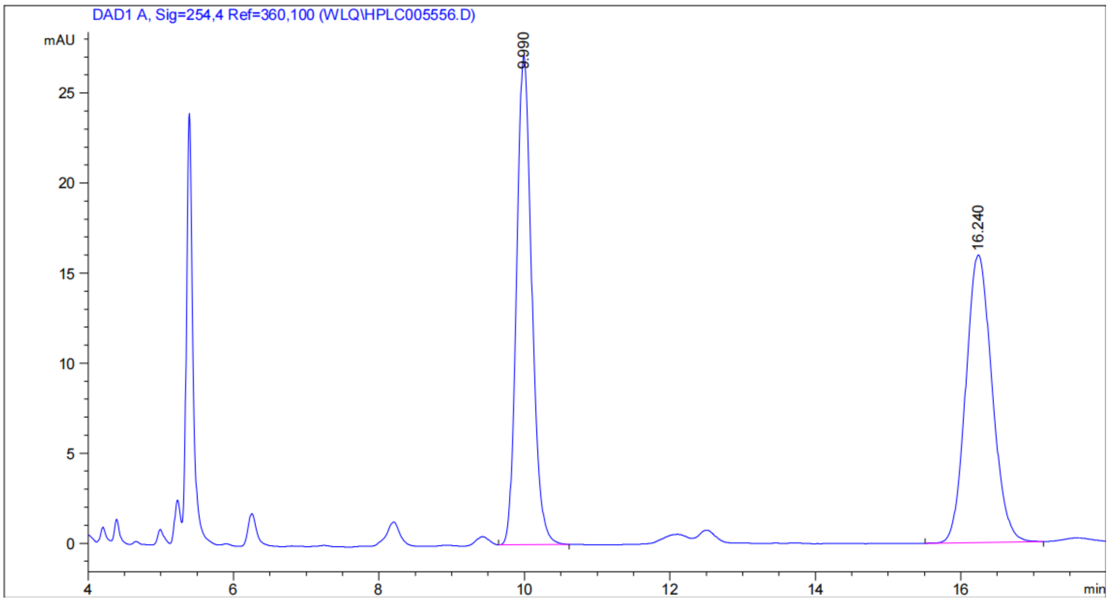

Peak #	Rt time [min]	Type	Width [min]	Peak Area [mAU*s]	Peak Height [mAU]	Peak Area %
1	9.990	BB	0.2259	395.15747	27.13070	49.6947
2	16.240	BB	0.3902	400.01346	15.96898	50.3053

HRMS (ESI) of diethyl (2*R*,5*S*)-5-(furan-2-yl)-2-phenyl-3-tosyloxazolidine-4,4-dicarboxylate (3aj)

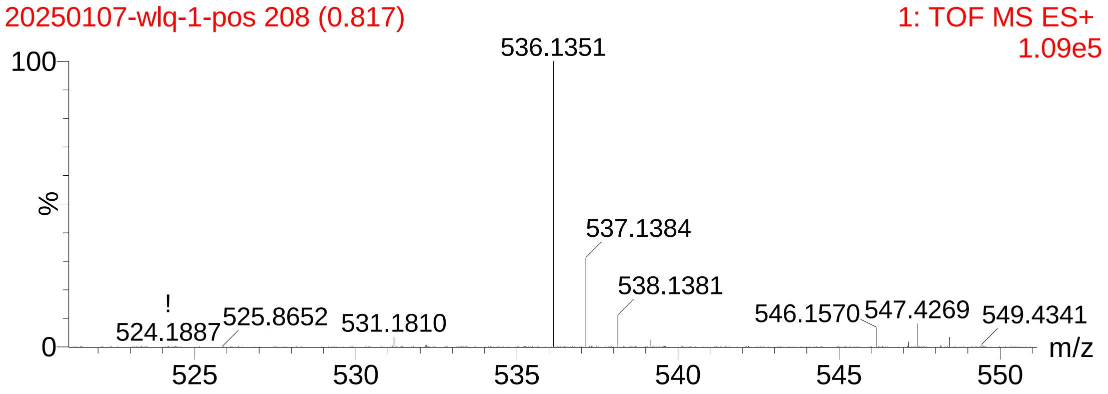

^{1}H NMR of diethyl (2*R*,5*S*)-3-(methylsulfonyl)-2,5-diphenyloxazolidine-4,4-dicarboxylate (3ba) (400 MHz, $CDCl_3$)

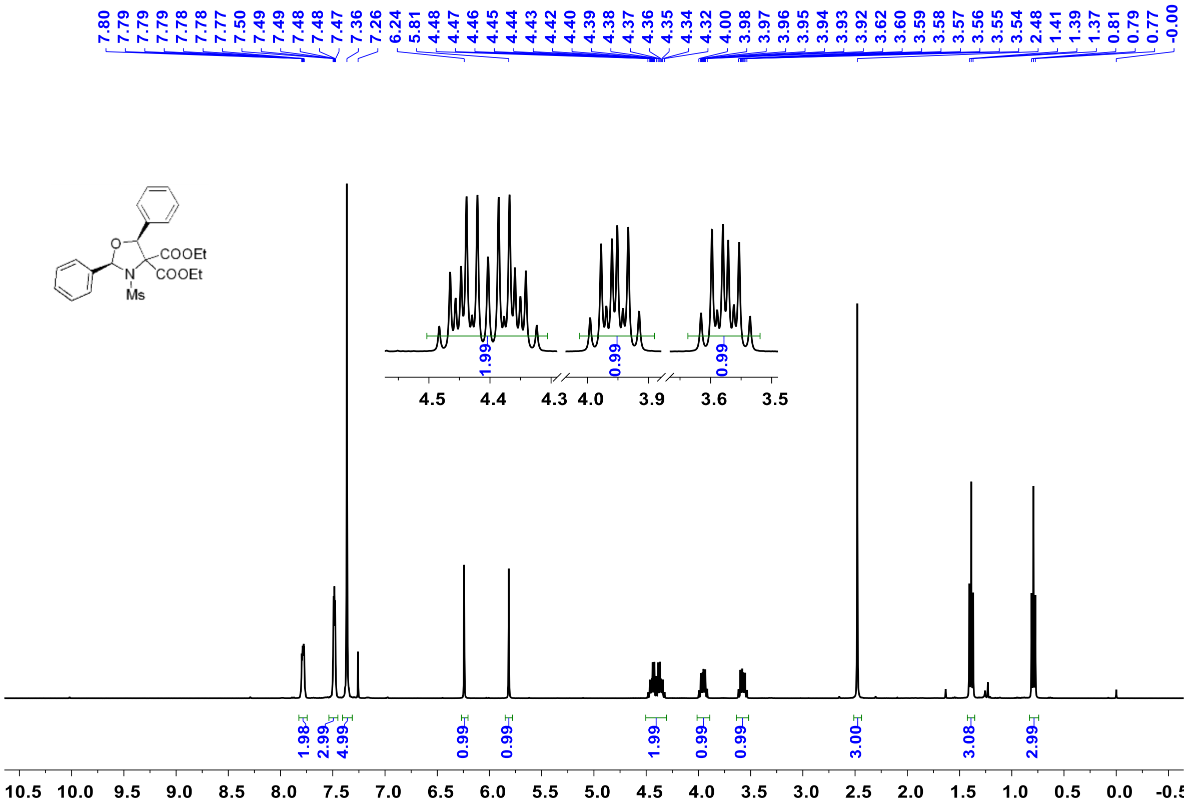

^{13}C{^{1}H} NMR of diethyl (2*R*,5*S*)-3-(methylsulfonyl)-2,5-diphenyloxazolidine-4,4-dicarboxylate (3ba) (101 MHz, $CDCl_3$)

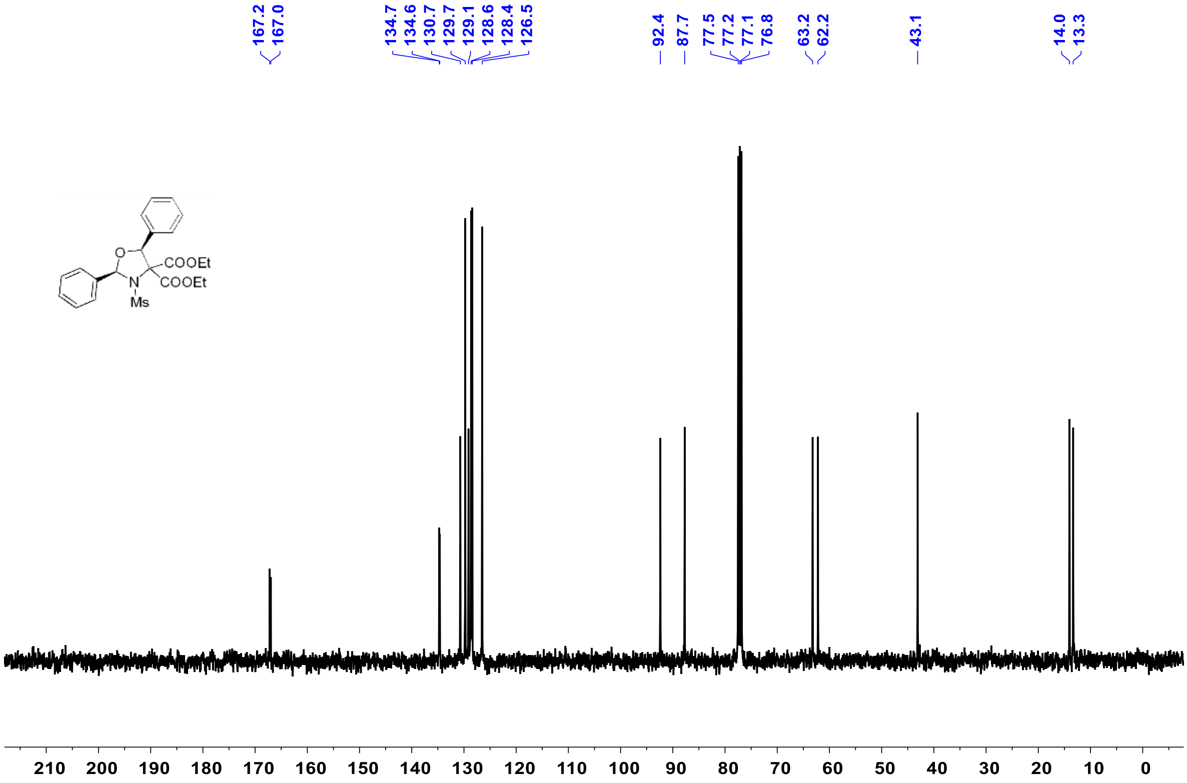

HPLC graph of racemic 3ba

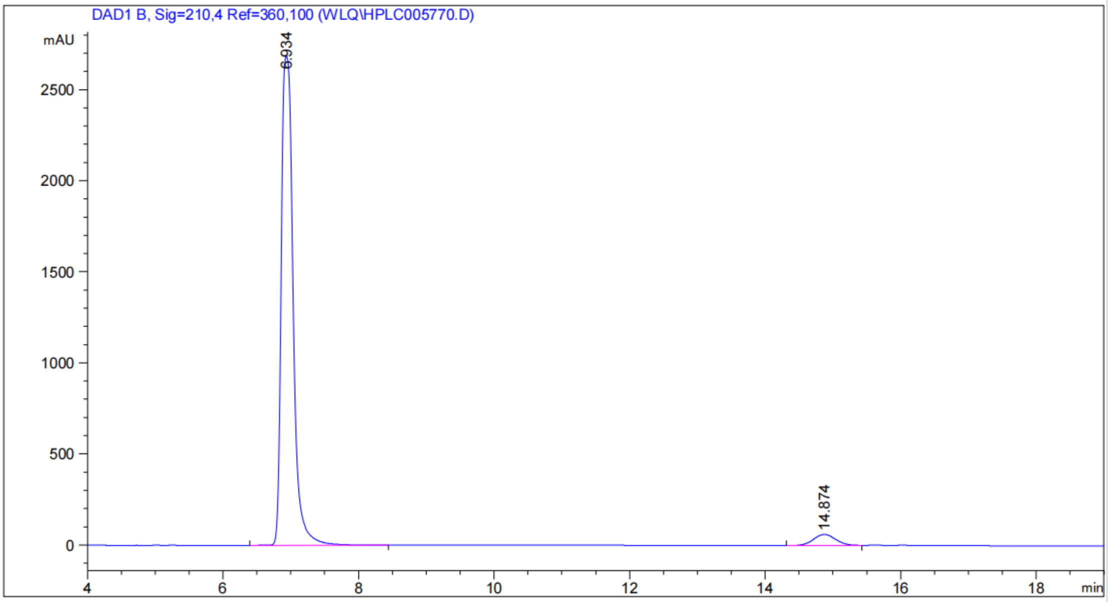

Peak #	Rt time [min]	Type	Width [min]	Peak Area [mAU*s]	Peak Height [mAU]	Peak Area %
1	6.934	BB	0.1926	3.28358e4	2686.03564	95.9825
2	14.874	BB	0.3564	1374.40796	60.18734	4.0175

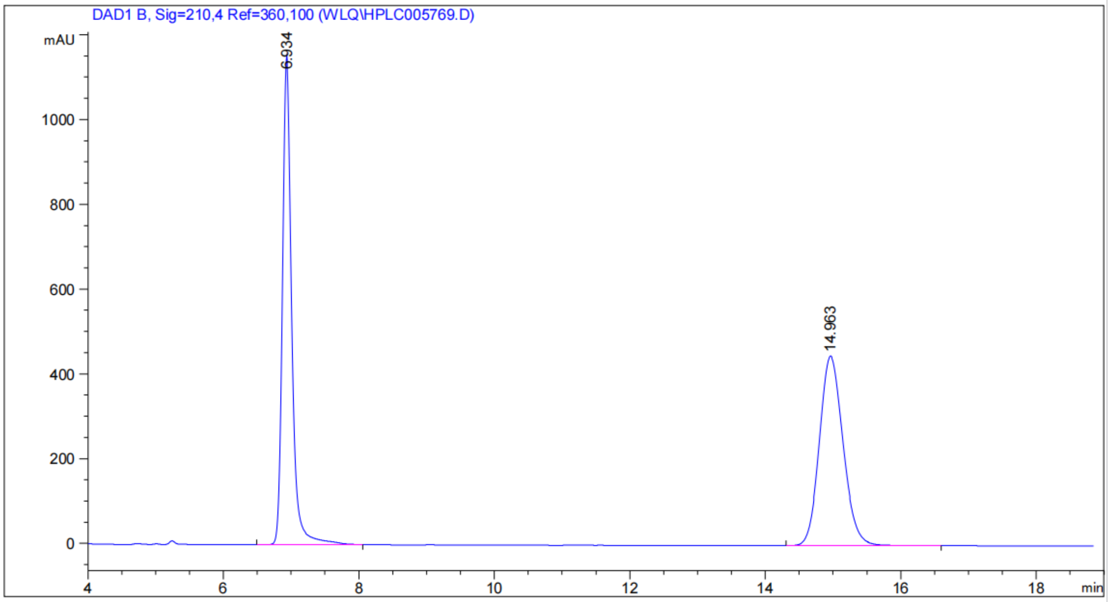

Peak #	Rt time [min]	Type	Width [min]	Peak Area [mAU*s]	Peak Height [mAU]	Peak Area %
1	6.934	BB	0.1466	1.11224e4	1152.38037	50.6209
2	14.963	BB	0.3751	1.08496e4	447.11707	49.3791

^{1}H NMR of diethyl (2*R*,5*S*)-3-(methylsulfonyl)-2-phenyl-5-(p-tolyl)oxazolidine-4,4-dicarboxylate (3bb) (400 MHz, $CDCl_3$)

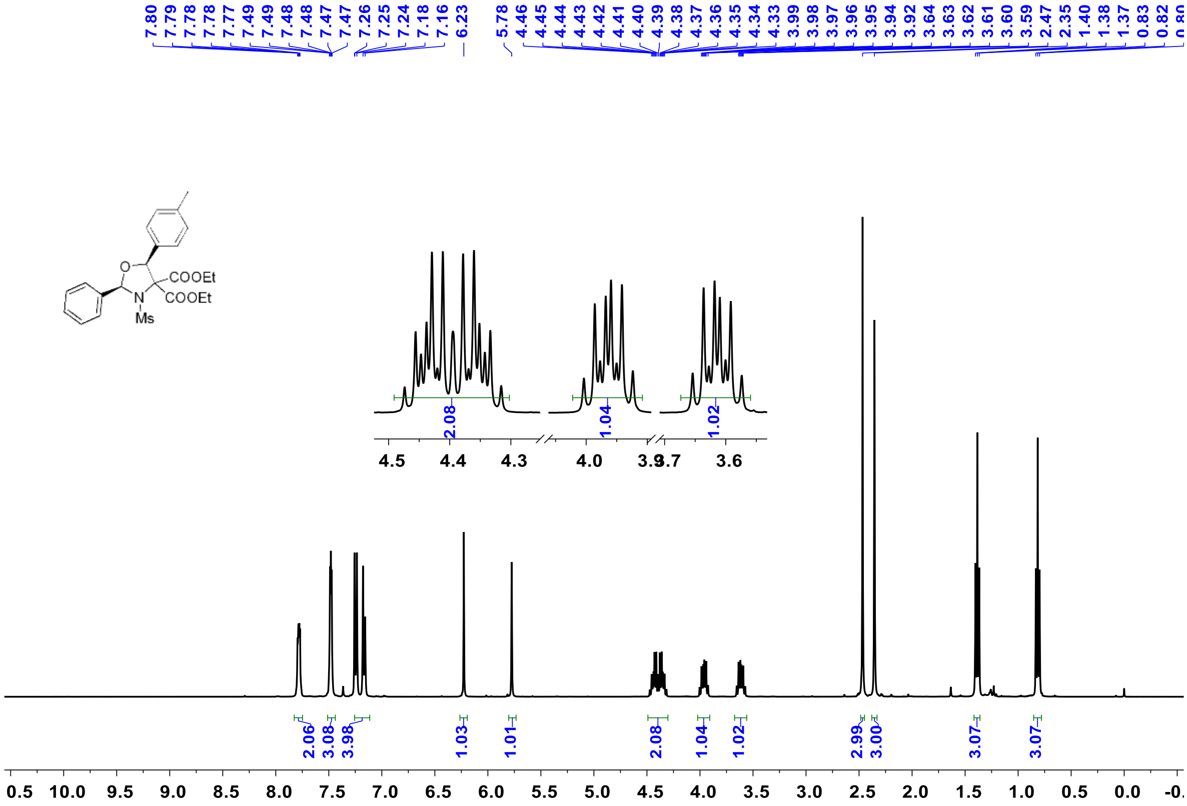

^{13}C{^{1}H} NMR of diethyl (2*R*,5*S*)-3-(methylsulfonyl)-2-phenyl-5-(p-tolyl)oxazolidine-4,4-dicarboxylate (3bb) (101 MHz, $CDCl_3$)

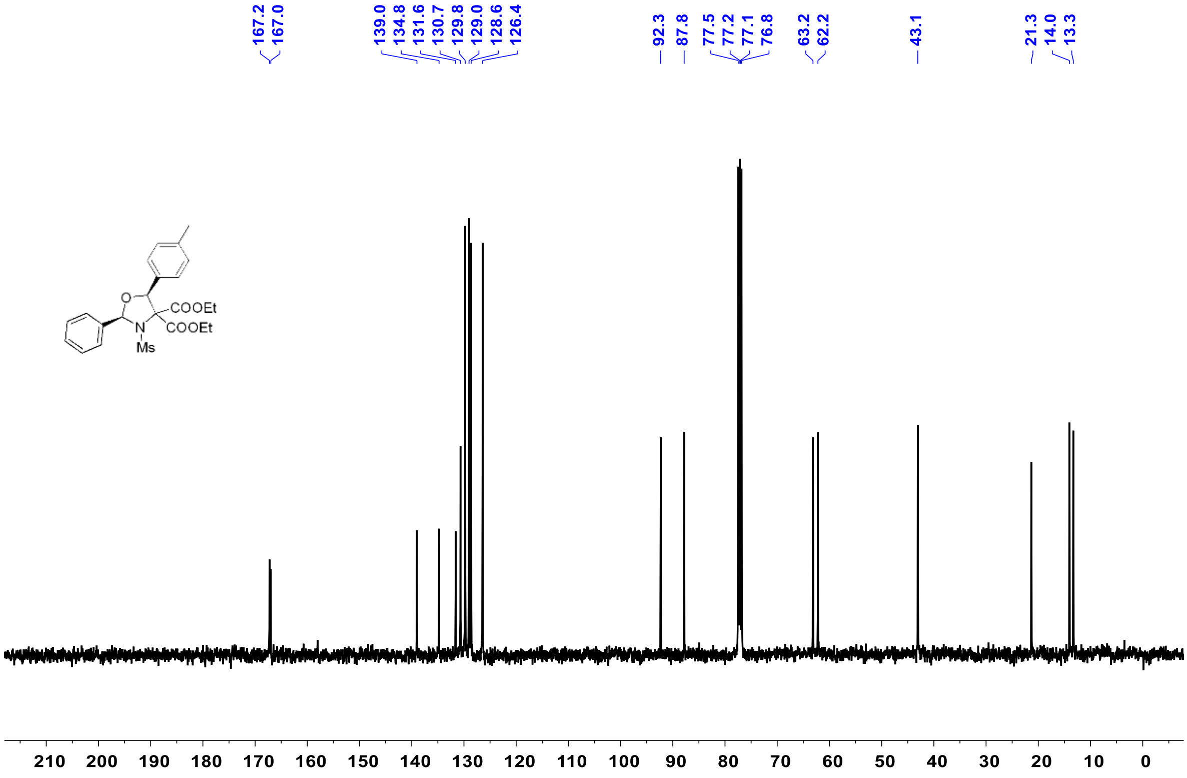

HPLC graph of racemic 3bb

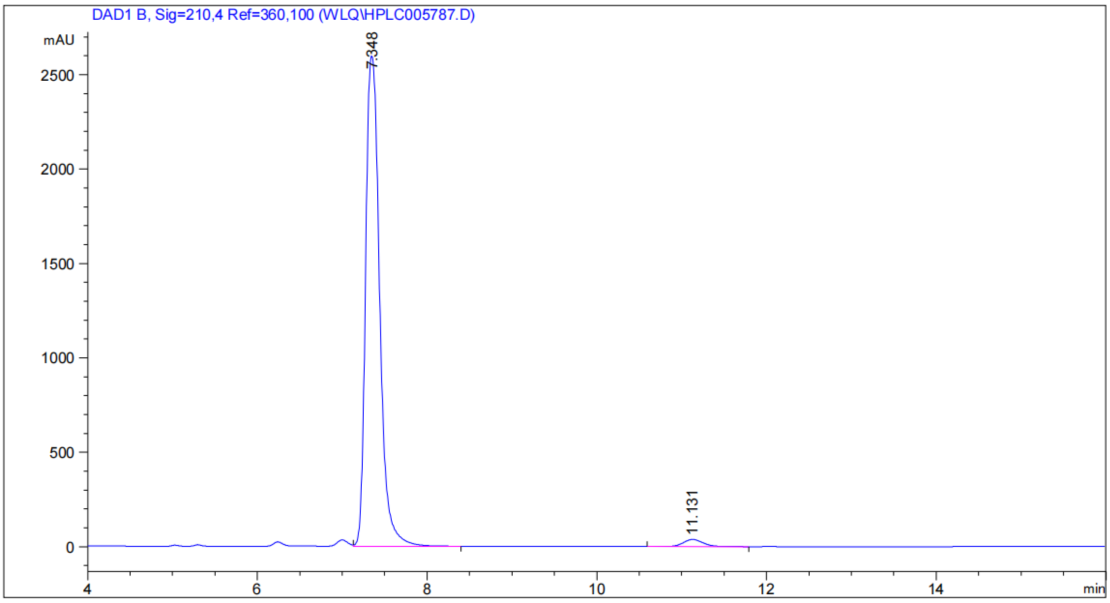

Peak #	Rt time [min]	Type	Width [min]	Peak Area [mAU*s]	Peak Height [mAU]	Peak Area %
1	7.348	VB	0.1799	2.97990e4	2595.73315	97.9656
2	11.131	BB	0.2502	618.83038	37.95324	2.0344

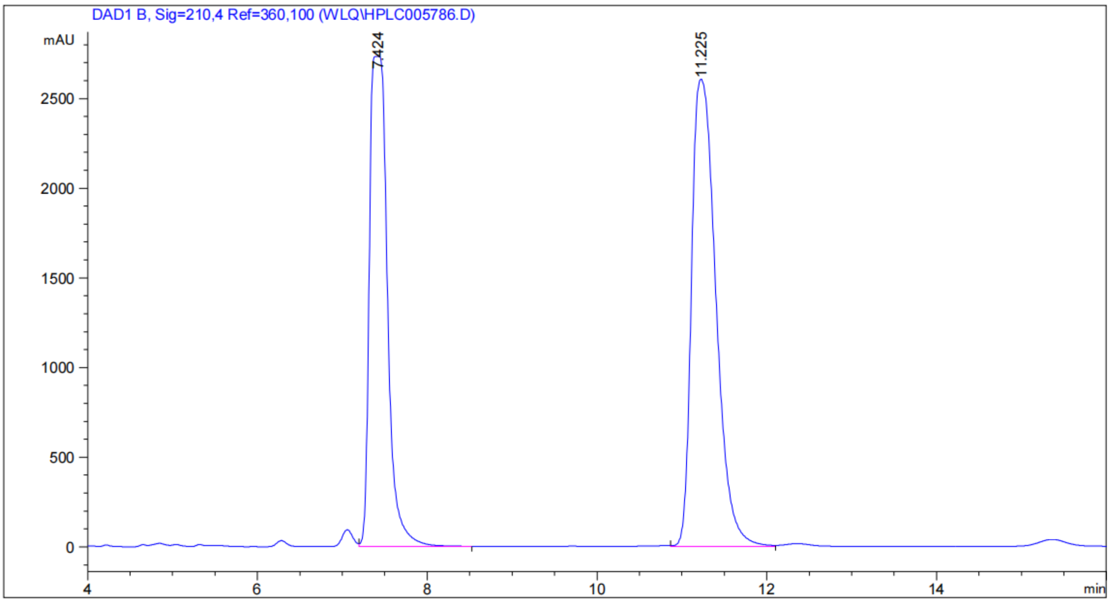

Peak #	Rt time [min]	Type	Width [min]	Peak Area [mAU*s]	Peak Height [mAU]	Peak Area %
1	7.424	VB	0.2012	4.01182e4	2734.49780	43.7015
2	11.225	VV	0.3113	5.16824e4	2606.35132	56.2985

HRMS (ESI) of diethyl (2*R*,5*S*)-3-(methylsulfonyl)-2-phenyl-5-(p-tolyl)oxazolidine-4,4-dicarboxylate (3bb)

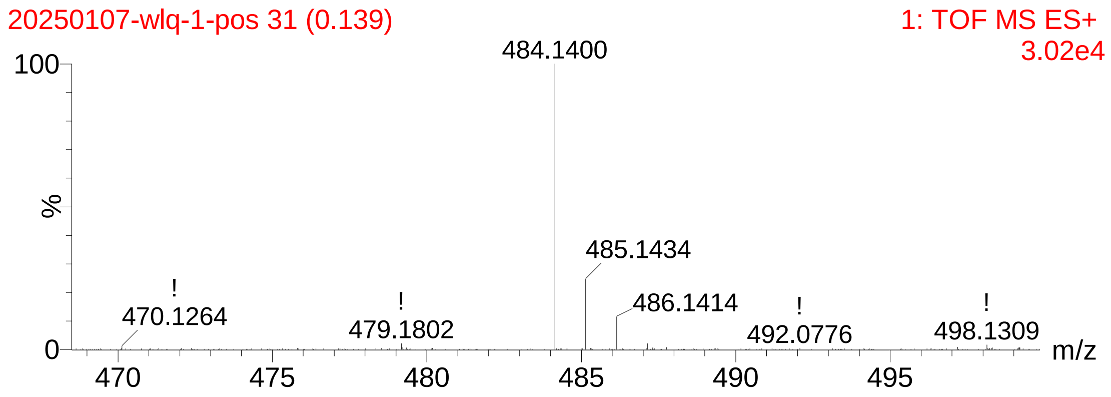

^{1}H NMR of diethyl (2*R*,5*S*)-5-(4-isopropylphenyl)-3-(methylsulfonyl)-2-phenyloxazolidine-4,4-dicarboxylate (3bc) (400 MHz, $CDCl_3$)

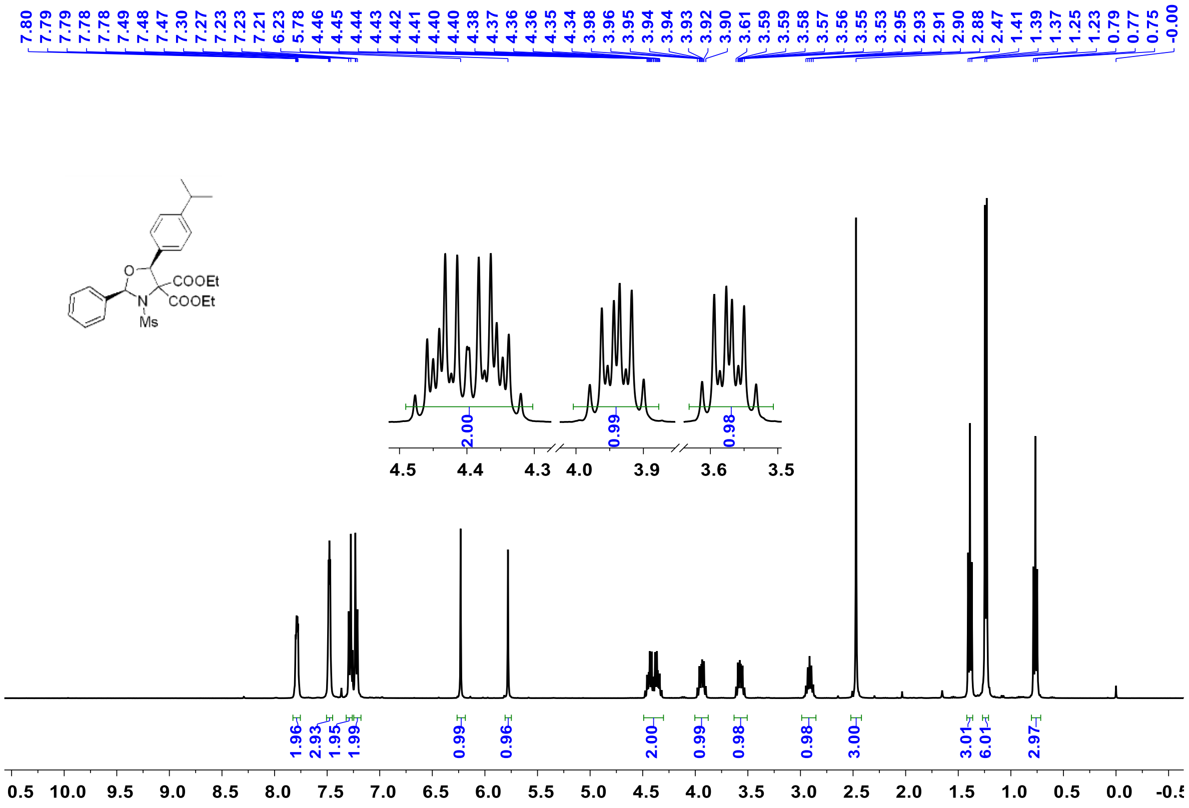

^{13}C{^{1}H} NMR of diethyl (2*R*,5*S*)-5-(4-isopropylphenyl)-3-(methylsulfonyl)-2-phenyloxazolidine-4,4-dicarboxylate (3bc) (101 MHz, $CDCl_3$)

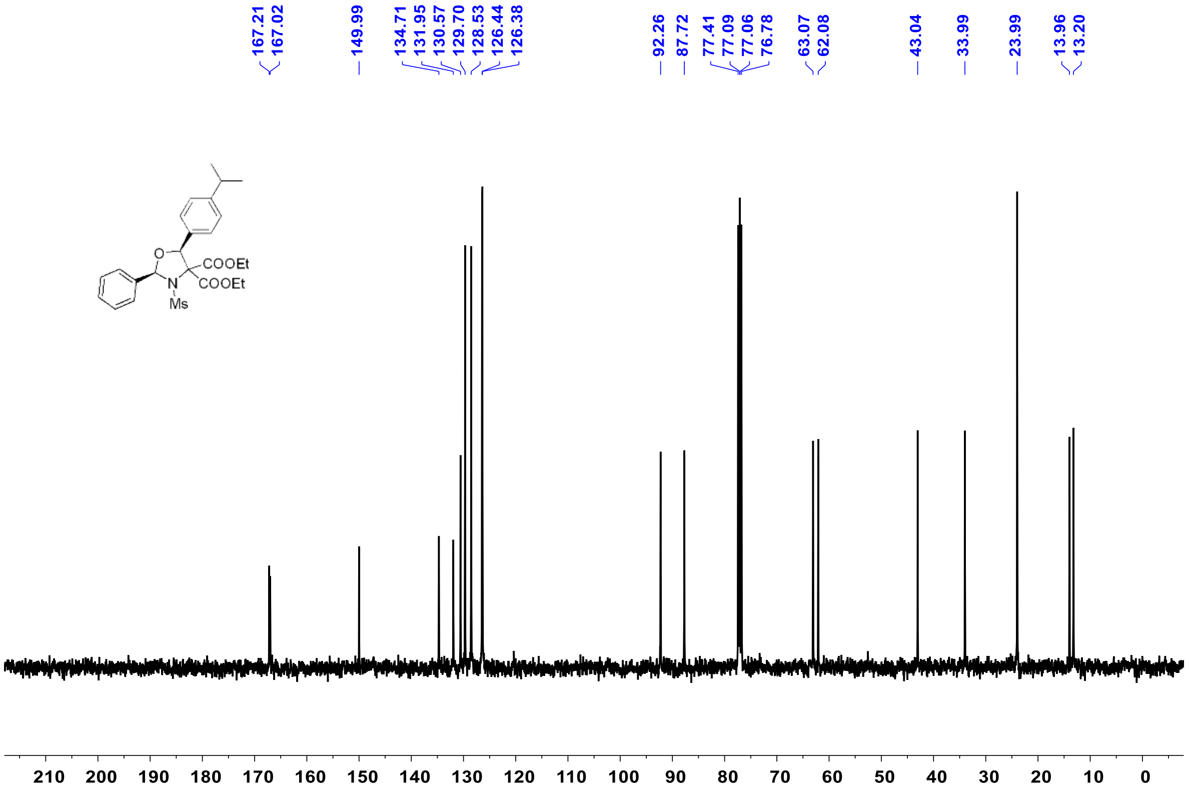

HPLC graph of racemic 3bc

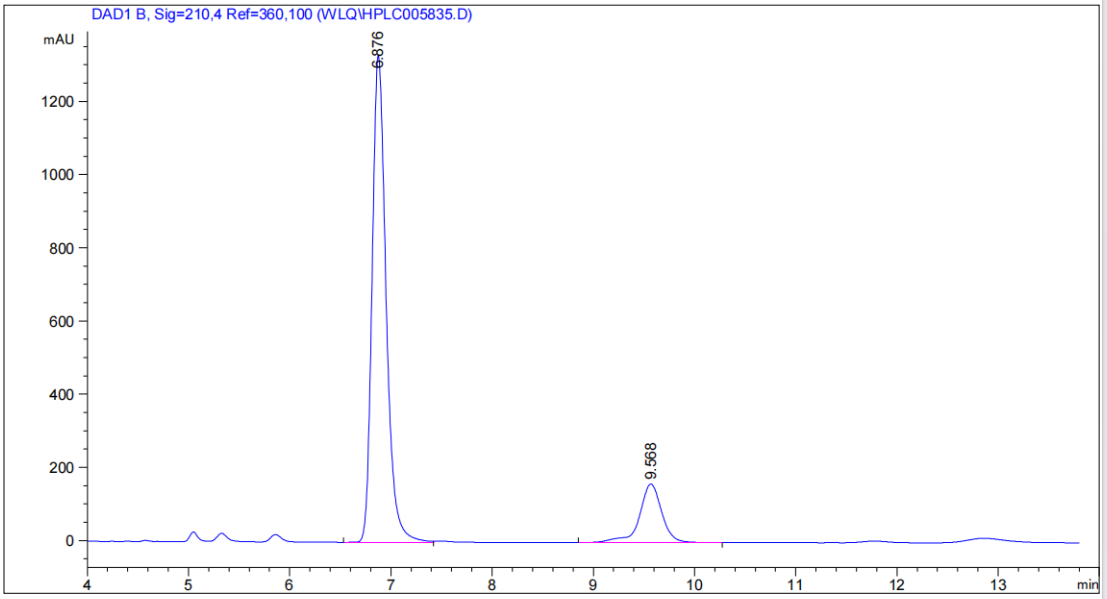

Peak #	Rt time [min]	Type	Width [min]	Peak Area [mAU*s]	Peak Height [mAU]	Peak Area %
1	6.876	BV	0.1480	1.27814e4	1331.25537	83.9152
2	9.568	BB	0.2307	2449.92944	159.96590	16.0848

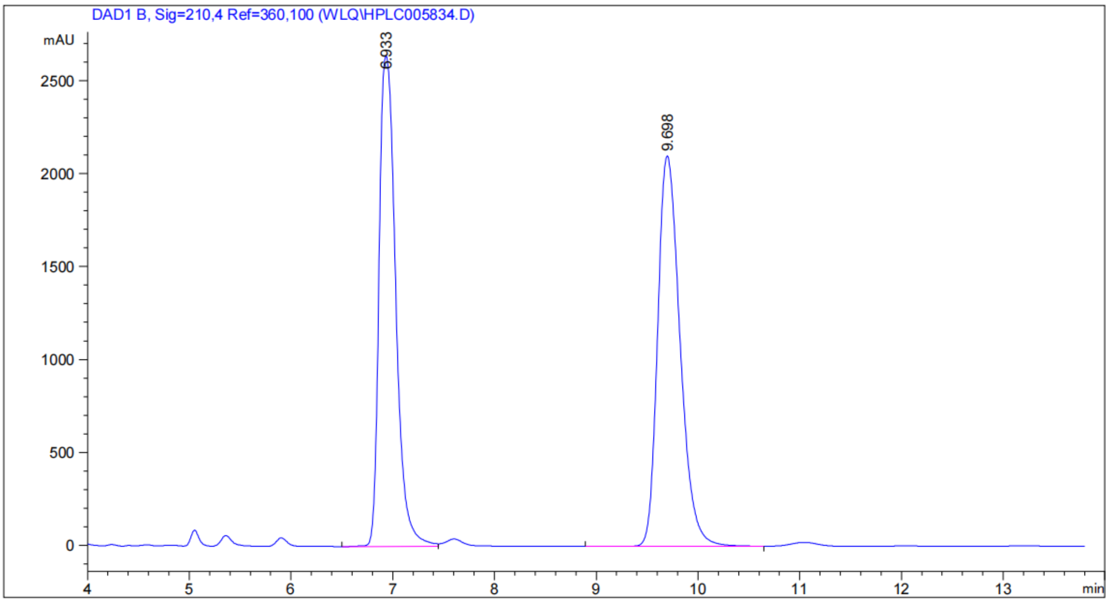

Peak #	Rt time [min]	Type	Width [min]	Peak Area [mAU*s]	Peak Height [mAU]	Peak Area %
1	6.933	BV	0.1783	2.99070e4	2636.85620	48.2650
2	9.698	BB	0.2382	3.20572e4	2099.03491	51.7350

HRMS (ESI) of diethyl (2*R*,5*S*)-5-(4-isopropylphenyl)-3-(methylsulfonyl)-2-phenyloxazolidine-4,4-dicarboxylate (3bc)

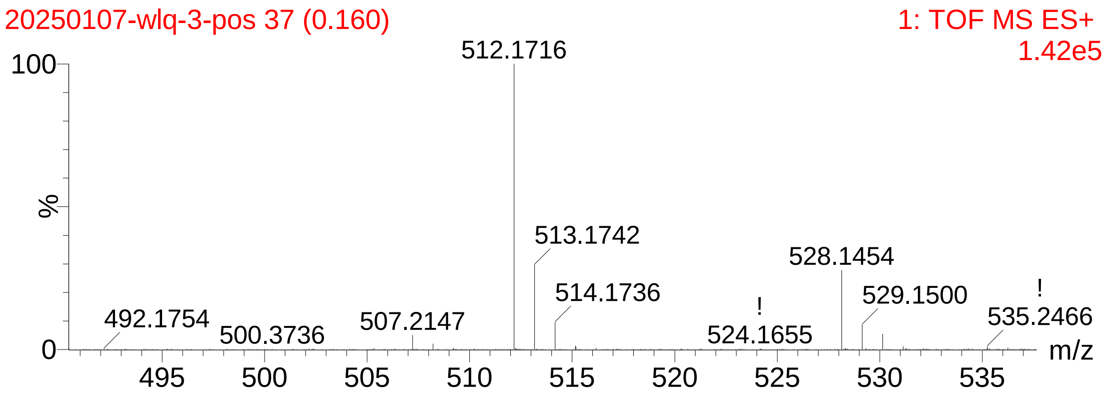

^{1}H NMR of diethyl (2*R*,5*S*)-5-(4-methoxyphenyl)-3-(methylsulfonyl)-2-phenyloxazolidine-4,4-dicarboxylate (3bd) (400 MHz, $CDCl_3$)

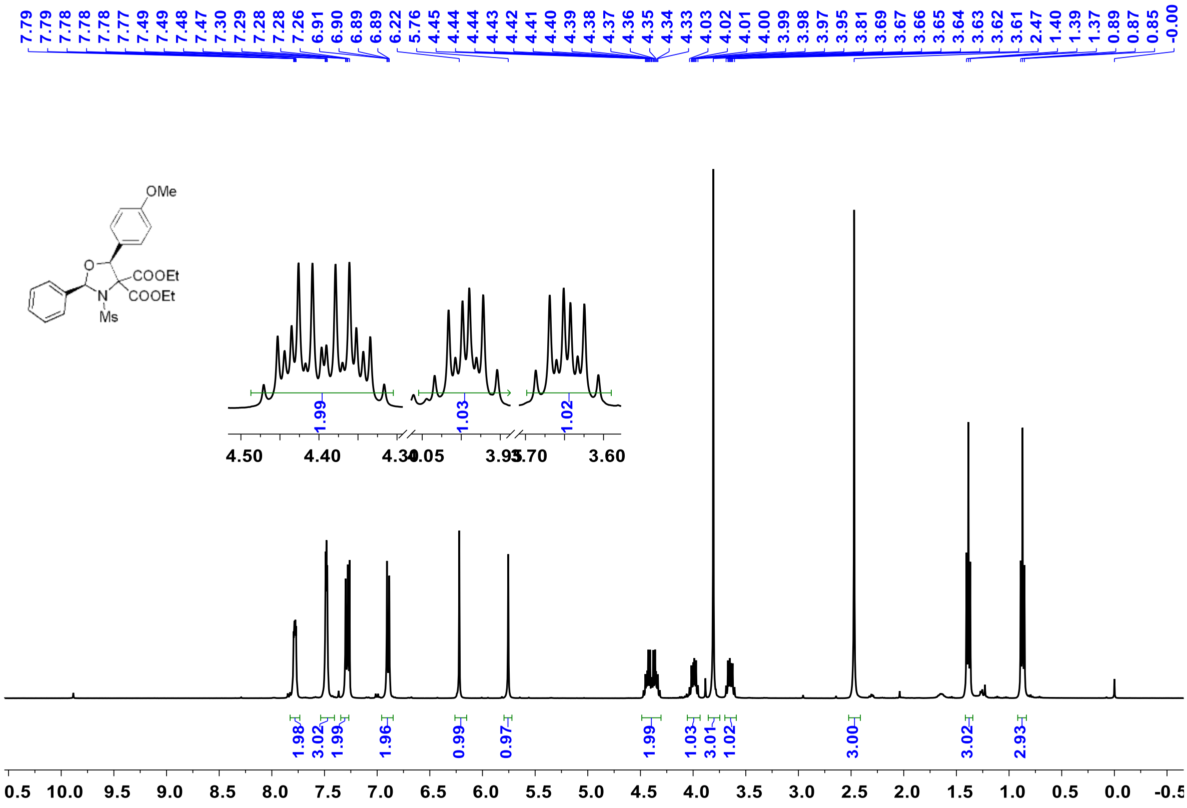

^{13}C{^{1}H} NMR of Diethyl (2*R*,5*S*)-5-(4-methoxyphenyl)-3-(methylsulfonyl)-2-phenyloxazolidine-4,4-dicarboxylate (3bd) (101 MHz, $CDCl_3$)

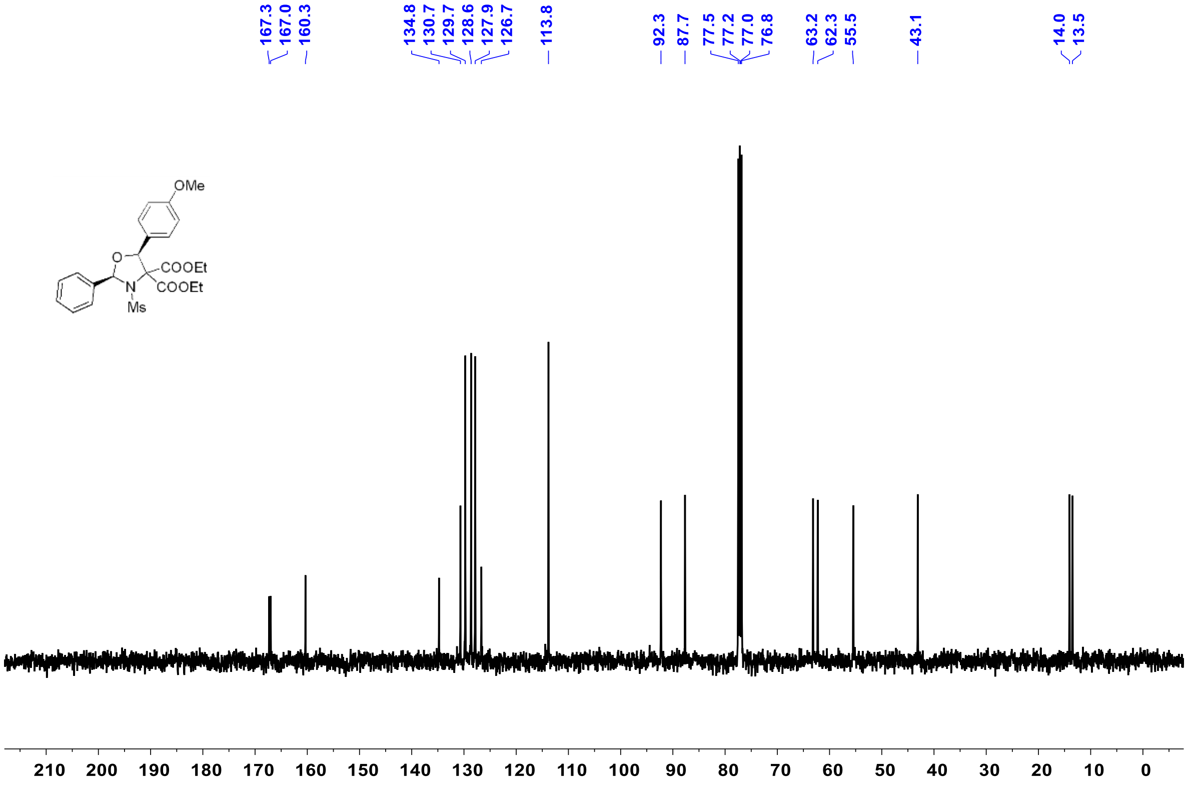

HPLC graph of racemic 3bd

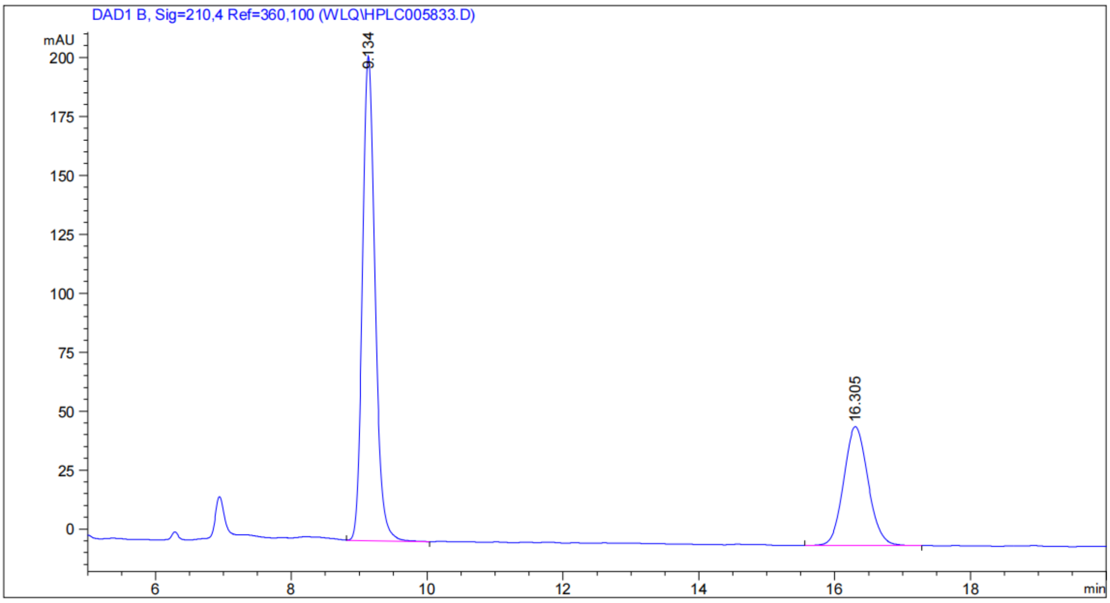

Peak #	Rt time [min]	Type	Width [min]	Peak Area [mAU*s]	Peak Height [mAU]	Peak Area %
1	9.134	BB	0.1956	2634.00415	205.36174	67.6562
2	16.305	BB	0.3873	1259.21606	50.42622	32.3438

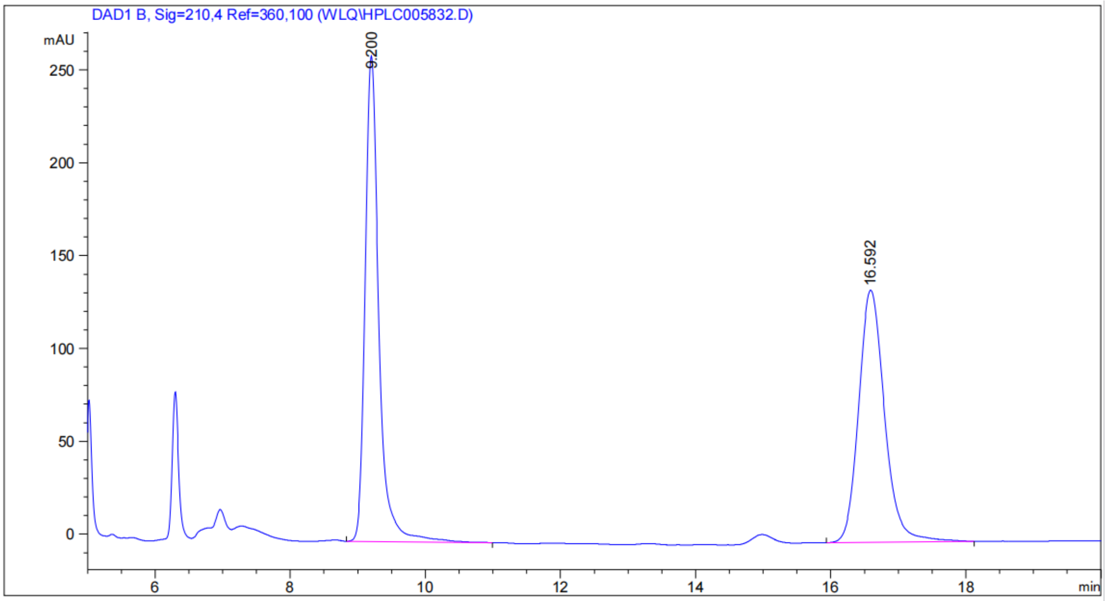

Peak #	Rt time [min]	Type	Width [min]	Peak Area [mAU*s]	Peak Height [mAU]	Peak Area %
1	9.200	BB	0.2127	3690.45679	261.37576	50.5341
2	16.592	BB	0.4084	3612.44214	135.77563	49.4659

HRMS (ESI) of diethyl (2*R*,5*S*)-5-(4-methoxyphenyl)-3-(methylsulfonyl)-2-phenyloxazolidine-4,4-dicarboxylate (3bd)

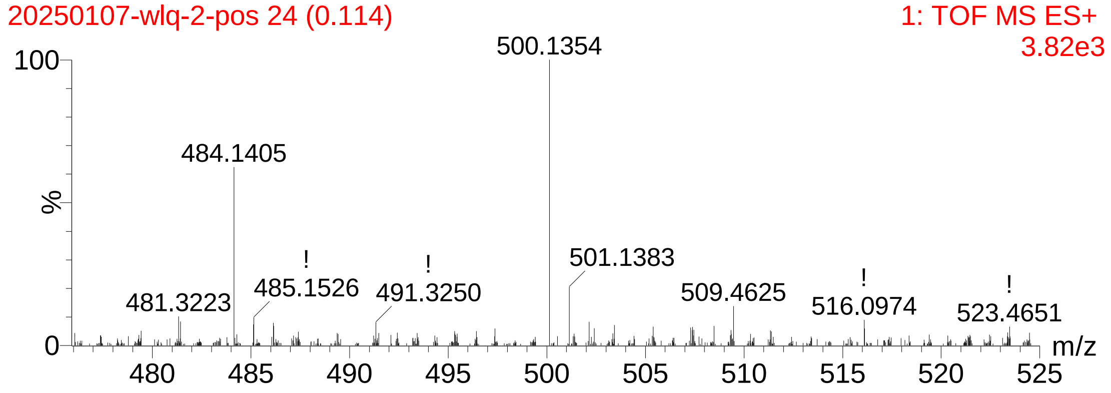

^{1}H NMR of diethyl (2*R*,5*S*)-5-(4-bromophenyl)-3-(methylsulfonyl)-2-phenyloxazolidine-4,4-dicarboxylate (3bf) (400 MHz, $CDCl_3$)

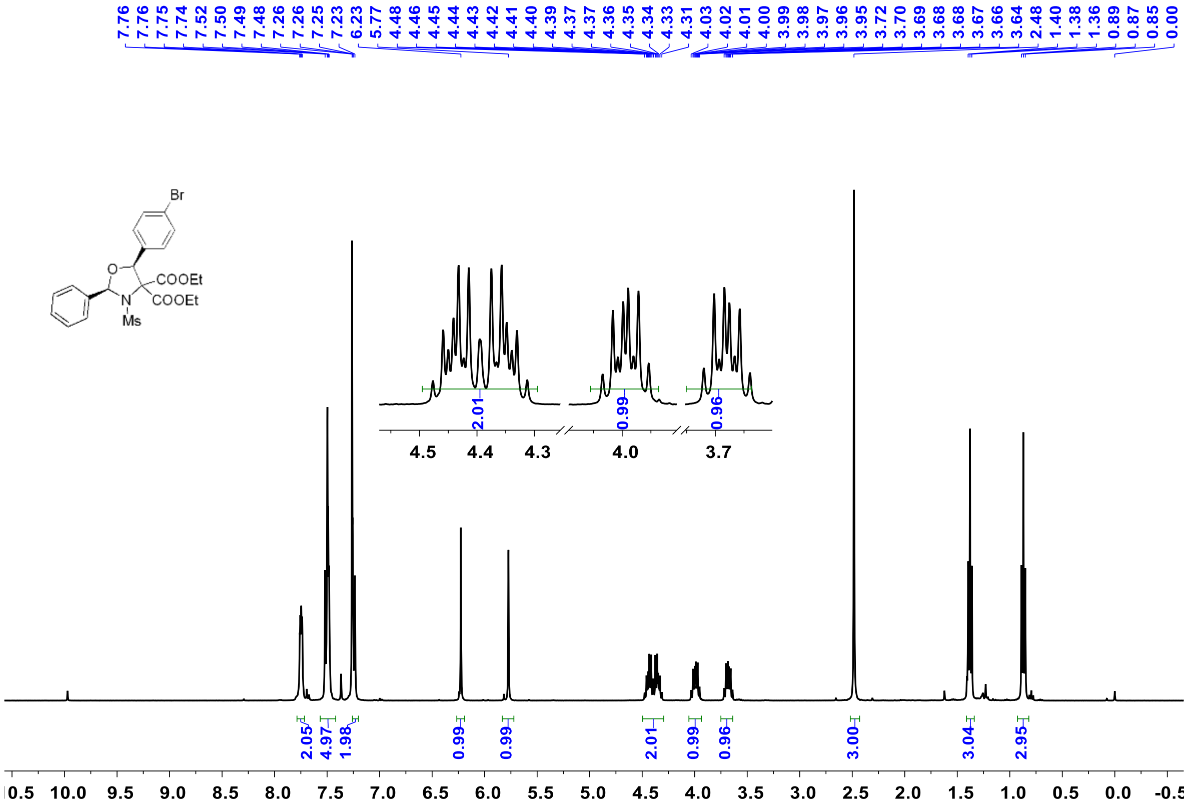

^{13}C{^{1}H} NMR of diethyl (2*R*,5*S*)-5-(4-bromophenyl)-3-(methylsulfonyl)-2-phenyloxazolidine-4,4-dicarboxylate (3bf) (101 MHz, $CDCl_3$)

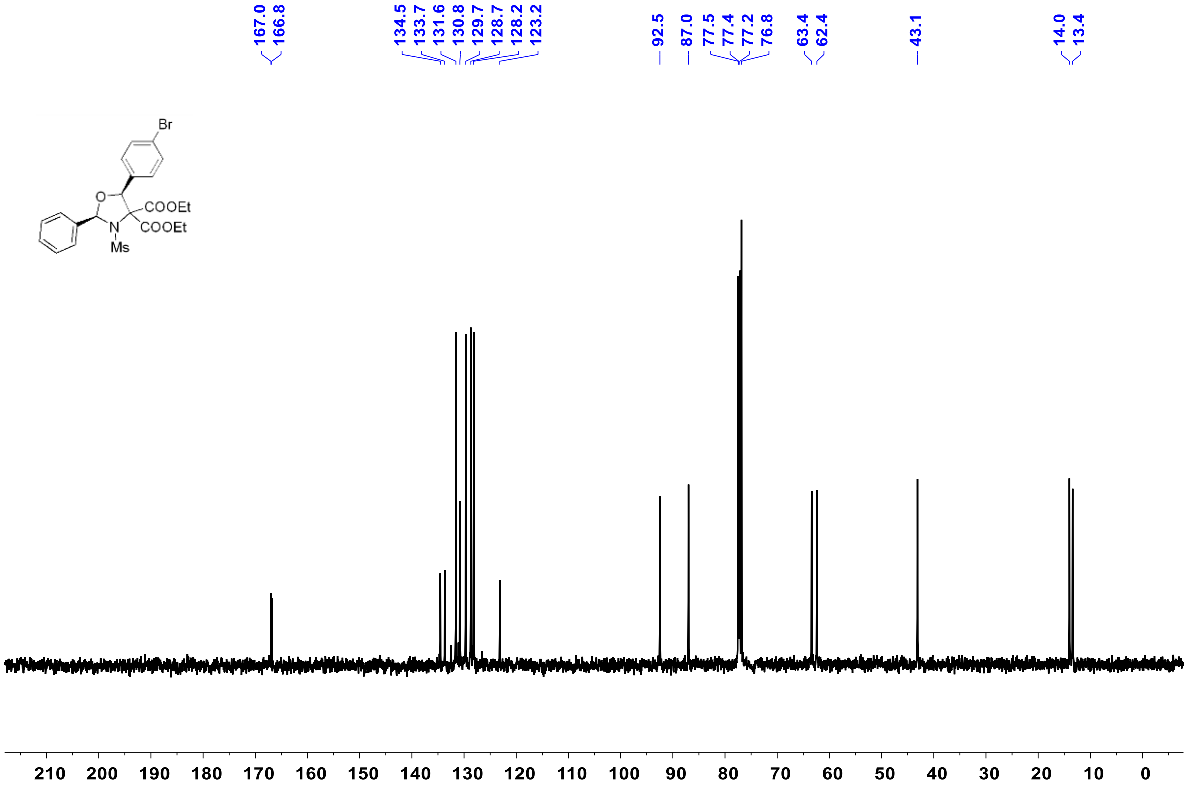

HPLC graph of racemic 3bf

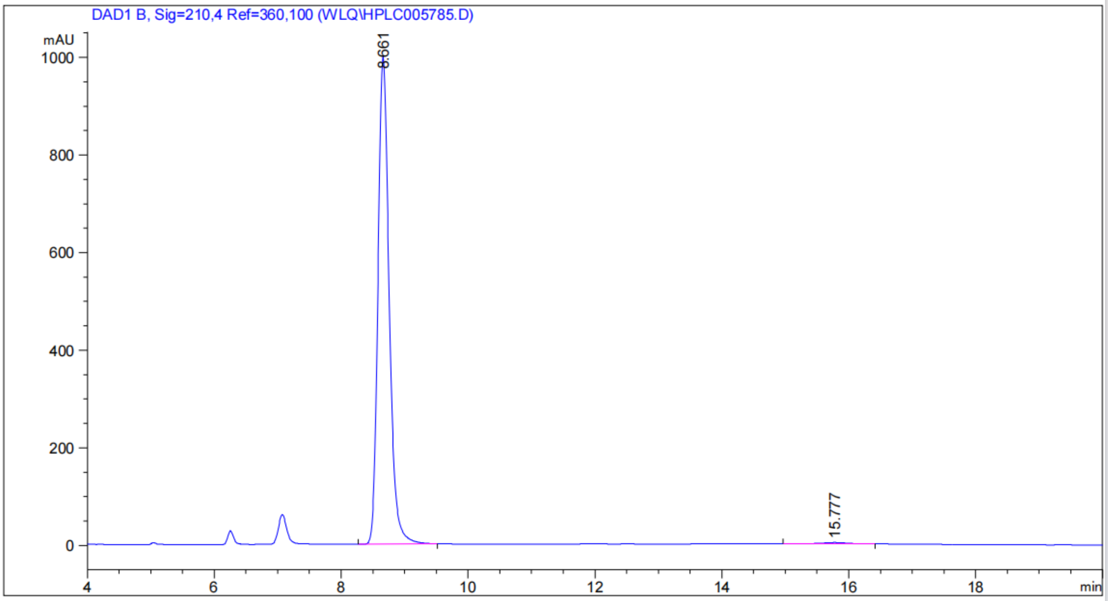

Peak #	Rt time [min]	Type	Width [min]	Peak Area [mAU*s]	Peak Height [mAU]	Peak Area %
1	8.661	BB	0.1886	1.22246e4	1000.07172	99.3450
2	15.777	BB	0.4288	80.60319	2.72711	0.6550

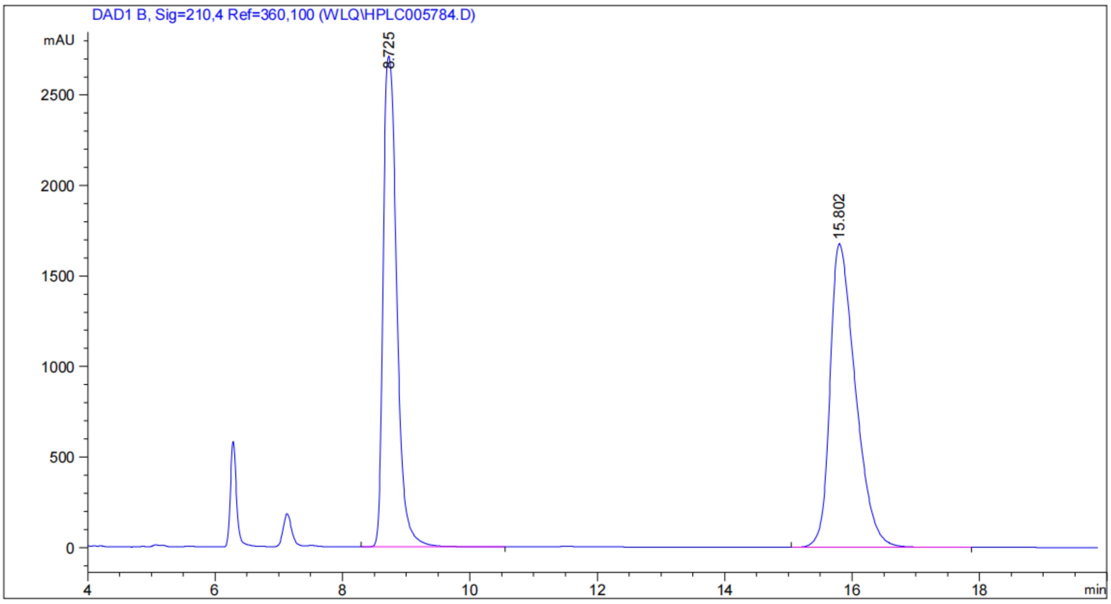

Peak #	Rt time [min]	Type	Width [min]	Peak Area [mAU*s]	Peak Height [mAU]	Peak Area %
1	8.725	BB	0.2312	4.02433e4	2710.25366	46.4211
2	15.802	BB	0.4211	4.64485e4	1677.05078	53.5789

HRMS (ESI) of diethyl (2*R*,5*S*)-5-(4-bromophenyl)-3-(methylsulfonyl)-2-phenyloxazolidine-4,4-dicarboxylate (3bf)

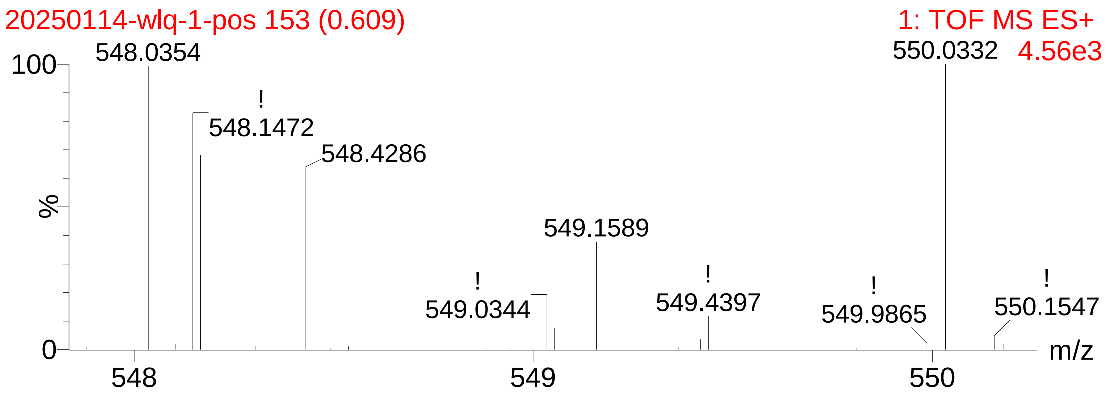

^{1}H NMR of diethyl (2*R*,5*S*)-3-(methylsulfonyl)-2-phenyl-5-(3,4,5-trimethoxyphenyl)oxazolidine-4,4-dicarboxylate (3bi) (400 MHz, CDCl3)

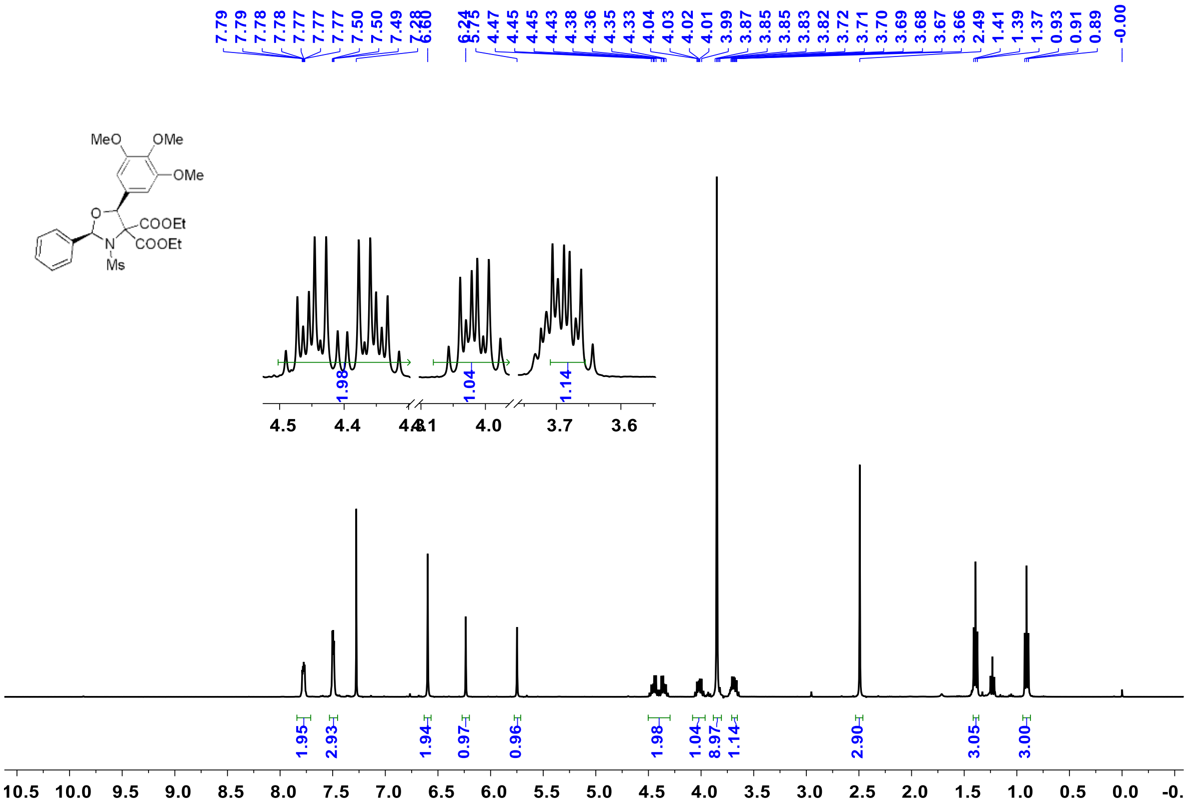

^{13}C{^{1}H} NMR of Diethyl (2*R*,5*S*)-3-(methylsulfonyl)-2-phenyl-5-(3,4,5-trimethoxyphenyl)oxazolidine-4,4-dicarboxylate (3bi) (101 MHz, CDCl3)

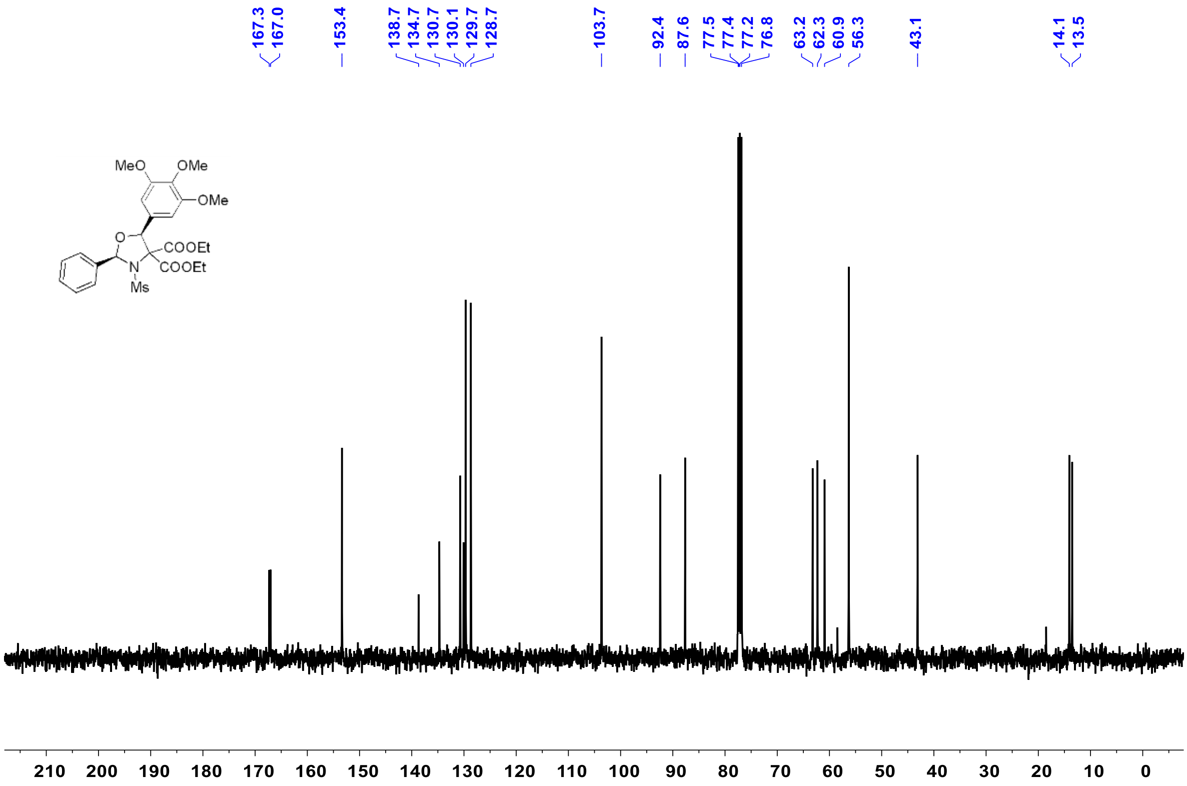

HPLC graph of racemic 3bi

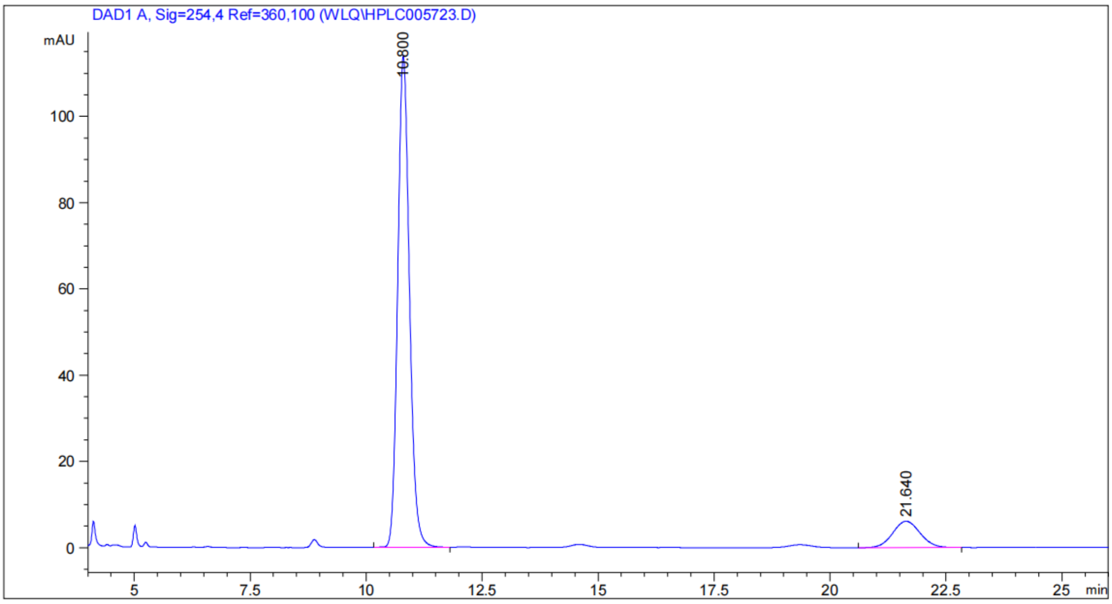

Peak #	Rt time [min]	Type	Width [min]	Peak Area [mAU*s]	Peak Height [mAU]	Peak Area %
1	10.800	BB	0.2702	1992.61841	113.83643	88.8077
2	21.640	BB	0.6246	251.12764	6.12117	11.1923

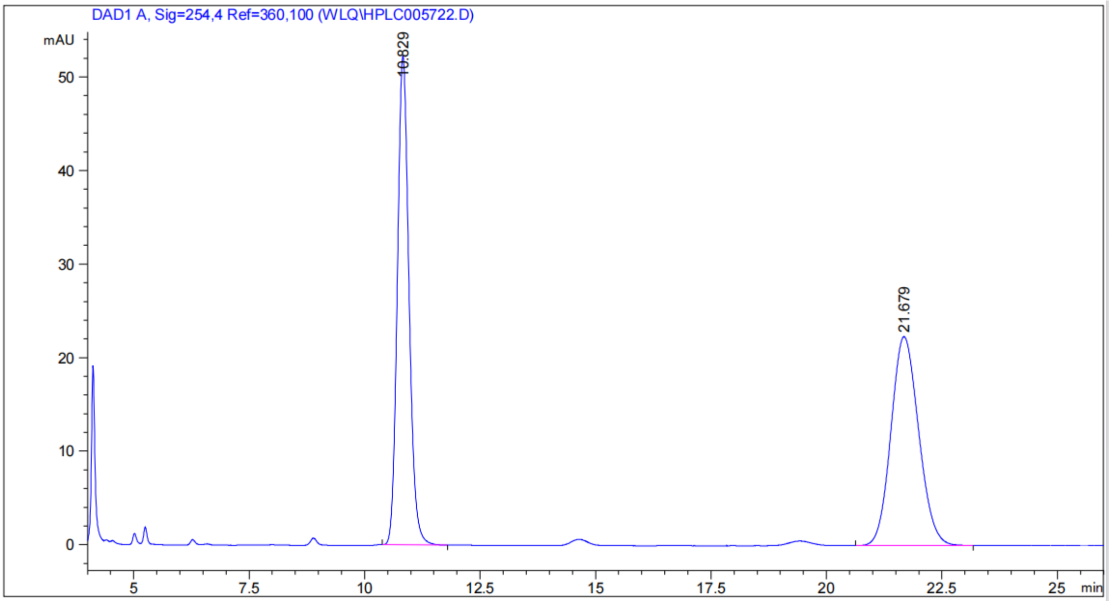

Peak #	Rt time [min]	Type	Width [min]	Peak Area [mAU*s]	Peak Height [mAU]	Peak Area %
1	10.829	BB	0.2691	909.26288	52.22946	49.4701
2	21.679	BB	0.6472	928.74323	22.33172	50.5299

HRMS (ESI) of diethyl (2*R*,5*S*)-3-(methylsulfonyl)-2-phenyl-5-(3,4,5-trimethoxyphenyl)oxazolidine-4,4-dicarboxylate (3bi)

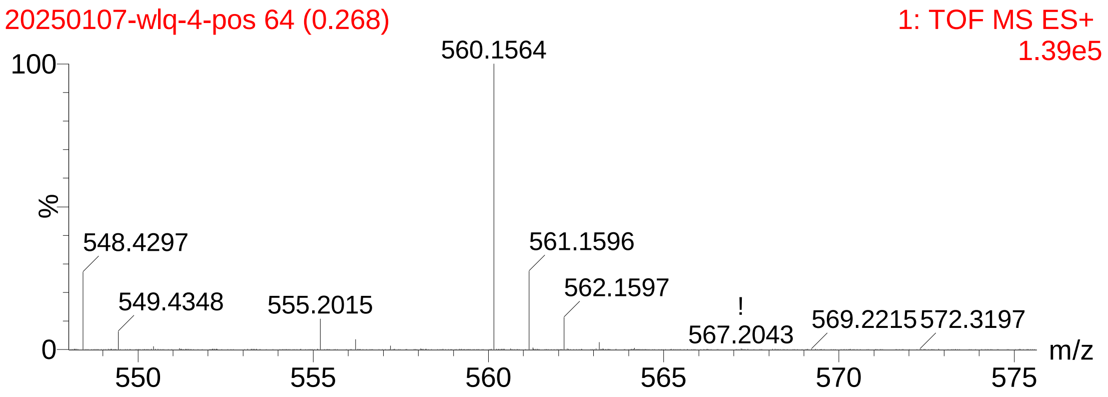

^{1}H NMR of diethyl (2*R*,5*S*)-3-(methylsulfonyl)-2-phenyl-5-(m-tolyl)oxazolidine-4,4-dicarboxylate (3bk) (400 MHz, $CDCl_3$)

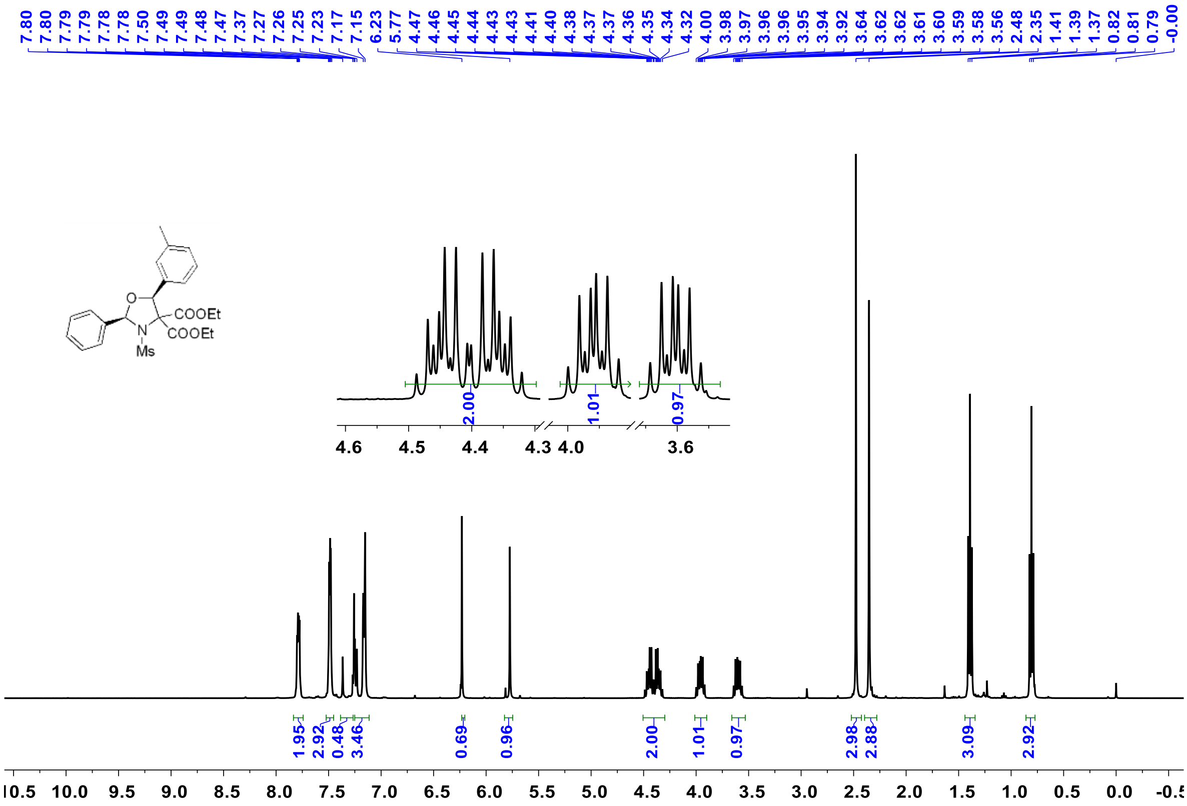

^{13}C{^{1}H} NMR of Diethyl (2*R*,5*S*)-3-(methylsulfonyl)-2-phenyl-5-(m-tolyl)oxazolidine-4,4-dicarboxylate (3bk) (101 MHz, $CDCl_3$)

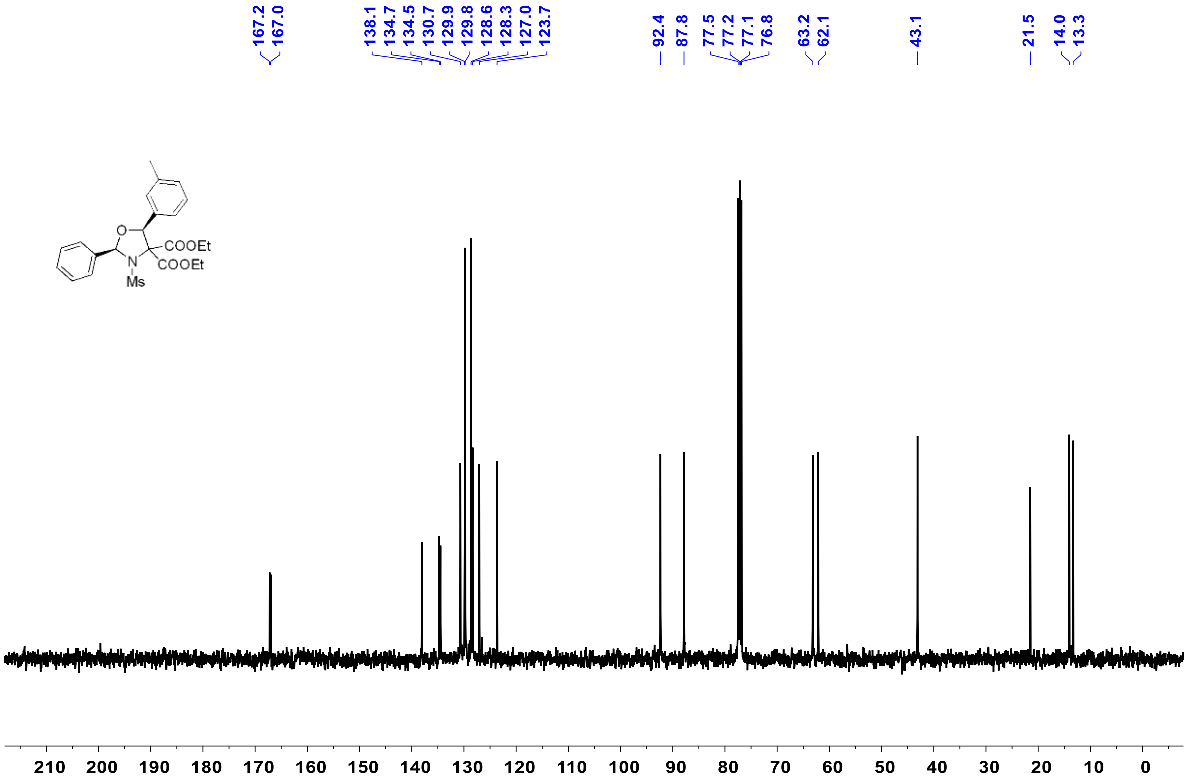

HPLC graph of racemic 3bk

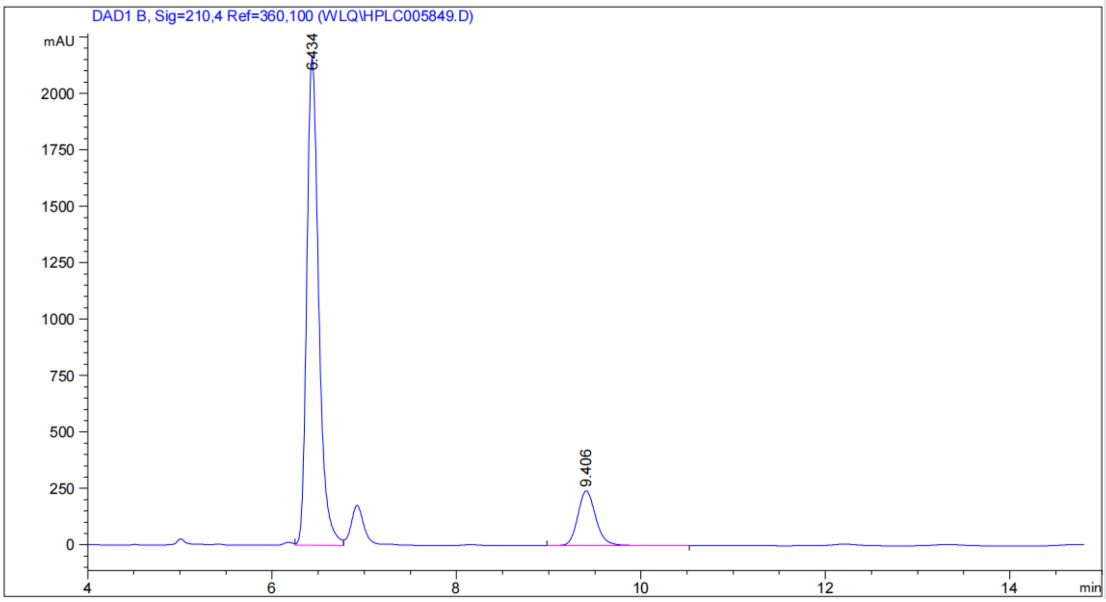

Peak #	Rt time [min]	Type	Width [min]	Peak Area [mAU*s]	Peak Height [mAU]	Peak Area %
1	6.434	VV	0.1397	1.95819e4	2161.31567	85.6851
2	9.406	BB	0.2078	3271.42700	241.81616	14.3149

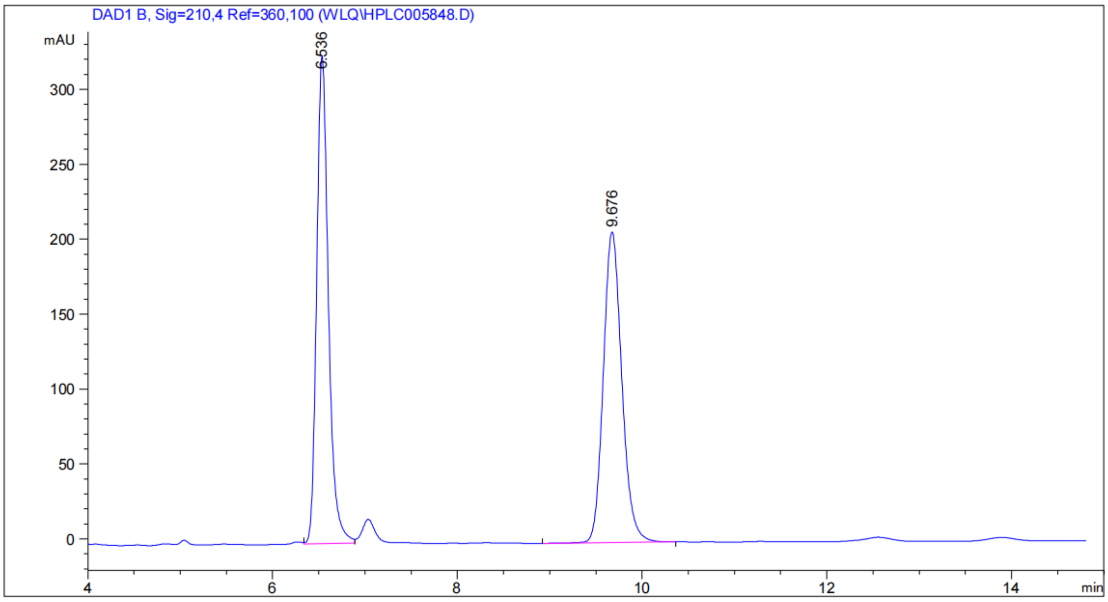

Peak #	Rt time [min]	Type	Width [min]	Peak Area [mAU*s]	Peak Height [mAU]	Peak Area %
1	6.536	VV	0.1348	2871.47070	325.77151	49.9299
2	9.676	BB	0.2140	2879.53418	207.38139	50.0701

HRMS (ESI) of diethyl (2*R*,5*S*)-3-(methylsulfonyl)-2-phenyl-5-(m-tolyl)oxazolidine-4,4-dicarboxylate (3bk)

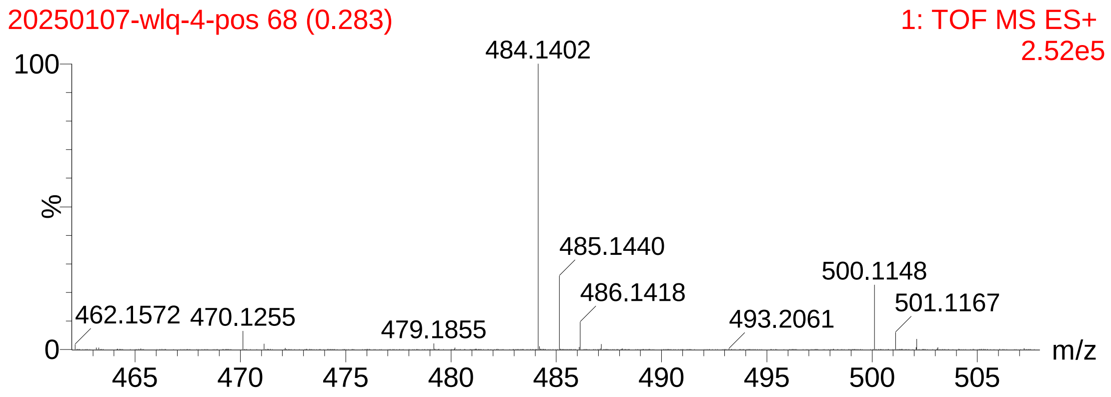

^{1}H NMR of diethyl (2*R*,5*S*)-3-(methylsulfonyl)-5-(4-nitrophenyl)-2-phenyloxazolidine-4,4-dicarboxylate (3bl) (400 MHz, $CDCl_3$)

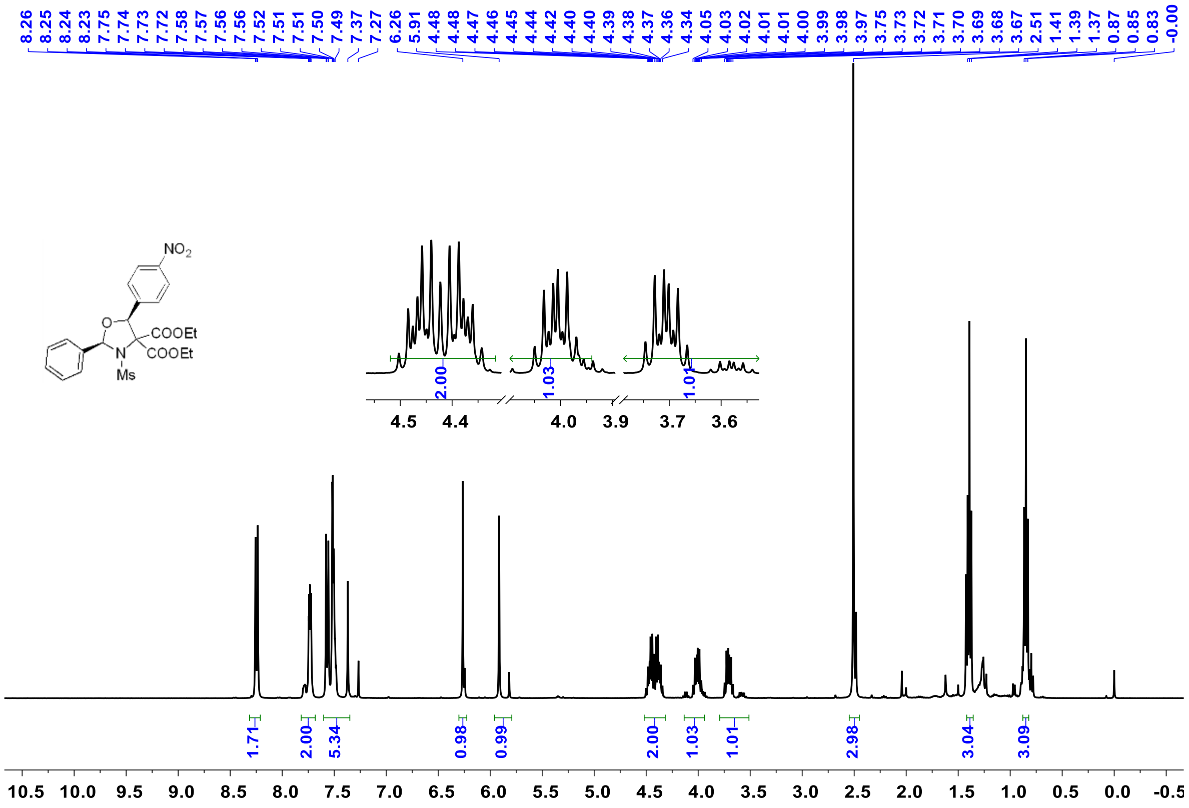

^{13}C{^{1}H} NMR of diethyl (2*R*,5*S*)-3-(methylsulfonyl)-5-(4-nitrophenyl)-2-phenyloxazolidine-4,4-dicarboxylate (3bl) (101 MHz, $CDCl_3$)

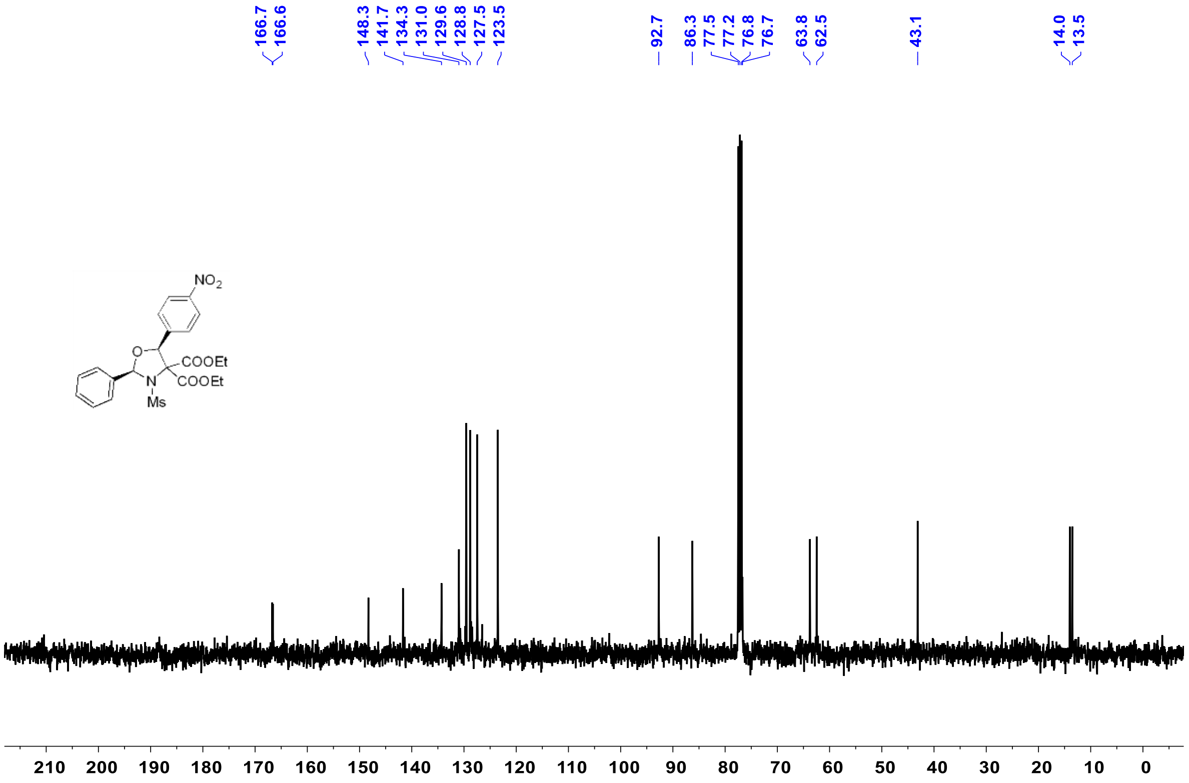

HPLC graph of racemic 3bl

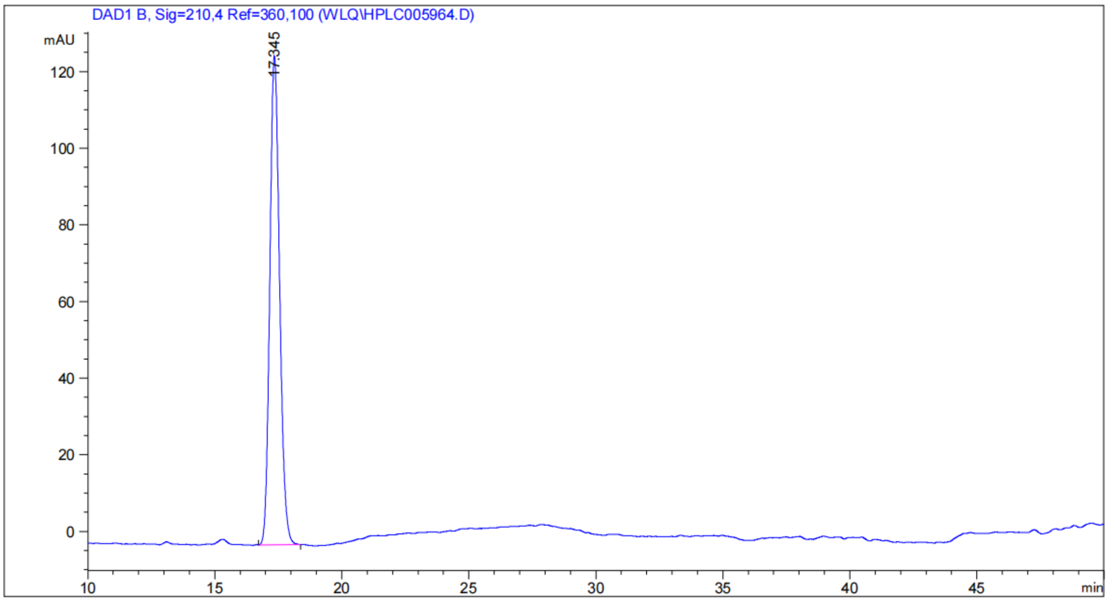

Peak #	Rt time [min]	Type	Width [min]	Peak Area [mAU*s]	Peak Height [mAU]	Peak Area %
1	17.345	BB	0.4133	3402.02734	127.51382	100.0000

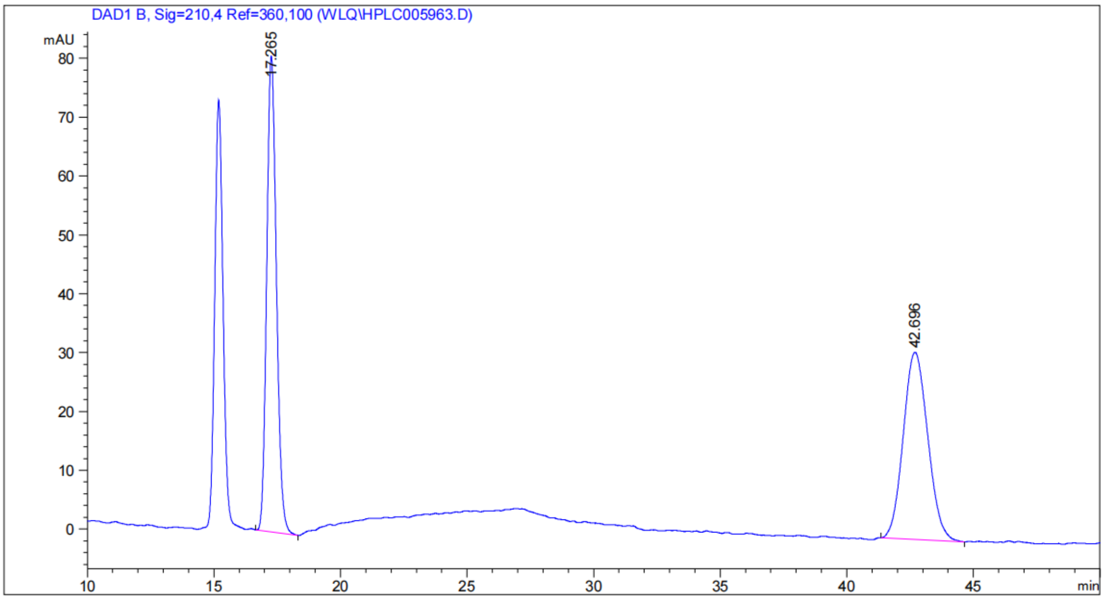

Peak #	Rt time [min]	Type	Width [min]	Peak Area [mAU*s]	Peak Height [mAU]	Peak Area %
1	17.265	BB	0.4089	2141.34204	80.88268	49.9958
2	42.696	BB	1.0215	2141.70142	31.80822	50.0042

HRMS (ESI) of diethyl (2*R*,5*S*)-3-(methylsulfonyl)-5-(4-nitrophenyl)-2-phenyloxazolidine-4,4-dicarboxylate (3bl)

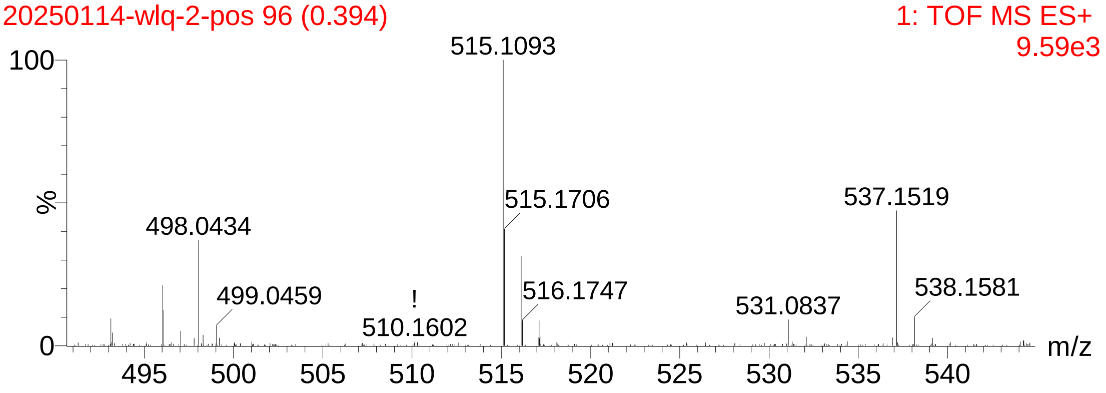

^{1}H NMR of diethyl (2*R*,5*S*)-3-(methylsulfonyl)-5-(naphthalen-1-yl)-2-phenyloxazolidine-4,4-dicarboxylate (3bm) (400 MHz, $CDCl_3$)

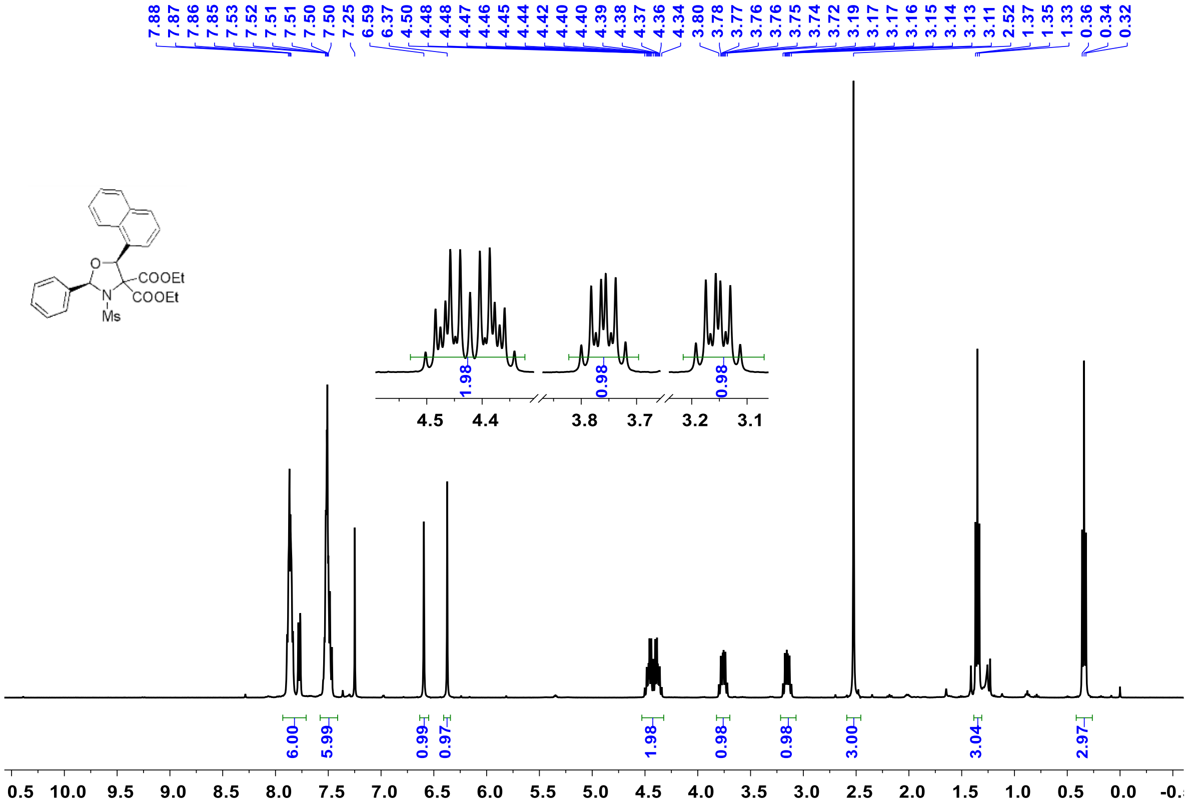

^{13}C{^{1}H} NMR of diethyl (2*R*,5*S*)-3-(methylsulfonyl)-5-(naphthalen-1-yl)-2-phenyloxazolidine-4,4-dicarboxylate (3bm) (101 MHz, $CDCl_3$)

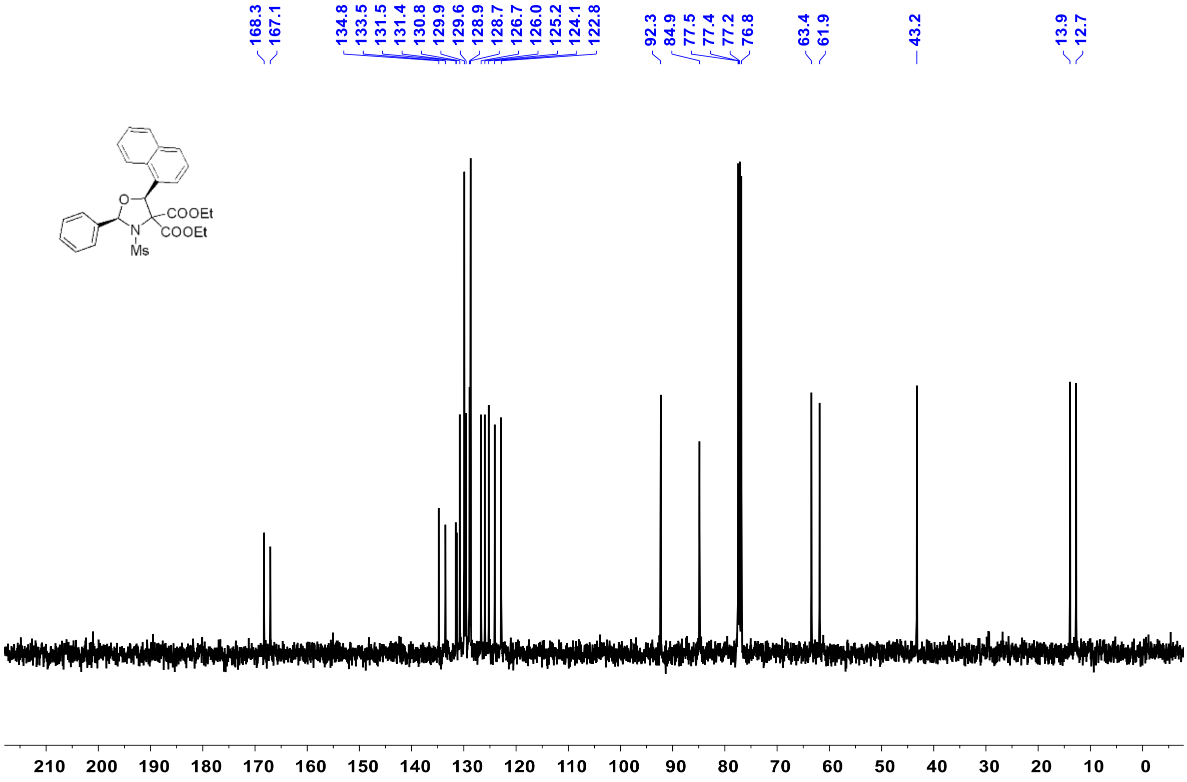

HPLC graph of racemic 3bm

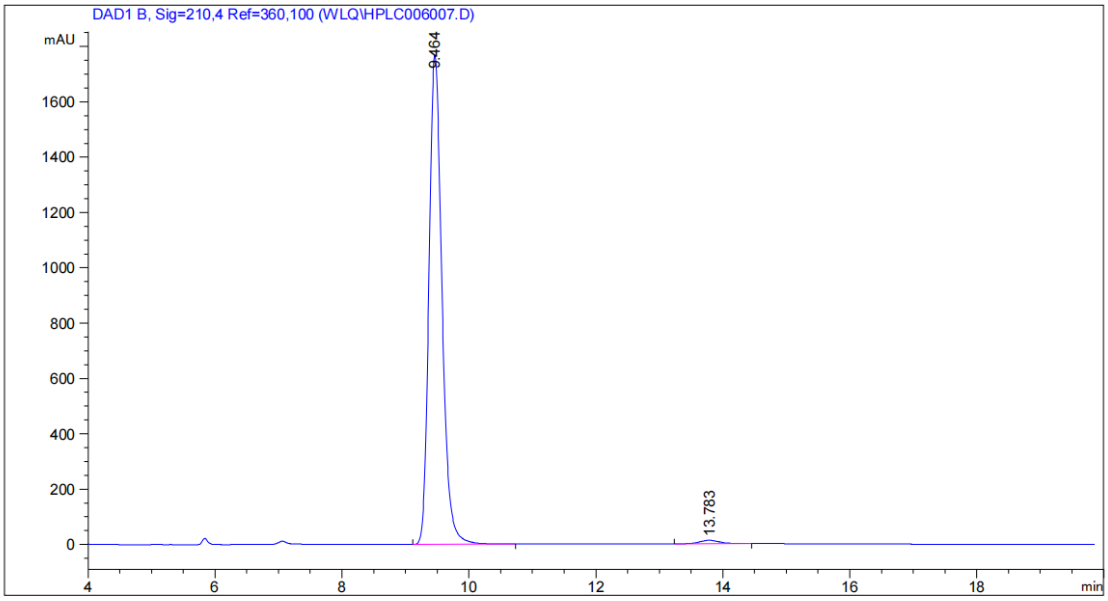

Peak #	Rt time [min]	Type	Width [min]	Peak Area [mAU*s]	Peak Height [mAU]	Peak Area %
1	9.464	BB	0.2172	2.50154e4	1767.02686	98.9493
2	13.783	BB	0.3320	265.62936	12.29739	1.0507

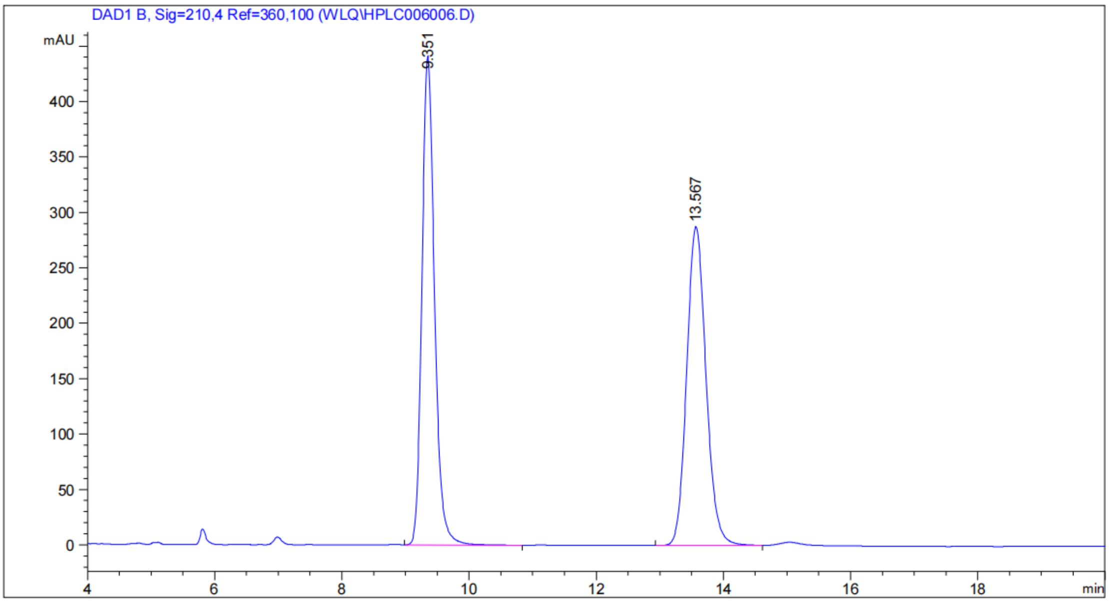

Peak #	Rt time [min]	Type	Width [min]	Peak Area [mAU*s]	Peak Height [mAU]	Peak Area %
1	9.351	BB	0.2124	6063.99609	441.07956	49.9977
2	13.567	BB	0.3279	6064.55566	287.73093	50.0023

HRMS (ESI) of diethyl (2*R*,5*S*)-3-(methylsulfonyl)-5-(naphthalen-1-yl)-2-phenyloxazolidine-4,4-dicarboxylate (3bm)

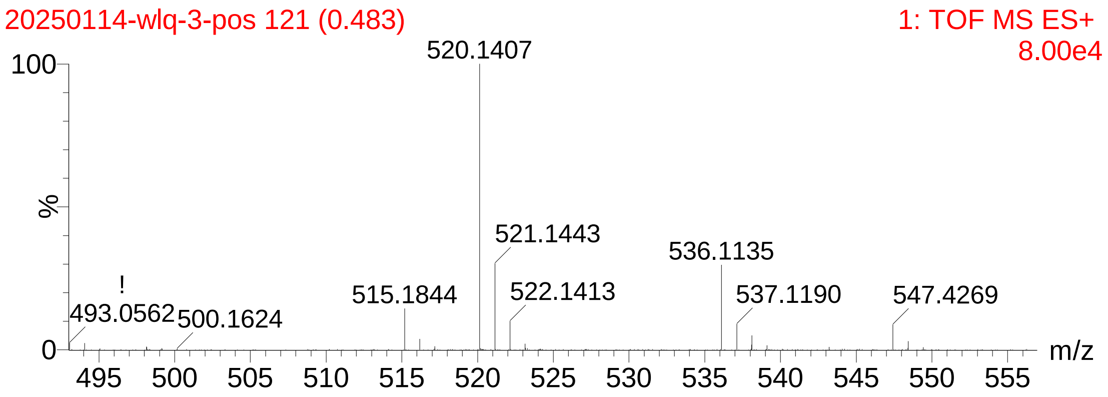

^{1}H NMR of diethyl (2*R*,5*S*)-3-(methylsulfonyl)-2-phenyl-5-(thiophen-2-yl)oxazolidine-4,4-dicarboxylate (3bn) (400 MHz, $CDCl_3$)

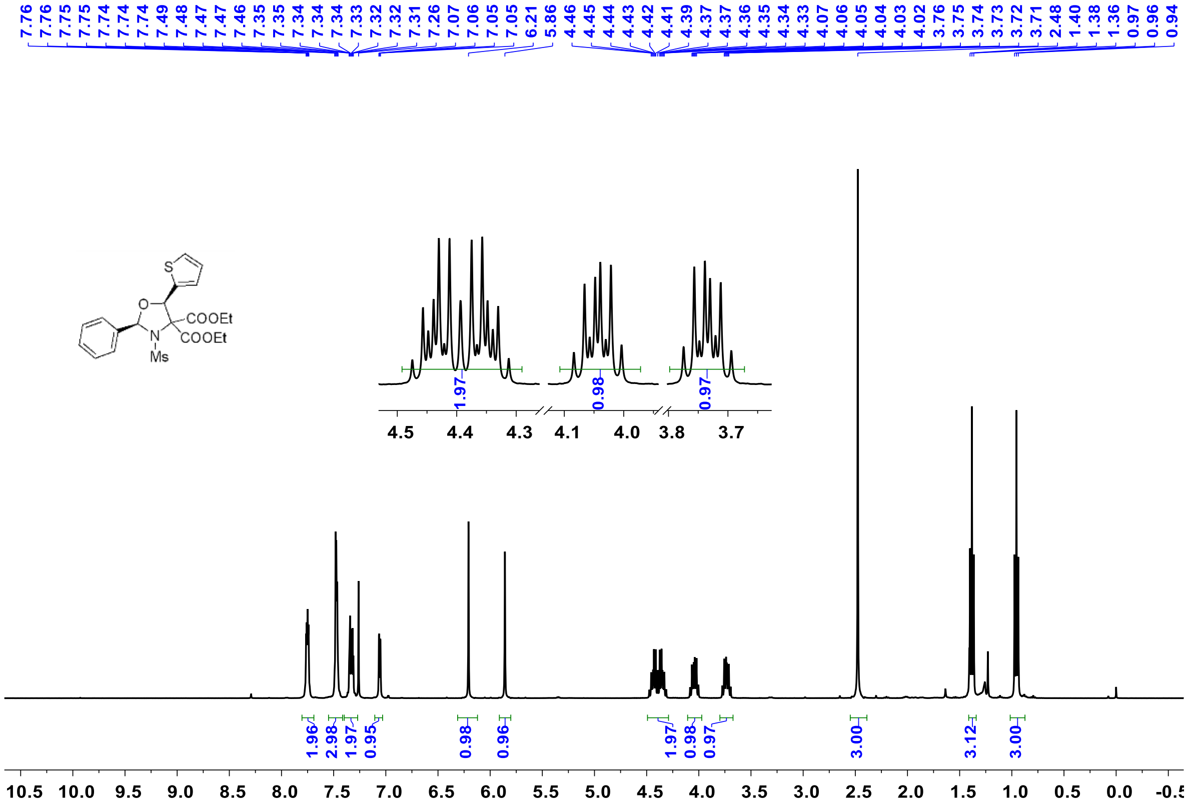

$^{13}C\{^{1}H\}$ NMR of diethyl (2*R*,5*S*)-3-(methylsulfonyl)-2-phenyl-5-(thiophen-2-yl)oxazolidine-4,4-dicarboxylate (3bn) (101 MHz, $CDCl_3$)

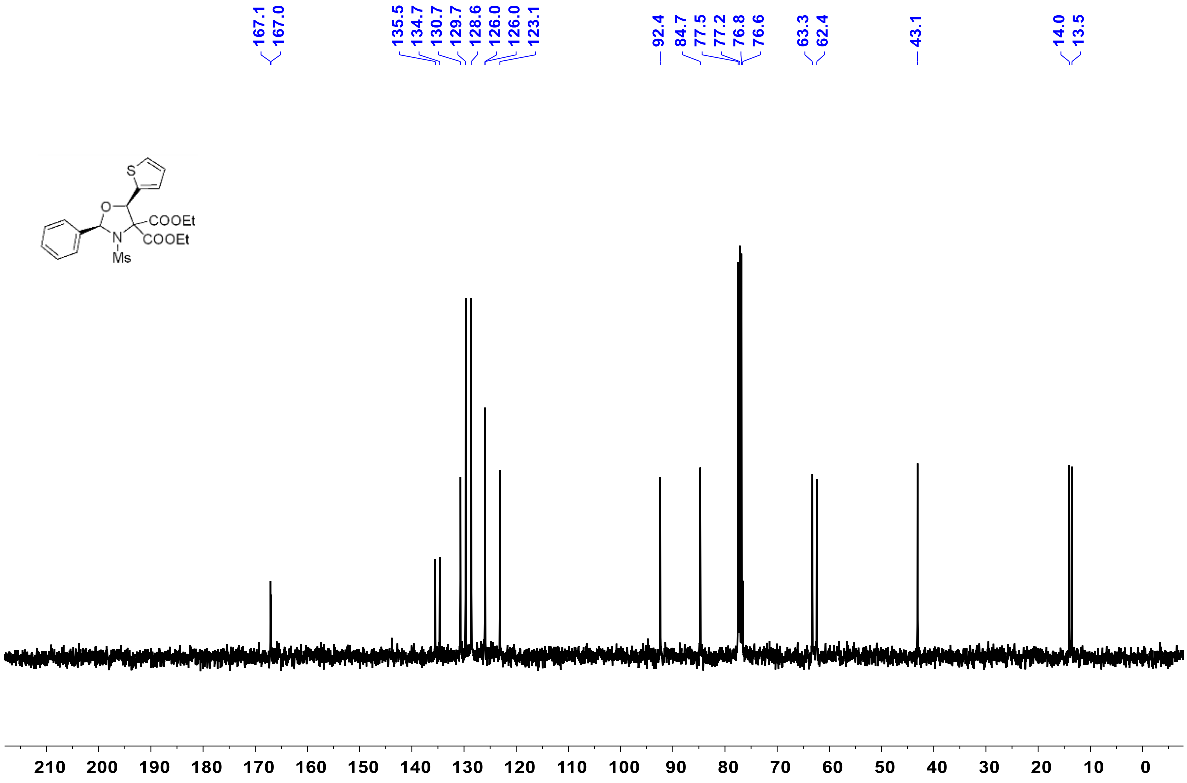

HPLC graph of racemic 3bn

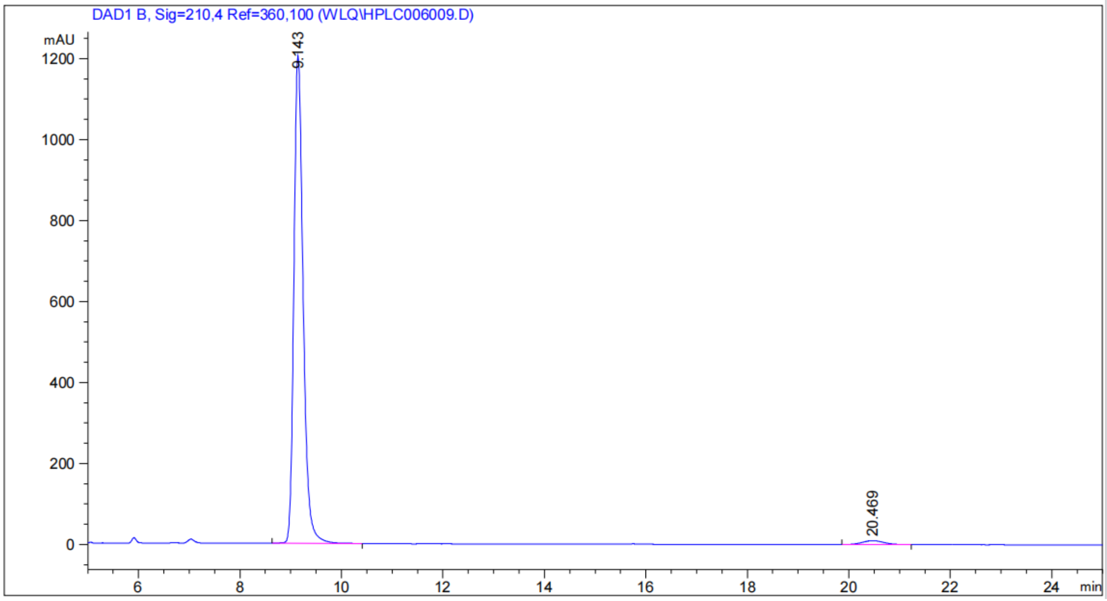

Peak #	Rt time [min]	Type	Width [min]	Peak Area [mAU*s]	Peak Height [mAU]	Peak Area %
1	9.143	BB	0.1934	1.52073e4	1203.98877	98.1518
2	20.469	BB	0.4570	286.35001	9.68245	1.8482

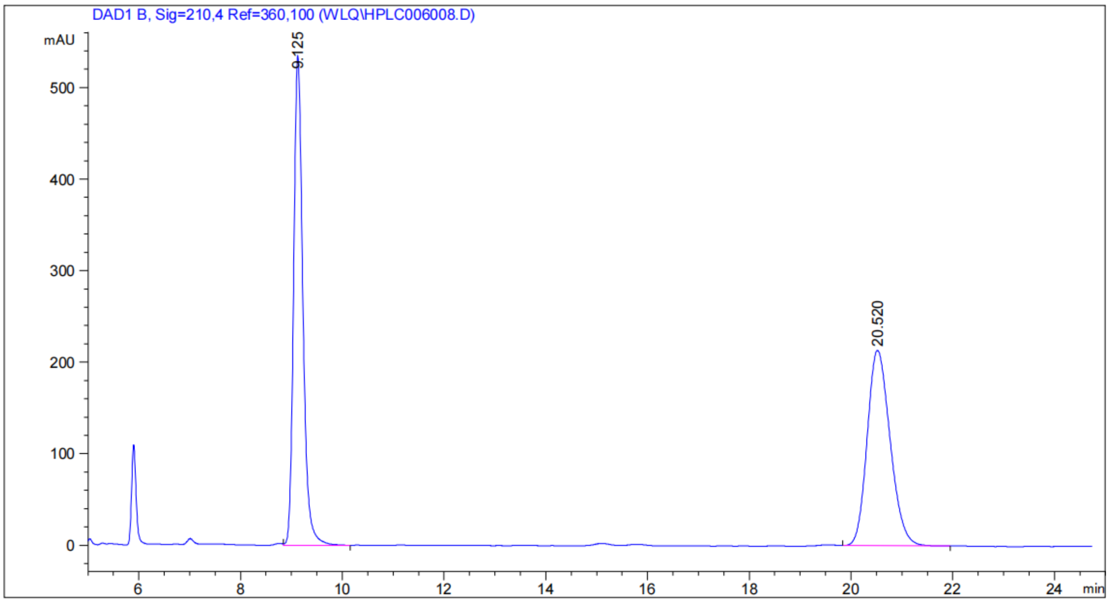

Peak #	Rt time [min]	Type	Width [min]	Peak Area [mAU*s]	Peak Height [mAU]	Peak Area %
1	9.125	VB	0.1936	6759.43701	534.43842	50.2376
2	20.520	BB	0.4857	6695.50635	213.58229	49.7624

HRMS (ESI) of diethyl (2*R*,5*S*)-3-(methylsulfonyl)-2-phenyl-5-(thiophen-2-yl)oxazolidine-4,4-dicarboxylate (3bn)

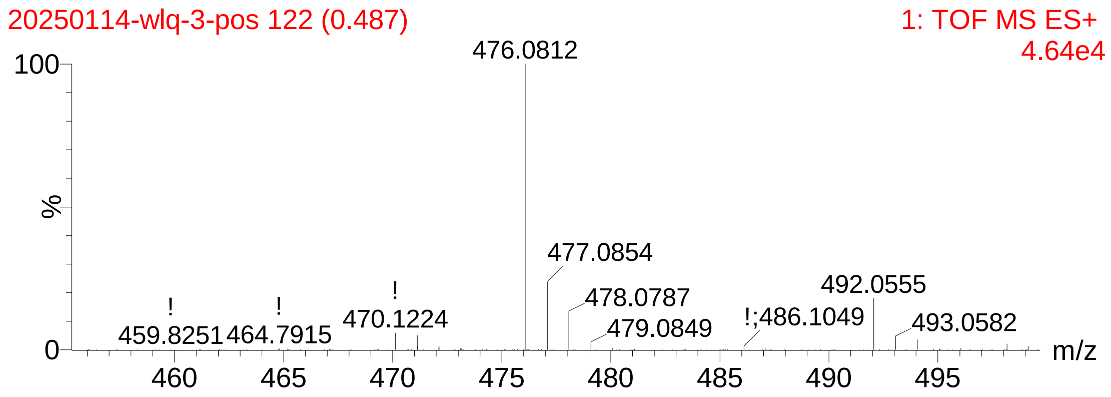

^{1}H NMR of diethyl (2*R*,5*S*)-5-((E)-4-bromostyryl)-3-(methylsulfonyl)-2-phenyloxazolidine-4,4-dicarboxylate (3bo) (400 MHz, $CDCl_3$)

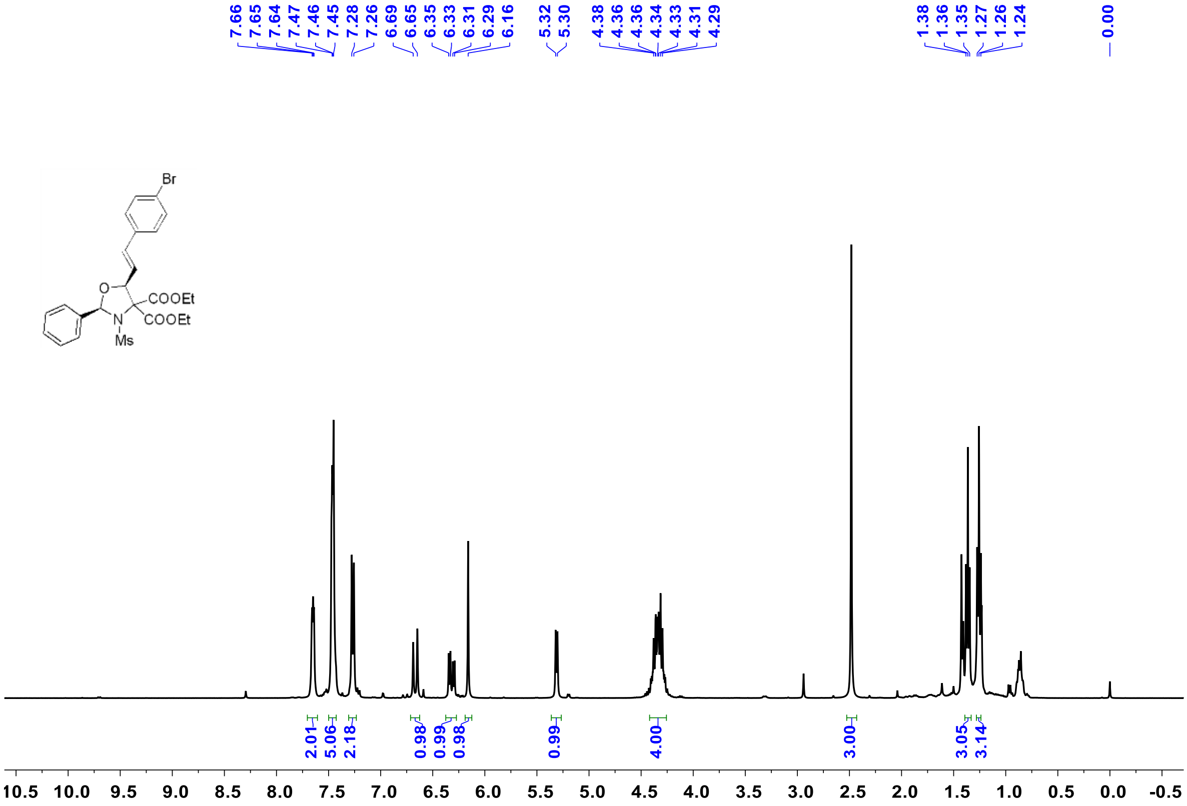

^{13}C{^{1}H} NMR of diethyl (2*R*,5*S*)-5-((E)-4-bromostyryl)-3-(methylsulfonyl)-2-phenyloxazolidine-4,4-dicarboxylate (3bo) (101 MHz, $CDCl_3$)

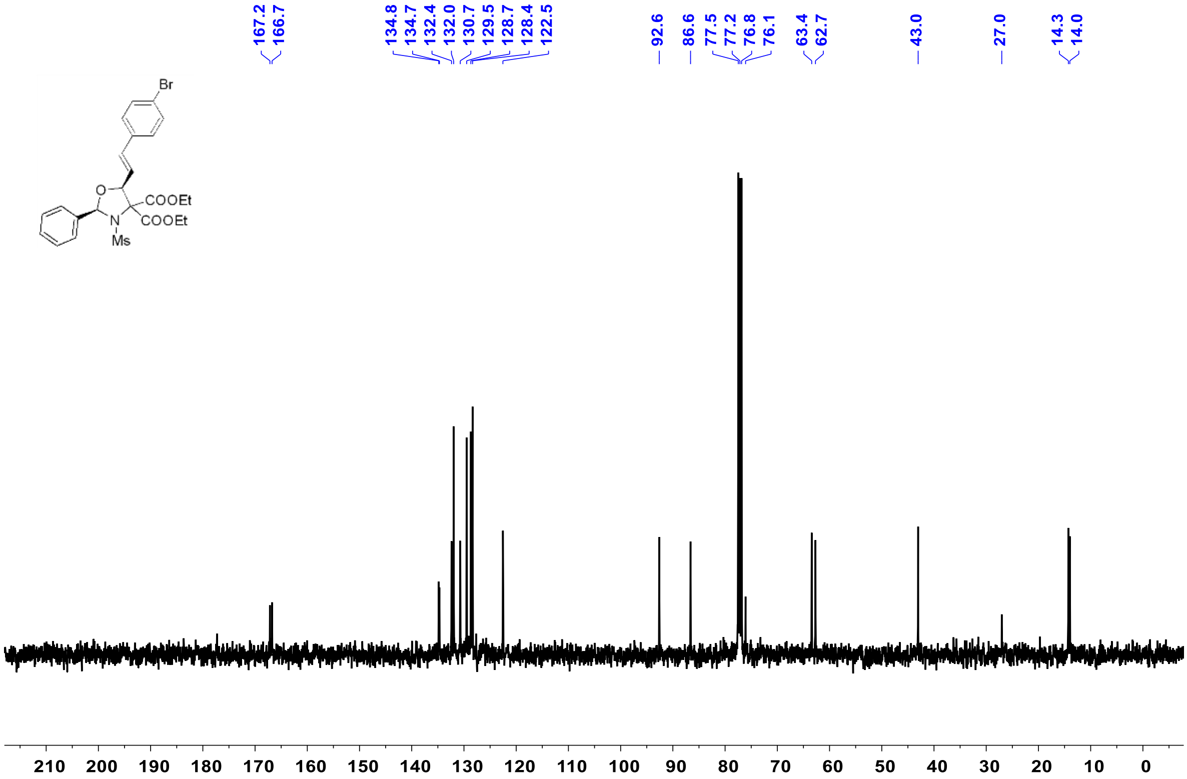

HPLC graph of racemic 3bo

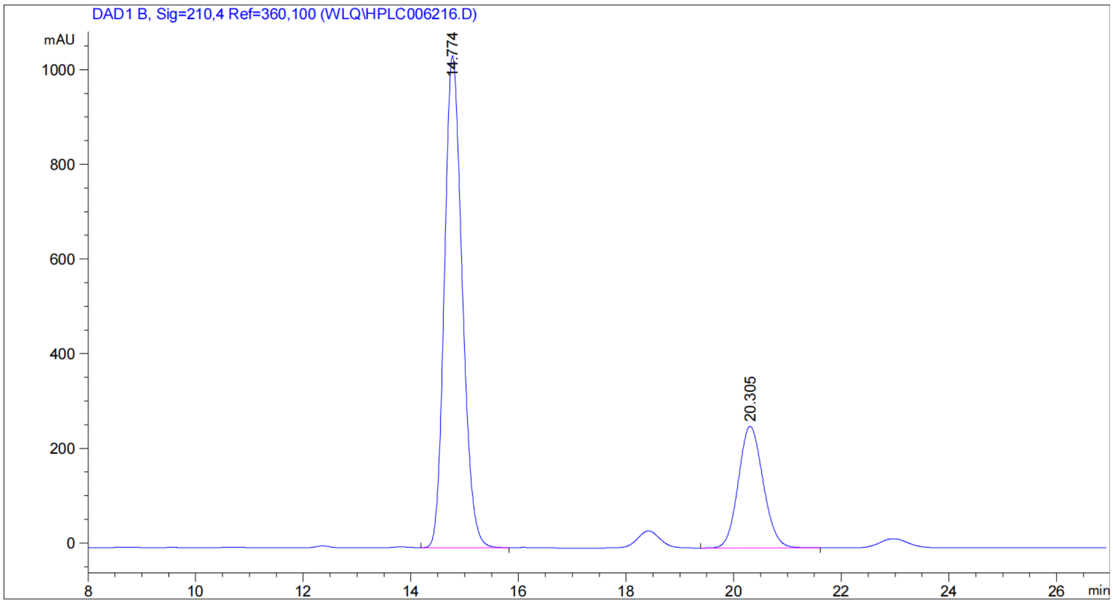

Peak #	Rt time [min]	Type	Width [min]	Peak Area [mAU*s]	Peak Height [mAU]	Peak Area %
1	14.774	BB	0.3566	2.39177e4	1038.86560	74.4004
2	20.305	BB	0.4995	8229.60059	257.08841	25.5996

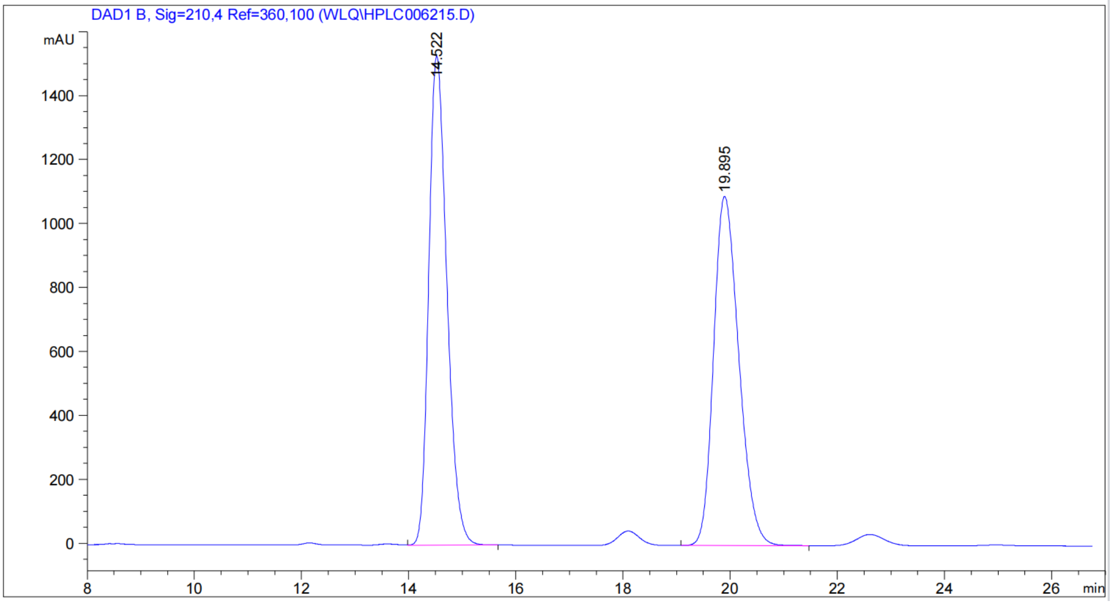

Peak #	Rt time [min]	Type	Width [min]	Peak Area [mAU*s]	Peak Height [mAU]	Peak Area %
1	14.522	VB	0.3632	3.58197e4	1529.43457	50.0736
2	19.895	BB	0.5055	3.57145e4	1092.22119	49.9264

HRMS (ESI) of diethyl (2*R*,5*S*)-5-((E)-4-bromostyryl)-3-(methylsulfonyl)-2-phenyloxazolidine-4,4-dicarboxylate (3bo)

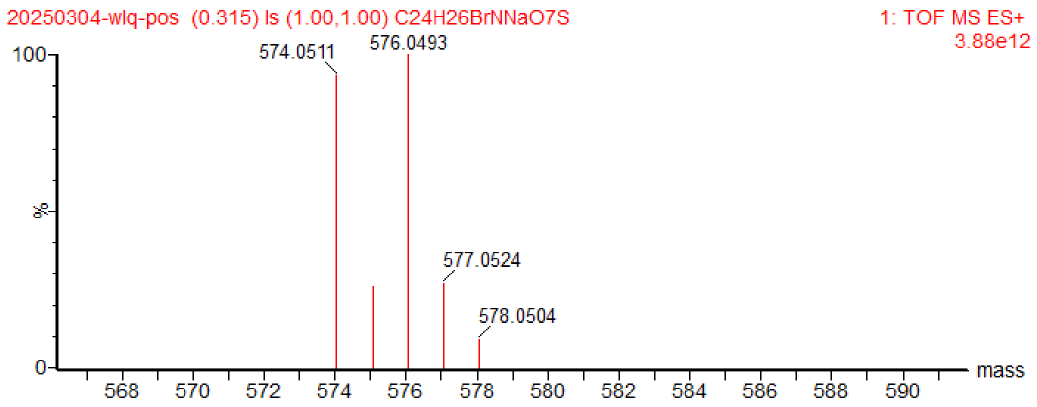

^{1}H NMR of diethyl (2*R*,5*S*)-2,5-diphenyl-3-(phenylsulfonyl)oxazolidine-4,4-dicarboxylate (3ca) (400 MHz, $CDCl_3$)

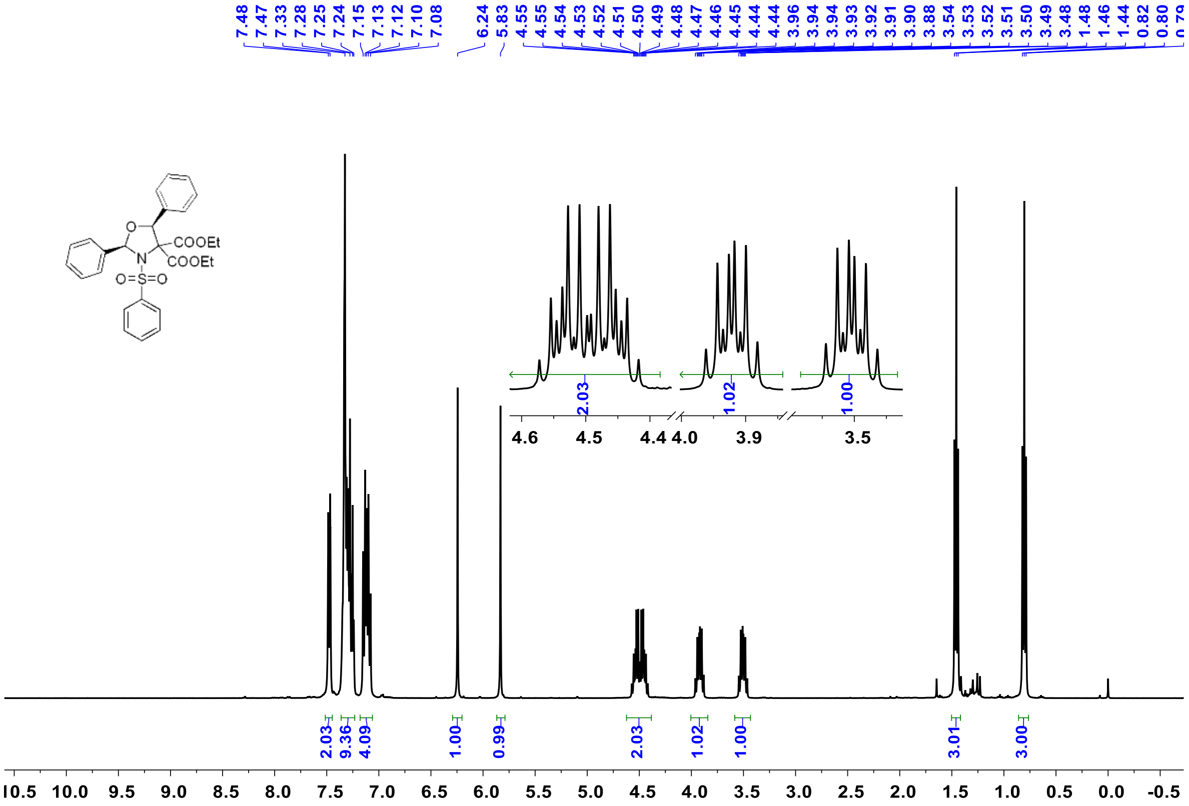

^{13}C{^{1}H} NMR of diethyl (2*R*,5*S*)-2,5-diphenyl-3-(phenylsulfonyl)oxazolidine-4,4-dicarboxylate (3ca) (101 MHz, $CDCl_3$)

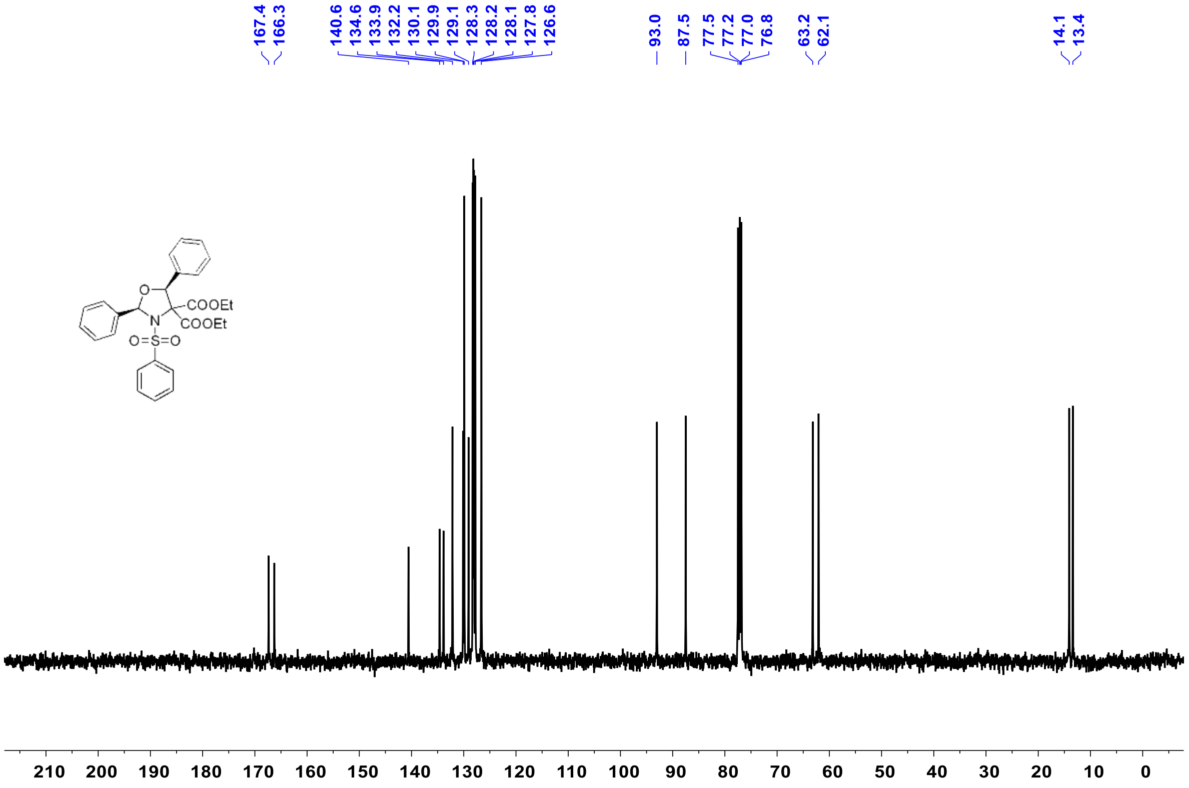

HPLC graph of racemic 3ca

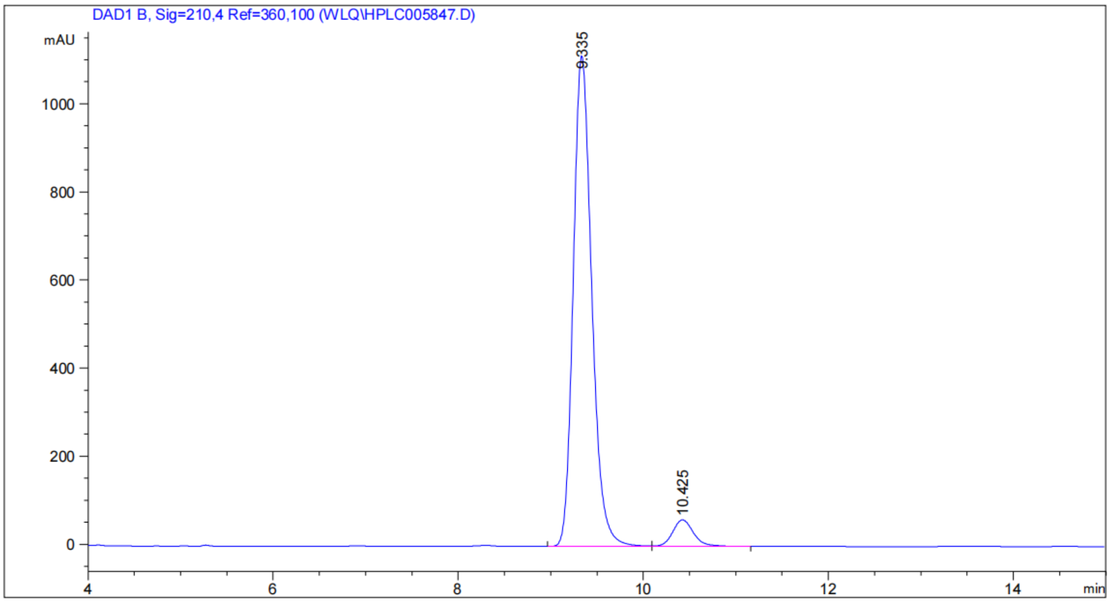

Peak #	Rt time [min]	Type	Width [min]	Peak Area [mAU*s]	Peak Height [mAU]	Peak Area %
1	9.335	BV	0.2135	1.54057e4	1113.04419	94.1921
2	10.425	VB	0.2419	949.92206	60.27952	5.8079

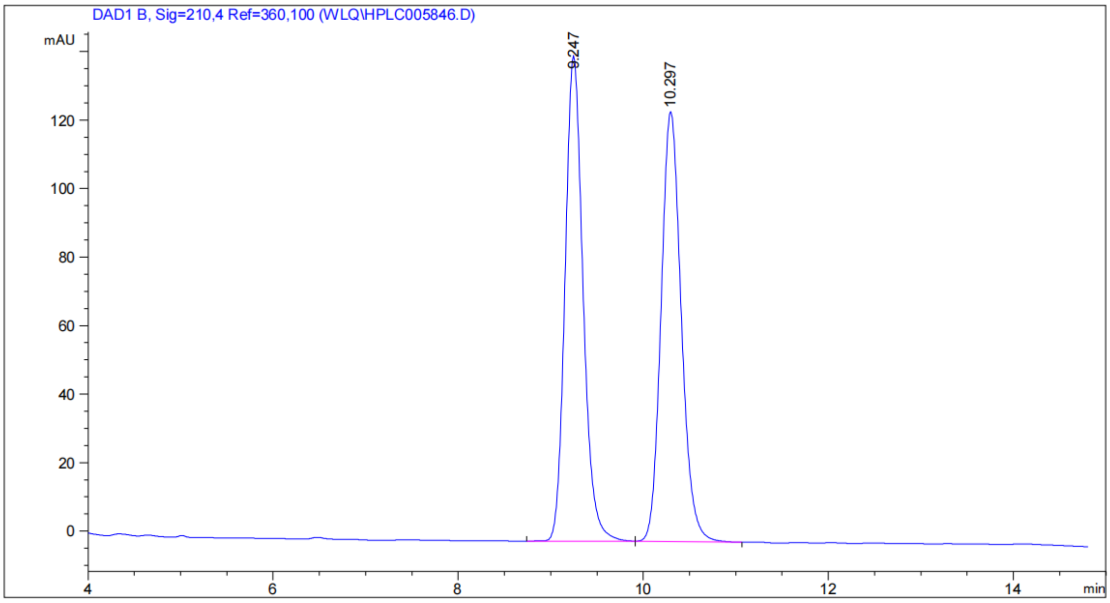

Peak #	Rt time [min]	Type	Width [min]	Peak Area [mAU*s]	Peak Height [mAU]	Peak Area %
1	9.247	BB	0.2072	1906.79297	141.55803	50.1395
2	10.297	BB	0.2323	1896.18225	125.47639	49.8605

^{1}H NMR of diethyl (2*R*,5*S*)-3-((4-chlorophenyl)sulfonyl)-2,5-diphenyloxazolidine-4,4-dicarboxylate (3da) (400 MHz, $CDCl_3$)

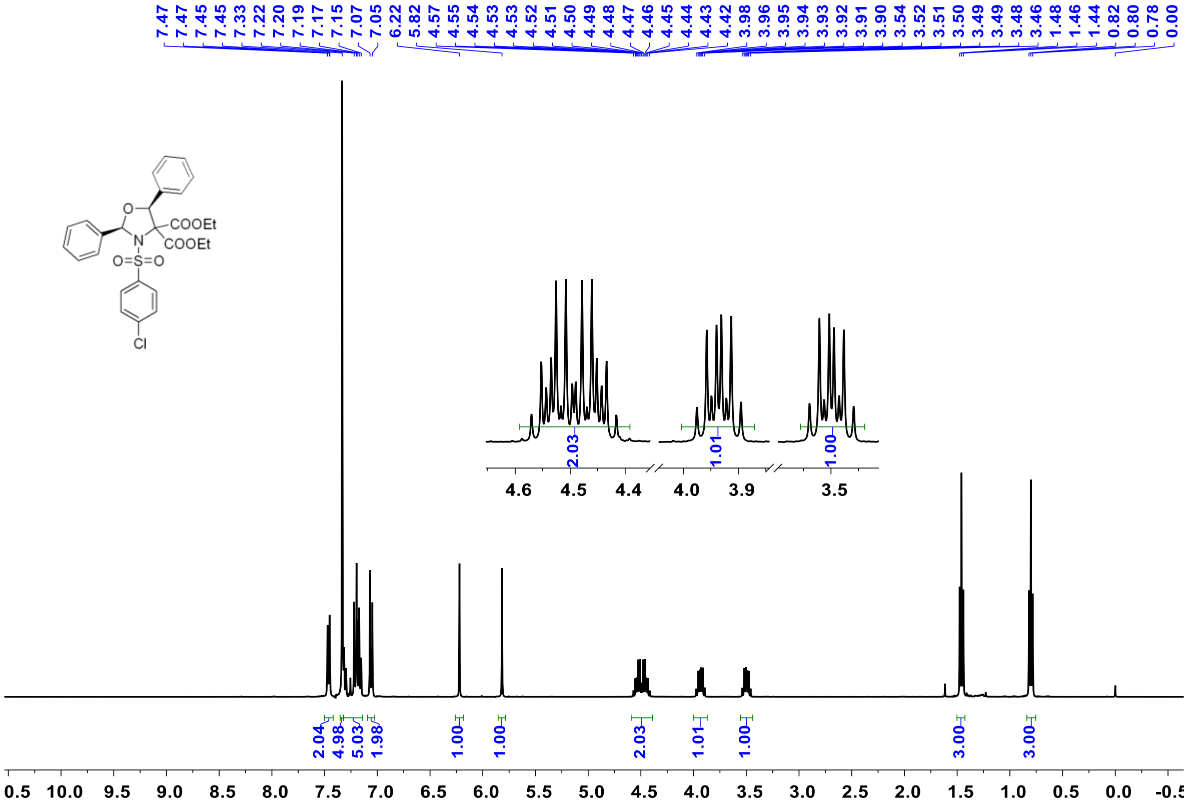

^{13}C{^{1}H} NMR of diethyl (2*R*,5*S*)-3-((4-chlorophenyl)sulfonyl)-2,5-diphenyloxazolidine-4,4-dicarboxylate (3da) (101 MHz, $CDCl_3$)

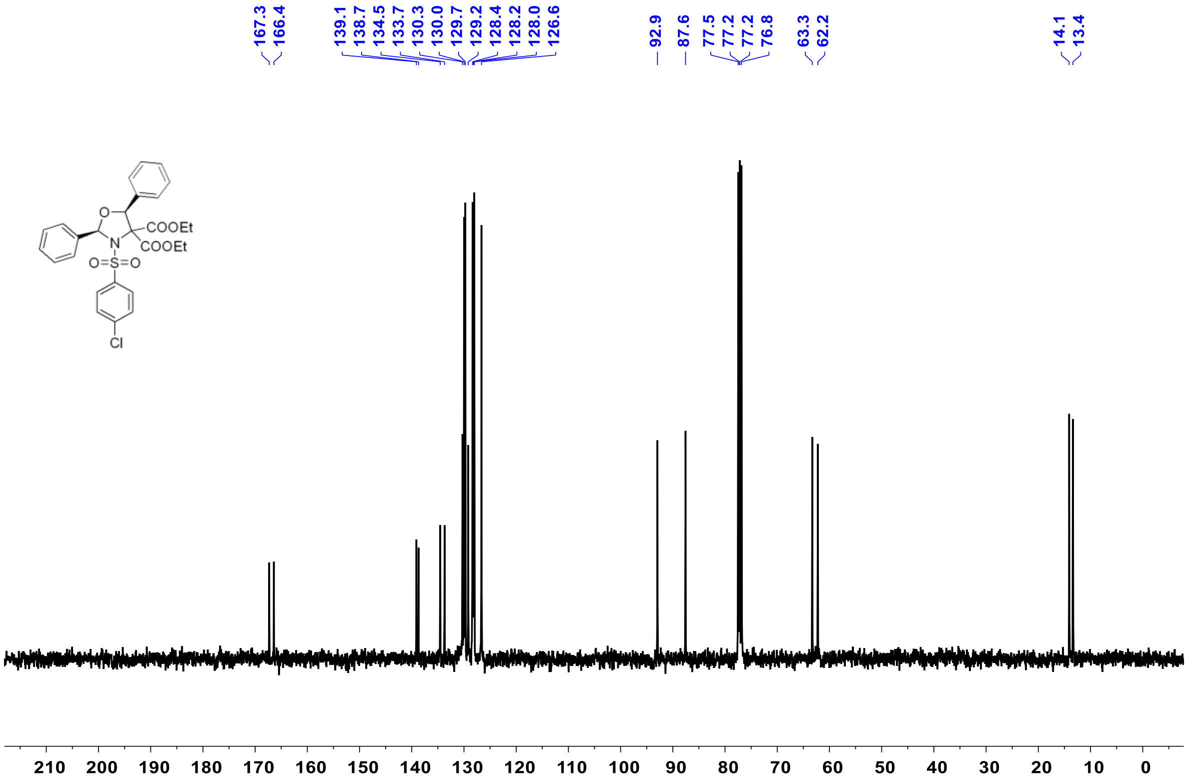

HPLC graph of racemic 3da

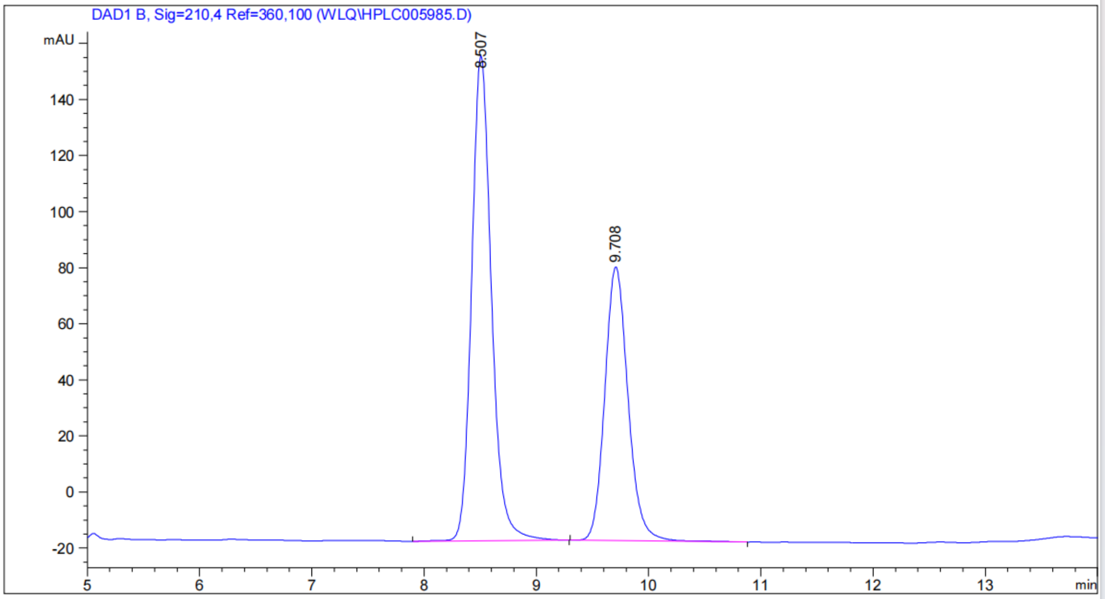

Peak #	Rt time [min]	Type	Width [min]	Peak Area [mAU*s]	Peak Height [mAU]	Peak Area %
1	8.507	BB	0.1890	2120.44141	172.94016	60.1663
2	9.708	BB	0.2218	1403.85986	97.61032	39.8337

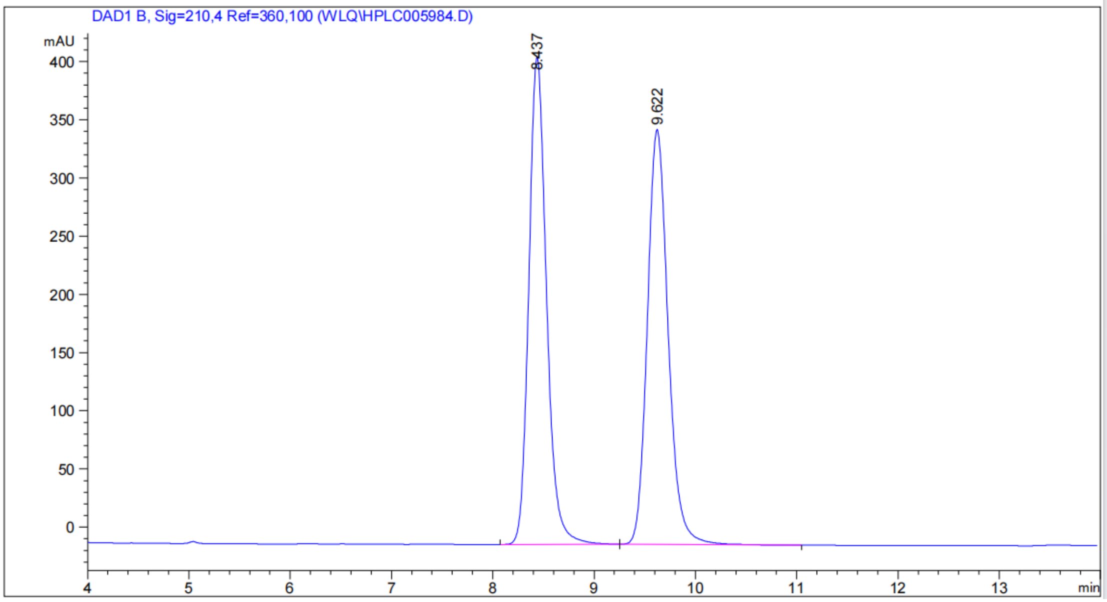

Peak #	Rt time [min]	Type	Width [min]	Peak Area [mAU*s]	Peak Height [mAU]	Peak Area %
1	8.437	BB	0.1855	5077.75146	418.71432	49.9916
2	9.622	BB	0.2202	5079.46729	356.63943	50.0084

^{1}H NMR of dimethyl (2*R*,5*S*)-5-(4-bromophenyl)-3-(methylsulfonyl)-2-phenyloxazolidine-4,4-dicarboxylate (3ef) (400 MHz, $CDCl_3$)

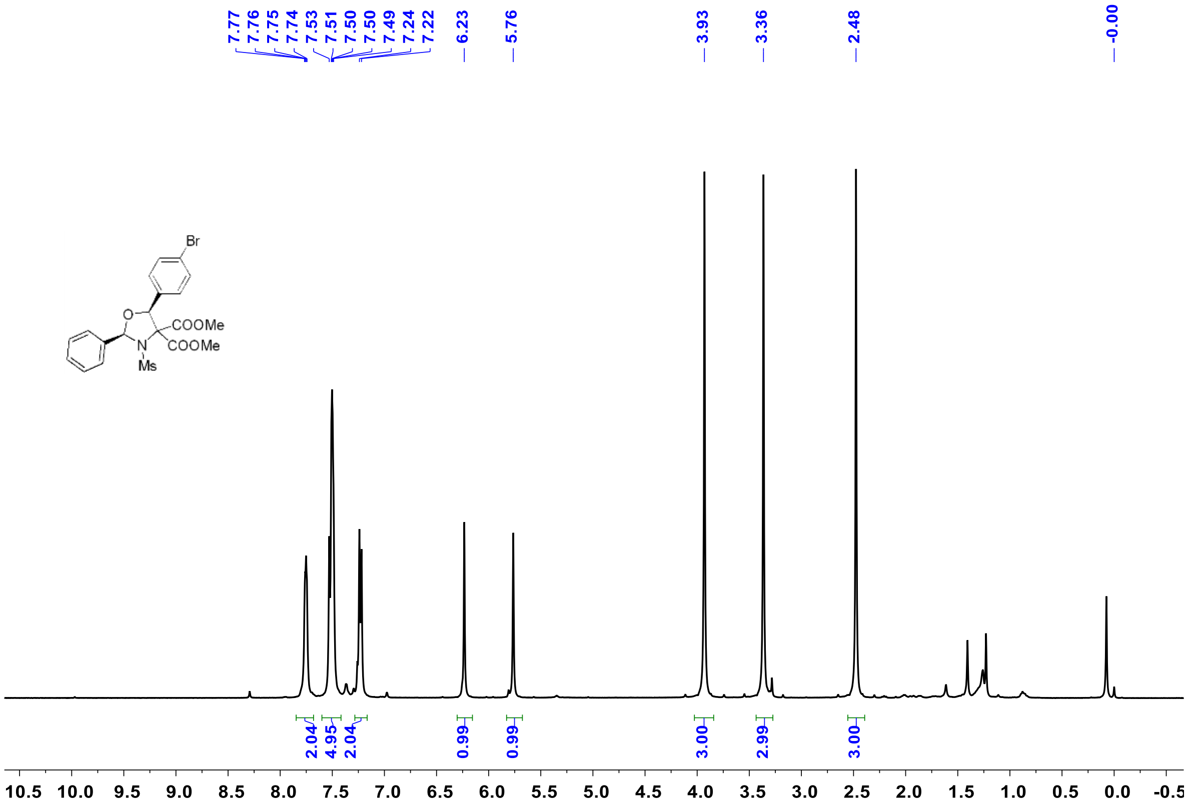

^{13}C{^{1}H} NMR of dimethyl (2*R*,5*S*)-5-(4-bromophenyl)-3-(methylsulfonyl)-2-phenyloxazolidine-4,4-dicarboxylate (3ef) (101 MHz, $CDCl_3$)

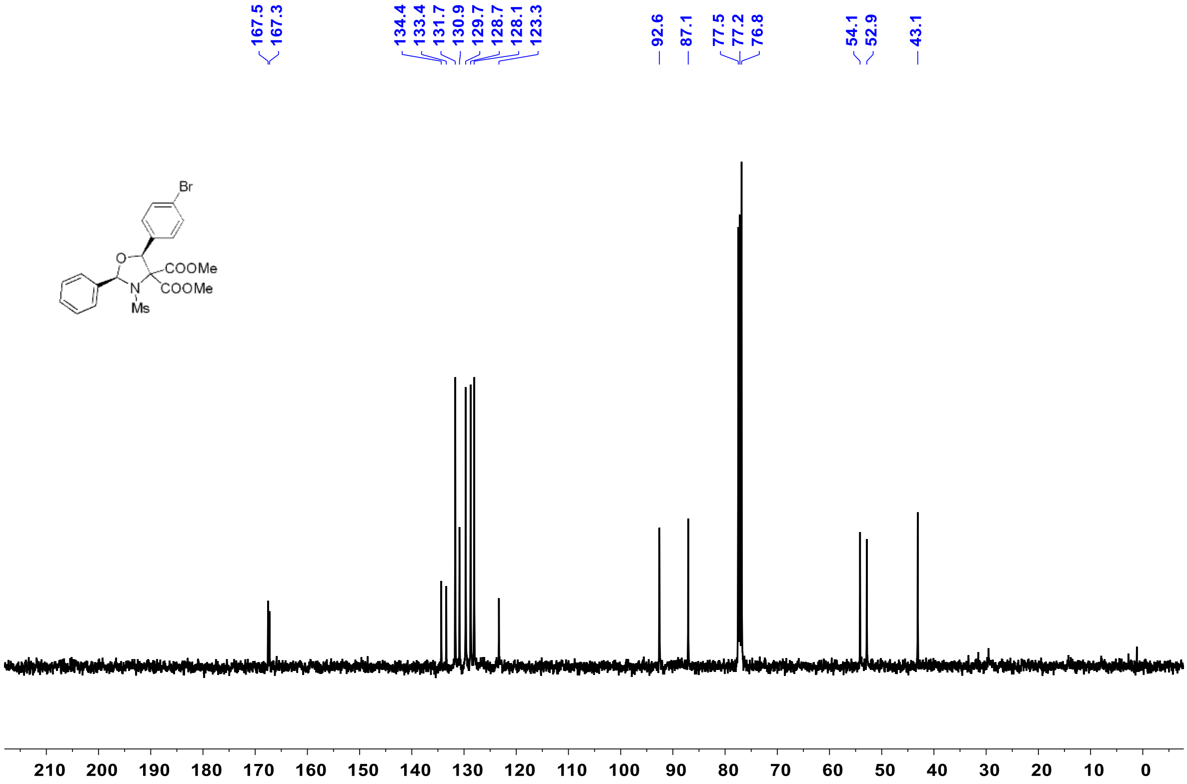

HPLC graph of racemic 3ef

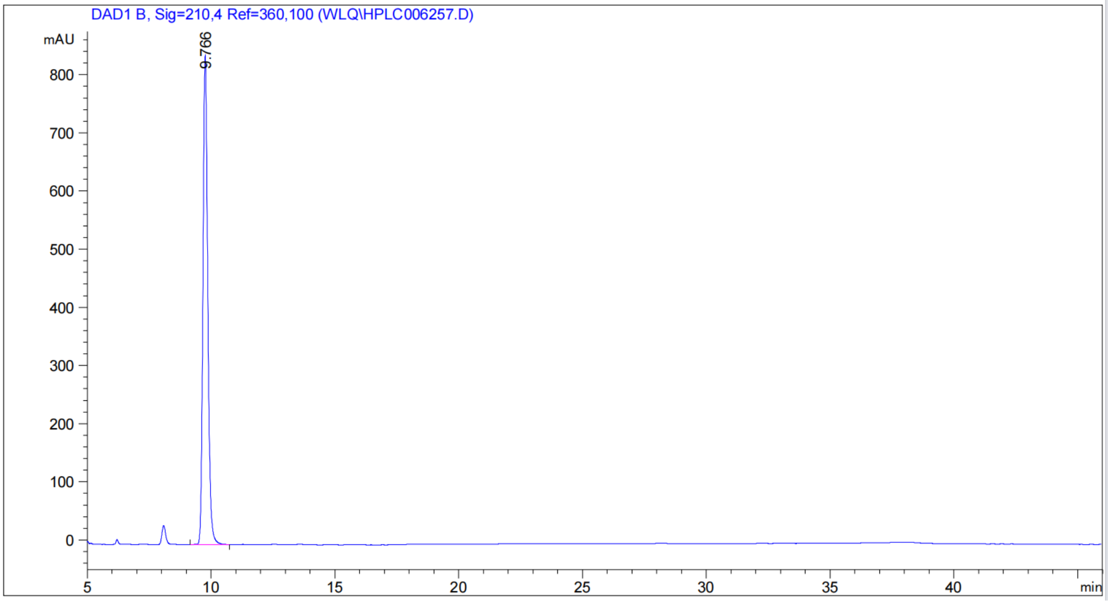

Peak #	Rt time [min]	Type	Width [min]	Peak Area [mAU*s]	Peak Height [mAU]	Peak Area %
1	9.766	BB	0.2086	1.14284e4	840.67926	100.0000

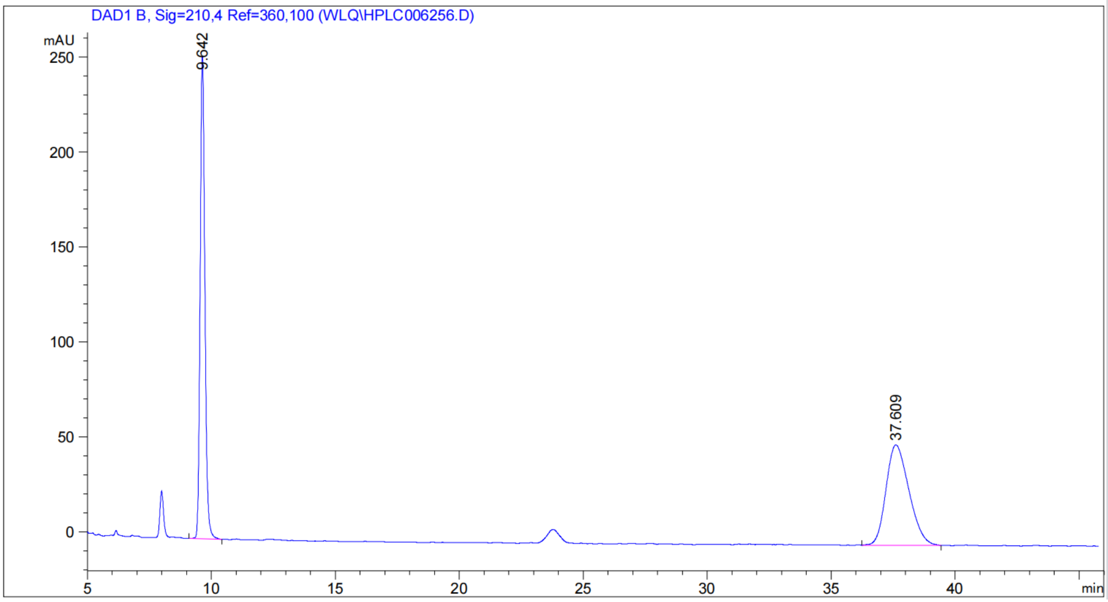

Peak #	Rt time [min]	Type	Width [min]	Peak Area [mAU*s]	Peak Height [mAU]	Peak Area %
1	9.642	BB	0.2054	3384.40942	254.04614	49.9203
2	37.609	BB	1.0034	3395.21313	52.85326	50.0797

HRMS (ESI) of dimethyl (2*R*,5*S*)-5-(4-bromophenyl)-3-(methylsulfonyl)-2-phenyloxazolidine-4,4-dicarboxylate (3ef)

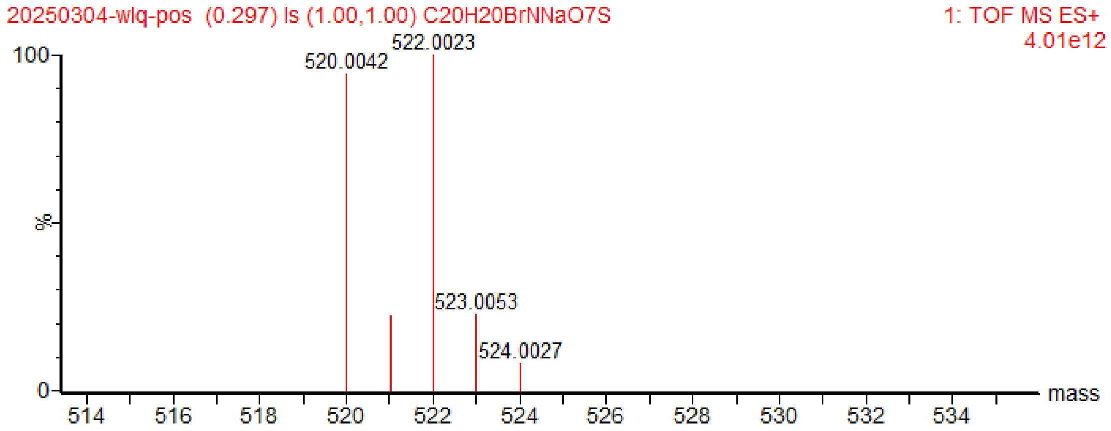

^{1}H NMR of diisopropyl (2*R*,5*S*)-5-(4-bromophenyl)-3-(methylsulfonyl)-2-phenyloxazolidine-4,4-dicarboxylate (3ff) (400 MHz, $CDCl_3$)

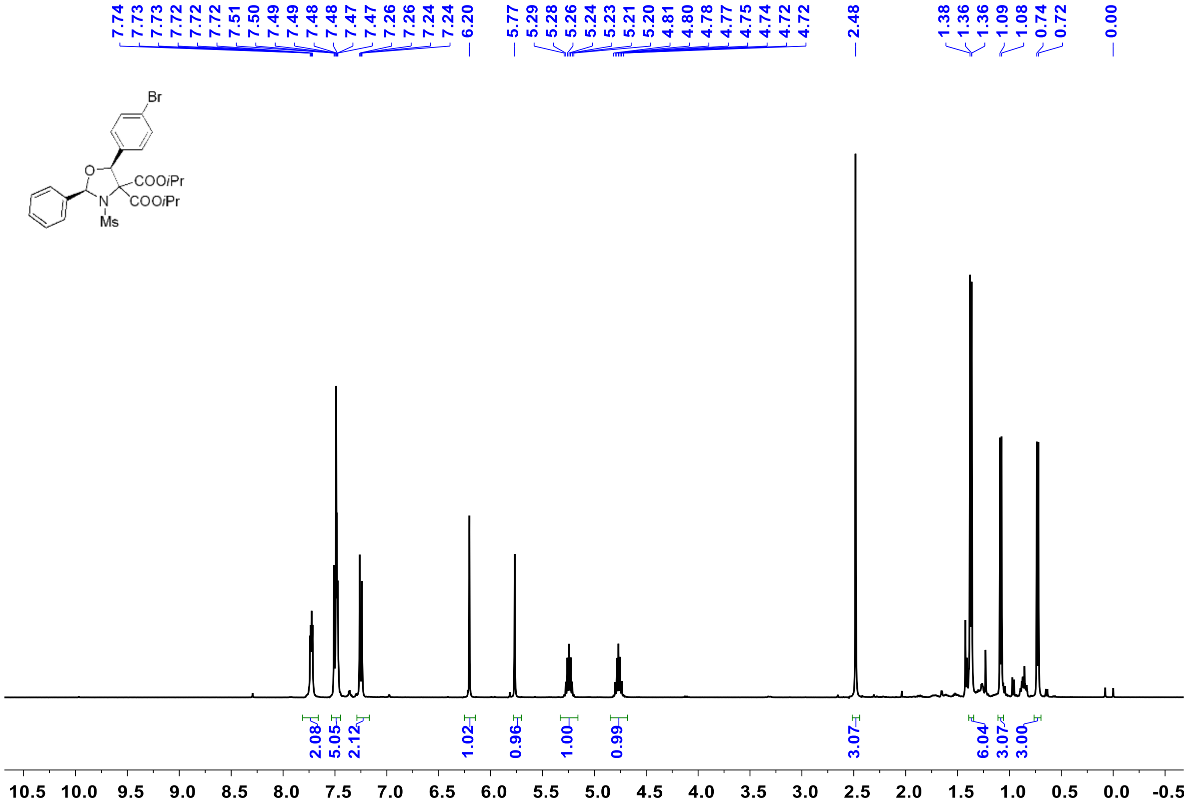

^{13}C{^{1}H} NMR of diisopropyl (2*R*,5*S*)-5-(4-bromophenyl)-3-(methylsulfonyl)-2-phenyloxazolidine-4,4-dicarboxylate (3ff) (101 MHz, $CDCl_3$)

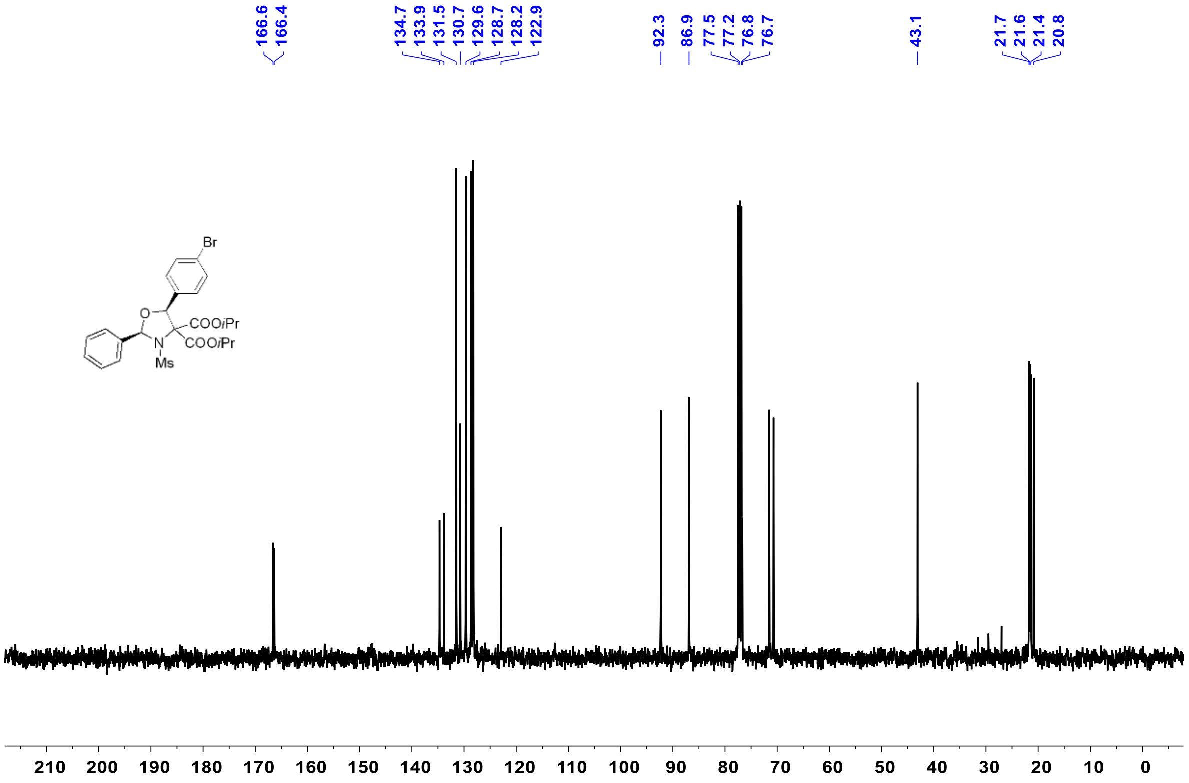

HPLC graph of racemic 3ff

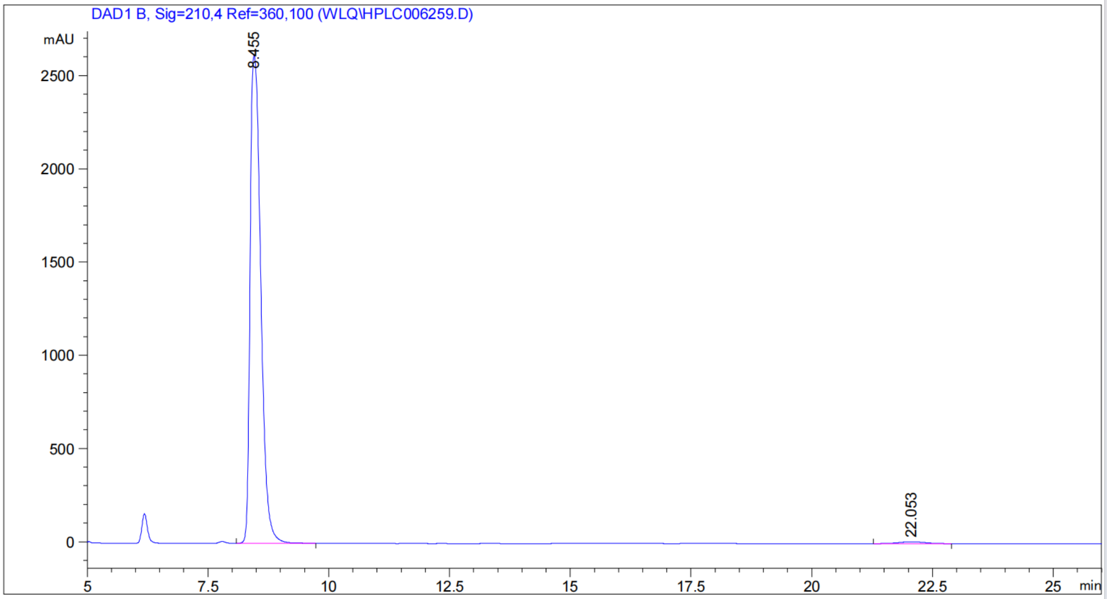

Peak #	Rt time [min]	Type	Width [min]	Peak Area [mAU*s]	Peak Height [mAU]	Peak Area %
1	8.455	BB	0.2407	4.00890e4	2616.90747	99.1087
2	22.053	BB	0.6017	360.54666	9.52316	0.8913

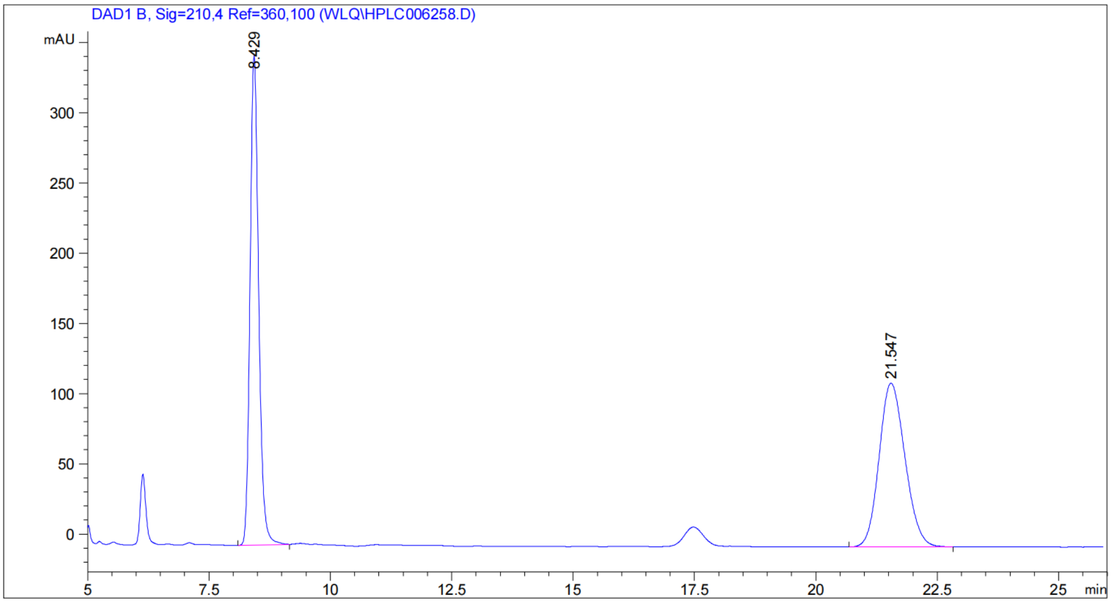

Peak #	Rt time [min]	Type	Width [min]	Peak Area [mAU*s]	Peak Height [mAU]	Peak Area %
1	8.429	BB	0.1897	4292.60986	348.57767	49.8746
2	21.547	BB	0.5775	4314.20068	116.64846	50.1254

HRMS (ESI) of diisopropyl (2*R*,5*S*)-5-(4-bromophenyl)-3-(methylsulfonyl)-2-phenyloxazolidine-4,4-dicarboxylate (3ff)

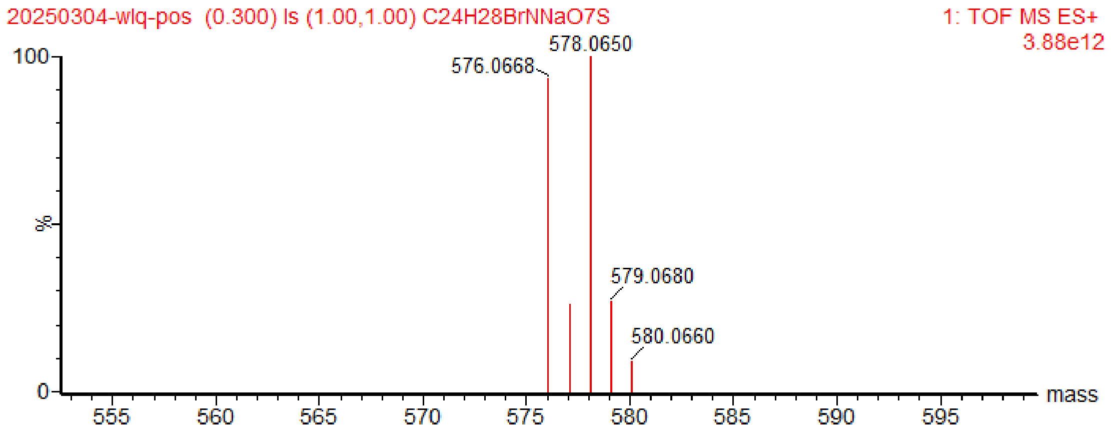

^{1}H NMR of diethyl (2*R*,5*S*)-2-(4-bromophenyl)-5-phenyl-3-tosyloxazolidine-4,4-dicarboxylate (3ga) (400 MHz, $CDCl_3$)

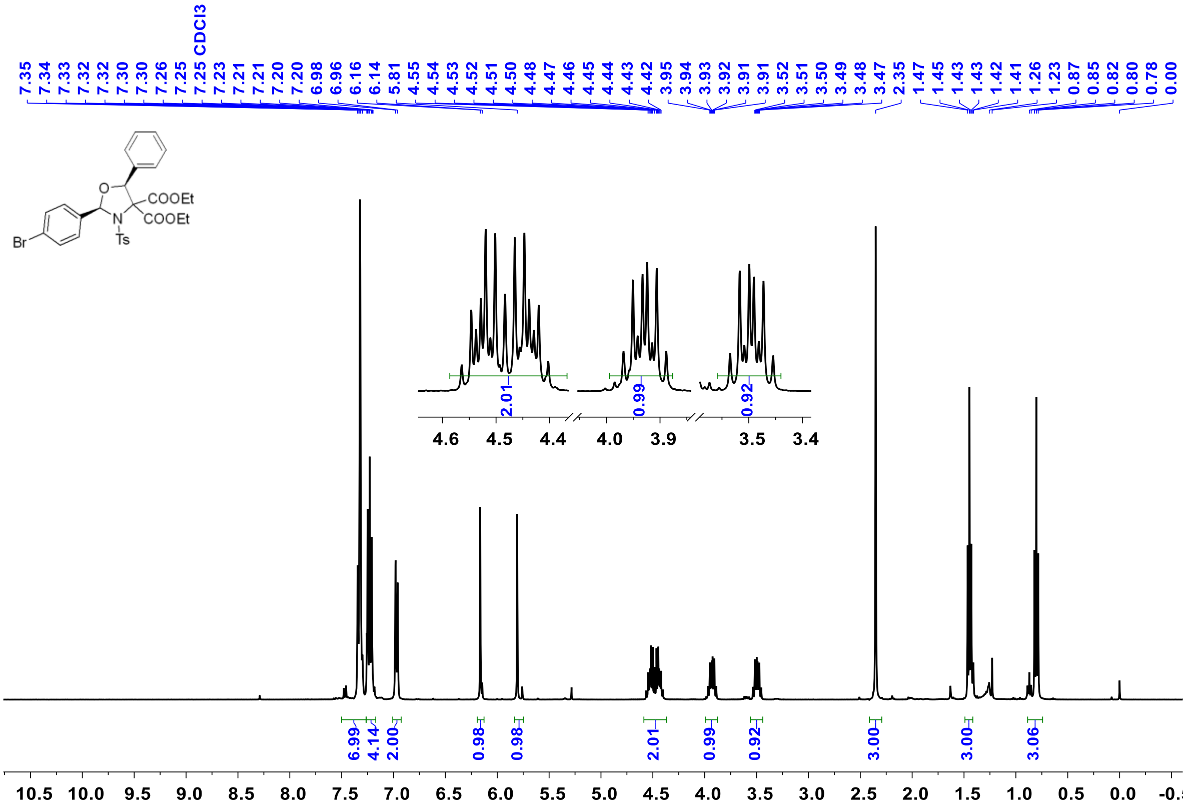

^{13}C{^{1}H} NMR of diethyl (2*R*,5*S*)-2-(4-bromophenyl)-5-phenyl-3-tosyloxazolidine-4,4-dicarboxylate (3ga) (101 MHz, $CDCl_3$)

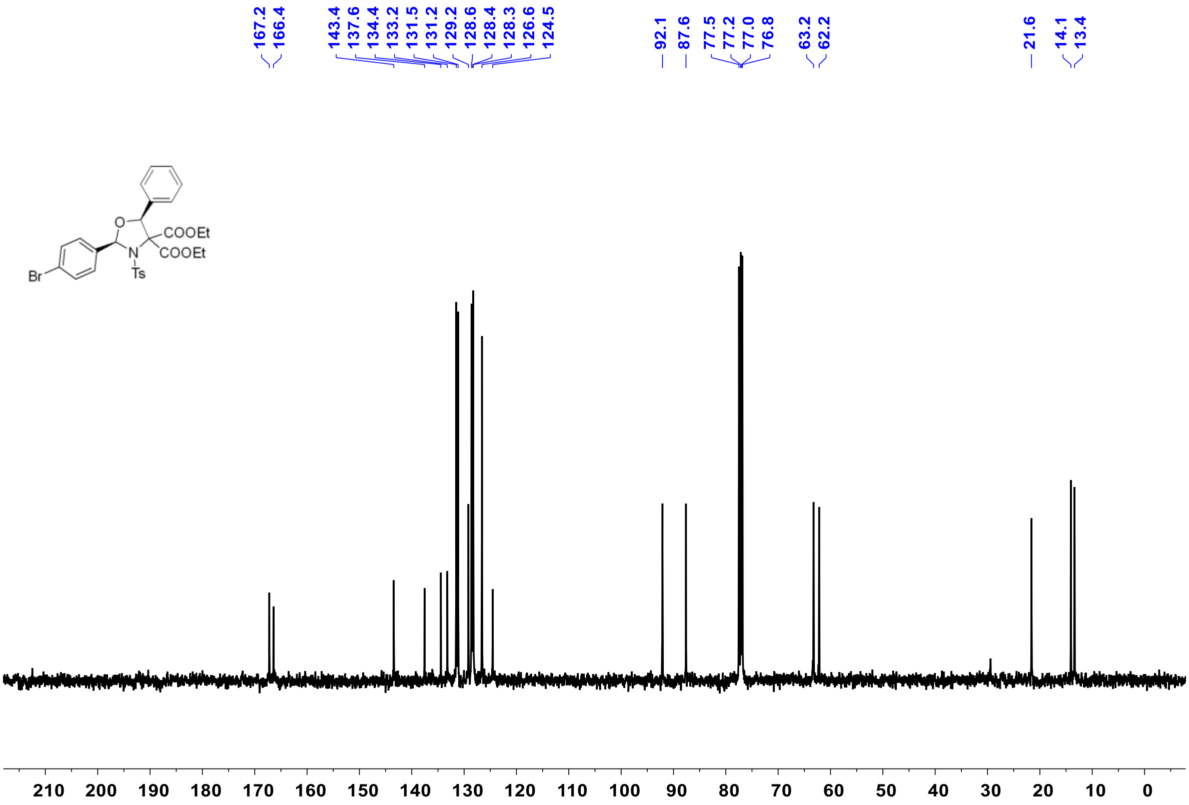

HPLC graph of racemic 3ga

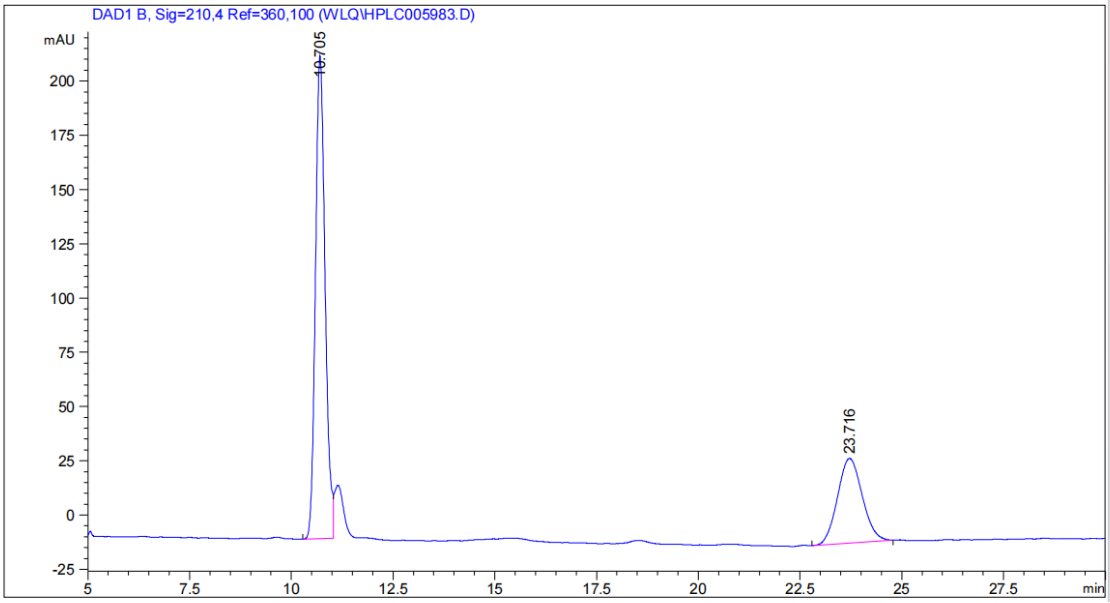

Peak #	Rt time [min]	Type	Width [min]	Peak Area [mAU*s]	Peak Height [mAU]	Peak Area %
1	10.705	BV	0.2537	3655.59546	222.46999	68.9237
2	23.716	BB	0.6506	1648.23315	39.03730	31.0763

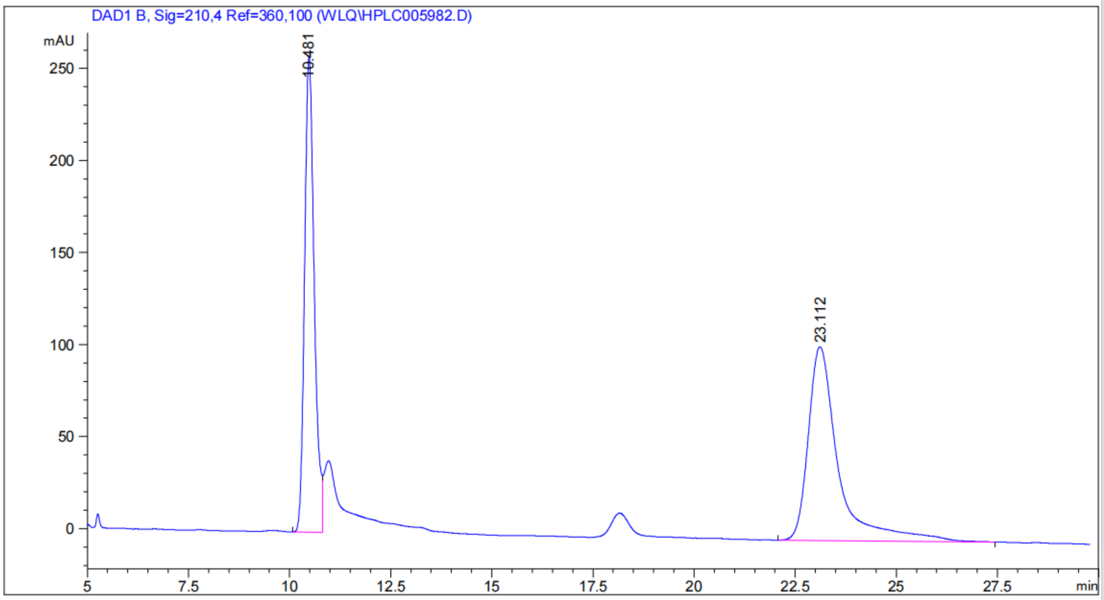

Peak #	Rt time [min]	Type	Width [min]	Peak Area [mAU*s]	Peak Height [mAU]	Peak Area %
1	10.481	BV	0.2536	4284.74805	258.23804	43.9936
2	23.112	BB	0.7645	5454.73975	105.27739	56.0064

HRMS (ESI) of diethyl (2*R*,5*S*)-2-(4-bromophenyl)-5-phenyl-3-tosyloxazolidine-4,4-dicarboxylate (3ga)

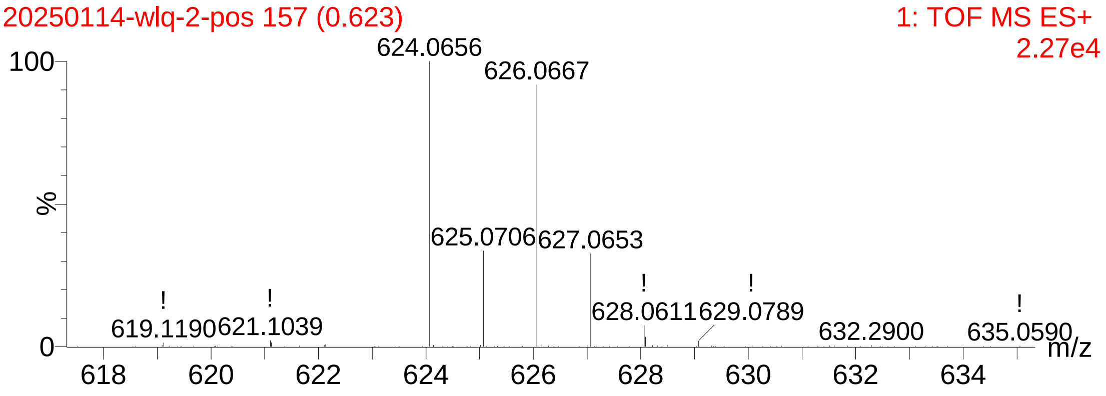

Supplement: File 1 — Analytic data and copies of 1H and 13C NMR spectra of compounds 1 and 3, copies of HRMS spectra of unknown compound 3 and copies of HLPC profiles of compounds 3. [file Beilstein_J_Org_Chem-21-1087-s001.pdf]
